# Supplementary material for: Hypoxia promotes tumor immune evasion by suppressing MHC-I expression and antigen presentation
Source: EMBO J. 2025 Jan 3;44(3):903–22. doi: 10.1038/s44318-024-00319-7 (PMC11790895; doi:10.1038/s44318-024-00319-7)
Supplement: Supplementary file 1 — Appendix [file 44318_2024_319_MOESM1_ESM.pdf]

Hypoxia promotes tumor immune evasion by suppressing MHC-I expression and antigen presentation

Hala Estephan[1], Arun Tailor[2], Robert Parker[2], McKenzie Kreamer [3], Ioanna Papandreou[3], Leticia Campo[1], Alistair Easton[1], Eui Jung Moon[1], Nicholas C. Denko[3], Nicola Ternette[2], Ester M. Hammond[1], Amato J. Giaccia[1*]

[1] Department of Oncology, The University of Oxford, Oxford, OX3 7DQ, UK

[2] Centre for Immuno-Oncology, Nuffield Department of Medicine, University of Oxford, Oxford OX37BN, UK

[3] Department of Radiation Oncology, OSU Wexner Medical Center, James Cancer Hospital and Solove Research Institute, Ohio State University, Columbus, OH, USA.

* Corresponding author

amato.giaccia@oncology.ox.ac.uk

Table of contents

Appendix Figure S1	page 3
Appendix Figure S2	page 4
Appendix Figure S3	page 5
Appendix Figure S4	page 6
Appendix Figure S5	page 7
Appendix Figure S6	page 8
Appendix Figure S7	page 9
Appendix Figure S8	page 10
Appendix Figure S9	page 11
Appendix Figure S10	page 12

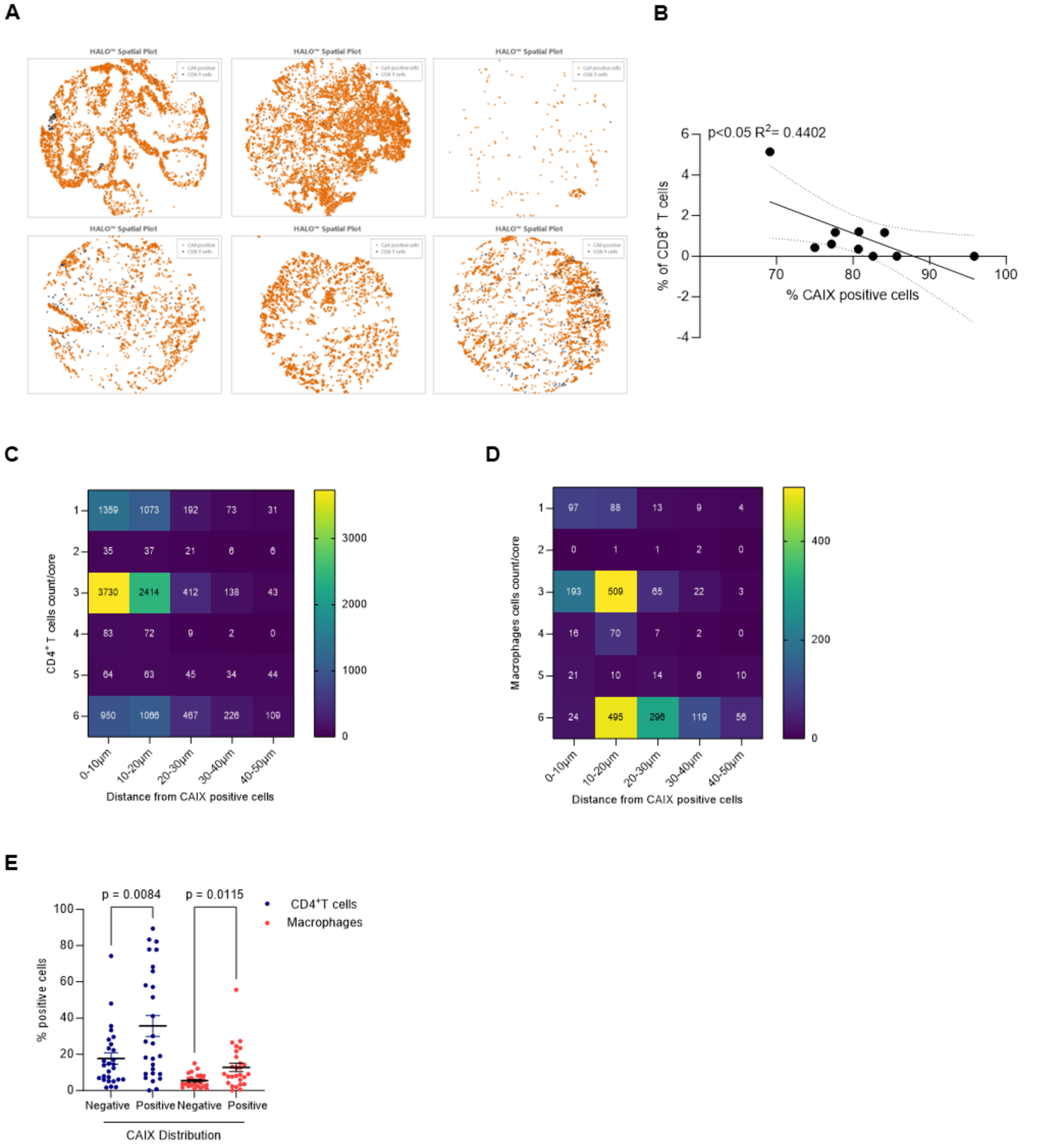

Appendix Fig.S1 Hypoxia correlates with reduced CD8⁺ T cells infiltration.

(**A**) Spatial plot generated using Halo® software showing the distribution of CAIX and CD8⁺T positive cells in 6 different tissue microarray from patients with colorectal cancer, further proximity analysis was performed on the same cores and is presented in figure 1C. (**B**) Machine learning-based quantification of CAIX and CD8⁺T positive cells following multiplex staining, graph showing negative correlation between CD8⁺T cells and CAIX positive cells. Statistical analysis was determined using simple linear regression test and Pearson correlation. (**C**) Heatmap showing the number of CD4⁺T cells distribution into the tissue based on the distance from CAIX positive areas. (**D**) Heatmap showing the number of macrophages distribution into the tissue based on the distance from CAIX positive areas. (**E**) Graph showing the distribution of CD4⁺ T cells and macrophages in CAIX negative and positive areas in different tissue microarrays from patients with colorectal cancer. Statistical analysis was determined using One-way Anova test.

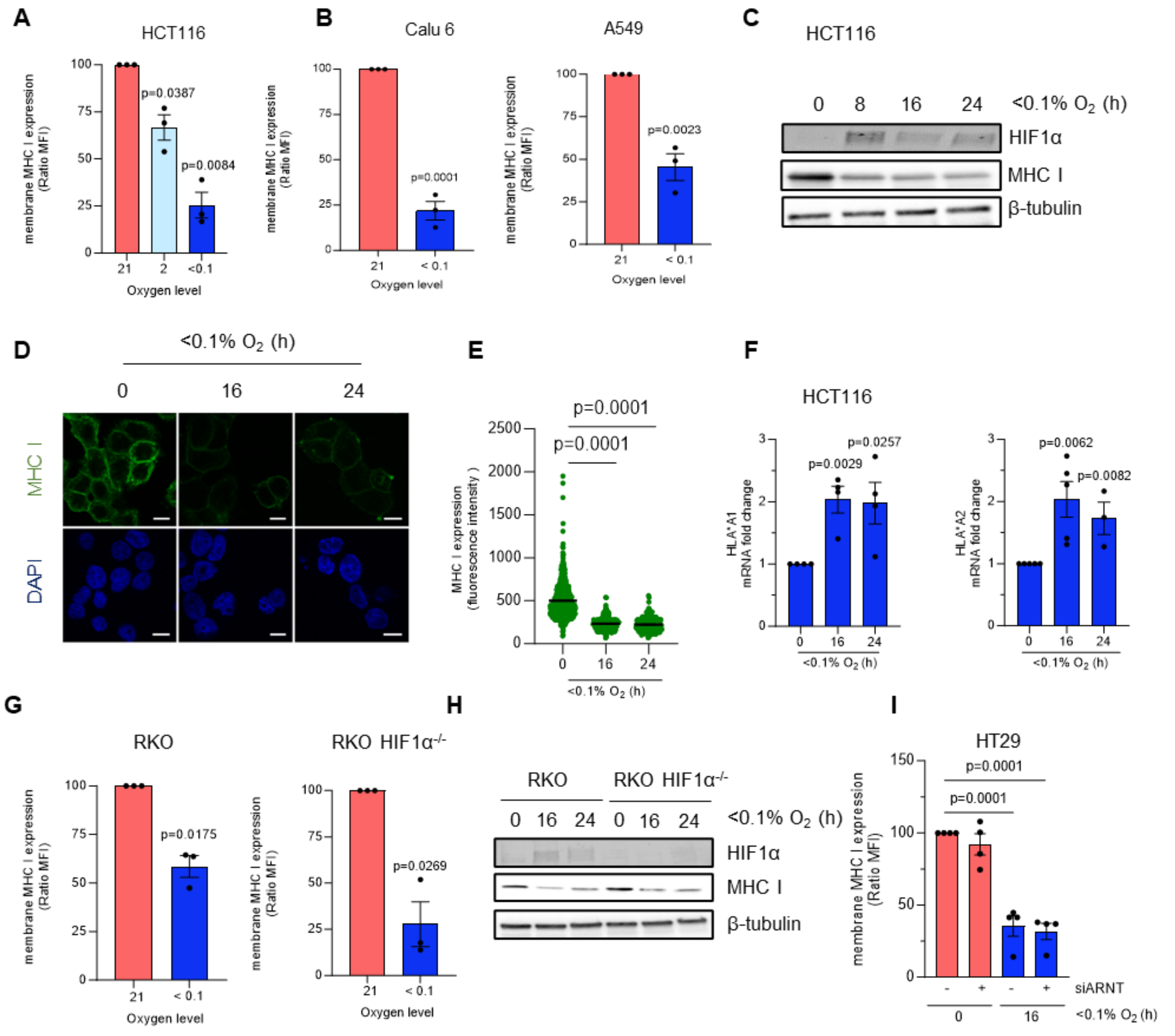

Appendix Fig.S2 Hypoxia leads to a decreased MHC I expression.

(**A**) HCT116 cells were exposed to hypoxia 21,2 and <0.1% O_2 for 24 hours. Flow cytometry-based analysis of MHC I expression on the cell surface was carried out. Graph showing mean fluorescence intensity of MHC I expression on the cell surface. Data represents mean ± SEM from three biological replicates. Statistical analysis was determined using paired student's t-test. (**B**) Calu 6 and A549 cells were exposed to hypoxia <0.1% O_2 for 24 hours. Flow cytometry-based analysis MHC I expression on the cell surface was carried out. Graph showing mean fluorescence intensity of MHC I expression on the cell surface. Data represents mean ± SEM from three biological replicates. Statistical analysis was determined using unpaired student's t-test. (**C**) HCT116 cells were exposed to hypoxia (<0.1% O_2) for the times indicated. MHC I protein level from whole cell lysates was detected by western blot. (n=3 biological replicates). (**D**) HT29 cells were exposed to hypoxia (<0.1% O_2) for the times indicated. Immunofluorescence staining for MHC I was then carried out. Representative images are shown (scale bar: 10 µm). MHC I staining in green, DAPI (blue) (n=3 biological replicates). (**E**) Graph showing the quantification of the fluorescence intensity of MHC I expression in HT29 cells. Data represents mean ± SEM from three biological replicates. Statistical analysis was determined using unpaired student's t-test. (**F**) HCT116 cells were exposed to <0.1% O_2 for the times indicated. qPCR for *HLA*A1* (n=4 biological replicates) and *HLA*A2* (n=3 biological replicates) genes was carried out. 18S served as a housekeeping gene. Data represents mean ± SEM. Statistical analysis was calculated using unpaired student's t-test. (**G**) RKO and RKO HIF1a$^{-/-}$ cells were exposed to hypoxia <0.1% O_2 for 24 hours. Flow cytometry-based analysis of MHC I expression on the cell surface was carried out. Graph showing mean fluorescence intensity of MHC I expression on the cell surface. Data represents mean ± SEM from three biological replicates. Statistical analysis was determined using paired student's t-test. (**H**) RKO and RKO HIF1a$^{-/-}$ were exposed to hypoxia <0.1% O_2 for the indicated times. MHC I protein level was detected by western blot (n=3 biological replicates). (**I**) HT29 cells were transfected with siARNT and exposed to hypoxia (<0.1% O_2) for 16 hours followed by flow cytometry. Graph showing mean fluorescence intensity of MHC I expression on the cell surface. Data represents mean ± SEM from four biological replicates. Statistical analysis was determined using unpaired student's t-test.

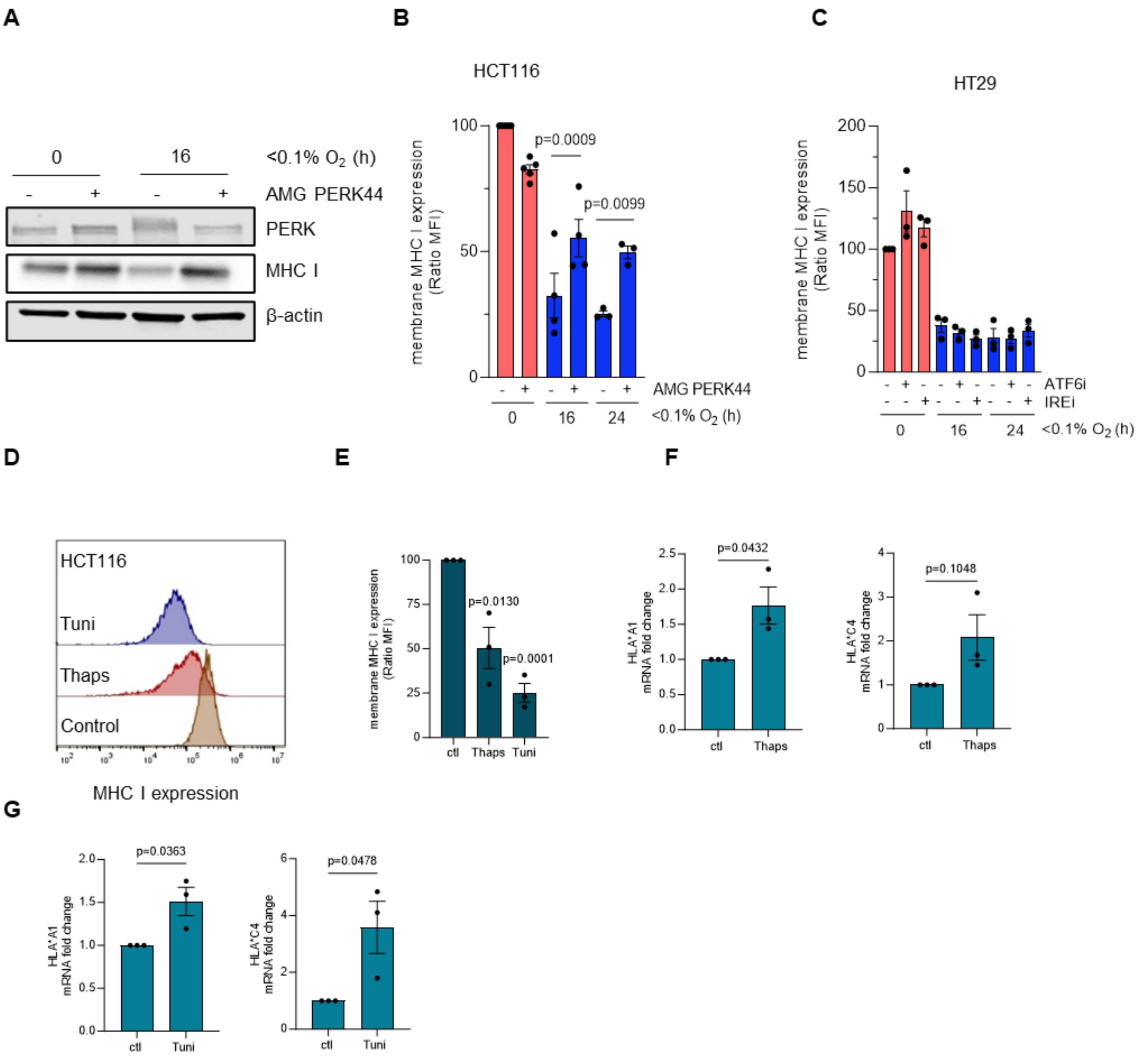

Appendix Fig.S3 PERK arm of the UPR regulates MHC I degradation under hypoxia.

(**A**) HCT116 cells were treated with PERK inhibitor (10 µM) and exposed to normoxia or hypoxia (<0.1% O_2) for 16 hours followed by western blotting as indicated (n=3 biological replicates). PERK inhibition was confirmed by the absence in the electrophoretic mobility shift of PERK on the western blot. (**B**) HCT116 cells were treated with PERK inhibitor (AMG PERK 44, 10 µM) and exposed to normoxia or hypoxia (<0.1% O_2) for 16 and 24 hours followed by flow cytometry. Graph showing mean fluorescence intensity of MHC I expression on the cell surface. Data represents mean ± SEM from three biological replicates. Statistical significance was determined with paired student's t-test. (**C**) HT29 cells were treated with ATF6 inhibitor (Ceapin-A7, 10 µM) or IREα inhibitor (4µ8c, 20 µM) and exposed to normoxia or hypoxia (<0.1% O_2) for 16 and 24 hours followed by flow cytometry. Graph showing mean fluorescence intensity of MHC I expression on the cell surface. Data represents mean ± SEM from three biological replicates. (**D**) HCT116 cells were treated with Thapsigargin (Thaps, 2 µM) and Tunicamycin (Tuni, 5 µg/ml) for 24 hours. Representative histograms showing MHC I expression determined using flow cytometry (n=3 biological replicates). (**E**) Graph showing mean fluorescence intensity of MHC I expression on the cell surface of HCT116 cells. Data represents mean **±** SEM from three biological replicates. Statistical significance was determined with unpaired student's t-test. (**F**) HT29 cells were treated with Thapsigargin (Thaps, 2 µM) for 24 hours. qPCR for *HLA*A1* and *HLA*C4* genes was carried out. 18S served as a housekeeping gene. Data represents mean ± SEM from three biological replicates. Statistical analysis was calculated using unpaired student's t-test. (**G**) HT29 were treated with Tunicamycin (tuni, 5 µg/ml) for 24 hours. qPCR for *HLA*A1* and *HLA*C4* genes was carried out. 18S served as a housekeeping gene. Data represents mean ± SEM from three biological replicates. Statistical analysis was calculated using unpaired student's t-test.

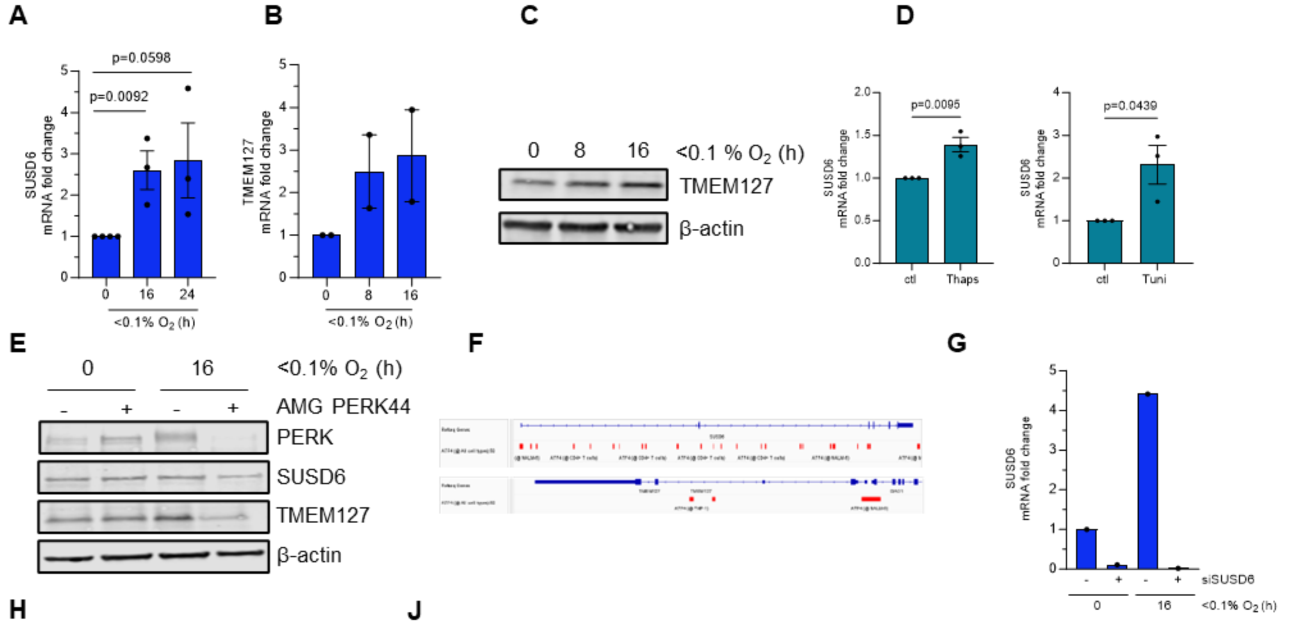

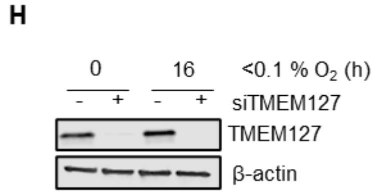

J

Correlation of mRNA expression of STUB1, WWP2 and hypoxia signature

TCGA datasets:	Spearman:	P value
STUB1 Colorectal (n=595)	0.33	<0.0001
WWP2 Colorectal (n=595)	-0.22	<0.0001

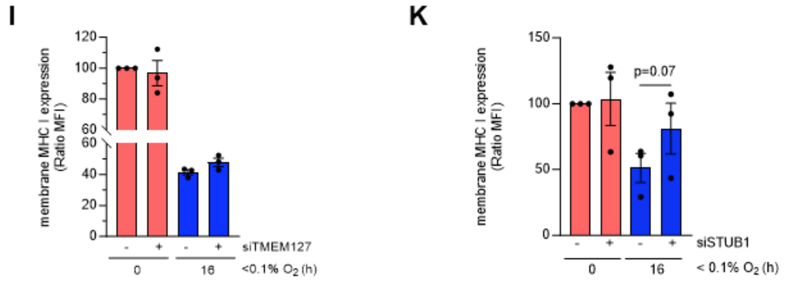

Appendix Fig.S4 SUSD6, TMEM127 and STUB1 are hypoxia-induced genes.

(**A**) HT29 cells were exposed to <0.1% O_2 for the times indicated. qPCR for SUSD6 was carried out. 18S served as a housekeeping gene. Data represents mean ± SEM from three biological replicates. Statistical significance was determined with unpaired student's t-test (**B**) HT29 cells were exposed to <0.1% O_2 for the times indicated. qPCR for TMEM127 was carried out. 18S served as a housekeeping gene (n=2 biological replicates). (**C**) HT29 cells were exposed to hypoxia (<0.1% O_2) for the times indicated. TMEM127 protein level from whole cell lysates was detected by western blot. A representative western blot of two biological replicates is shown. (**D**) HT29 cells were treated with Thapsigargin (Thaps, 2 µM) and Tunicamycin (tuni, 5 µg/ml) or for 24 hours. qPCR for SUSD6 gene was carried out. 18S served as a housekeeping gene. Data represents mean ± SEM from three biological replicates. Statistical significance was determined using unpaired student's t-test. (**E**) HT29 cells were treated with PERK inhibitor (10 µM) and exposed to normoxia or hypoxia (<0.1% O_2) for 16 hours followed by western blotting as indicated (n=2 biological replicates). (**F**) Integrative genomics viewer (IGV) showing the binding sites for ATF4 in SUSD6 gene. (**G**) HT29 cells were transfected with siSUSD6 and exposed to hypoxia (<0.1% O_2) for16 hours. qPCR for SUSD6 gene was carried out. 18S served as a housekeeping gene in the qPCR analysis (n=1 biological replicate). (**H**) HT29 cells were transfected with siTMEM127 and exposed to hypoxia (<0.1% O_2) for 16 hours and subjected to western blotting with antibodies indicated. A representative western blot of two biological replicates is shown. (**I**) HT29 cells were transfected with siTMEM127 and exposed to hypoxia (<0.1% O_2) for 16 hours. Mean fluorescence intensity of MHC I expression on the cell surface determined using flow cytometry is shown. Data represents mean ± SEM from three biological replicates. Statistical analysis was calculated using unpaired student's t-test. (**J**) Expression of STUB1 and WWP2 (mRNA) were correlated with the hypoxia metagene signature in the indicated TCGA colorectal cancer patient cohort. The number of patient samples is shown in brackets. Spearman's rank correlation coefficient and P values are shown for the Log10 median expression of STUB1, WWP2 and hypoxic signature. (**K**) HT29 cells were transfected with siSTUB1 and exposed to hypoxia (<0.1% O_2) for 16 hours. Mean fluorescence intensity of MHC I expression on the cell surface determined using flow cytometry is shown. Data represents mean ± SEM from three biological replicates. Statistical analysis was calculated using paired student's t-test.

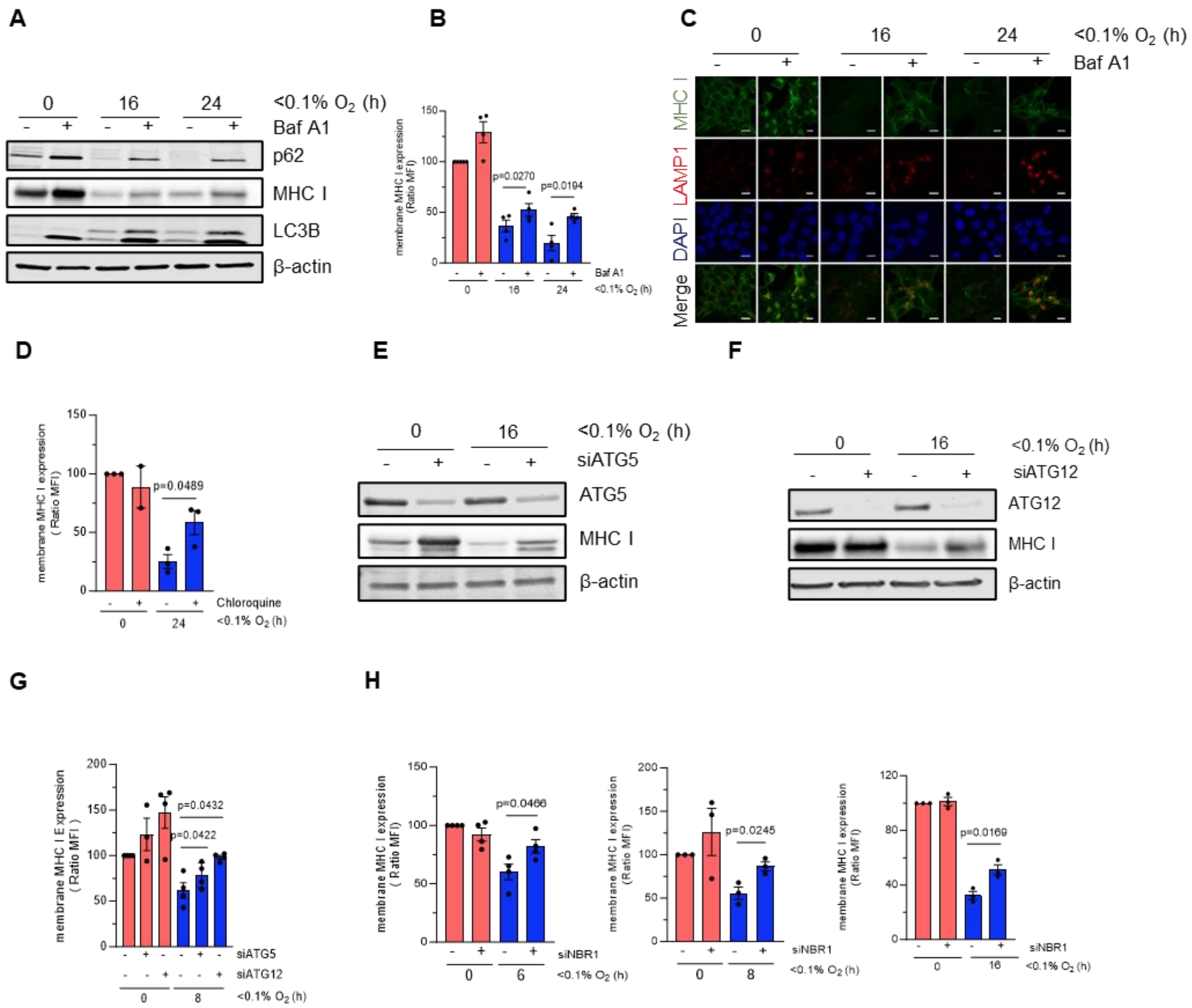

Appendix Fig.S5 Degradation of MHC I under hypoxia is linked to the activation of autophagy.

(**A**) HCT116 cells were exposed to hypoxia (<0.1% O_2) and treated with bafilomycin A1 (50 nM) for the times indicated. Western blotting was then carried out with antibodies indicated (n=3 biological replicates). (**B**) HCT116 cells were treated with bafilomycin A1 (50 nM) and exposed to hypoxia (<0.1% O_2) for 16 and 24 hours. MHC I expression on the cell surface was determined using flow cytometry. Graph showing mean fluorescence intensity of MHC I expression on the cell surface. Data represents mean ± SEM from four biological replicates. Statistical analysis was calculated using paired student's t-test. (**C**) HCT116 cells were treated with bafilomycin A1 (50 nM) and exposed to hypoxia (<0.1% O_2) for 16 and 24 hours. Staining for MHC I and LAMP1 was then carried out. representative images showing the localization of MHC I (green) relative to LAMP1 positive (red) lysosomes (scale bar: 10 µm) (n=3 biological replicates). (**D**) HT29 cells were treated with Chloroquine (50 µM) and exposed to hypoxia (<0.1% O_2) for 16 and 24 hours. MHC I expression on the cell surface was determined using flow cytometry. Graph showing mean fluorescence intensity of MHC I expression on the cell surface. Data represents mean ± SEM from three biological replicates. Statistical analysis was calculated using unpaired student's t-test. (**E**) HT29 cells were transfected with siATG5 and exposed to hypoxia (<0.1% O_2) for 16 hours and subjected to western blotting with antibodies indicated. A representative western blot of three biological replicates is shown. (**F**) HT29 cells were transfected with siATG12 and exposed to hypoxia (<0.1% O_2) for 16 hours and subjected to western blotting with antibodies indicated. A representative western blot of three biological replicates is shown. (**G**) HT29 cells were transfected with siATG5 or siATG12 and exposed to hypoxia (<0.1% O_2) for 8 hours. Graph showing mean fluorescence intensity of MHC I expression on the cell surface determined using flow cytometry. Data represents mean ± SEM from four biological replicates. Statistical analysis was calculated using paired student's t-test. (**H**) HT29 cells were transfected with siNBR1 and exposed to hypoxia (<0.1% O_2) for 6 (n=4),8 (n=3) and 16 (n=3) hours. Graph showing mean fluorescence intensity of MHC I expression on the cell surface determined using flow cytometry. Data represents mean ± SEM. Statistical analysis was calculated using unpaired student's t-test.

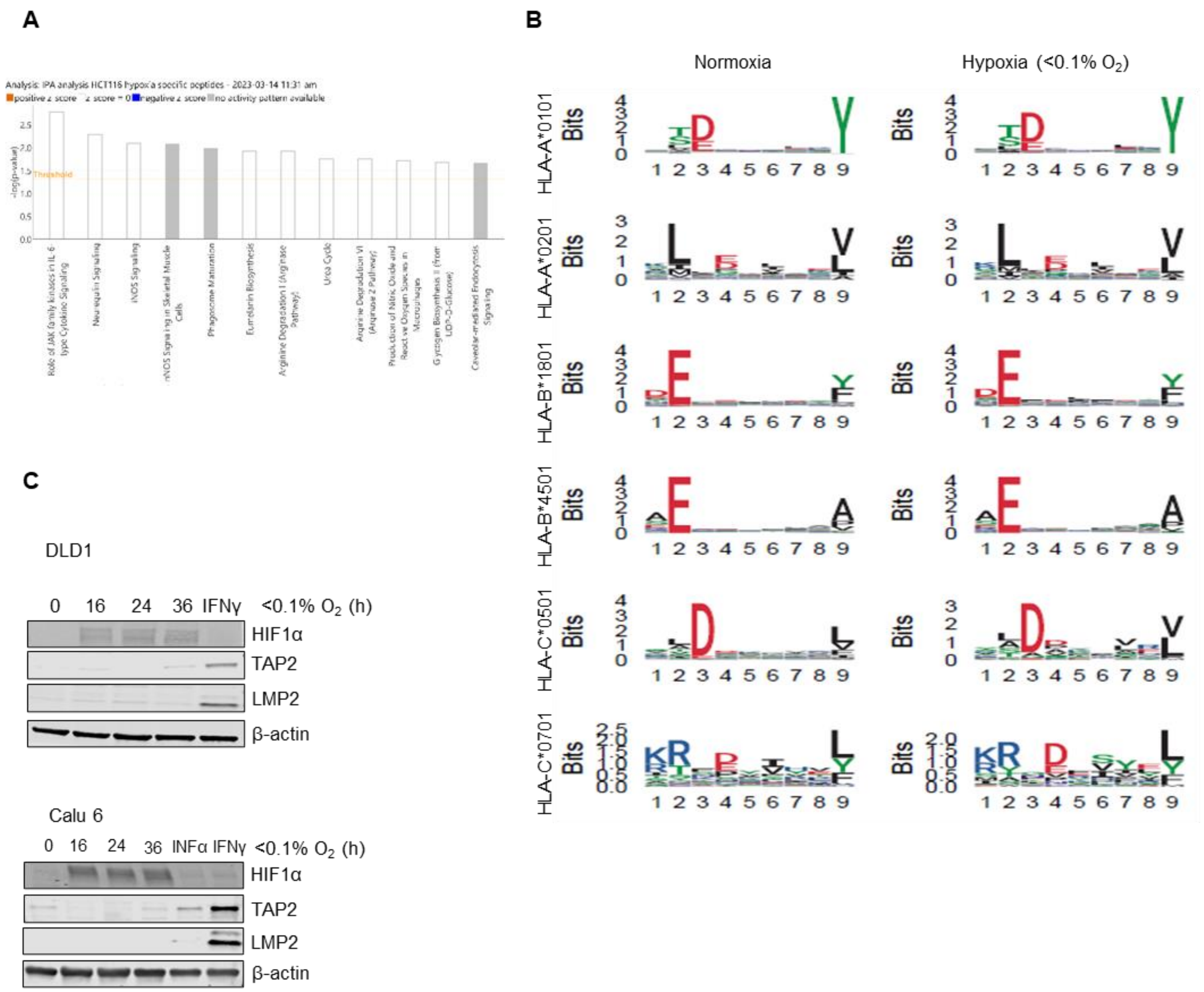

Appendix Fig.S6 Reduced MHC I expression under hypoxia is associated with decreased antigen presentation.

(**A**) Ingenuity Pathway Analysis of overexpressed source proteins derived from filtered hypoxia-specific peptides. (**B**) Seqlogo comparison of MHC binding motifs in normoxia (21% O_2) and hypoxia (<0.1% O_2) 24 hours after exposure to hypoxia. Peptides were assessed using NetMHCPan 4.1. (**C**) DLD1 and Calu6 cells were exposed to <0.1% O_2 for the times indicated and were subjected to western blotting with the antibodies indicated. INFα (1000 U/ml) and INFγ (100 ng/ml) were used as positive controls (n=3 biological replicates).

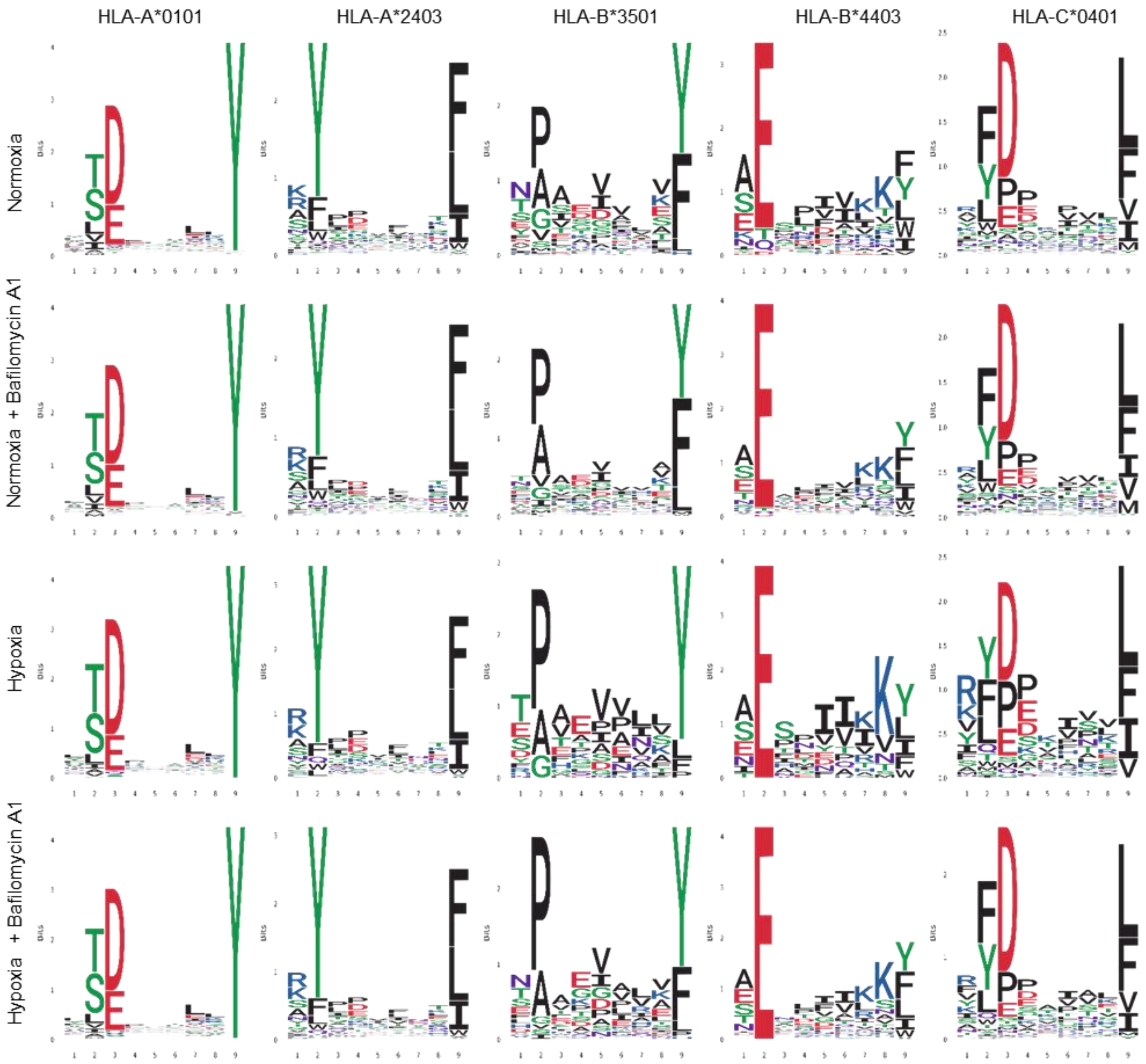

Appendix Fig.S7 MHC I binding motifs of the identified peptides between the different conditions.

Seqlogo comparison of MHC I binding motifs in normoxia (21% O_2), normoxia (21% O_2) + bafilomycin A1, hypoxia (<0.1% O_2) and hypoxia (<0.1% O_2) + bafilomycin A1. Peptides were assessed using NetMHCPan 4.1.

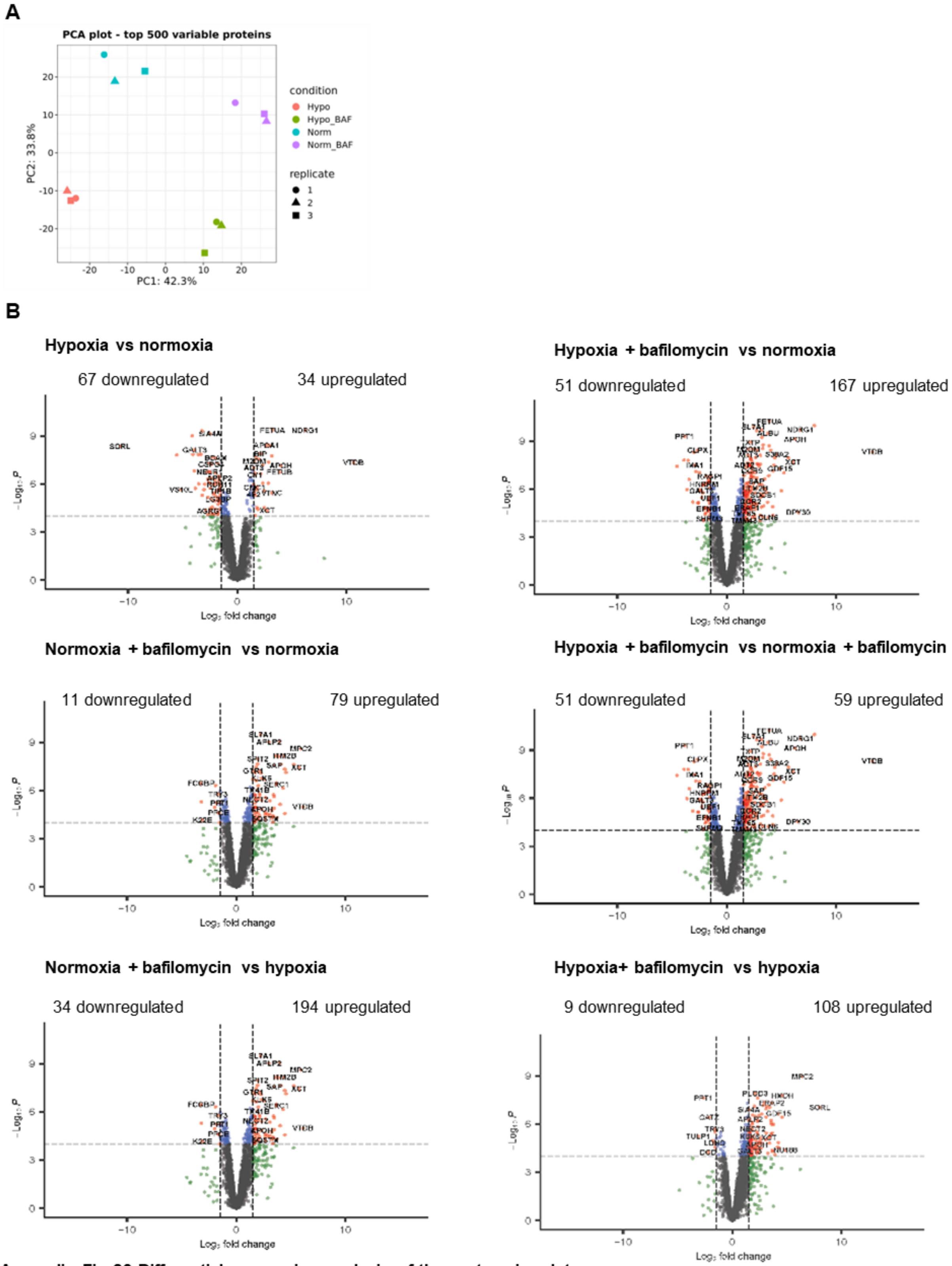

Appendix Fig.S8 Differential expression analysis of the proteomics data.

(**A**) PCA plot of treatment conditions and biological replicates for HT29 cells for proteomics data in HT29 cells. Data is representative of three biological replicates for each condition. (**B**) Volcano plot of differentially expressed proteins in HT29 cells exposed to normoxia or hypoxia (<0.1% O_2) and treated with bafilomycin A1 (50 nM) for 24 hours (limma, -Log10p /> 5, -Log2 fold change >1.5).

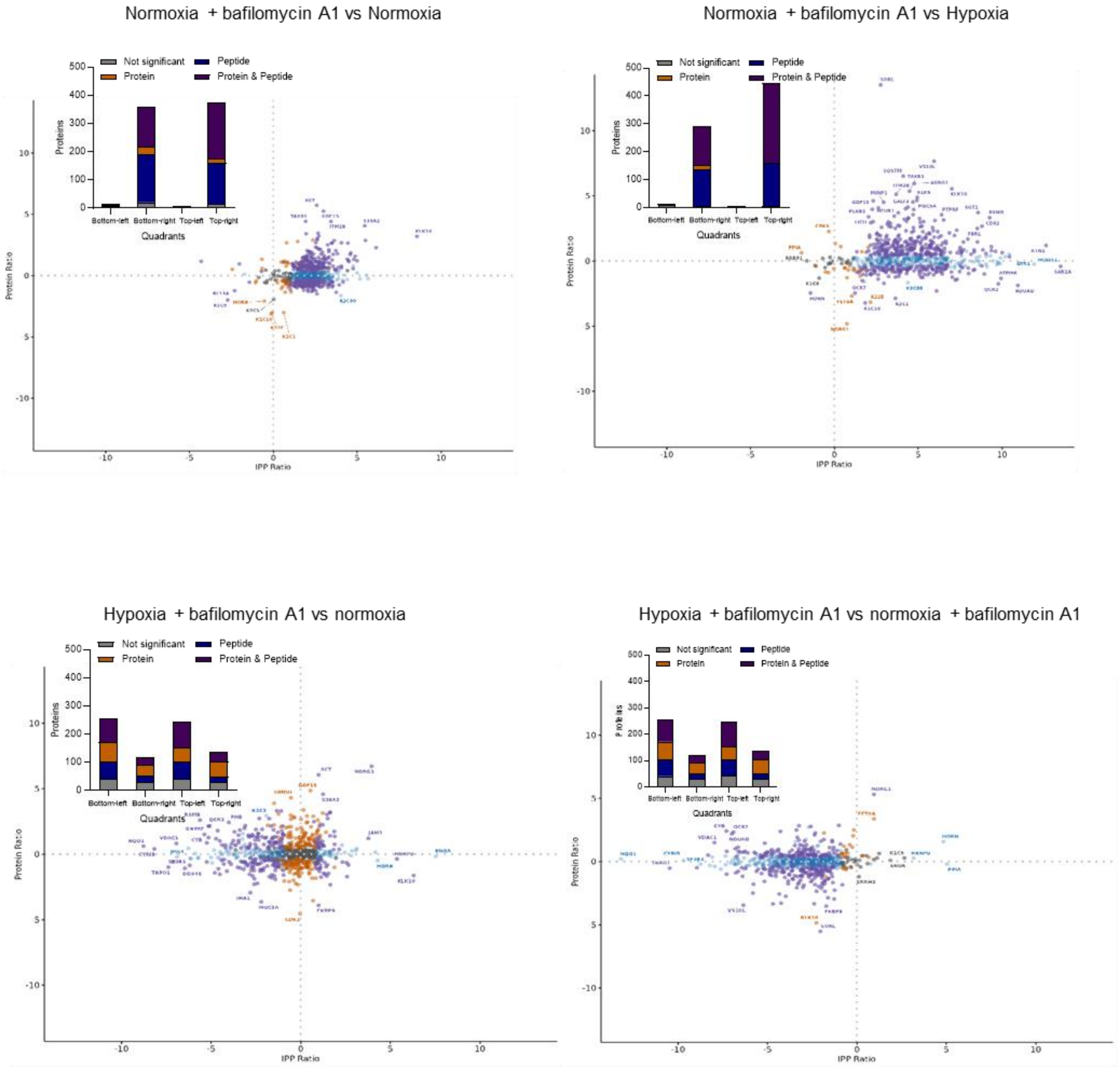

Appendix Fig.S9 Changes in the proteome do not correlate with the changes in the immunpeptidome.

Correlation plot of the proteome protein and the immunopeptidome peptide ratio between normoxia (21% O_2), normoxia + bafilomycin A1, hypoxia (<0.1% O_2) and hypoxia + bafilomycin A1. Significantly differentially expressed proteins and peptides are indicated by their colors. A bar chart to quantify proteins in each quadrant is presented next to each plot.

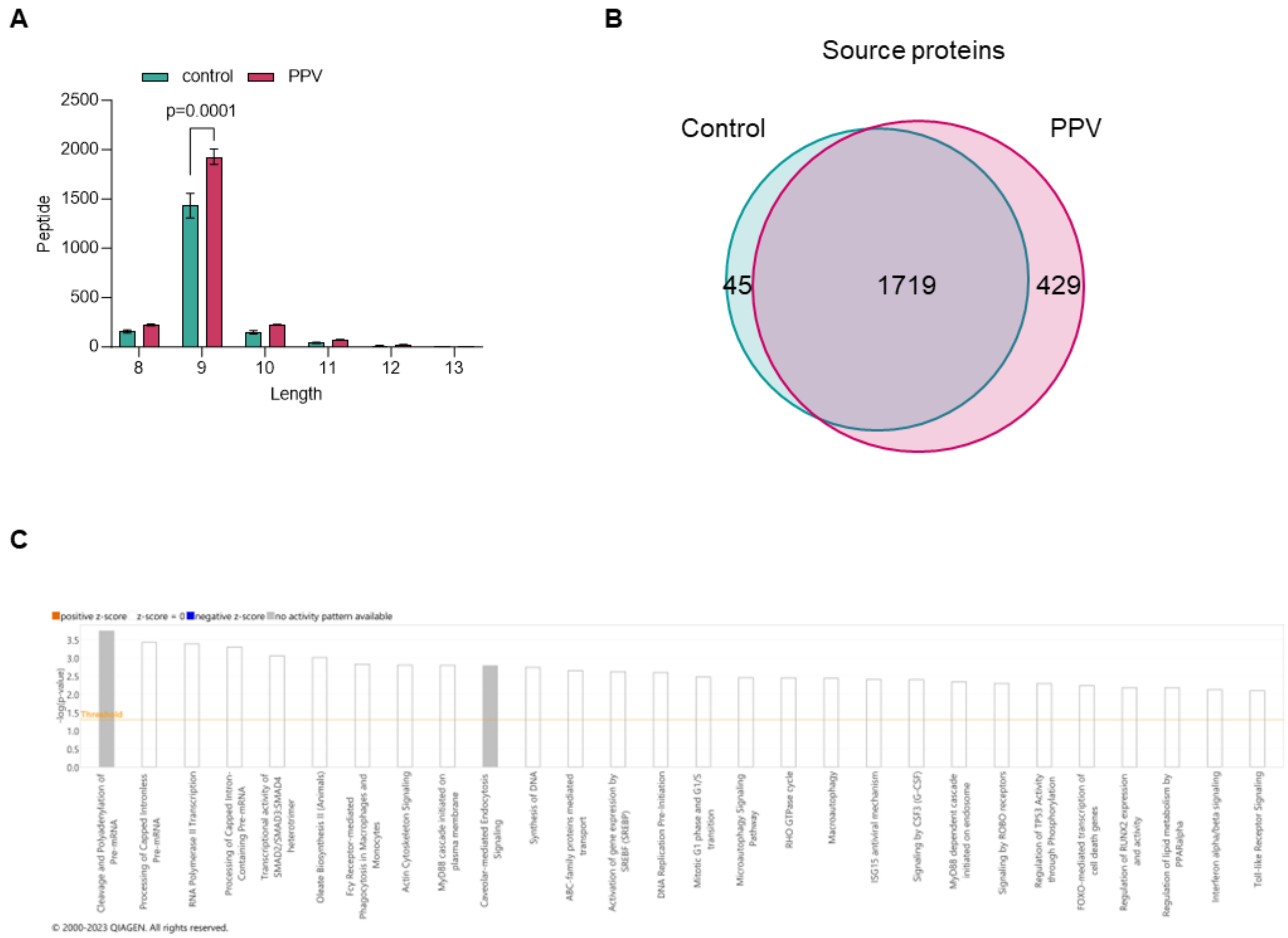

Appendix Fig.S10 Regulation of hypoxia *in vivo* induces a change in the presented peptides and their source proteins.

(**A**) Graph showing length frequency distribution of MHC I peptides in the indicated conditions. Data represent mean ± SEM from three different mice. Statistical analysis was determined using 2 Way-Anova test. (**B**) Venn diagram showing the number of unique source proteins in control and papaverine conditions. (**C**) Ingenuity Pathway Analysis of overexpressed source proteins derived from filtered papaverine unique peptides.
