# Supplementary figures and images for: Hypoxia promotes tumor immune evasion by suppressing MHC-I expression and antigen presentation (part 1 of 2)
Source: EMBO J. 2025 Jan 3;44(3):903–22. doi: 10.1038/s44318-024-00319-7 (PMC11790895; doi:10.1038/s44318-024-00319-7)

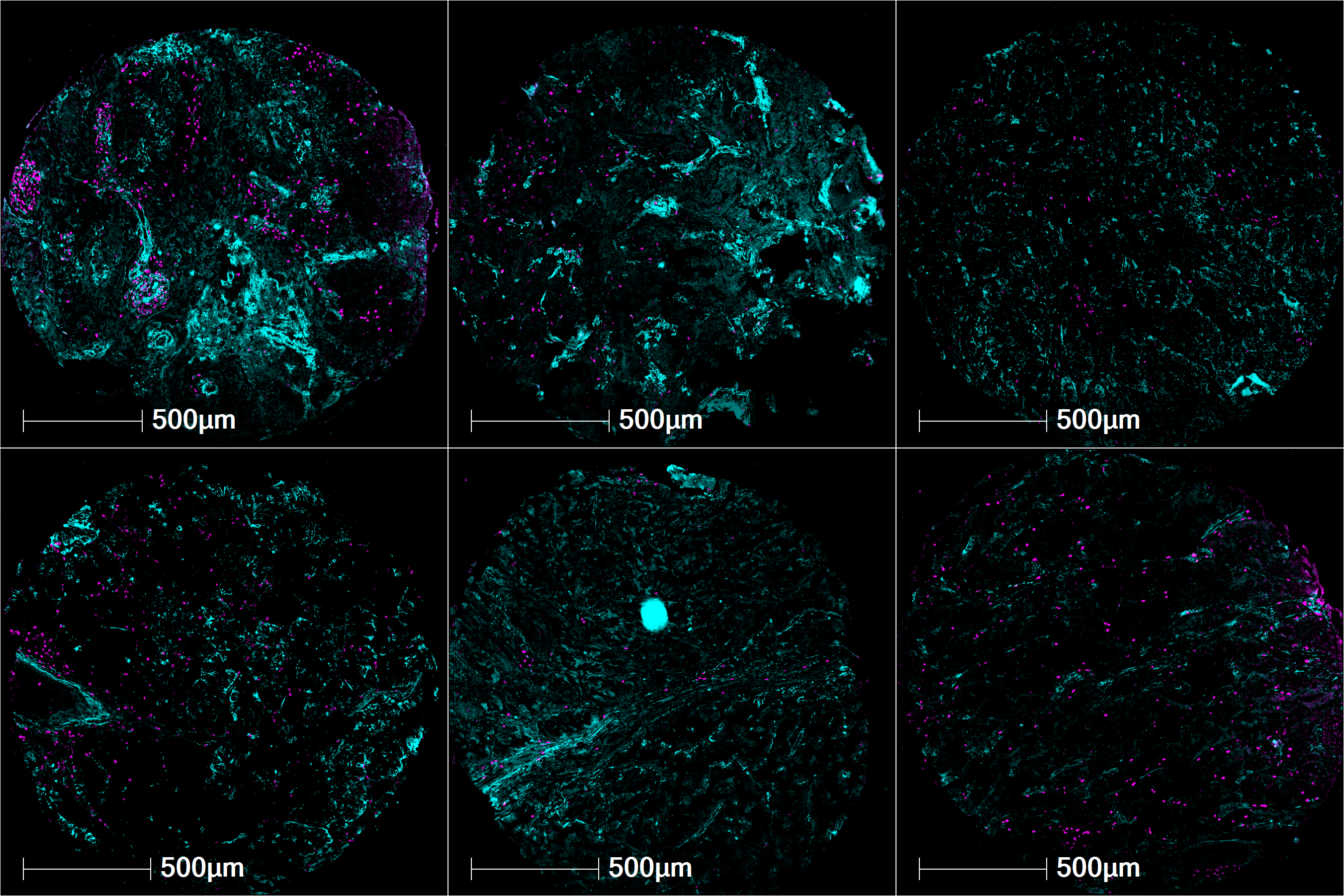

Supplement: Supplementary file 3 — Source data Fig. 1 [file 44318_2024_319_MOESM3_ESM.zip › EMBOJ-2024-117498-T-SourceDataForFigure1A-G/Figure 1 A/CAIX CD8 staining representative image .tiff]

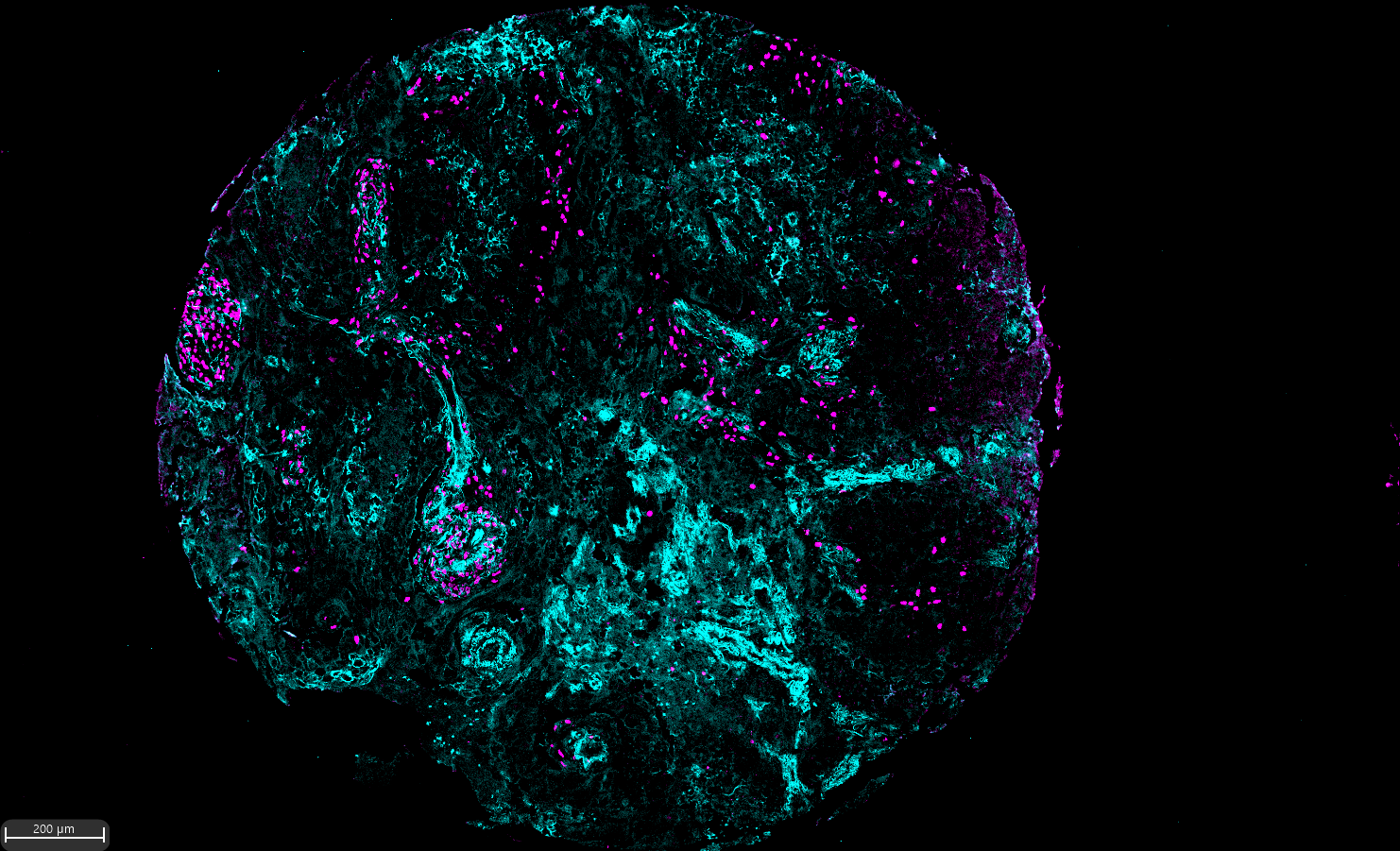

Supplement: Supplementary file 3 — Source data Fig. 1 [file 44318_2024_319_MOESM3_ESM.zip › EMBOJ-2024-117498-T-SourceDataForFigure1A-G/Figure 1 A/Core 1 CD8 CAIX.tiff]

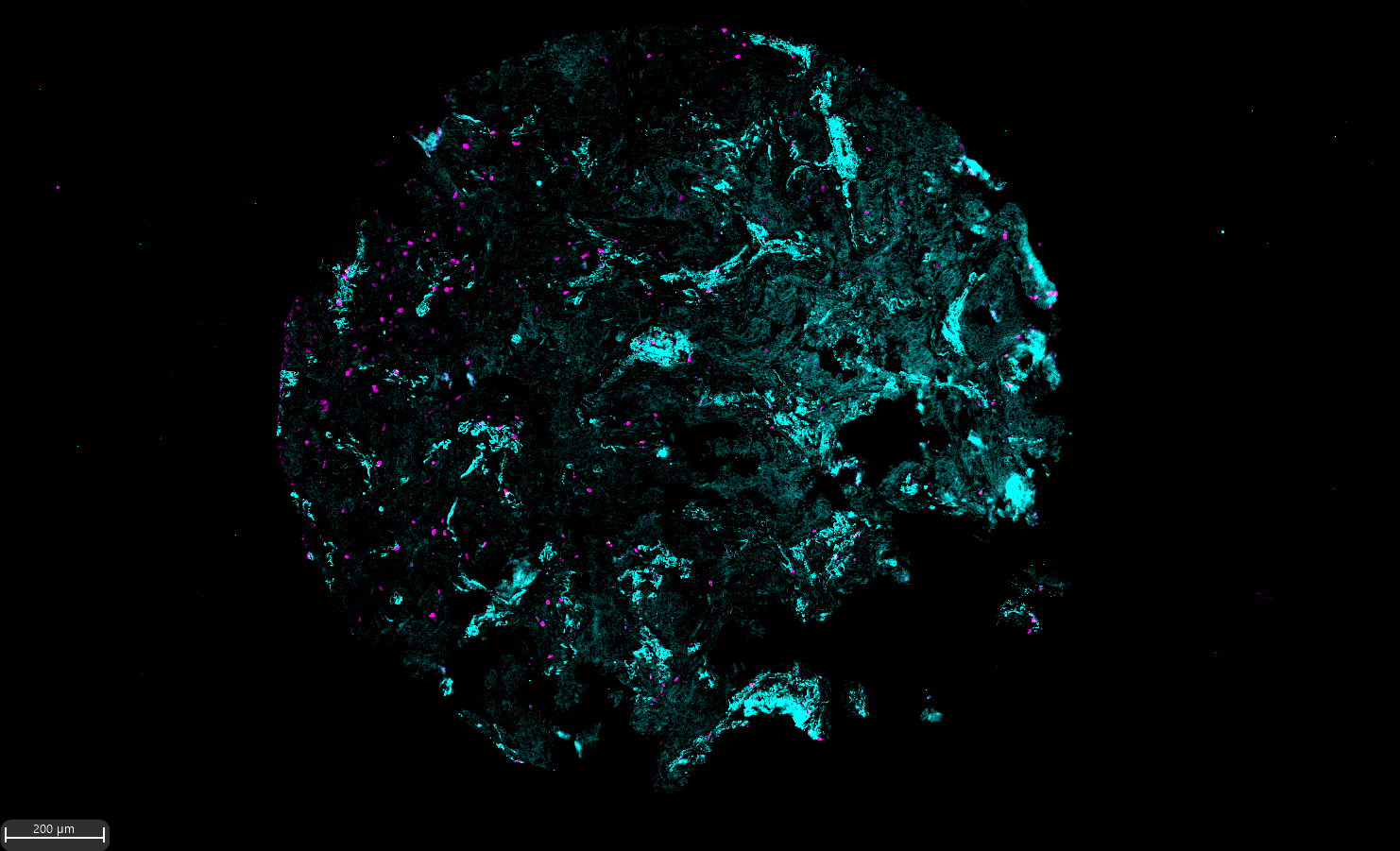

Supplement: Supplementary file 3 — Source data Fig. 1 [file 44318_2024_319_MOESM3_ESM.zip › EMBOJ-2024-117498-T-SourceDataForFigure1A-G/Figure 1 A/Core 2 CD8 CAIX.tiff]

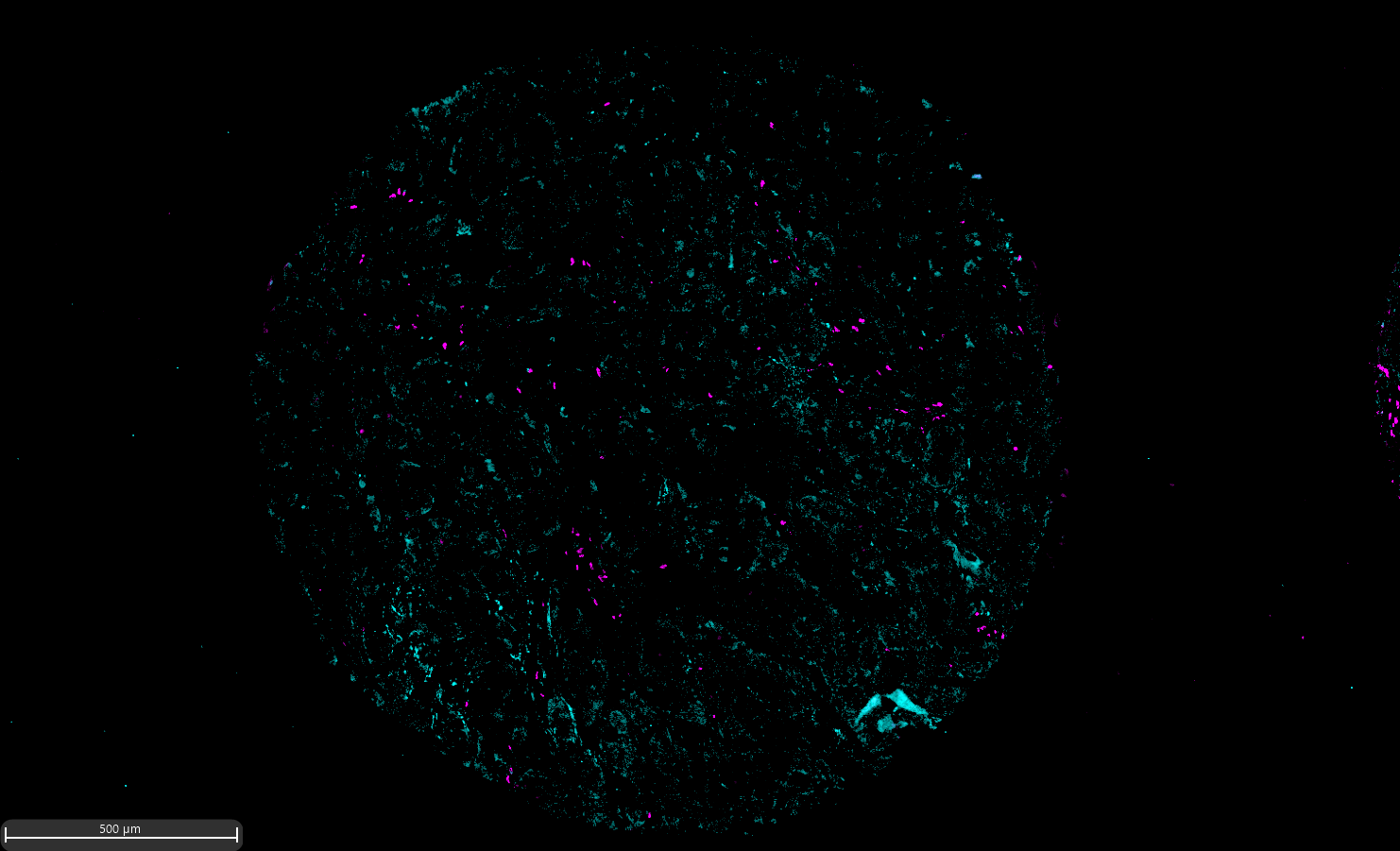

Supplement: Supplementary file 3 — Source data Fig. 1 [file 44318_2024_319_MOESM3_ESM.zip › EMBOJ-2024-117498-T-SourceDataForFigure1A-G/Figure 1 A/Core 3 CD8 CAIX.tiff]

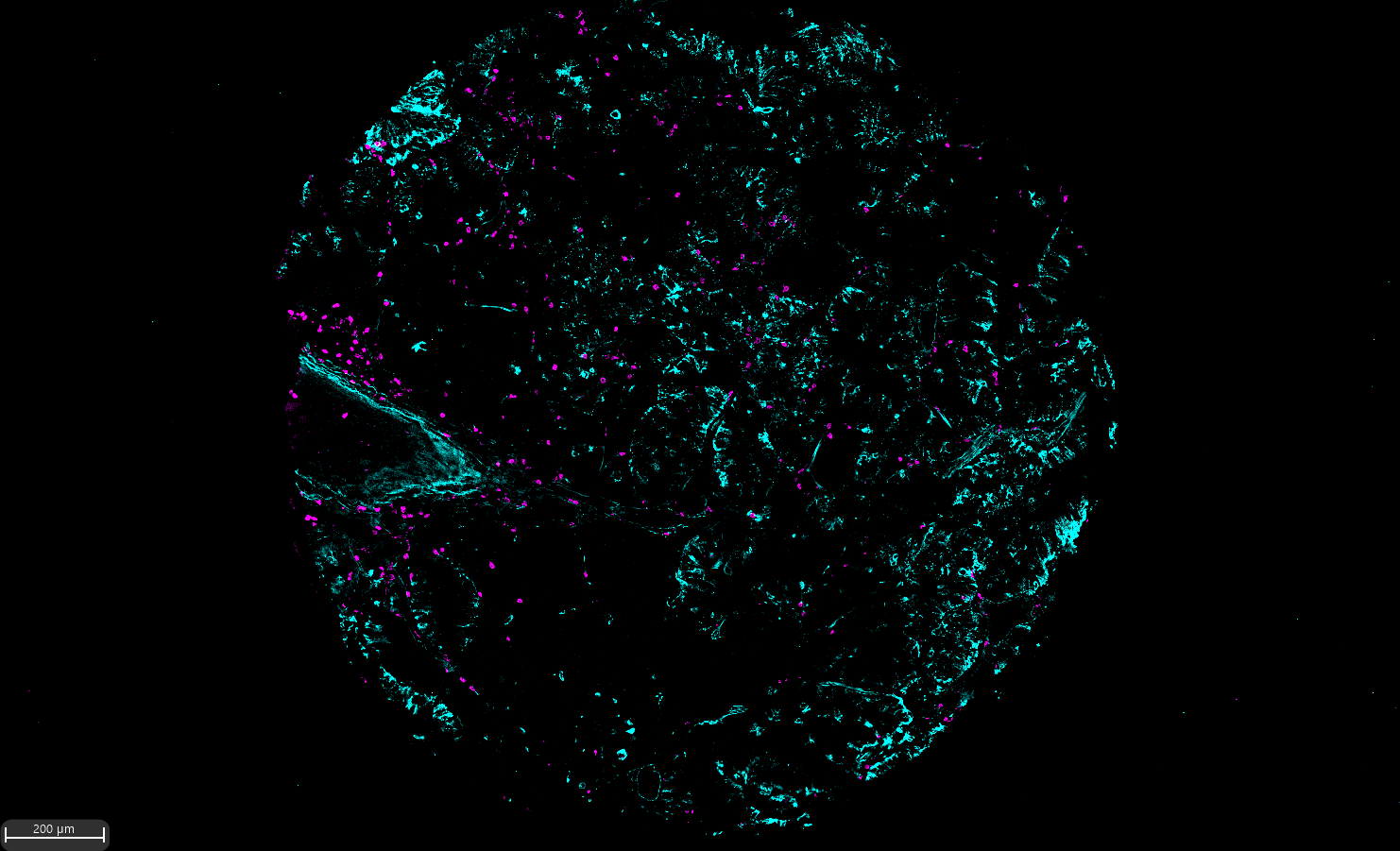

Supplement: Supplementary file 3 — Source data Fig. 1 [file 44318_2024_319_MOESM3_ESM.zip › EMBOJ-2024-117498-T-SourceDataForFigure1A-G/Figure 1 A/Core 4 CD8 CAIX.tiff]

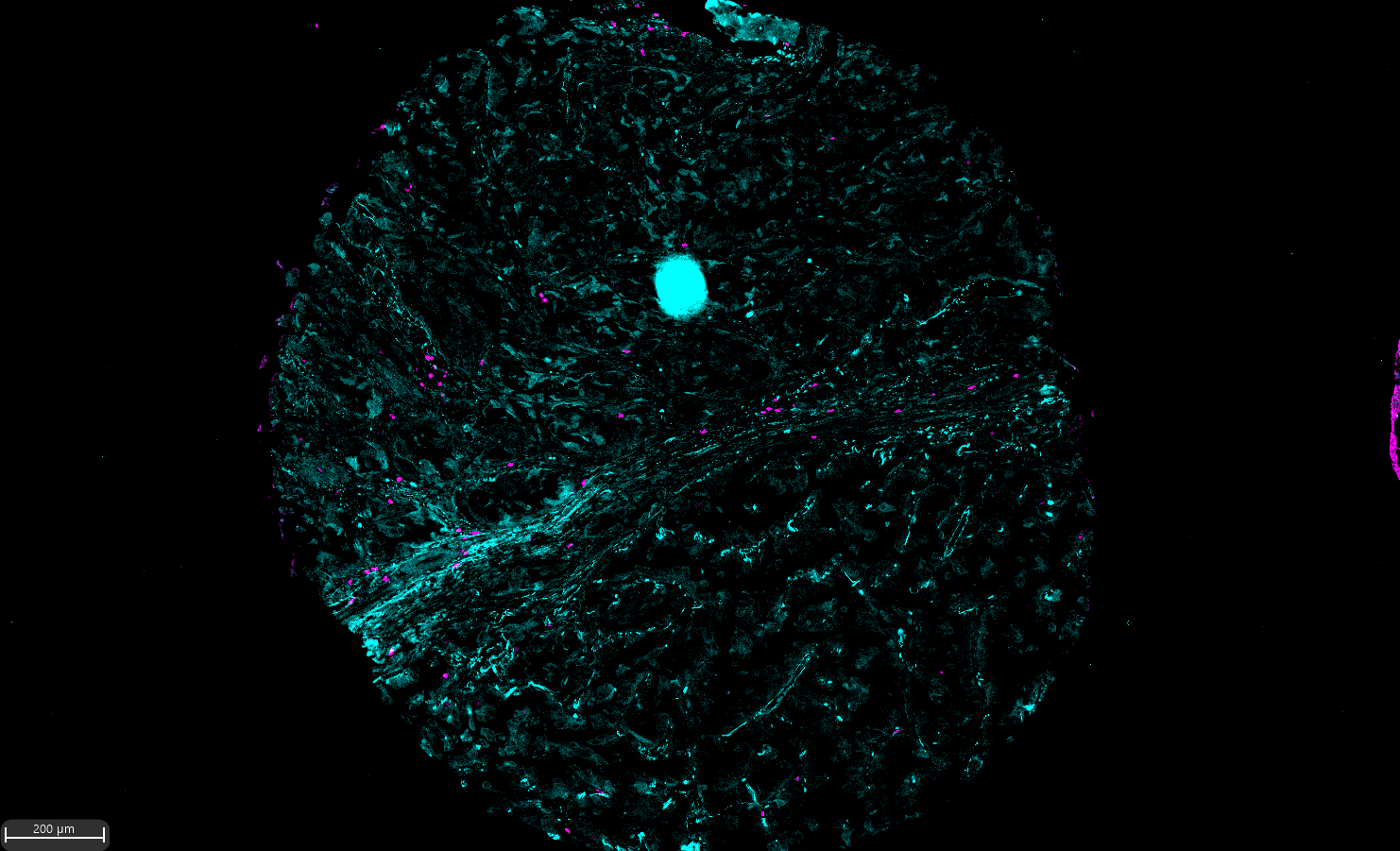

Supplement: Supplementary file 3 — Source data Fig. 1 [file 44318_2024_319_MOESM3_ESM.zip › EMBOJ-2024-117498-T-SourceDataForFigure1A-G/Figure 1 A/Core 5 CD8 CAIX.tiff]

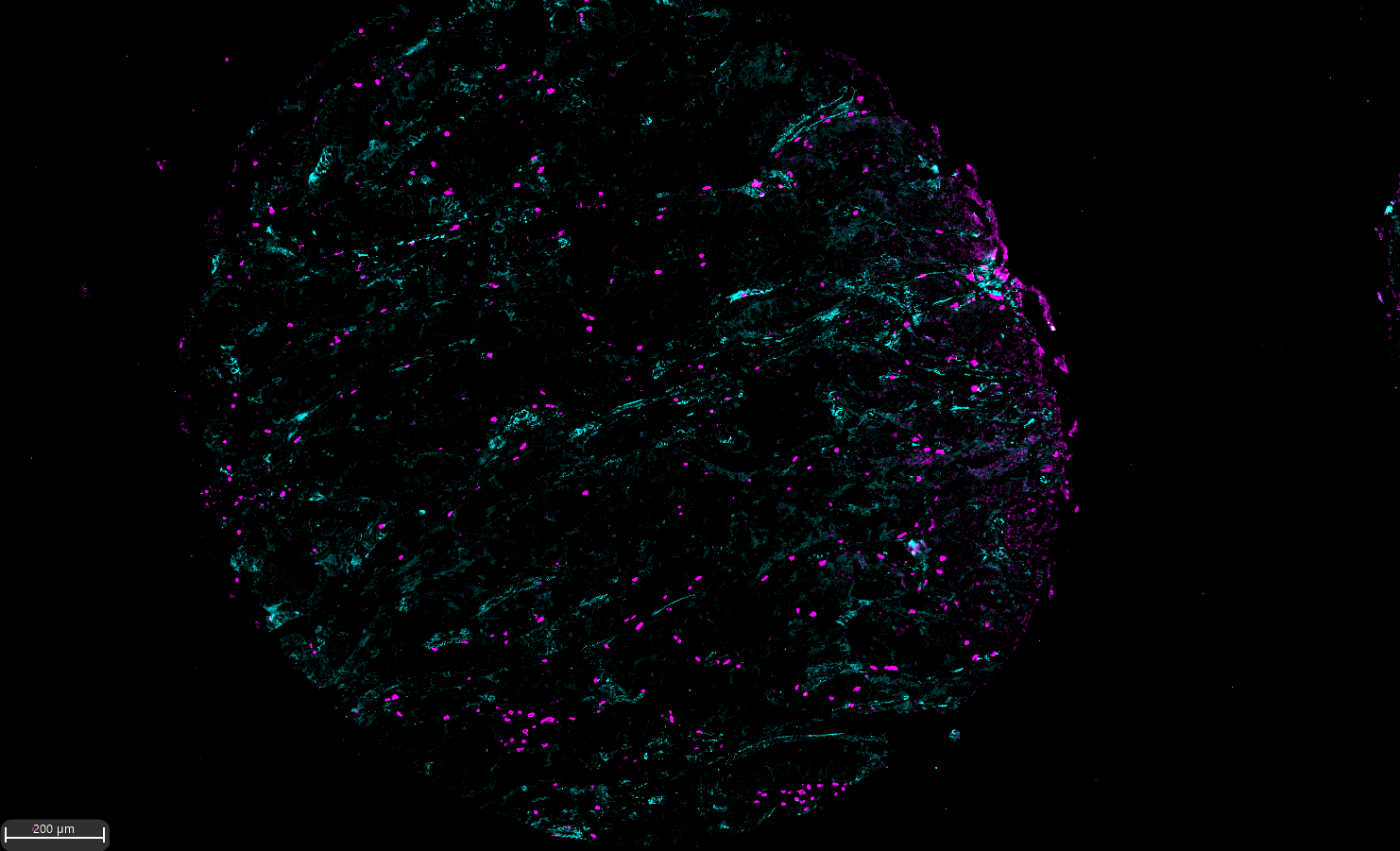

Supplement: Supplementary file 3 — Source data Fig. 1 [file 44318_2024_319_MOESM3_ESM.zip › EMBOJ-2024-117498-T-SourceDataForFigure1A-G/Figure 1 A/Core 6 CD8 CAIX.tiff]

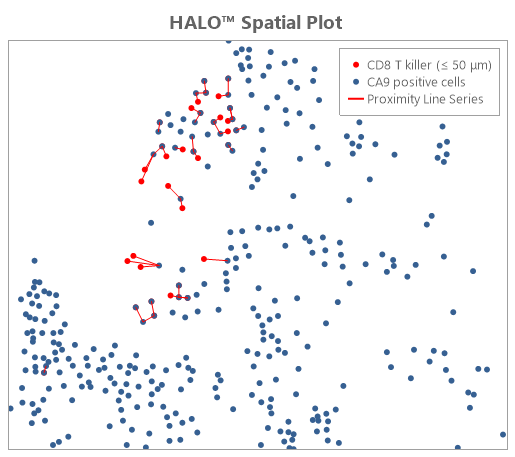

Supplement: Supplementary file 3 — Source data Fig. 1 [file 44318_2024_319_MOESM3_ESM.zip › EMBOJ-2024-117498-T-SourceDataForFigure1A-G/Figure 1 C/Proximity analysis Core 1.png]

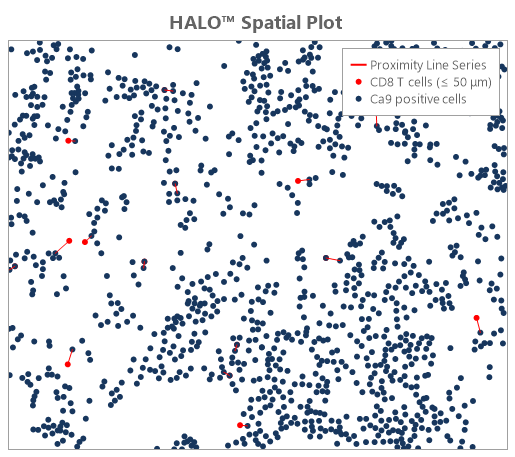

Supplement: Supplementary file 3 — Source data Fig. 1 [file 44318_2024_319_MOESM3_ESM.zip › EMBOJ-2024-117498-T-SourceDataForFigure1A-G/Figure 1 C/Proximity analysis Core 2.png]

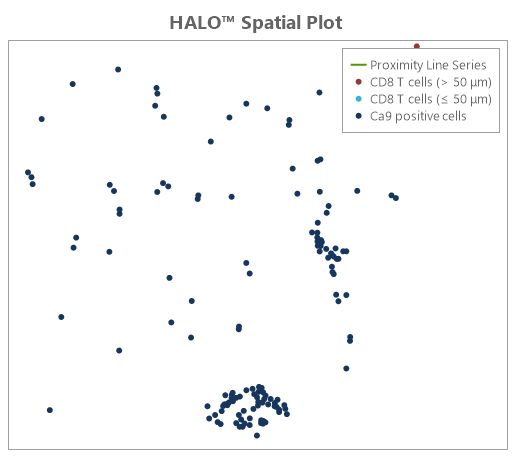

Supplement: Supplementary file 3 — Source data Fig. 1 [file 44318_2024_319_MOESM3_ESM.zip › EMBOJ-2024-117498-T-SourceDataForFigure1A-G/Figure 1 C/Proximity analysis Core 3.png]

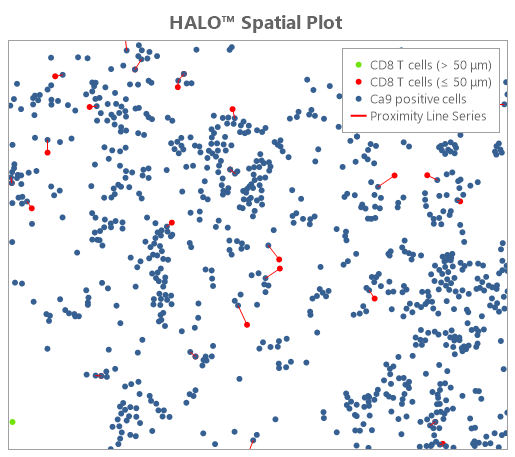

Supplement: Supplementary file 3 — Source data Fig. 1 [file 44318_2024_319_MOESM3_ESM.zip › EMBOJ-2024-117498-T-SourceDataForFigure1A-G/Figure 1 C/Proximity analysis Core 4.png]

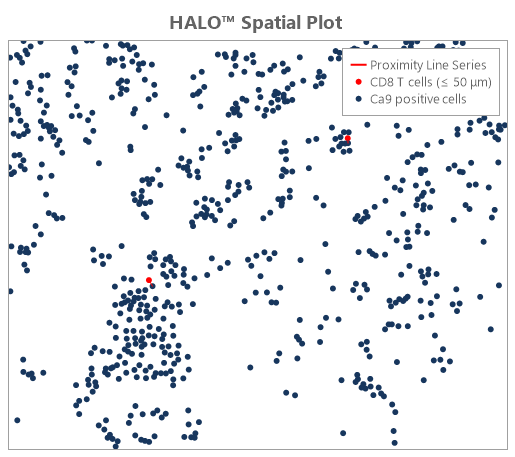

Supplement: Supplementary file 3 — Source data Fig. 1 [file 44318_2024_319_MOESM3_ESM.zip › EMBOJ-2024-117498-T-SourceDataForFigure1A-G/Figure 1 C/Proximity analysis Core 5.png]

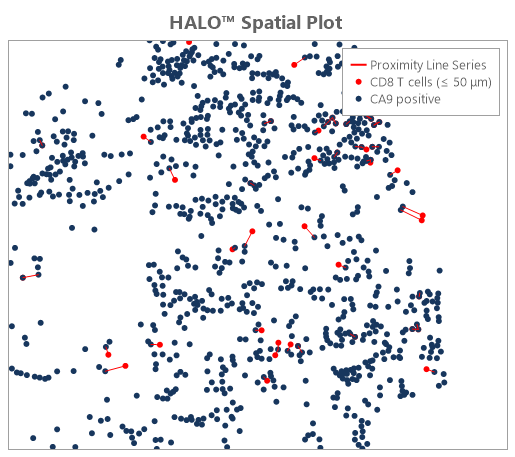

Supplement: Supplementary file 3 — Source data Fig. 1 [file 44318_2024_319_MOESM3_ESM.zip › EMBOJ-2024-117498-T-SourceDataForFigure1A-G/Figure 1 C/Proximity analysis Core 6.png]

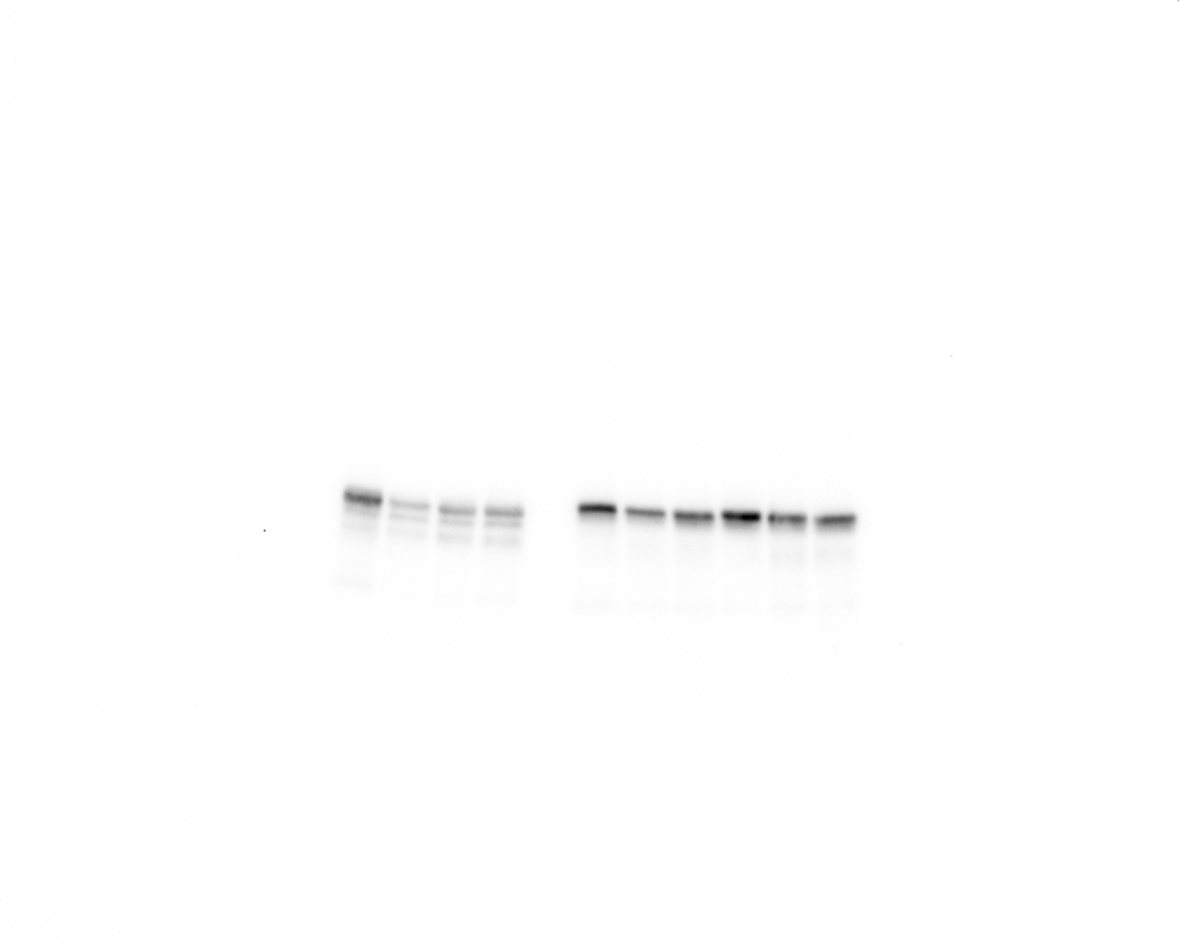

Supplement: Supplementary file 4 — Source data Fig. 2 [file 44318_2024_319_MOESM4_ESM.zip › EMBOJ-2024-117498-T-SourceDataForFigure2B-J/Figure 2 D/HT29_ Biological replicate n1/HT29_ western MHC I .tif]

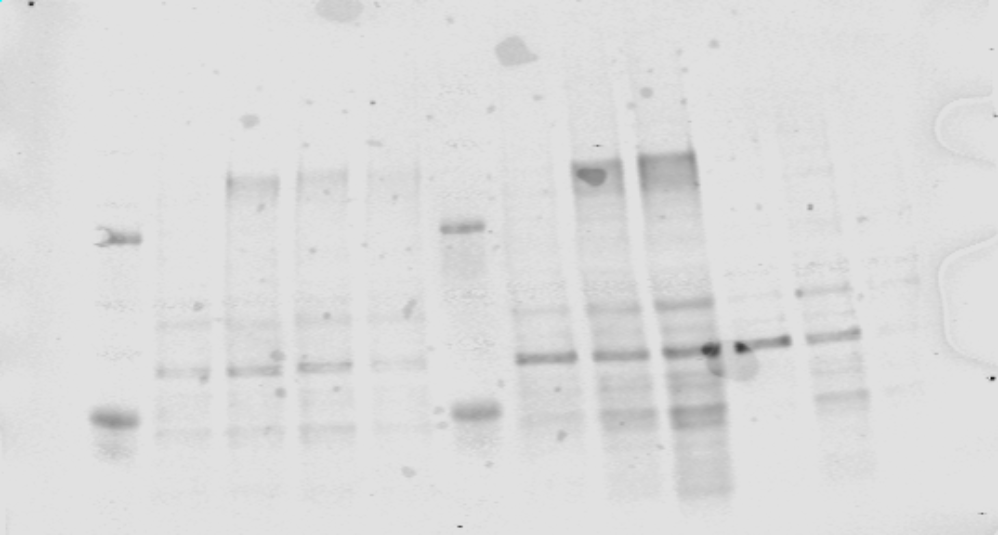

Supplement: Supplementary file 4 — Source data Fig. 2 [file 44318_2024_319_MOESM4_ESM.zip › EMBOJ-2024-117498-T-SourceDataForFigure2B-J/Figure 2 D/HT29_ Biological replicate n1/HT29_western_HIF1.tif]

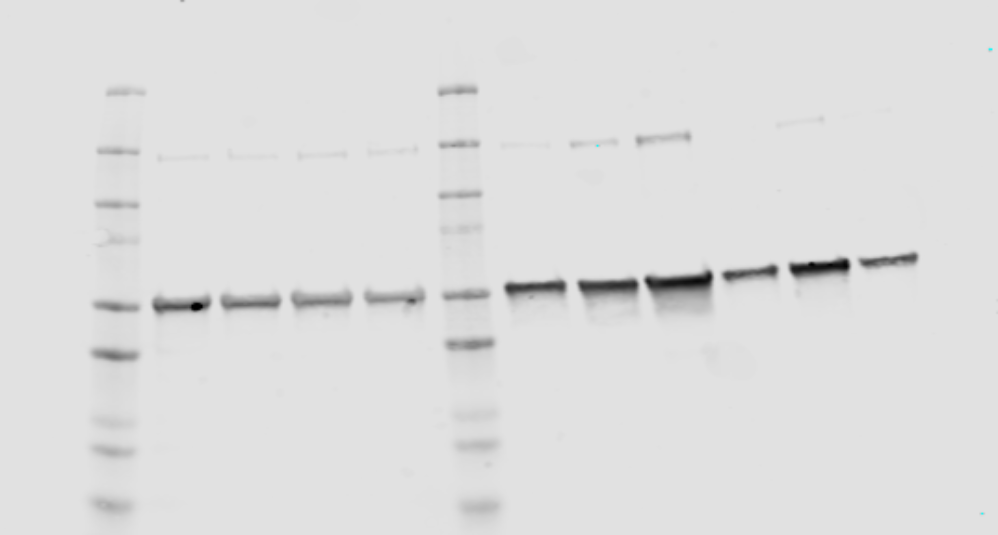

Supplement: Supplementary file 4 — Source data Fig. 2 [file 44318_2024_319_MOESM4_ESM.zip › EMBOJ-2024-117498-T-SourceDataForFigure2B-J/Figure 2 D/HT29_ Biological replicate n1/HT29_western_Tubulin.tif]

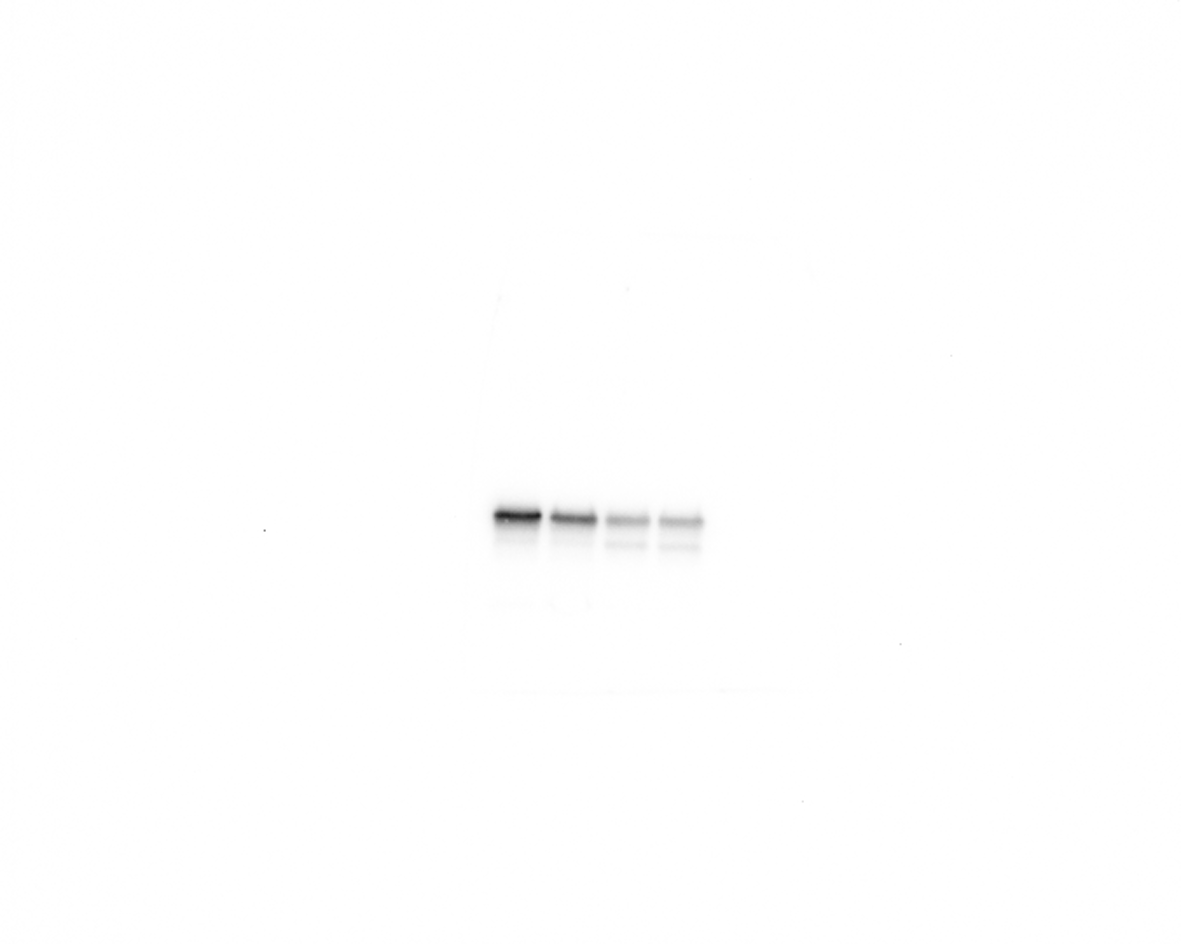

Supplement: Supplementary file 4 — Source data Fig. 2 [file 44318_2024_319_MOESM4_ESM.zip › EMBOJ-2024-117498-T-SourceDataForFigure2B-J/Figure 2 D/HT29_ Biological replicate n2/HT29_ western MHC I.tif]

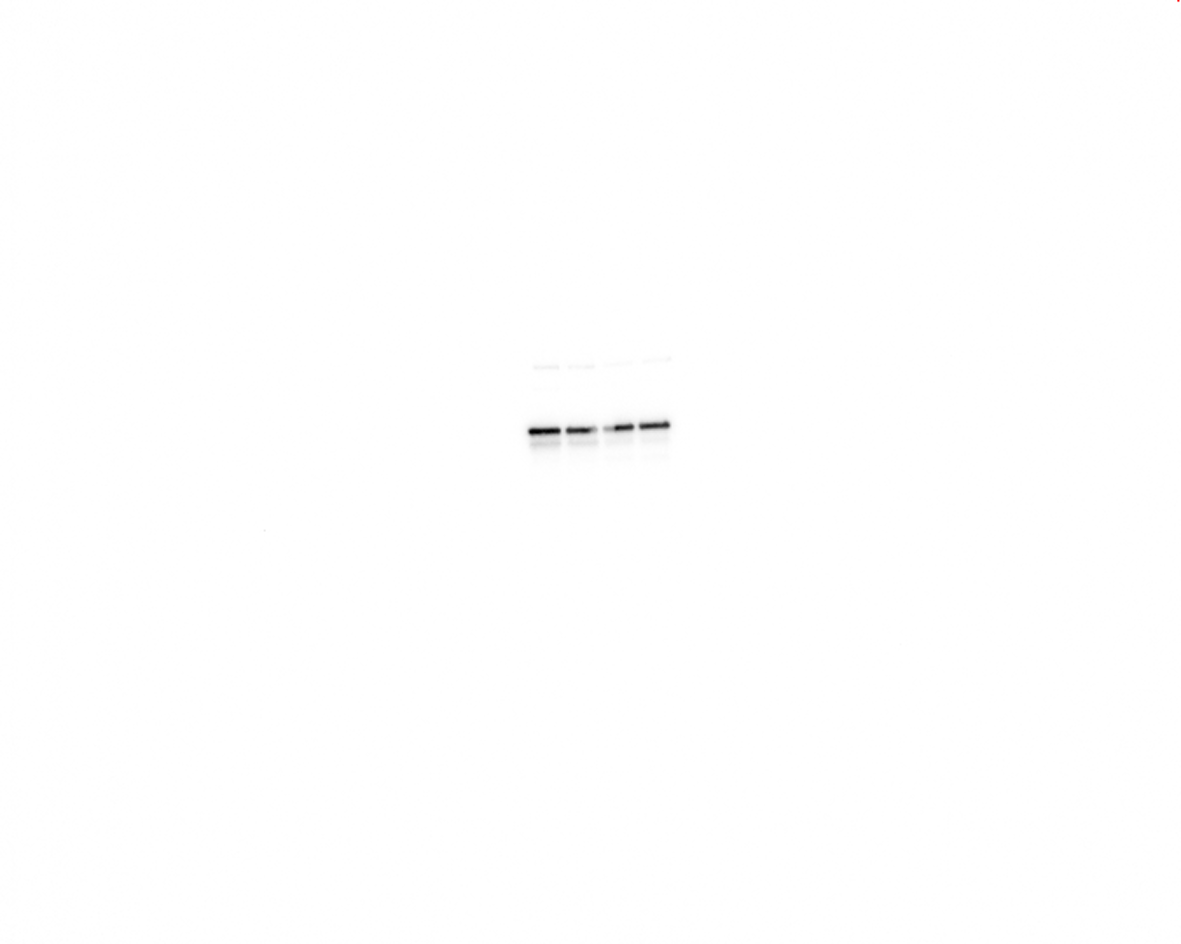

Supplement: Supplementary file 4 — Source data Fig. 2 [file 44318_2024_319_MOESM4_ESM.zip › EMBOJ-2024-117498-T-SourceDataForFigure2B-J/Figure 2 D/HT29_ Biological replicate n2/HT29_ Western Tubulin.tif]

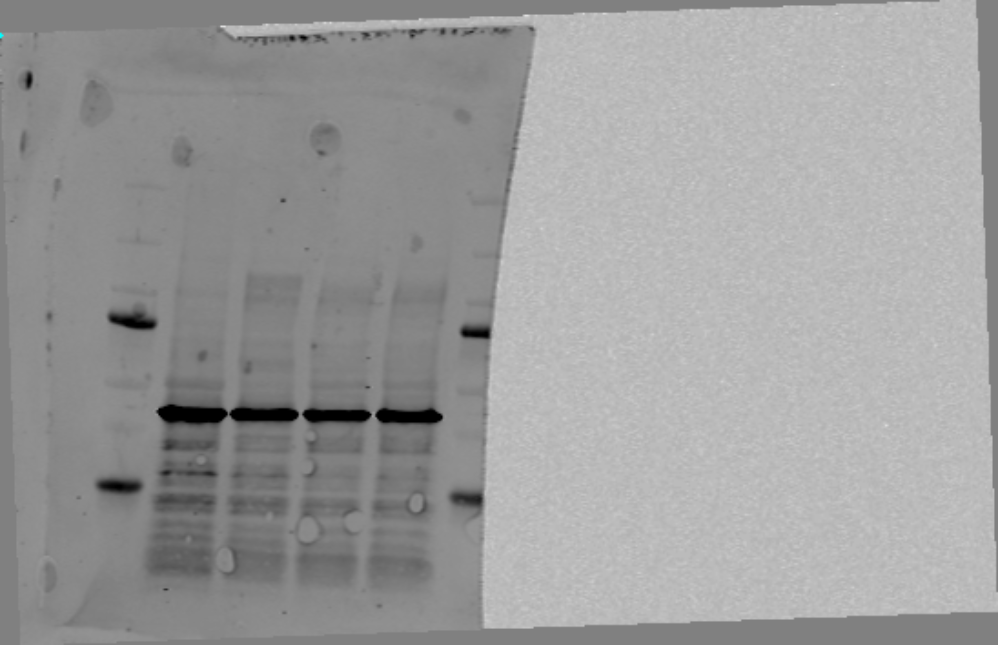

Supplement: Supplementary file 4 — Source data Fig. 2 [file 44318_2024_319_MOESM4_ESM.zip › EMBOJ-2024-117498-T-SourceDataForFigure2B-J/Figure 2 D/HT29_ Biological replicate n2/HT29_HIF_western.tif.tif]

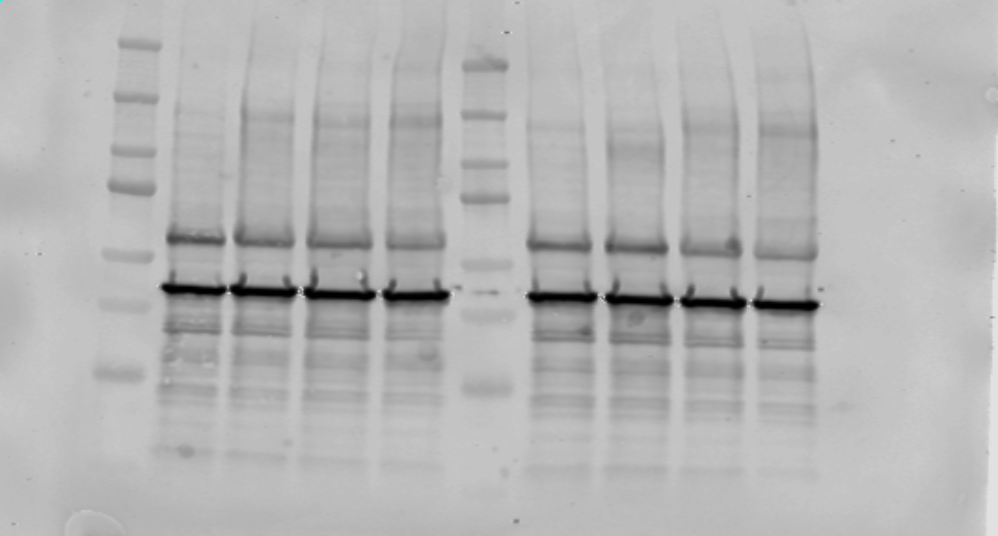

Supplement: Supplementary file 4 — Source data Fig. 2 [file 44318_2024_319_MOESM4_ESM.zip › EMBOJ-2024-117498-T-SourceDataForFigure2B-J/Figure 2 D/HT29_Biological replicate n3/HT29_HIF_western.tif.tif]

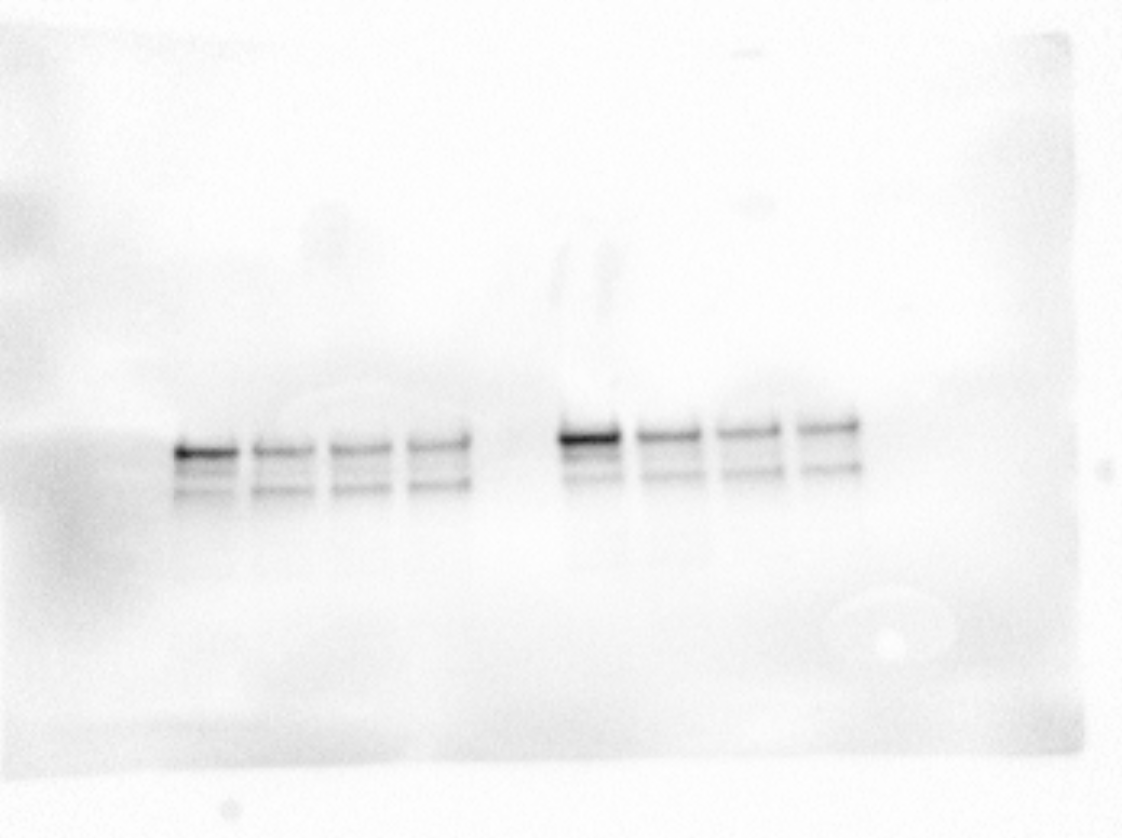

Supplement: Supplementary file 4 — Source data Fig. 2 [file 44318_2024_319_MOESM4_ESM.zip › EMBOJ-2024-117498-T-SourceDataForFigure2B-J/Figure 2 D/HT29_Biological replicate n3/HT29_MHC I_western.tif]

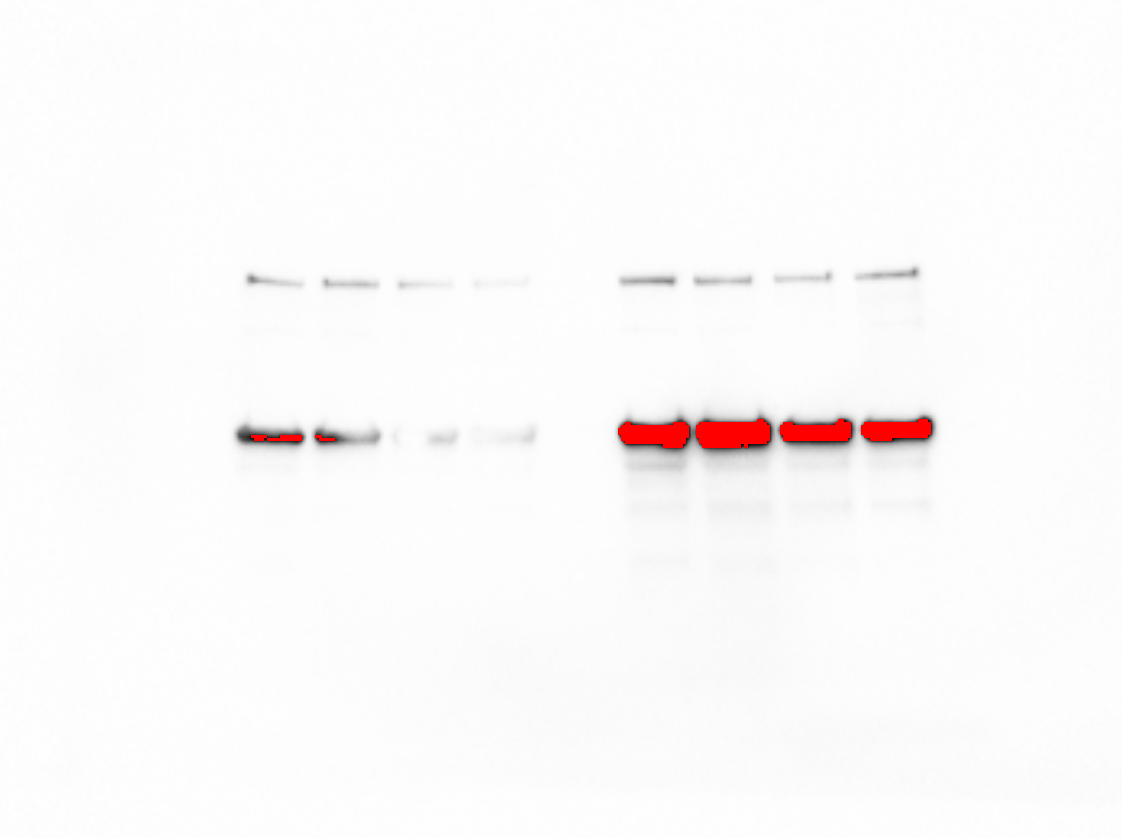

Supplement: Supplementary file 4 — Source data Fig. 2 [file 44318_2024_319_MOESM4_ESM.zip › EMBOJ-2024-117498-T-SourceDataForFigure2B-J/Figure 2 D/HT29_Biological replicate n3/HT29_Tubulin_western.tif]

HT29

MHC I

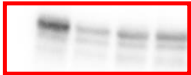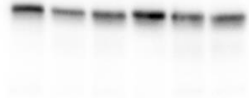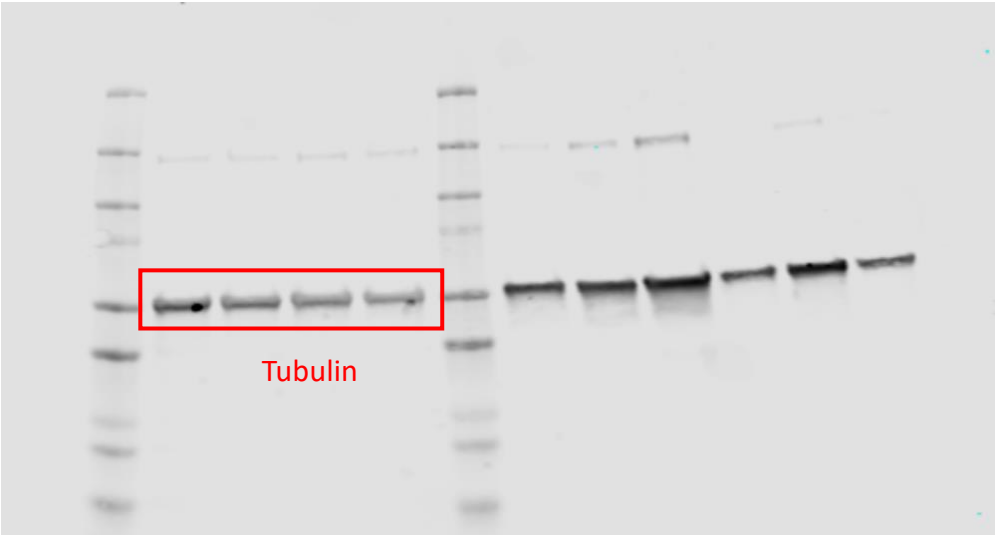

Tubulin

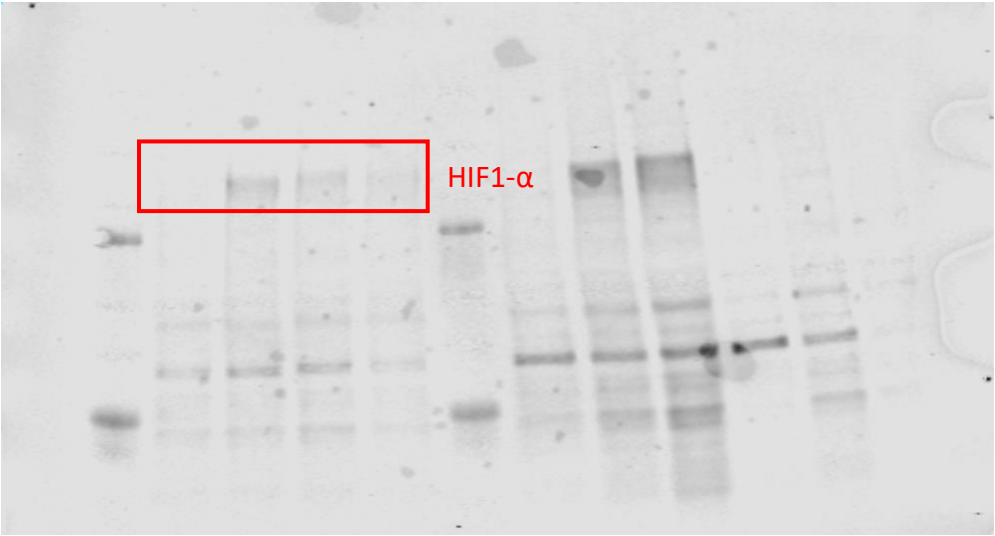

HIF1-α

MHC I

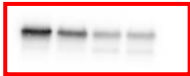

Tubulin

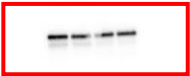

HIF1- $\alpha$

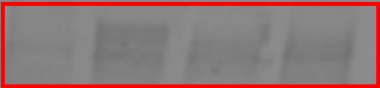

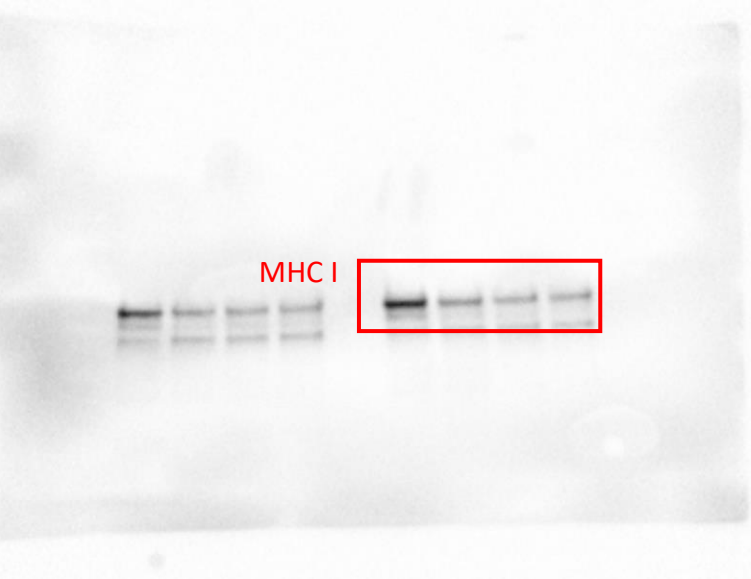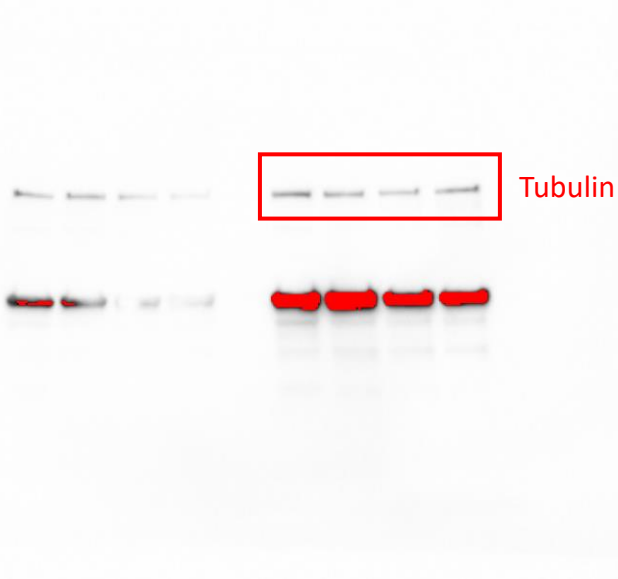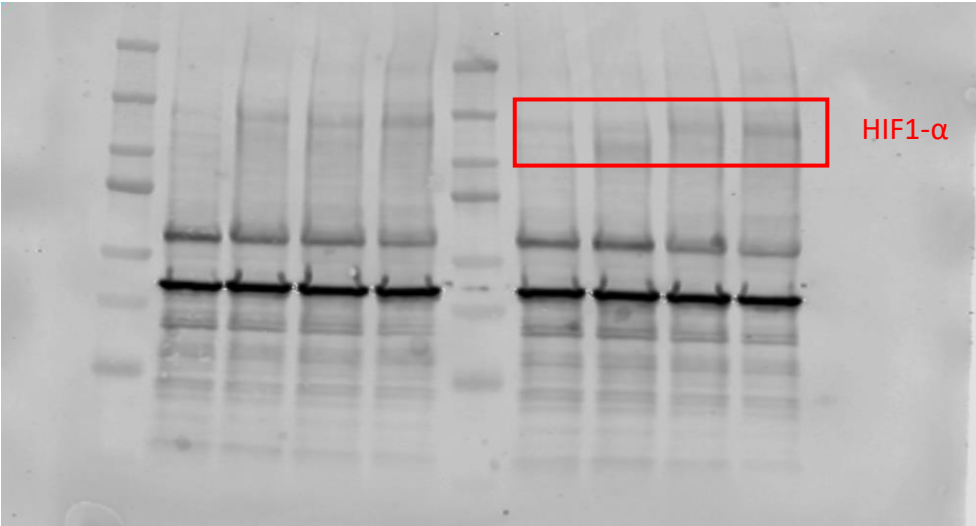

Supplement: Supplementary file 4 — Source data Fig. 2 [file 44318_2024_319_MOESM4_ESM.zip › EMBOJ-2024-117498-T-SourceDataForFigure2B-J/Figure 2 D/README/HT29_western_Biological replicates.pdf]

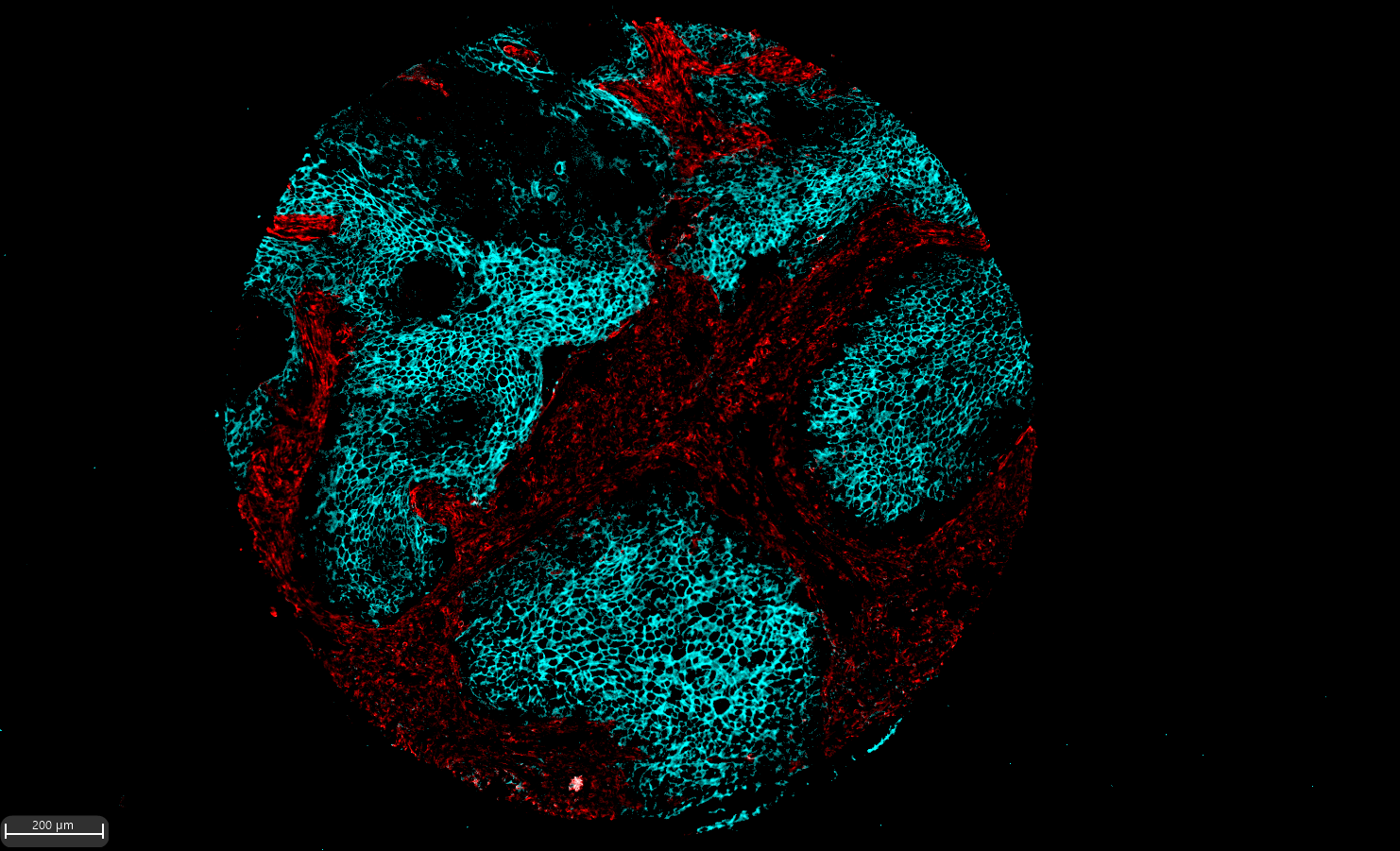

Supplement: Supplementary file 4 — Source data Fig. 2 [file 44318_2024_319_MOESM4_ESM.zip › EMBOJ-2024-117498-T-SourceDataForFigure2B-J/Figure 2 F/Core 1 MHC I CAIX.tiff]

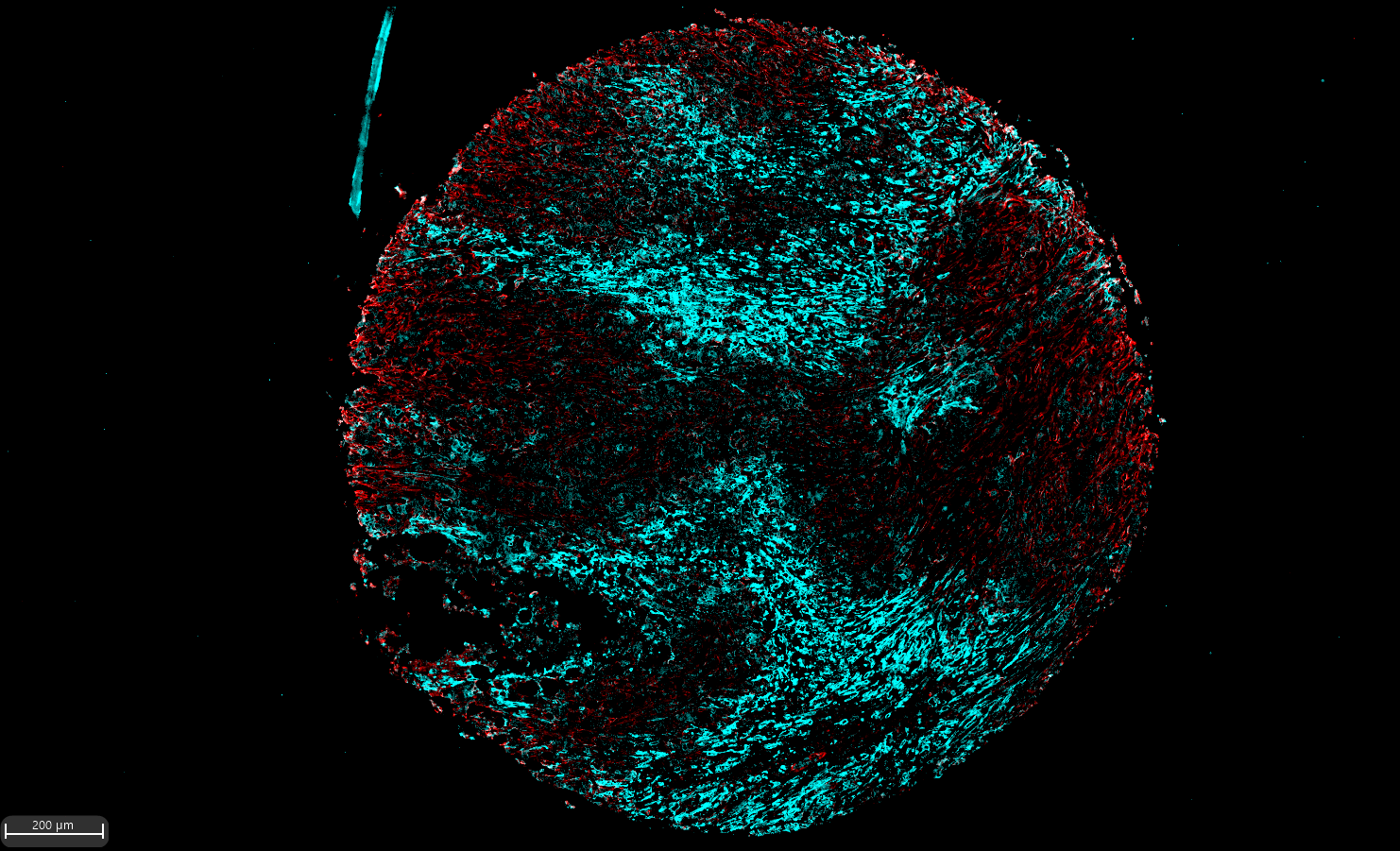

Supplement: Supplementary file 4 — Source data Fig. 2 [file 44318_2024_319_MOESM4_ESM.zip › EMBOJ-2024-117498-T-SourceDataForFigure2B-J/Figure 2 F/Core 2 MHC I CAIX.tiff]

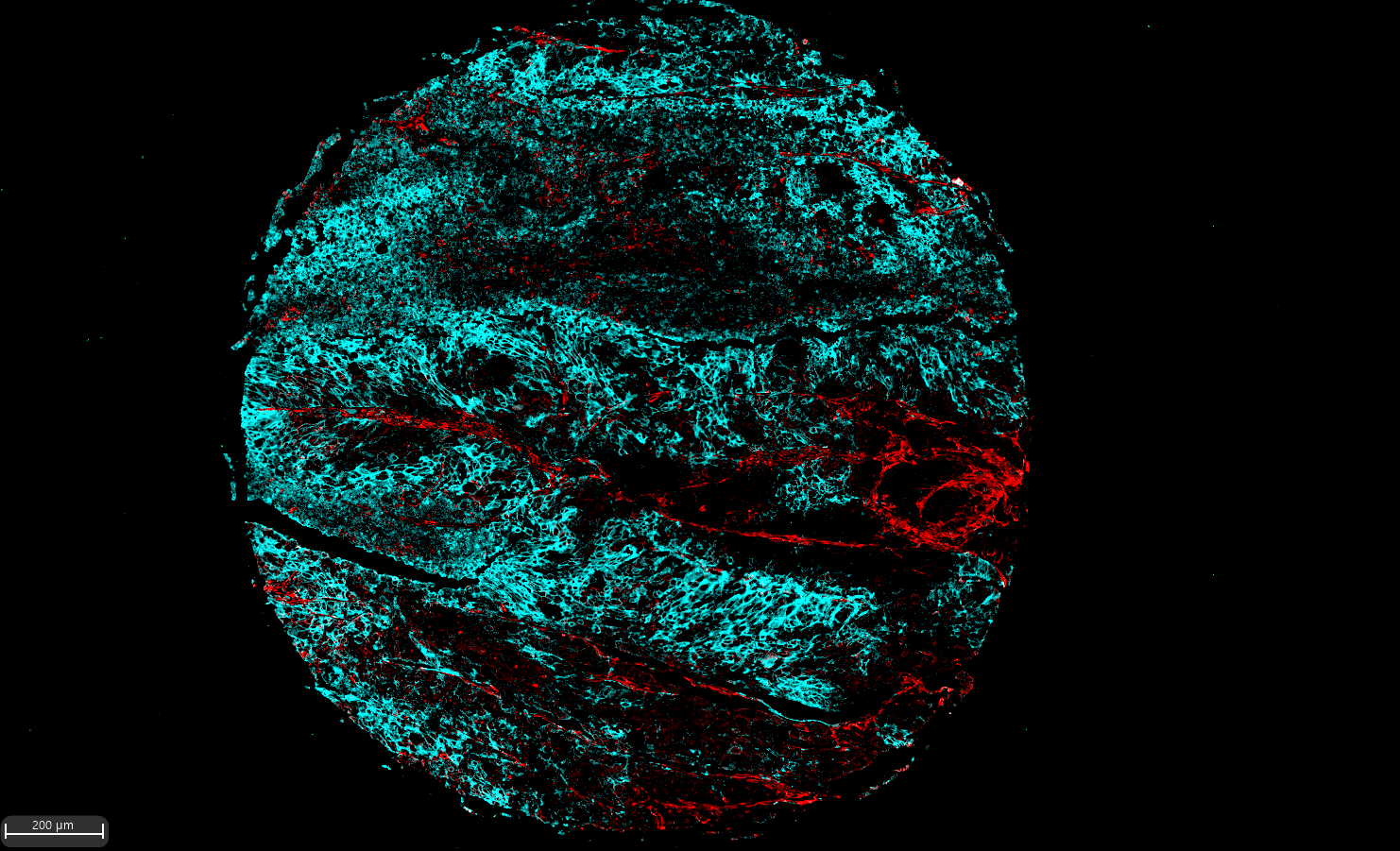

Supplement: Supplementary file 4 — Source data Fig. 2 [file 44318_2024_319_MOESM4_ESM.zip › EMBOJ-2024-117498-T-SourceDataForFigure2B-J/Figure 2 F/Core 3 MHC I CAIX.tiff]

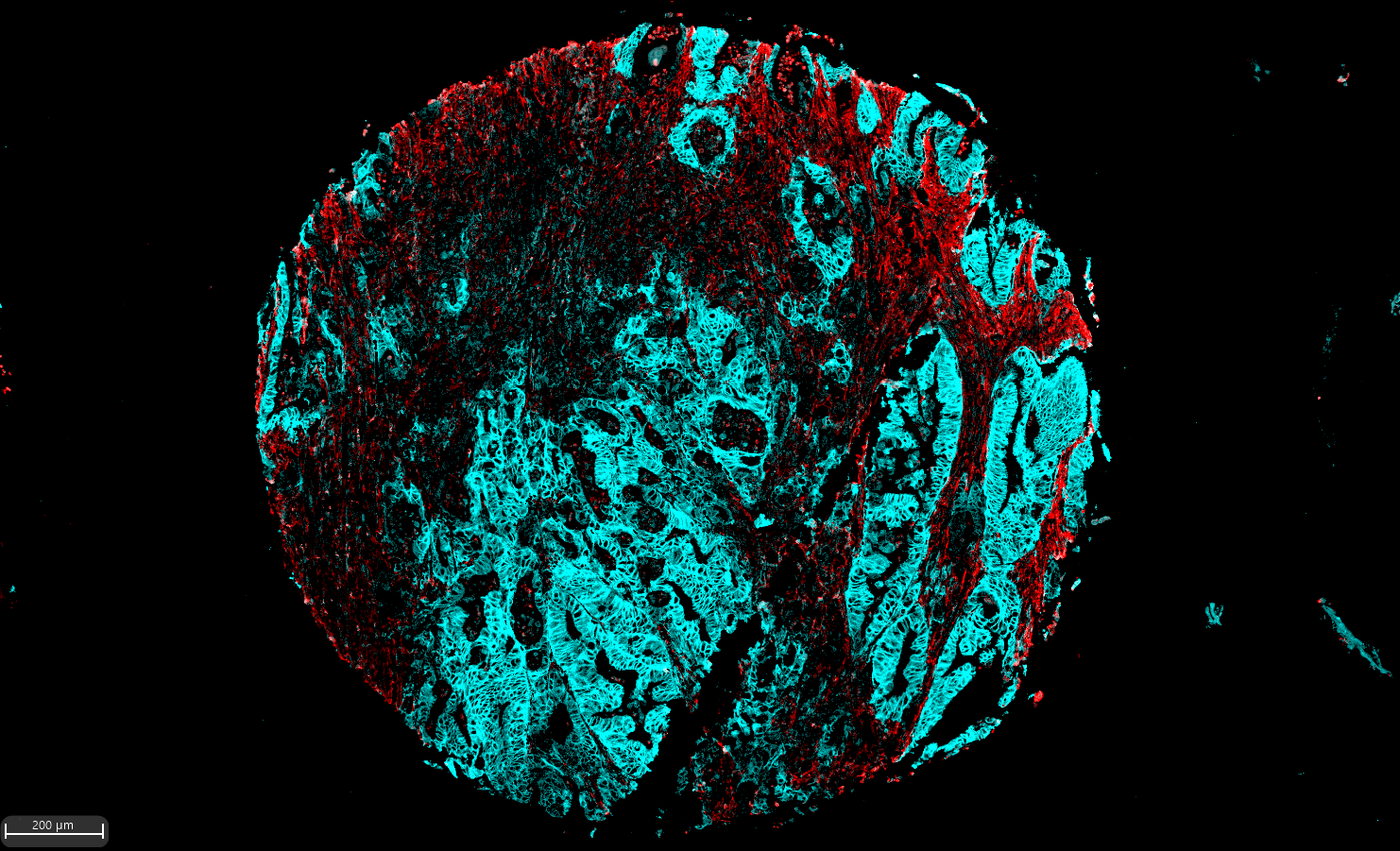

Supplement: Supplementary file 4 — Source data Fig. 2 [file 44318_2024_319_MOESM4_ESM.zip › EMBOJ-2024-117498-T-SourceDataForFigure2B-J/Figure 2 F/Core 4 MHC I CAIX.tiff]

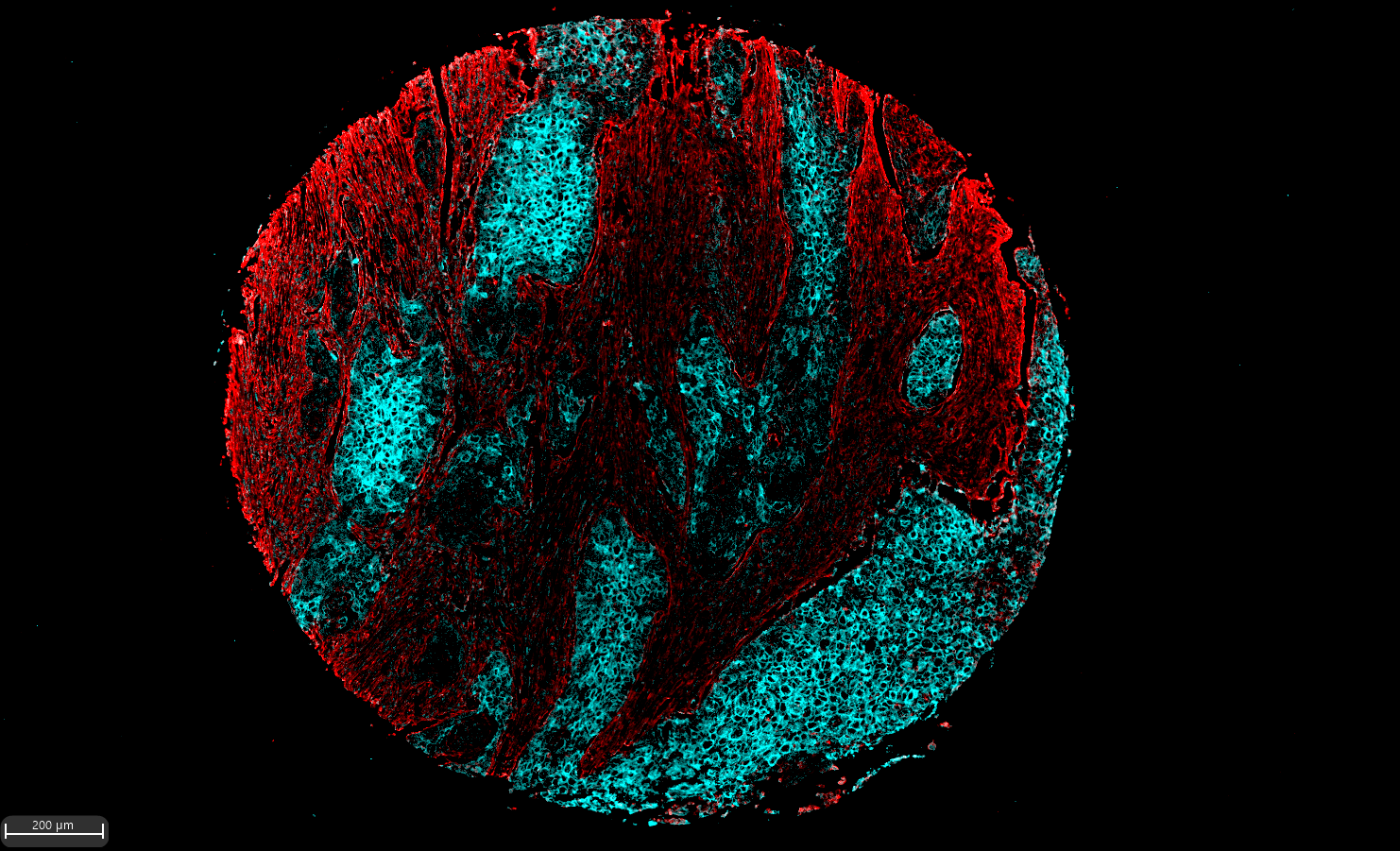

Supplement: Supplementary file 4 — Source data Fig. 2 [file 44318_2024_319_MOESM4_ESM.zip › EMBOJ-2024-117498-T-SourceDataForFigure2B-J/Figure 2 F/Core 5 MHC I CAIX.tiff]

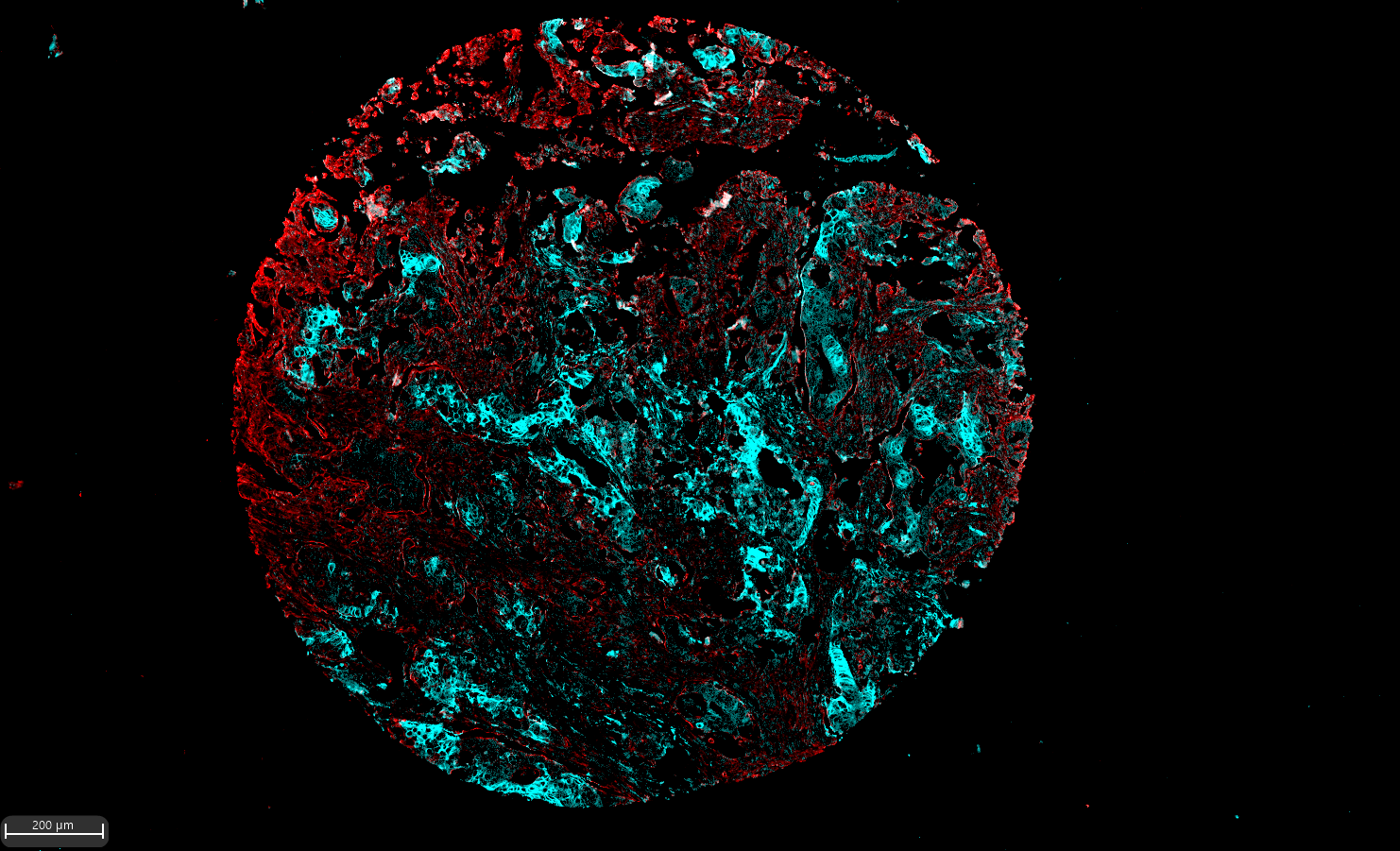

Supplement: Supplementary file 4 — Source data Fig. 2 [file 44318_2024_319_MOESM4_ESM.zip › EMBOJ-2024-117498-T-SourceDataForFigure2B-J/Figure 2 F/Core 6 MHC I CAIX.tiff]

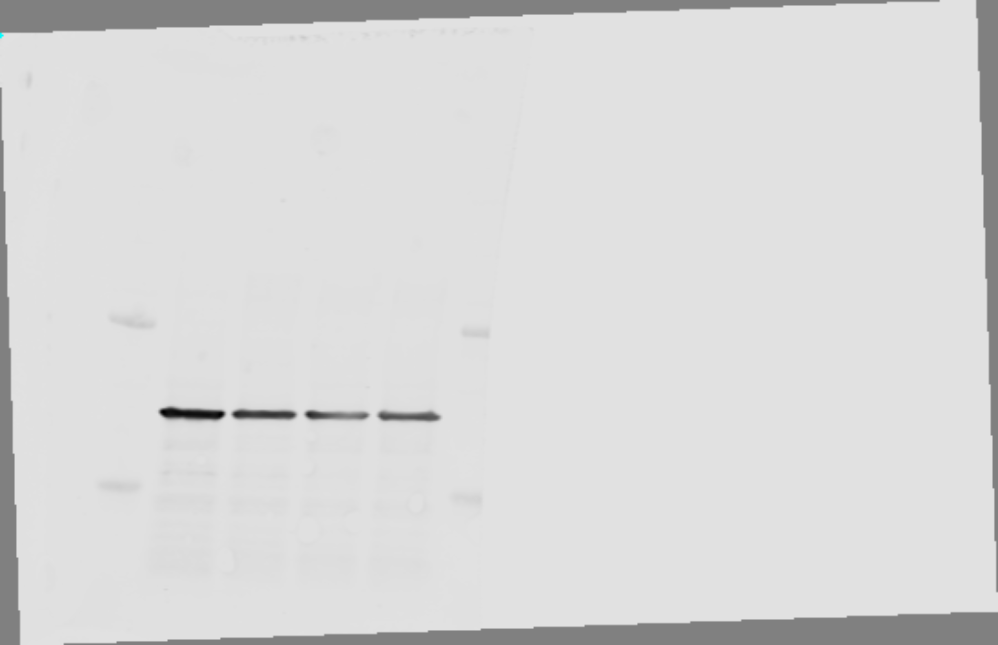

Supplement: Supplementary file 5 — Source data Fig. 3 [file 44318_2024_319_MOESM5_ESM.zip › EMBOJ-2024-117498-T-SourceDataForFigure3A-J/Figure 3 A/HT29_biological replicate n1_western/HT29_Actin_Western.tif]

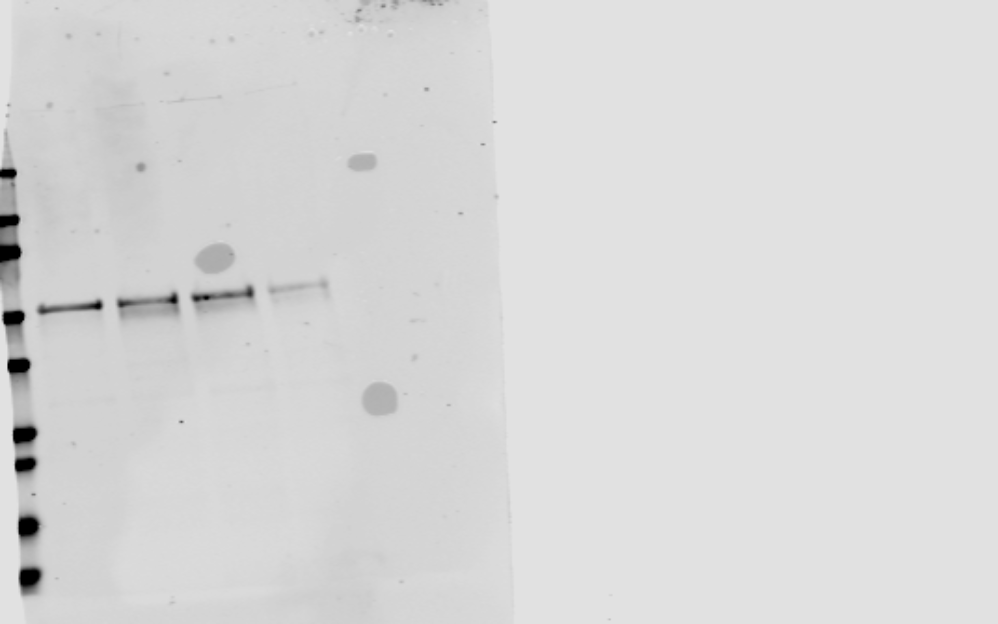

Supplement: Supplementary file 5 — Source data Fig. 3 [file 44318_2024_319_MOESM5_ESM.zip › EMBOJ-2024-117498-T-SourceDataForFigure3A-J/Figure 3 A/HT29_biological replicate n1_western/HT29_ATF4_Western.tif]

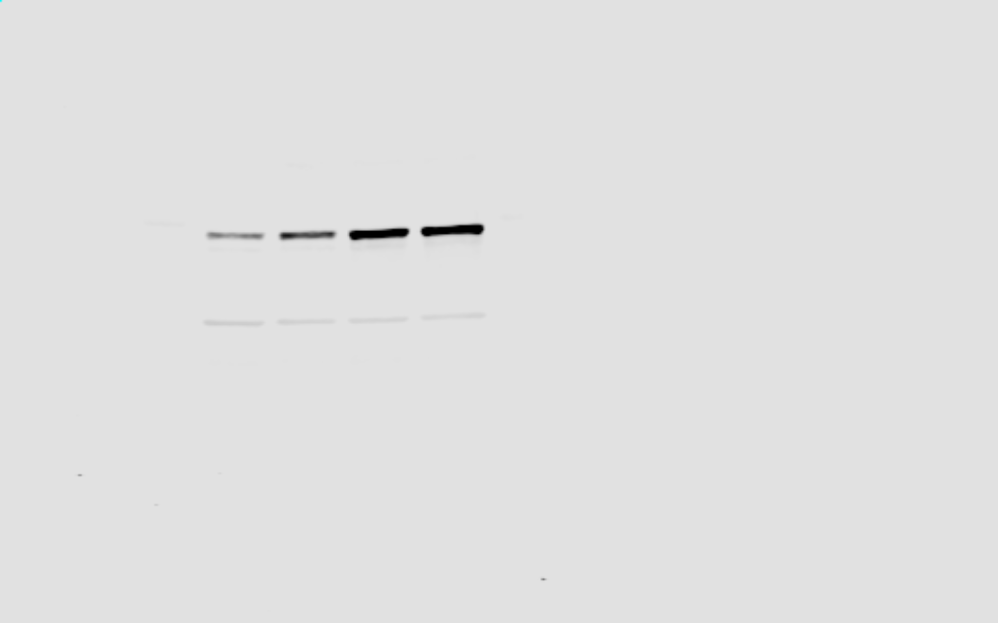

Supplement: Supplementary file 5 — Source data Fig. 3 [file 44318_2024_319_MOESM5_ESM.zip › EMBOJ-2024-117498-T-SourceDataForFigure3A-J/Figure 3 A/HT29_biological replicate n1_western/HT29_GRP78_Western.tif]

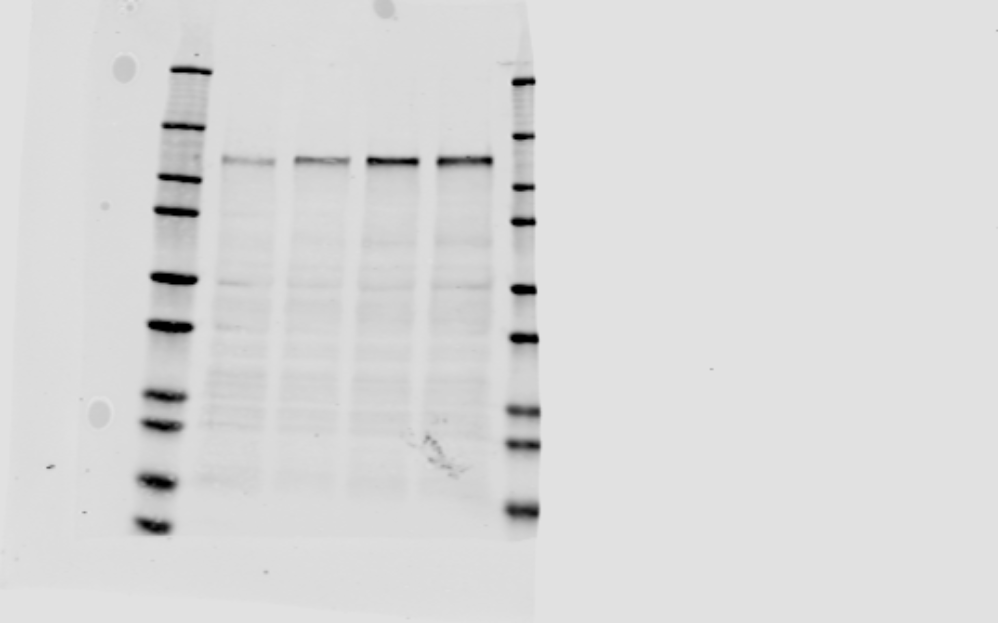

Supplement: Supplementary file 5 — Source data Fig. 3 [file 44318_2024_319_MOESM5_ESM.zip › EMBOJ-2024-117498-T-SourceDataForFigure3A-J/Figure 3 A/HT29_biological replicate n1_western/HT29_IRE_Western.tif]

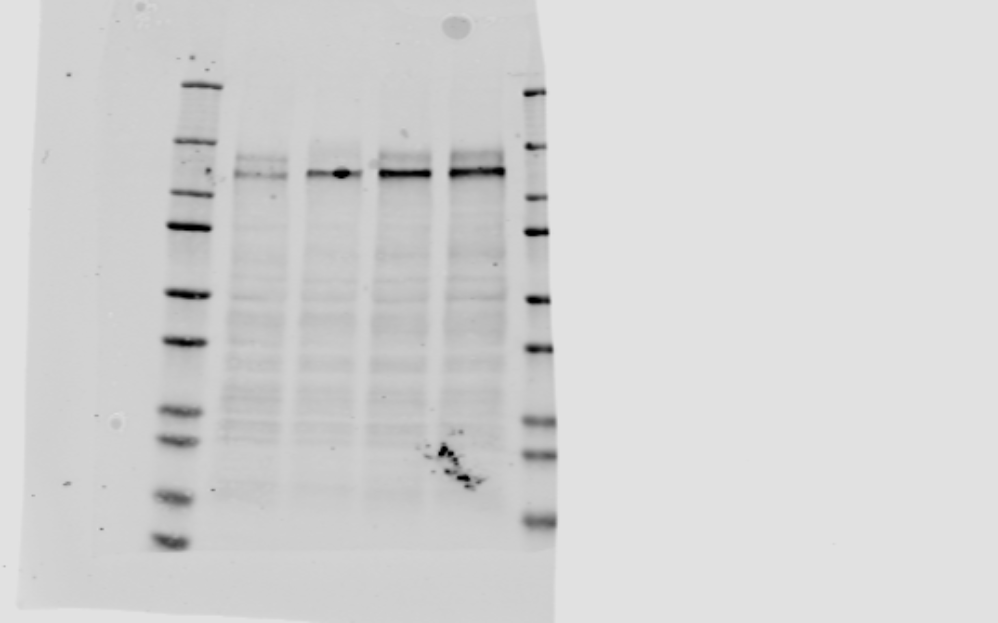

Supplement: Supplementary file 5 — Source data Fig. 3 [file 44318_2024_319_MOESM5_ESM.zip › EMBOJ-2024-117498-T-SourceDataForFigure3A-J/Figure 3 A/HT29_biological replicate n1_western/HT29_PERK_Western.tif]

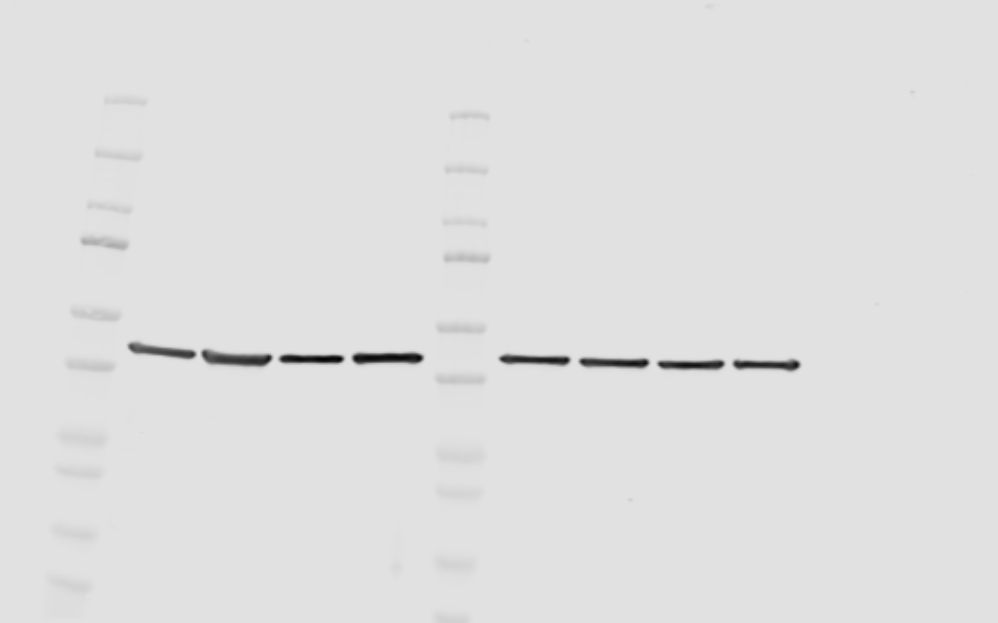

Supplement: Supplementary file 5 — Source data Fig. 3 [file 44318_2024_319_MOESM5_ESM.zip › EMBOJ-2024-117498-T-SourceDataForFigure3A-J/Figure 3 A/HT29_biological replicate n2_western/HT29_Actin_Western.tif]

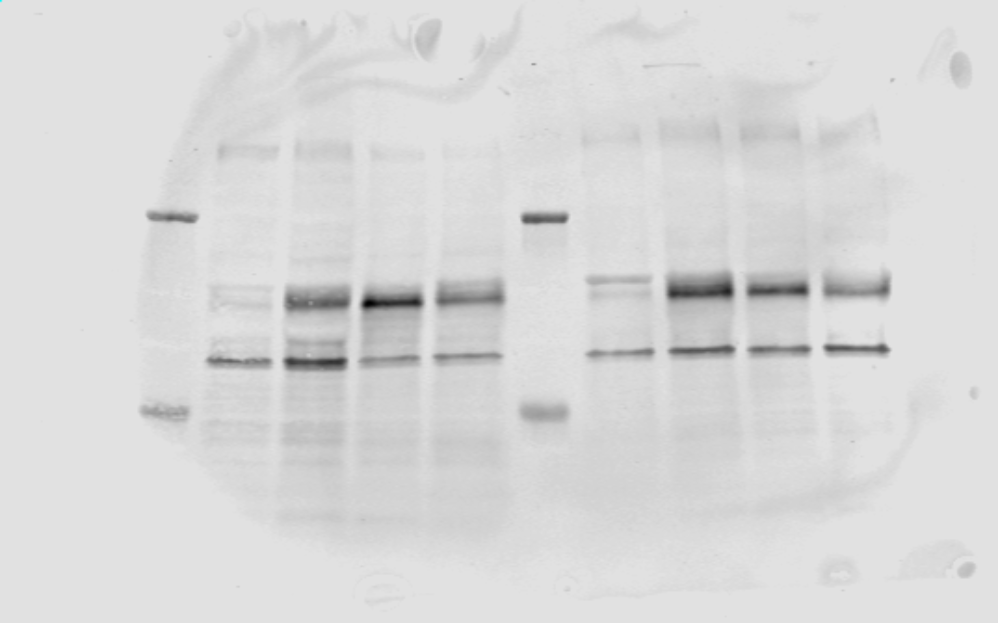

Supplement: Supplementary file 5 — Source data Fig. 3 [file 44318_2024_319_MOESM5_ESM.zip › EMBOJ-2024-117498-T-SourceDataForFigure3A-J/Figure 3 A/HT29_biological replicate n2_western/HT29_ATF4_Western.tif]

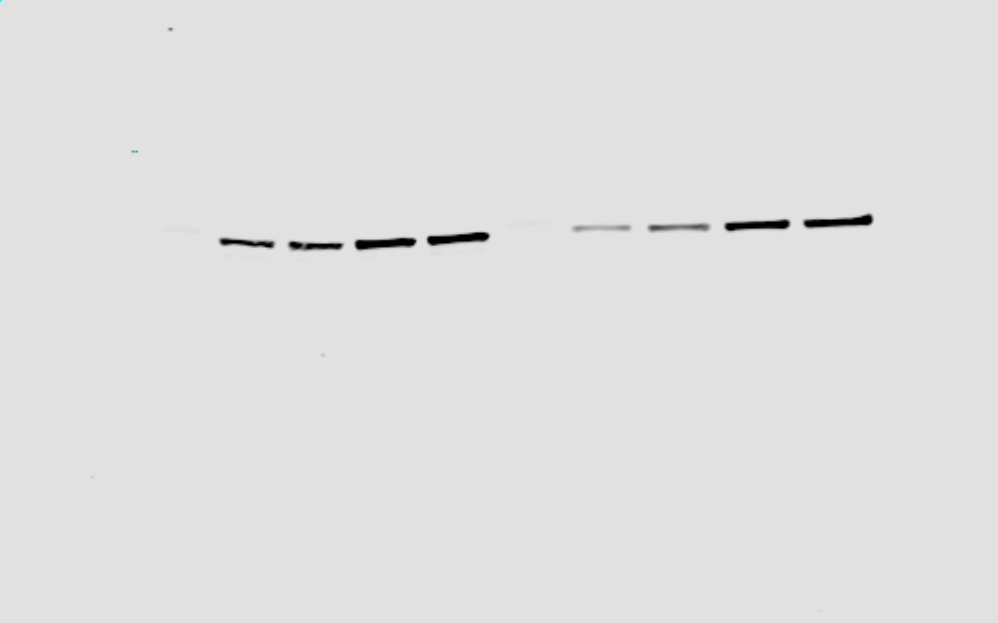

Supplement: Supplementary file 5 — Source data Fig. 3 [file 44318_2024_319_MOESM5_ESM.zip › EMBOJ-2024-117498-T-SourceDataForFigure3A-J/Figure 3 A/HT29_biological replicate n2_western/HT29_GRP78_Western.tif]

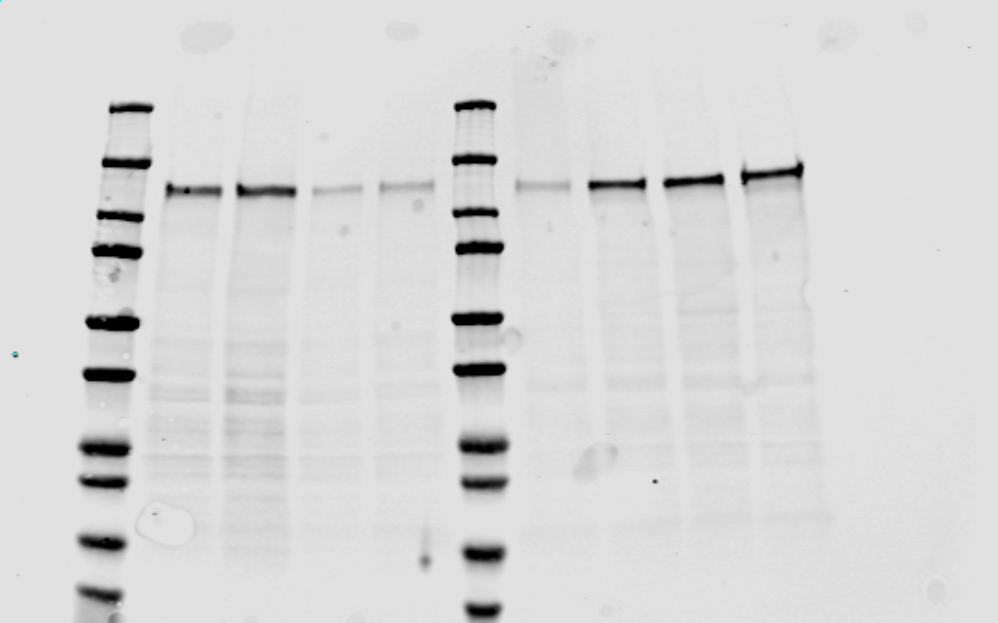

Supplement: Supplementary file 5 — Source data Fig. 3 [file 44318_2024_319_MOESM5_ESM.zip › EMBOJ-2024-117498-T-SourceDataForFigure3A-J/Figure 3 A/HT29_biological replicate n2_western/HT29_IRE_Western.tif]

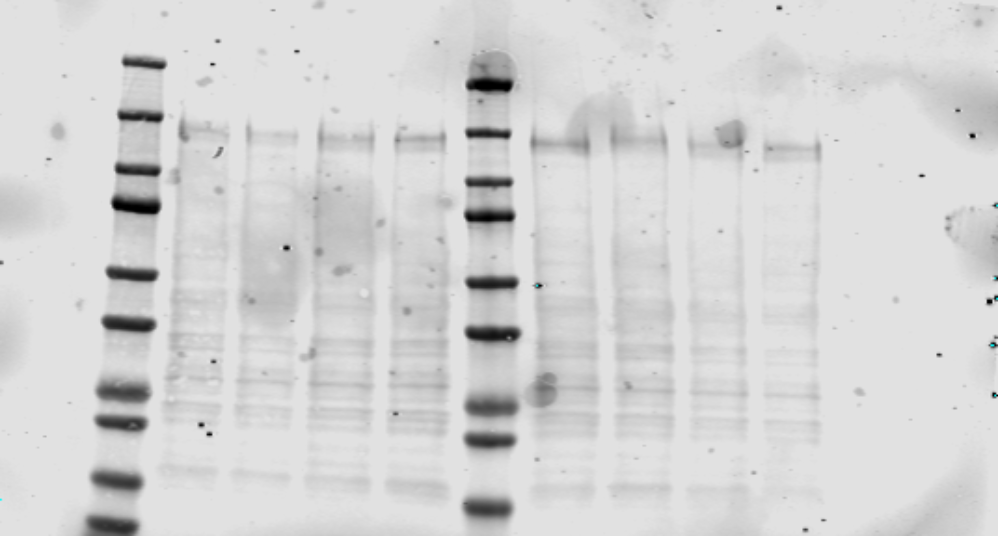

Supplement: Supplementary file 5 — Source data Fig. 3 [file 44318_2024_319_MOESM5_ESM.zip › EMBOJ-2024-117498-T-SourceDataForFigure3A-J/Figure 3 A/HT29_biological replicate n2_western/HT29_PERK_western.tif.tif]

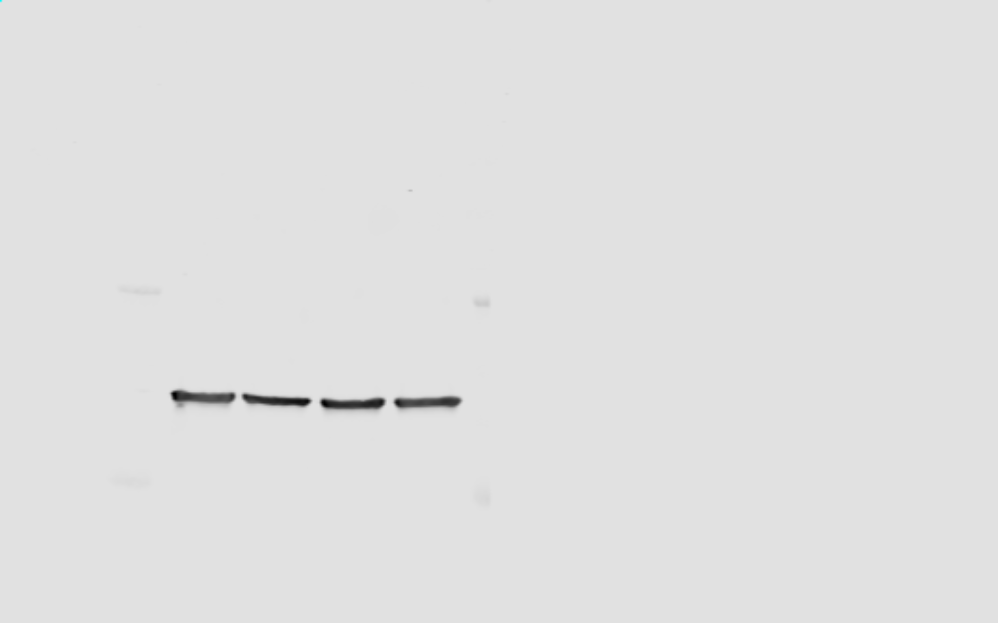

Supplement: Supplementary file 5 — Source data Fig. 3 [file 44318_2024_319_MOESM5_ESM.zip › EMBOJ-2024-117498-T-SourceDataForFigure3A-J/Figure 3 A/HT29_biological replicate n3_western/HT29_Actin_Western.tif]

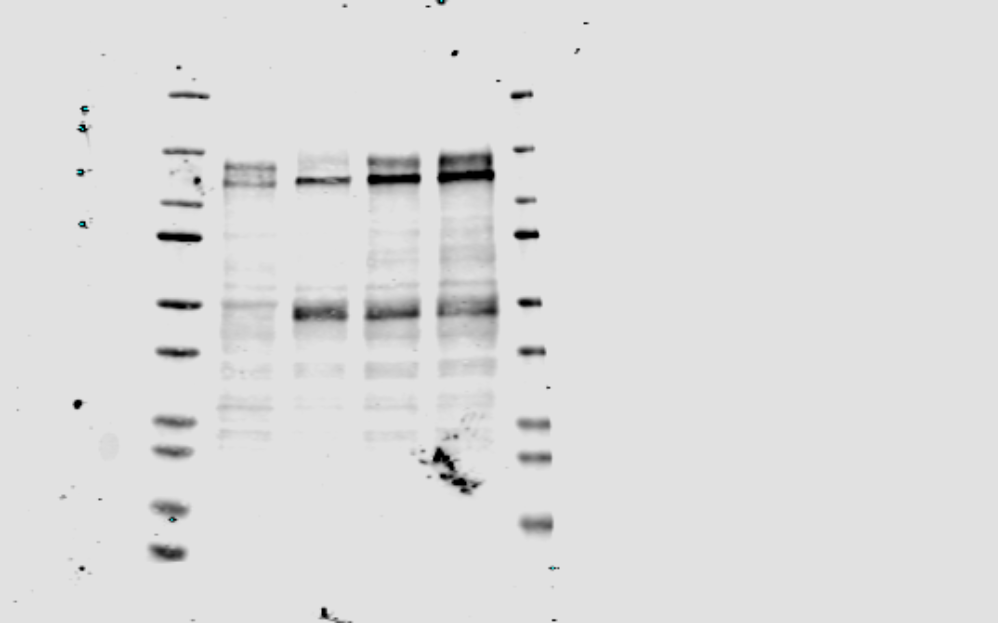

Supplement: Supplementary file 5 — Source data Fig. 3 [file 44318_2024_319_MOESM5_ESM.zip › EMBOJ-2024-117498-T-SourceDataForFigure3A-J/Figure 3 A/HT29_biological replicate n3_western/HT29_ATF4_Western.tif]

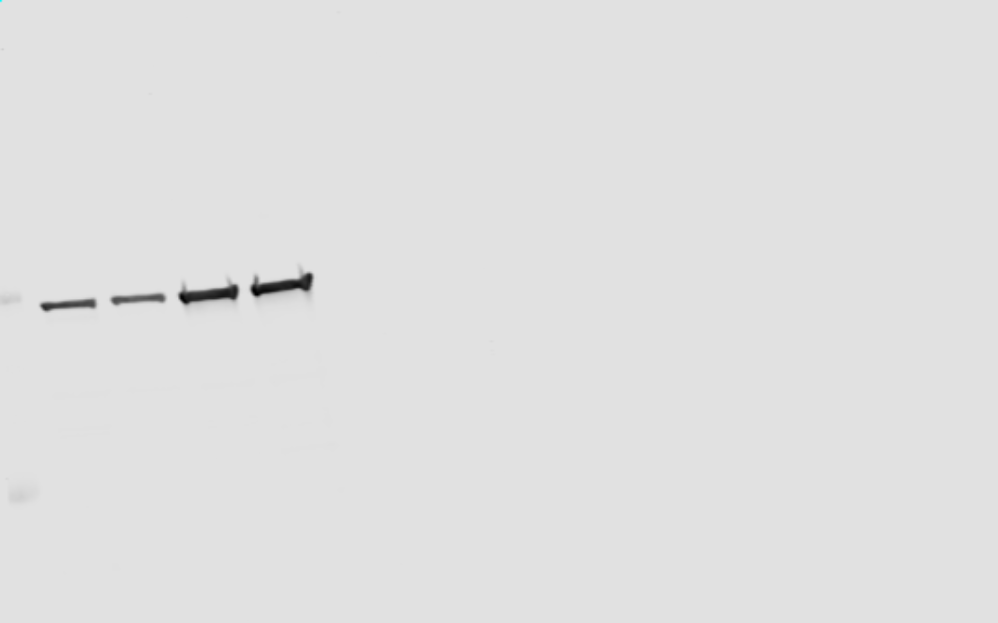

Supplement: Supplementary file 5 — Source data Fig. 3 [file 44318_2024_319_MOESM5_ESM.zip › EMBOJ-2024-117498-T-SourceDataForFigure3A-J/Figure 3 A/HT29_biological replicate n3_western/HT29_GRP78_Western.tif]

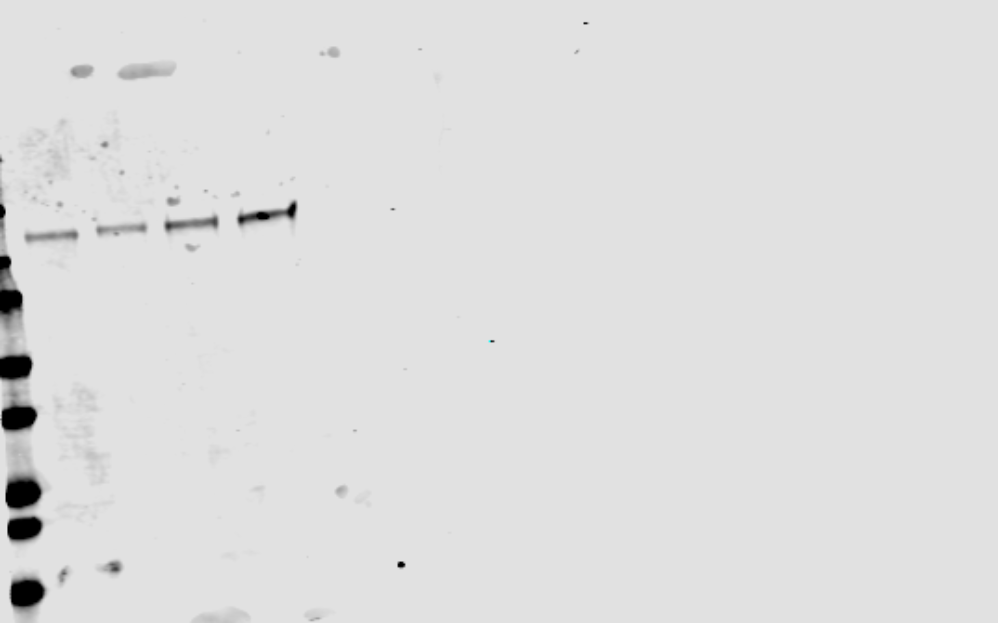

Supplement: Supplementary file 5 — Source data Fig. 3 [file 44318_2024_319_MOESM5_ESM.zip › EMBOJ-2024-117498-T-SourceDataForFigure3A-J/Figure 3 A/HT29_biological replicate n3_western/HT29_IRE_Western.tif]

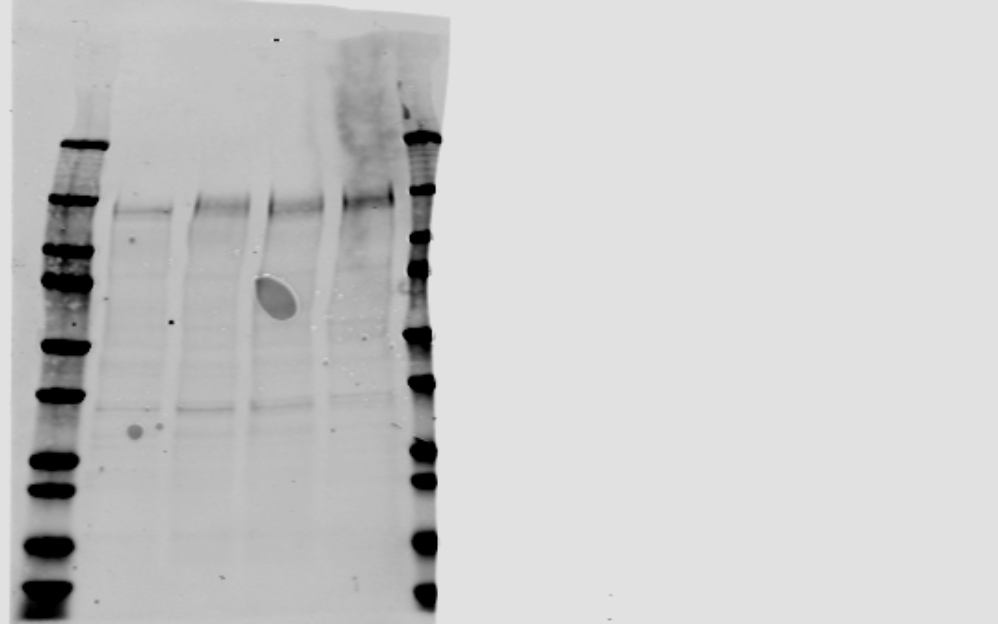

Supplement: Supplementary file 5 — Source data Fig. 3 [file 44318_2024_319_MOESM5_ESM.zip › EMBOJ-2024-117498-T-SourceDataForFigure3A-J/Figure 3 A/HT29_biological replicate n3_western/HT29_PERK_Western.tif]

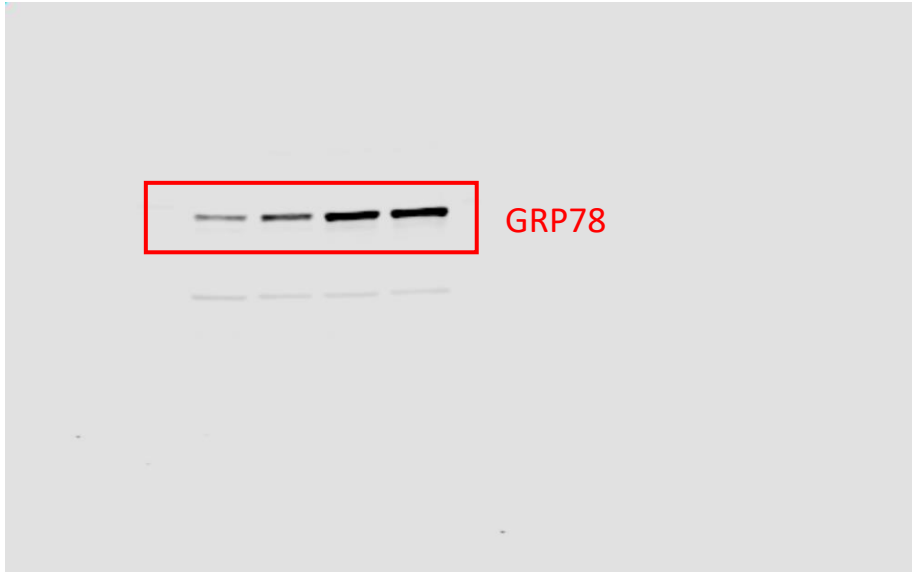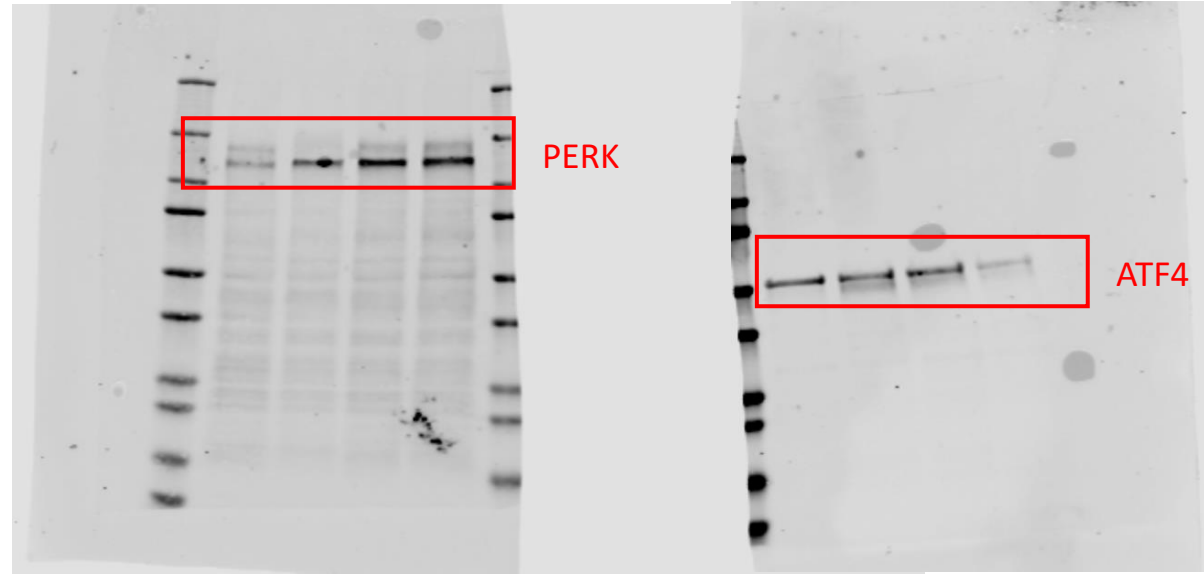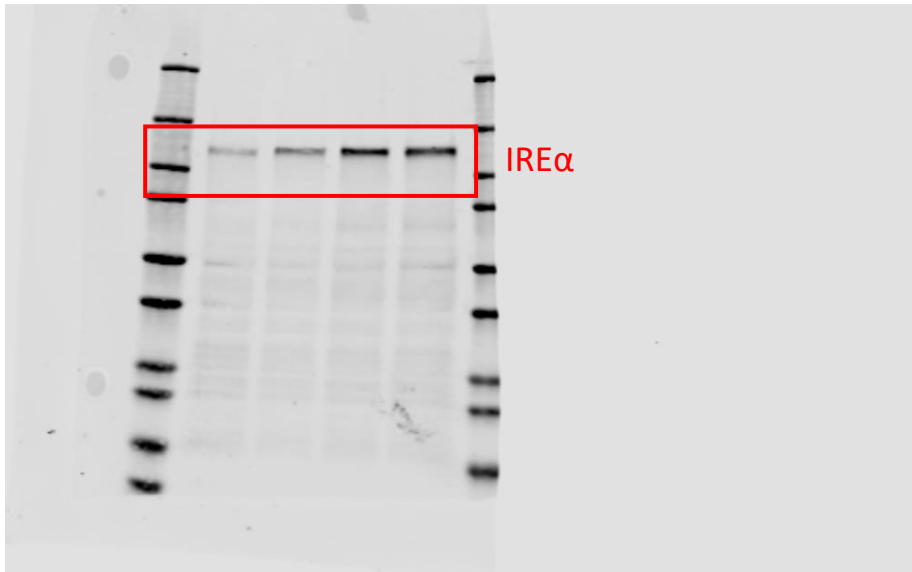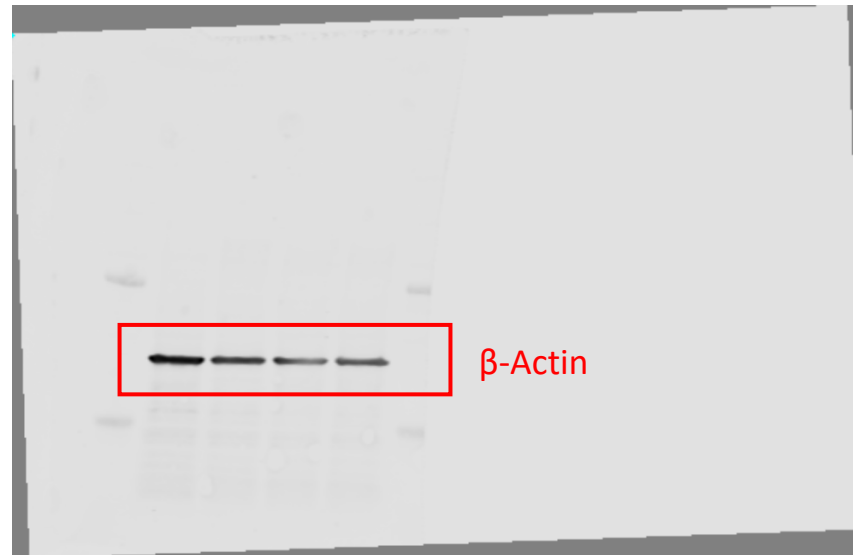

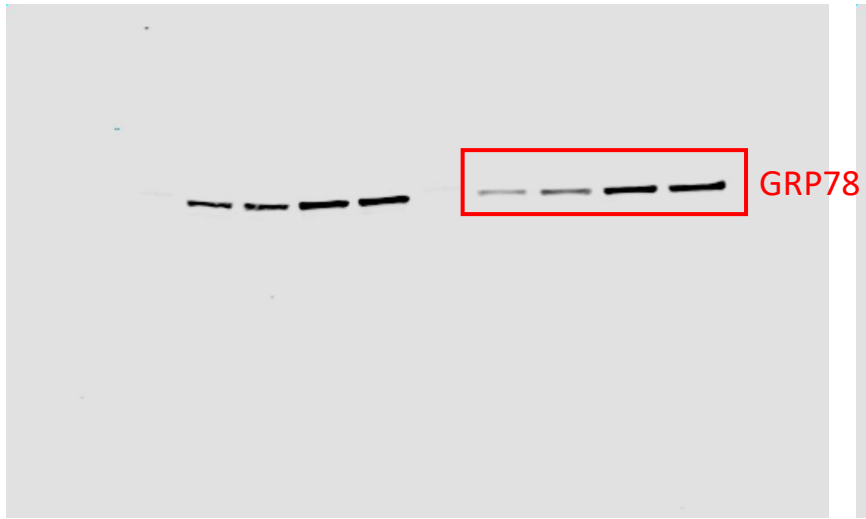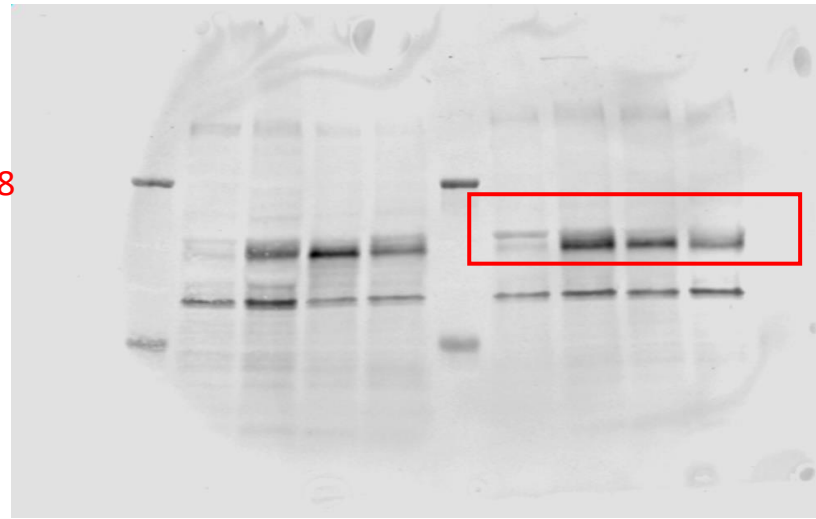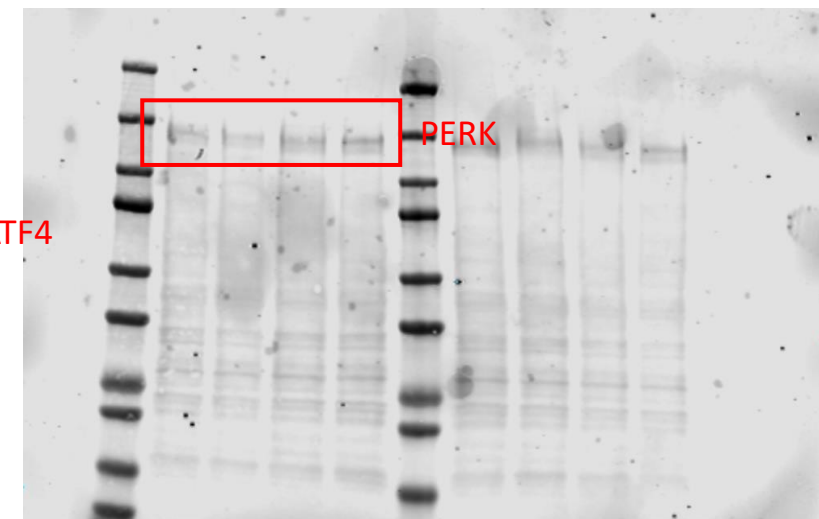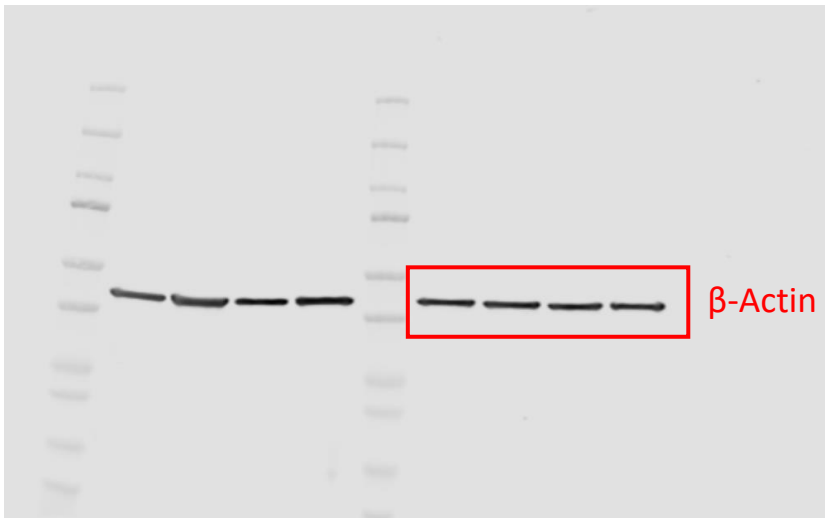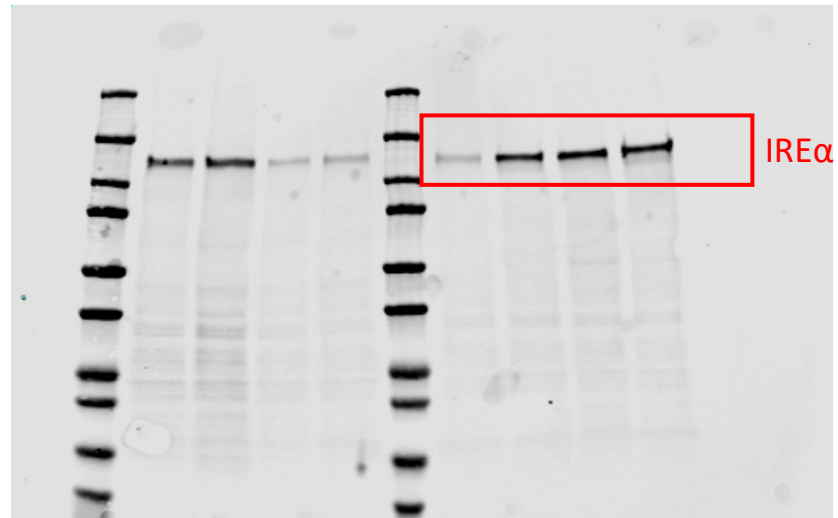

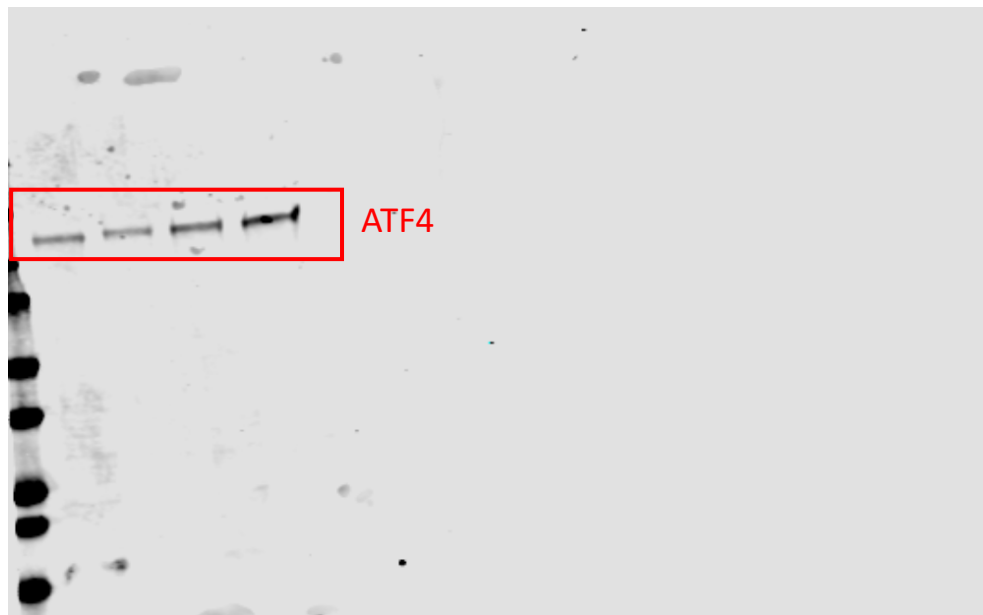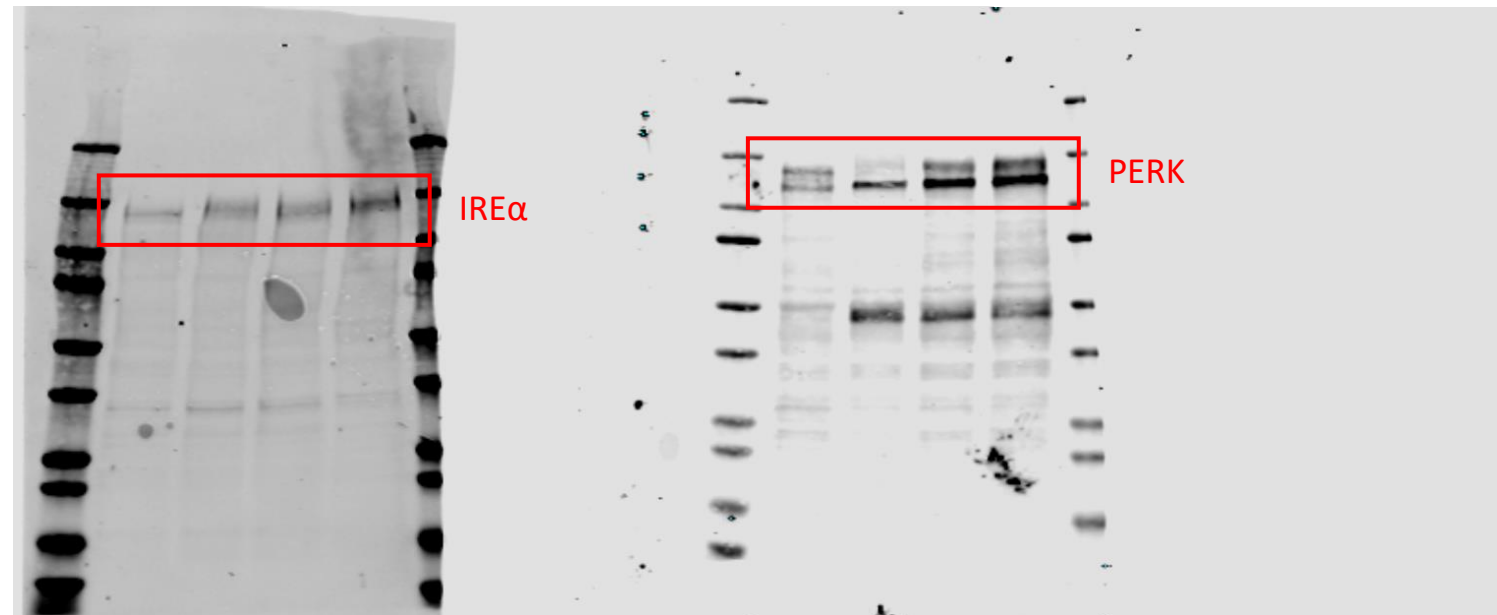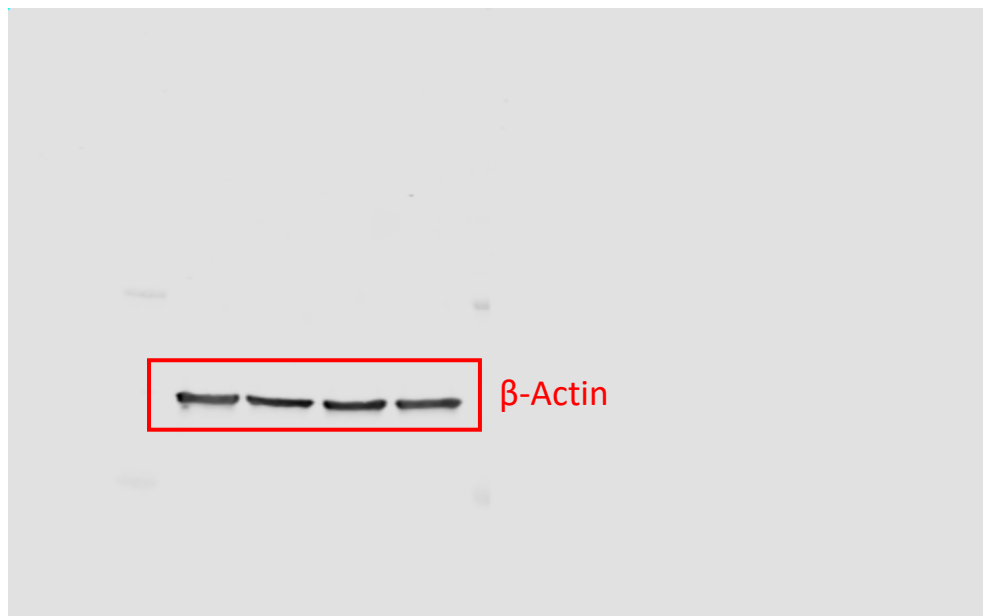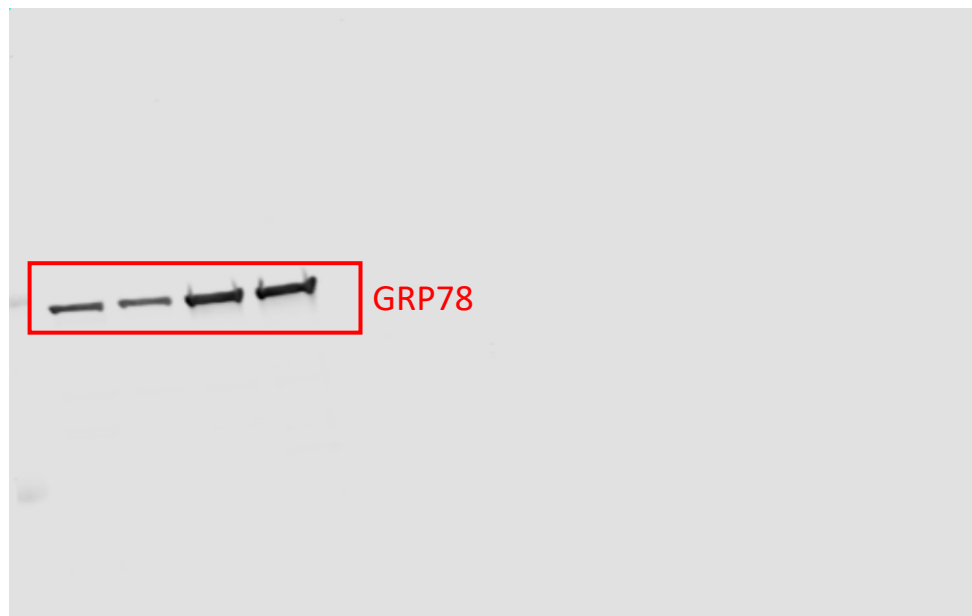

Supplement: Supplementary file 5 — Source data Fig. 3 [file 44318_2024_319_MOESM5_ESM.zip › EMBOJ-2024-117498-T-SourceDataForFigure3A-J/Figure 3 A/README/HT29_biological replicates_western.pdf]

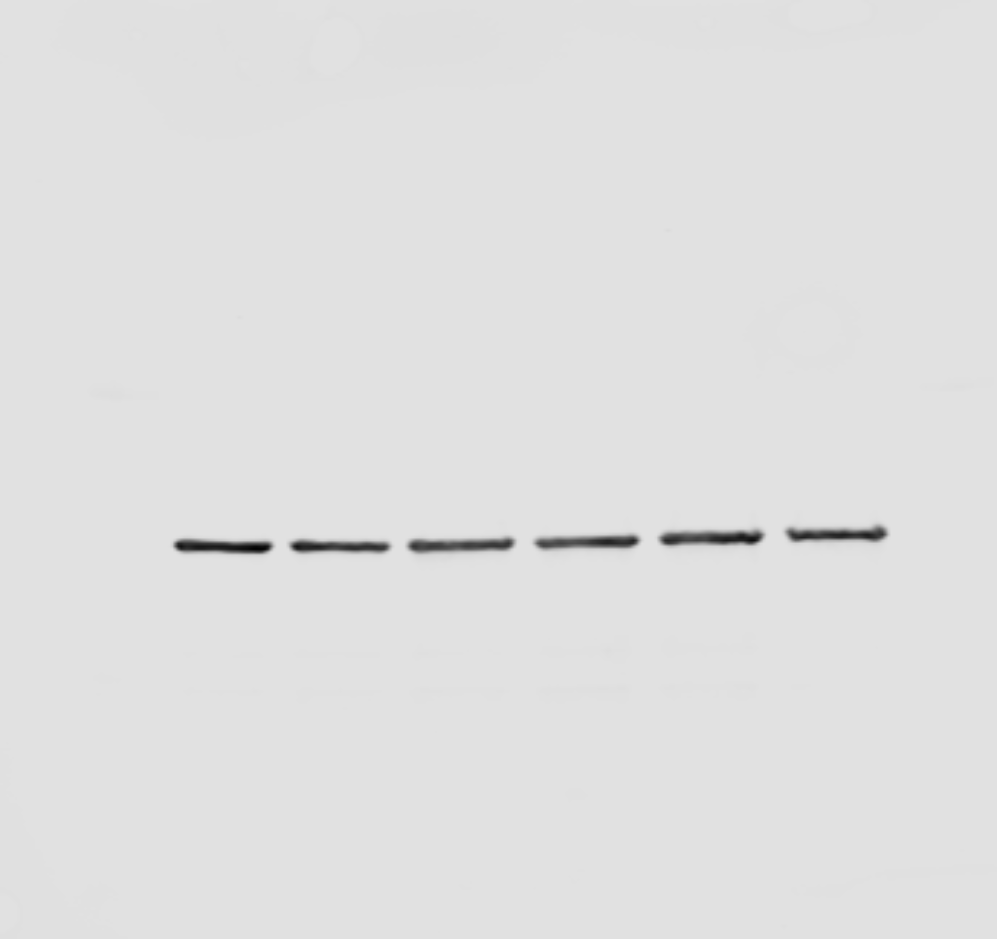

Supplement: Supplementary file 5 — Source data Fig. 3 [file 44318_2024_319_MOESM5_ESM.zip › EMBOJ-2024-117498-T-SourceDataForFigure3A-J/Figure 3 B/HT29_biological replicate n1/HT29_Actin_western.tif]

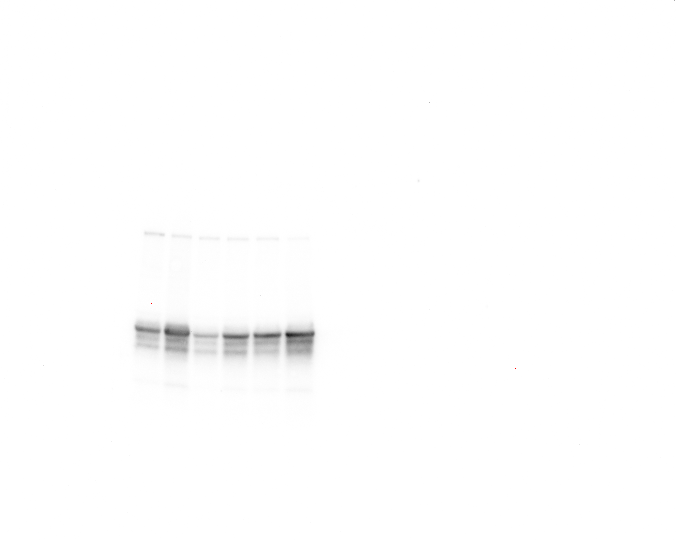

Supplement: Supplementary file 5 — Source data Fig. 3 [file 44318_2024_319_MOESM5_ESM.zip › EMBOJ-2024-117498-T-SourceDataForFigure3A-J/Figure 3 B/HT29_biological replicate n1/HT29_MHC I_western.tif]

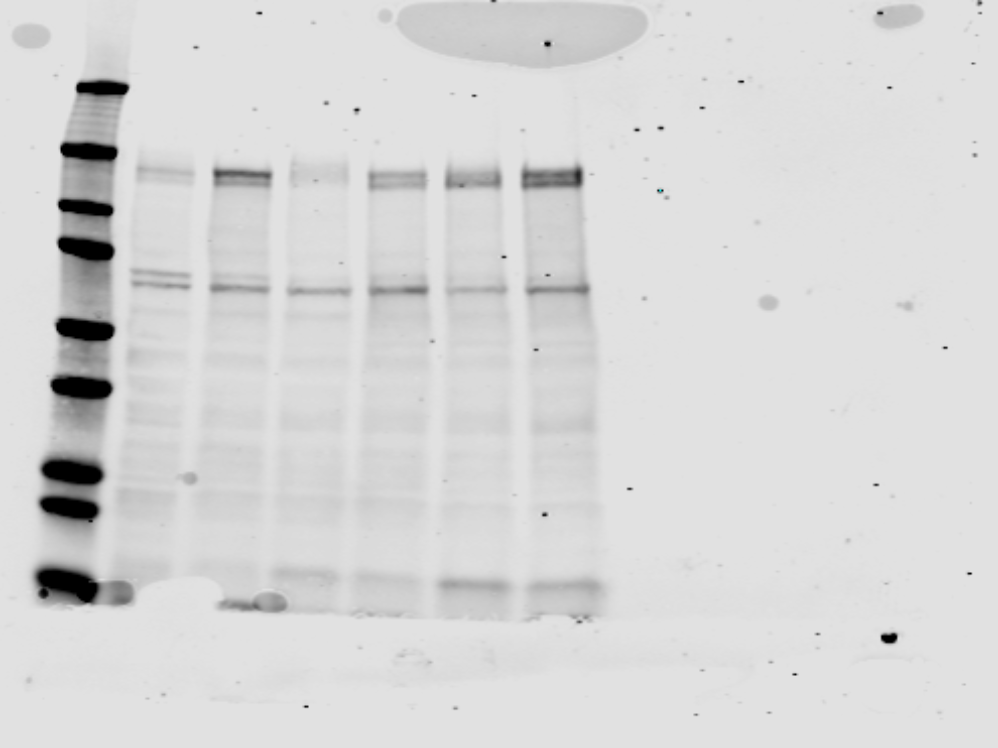

Supplement: Supplementary file 5 — Source data Fig. 3 [file 44318_2024_319_MOESM5_ESM.zip › EMBOJ-2024-117498-T-SourceDataForFigure3A-J/Figure 3 B/HT29_biological replicate n1/HT29_PERK_Western.tif.tif]

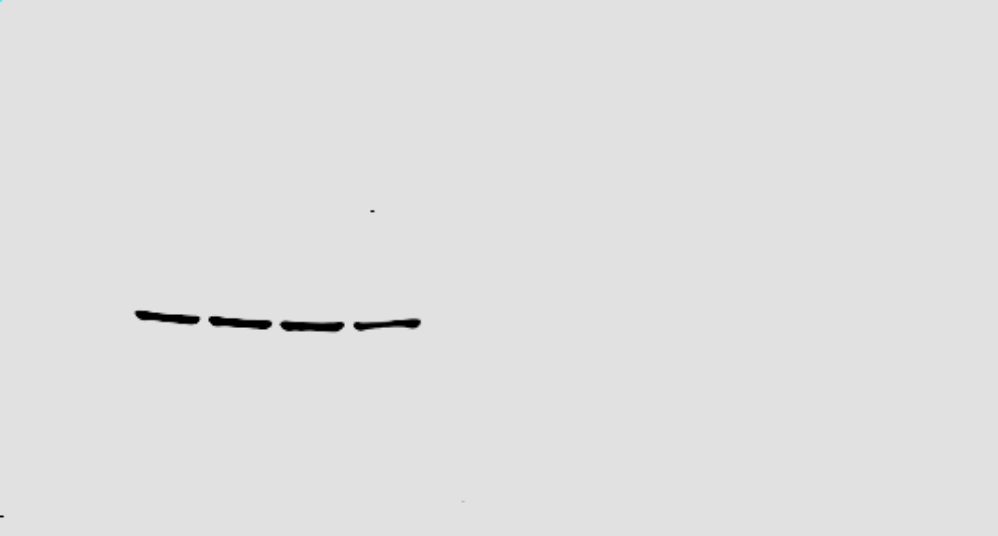

Supplement: Supplementary file 5 — Source data Fig. 3 [file 44318_2024_319_MOESM5_ESM.zip › EMBOJ-2024-117498-T-SourceDataForFigure3A-J/Figure 3 B/HT29_biological replicate n2/HT29_actin_western.tif]

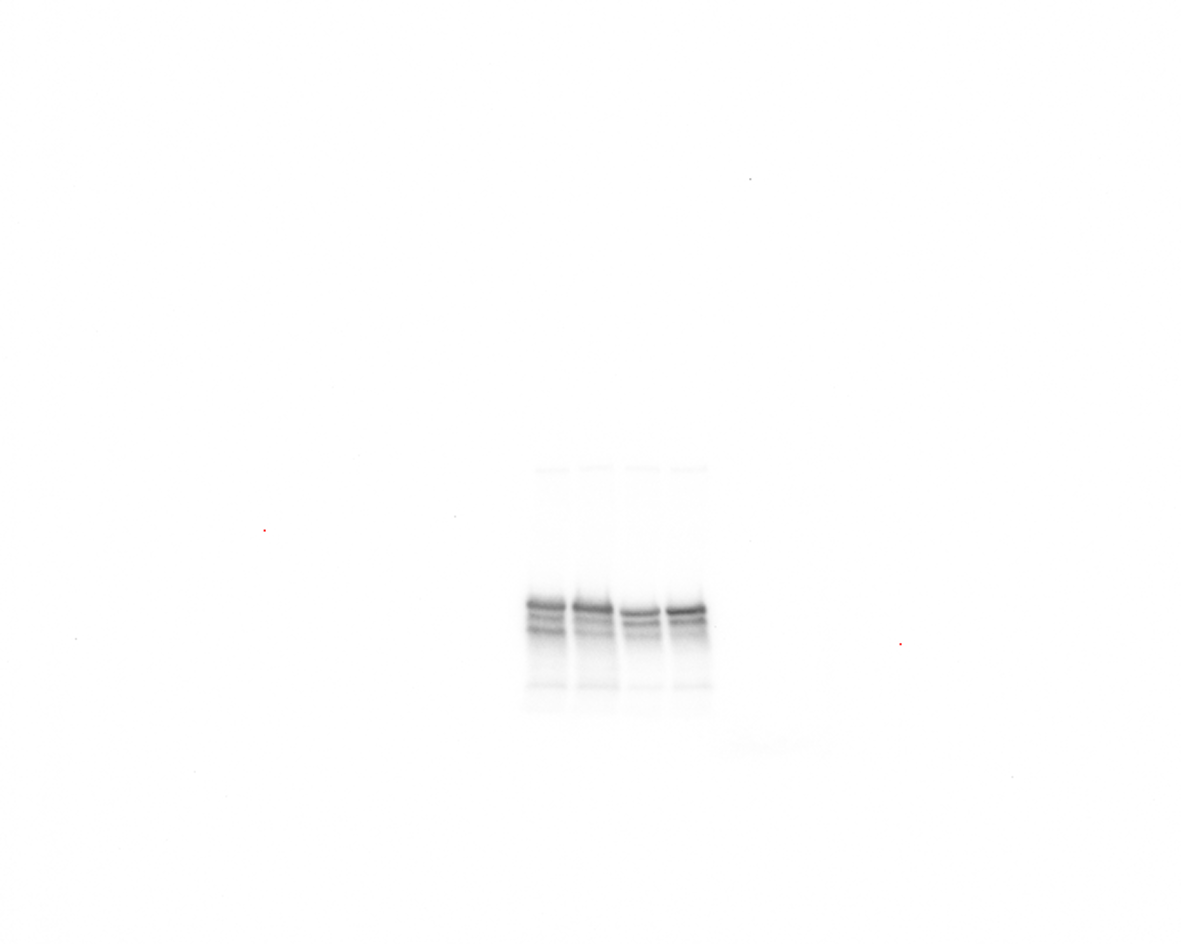

Supplement: Supplementary file 5 — Source data Fig. 3 [file 44318_2024_319_MOESM5_ESM.zip › EMBOJ-2024-117498-T-SourceDataForFigure3A-J/Figure 3 B/HT29_biological replicate n2/HT29_MHC I_western.tif]

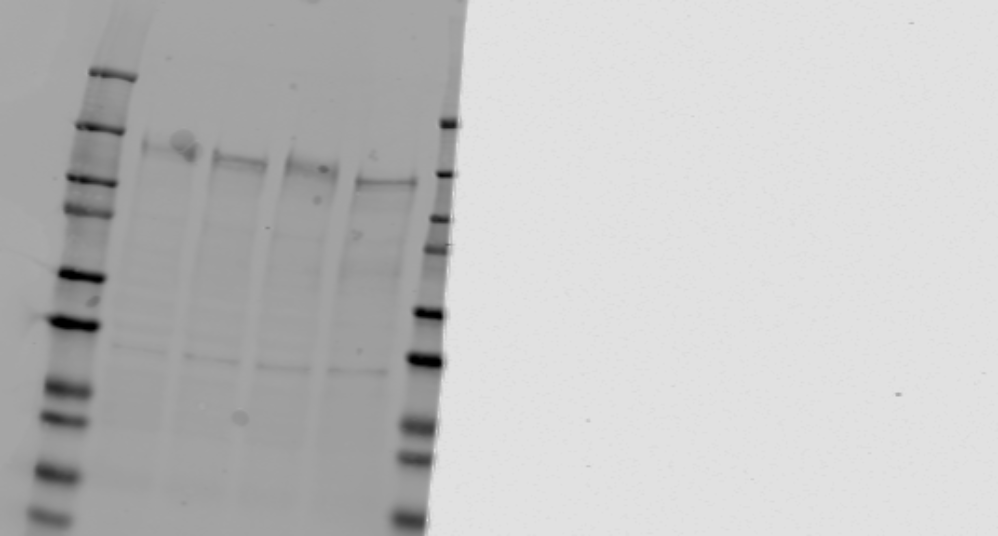

Supplement: Supplementary file 5 — Source data Fig. 3 [file 44318_2024_319_MOESM5_ESM.zip › EMBOJ-2024-117498-T-SourceDataForFigure3A-J/Figure 3 B/HT29_biological replicate n2/HT29_PERK_western.tif]

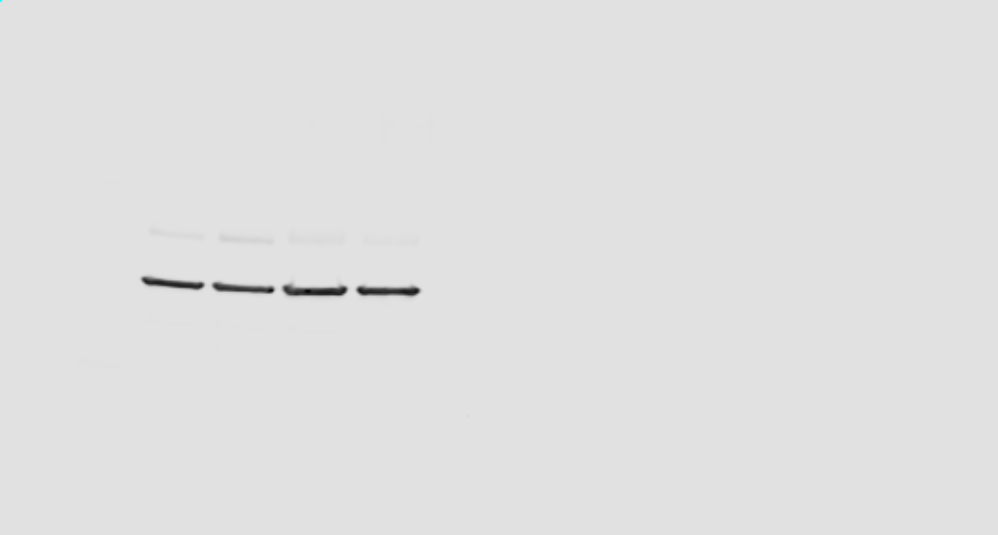

Supplement: Supplementary file 5 — Source data Fig. 3 [file 44318_2024_319_MOESM5_ESM.zip › EMBOJ-2024-117498-T-SourceDataForFigure3A-J/Figure 3 B/HT29_biological replicate n3/HT29_Actin_western.tif]

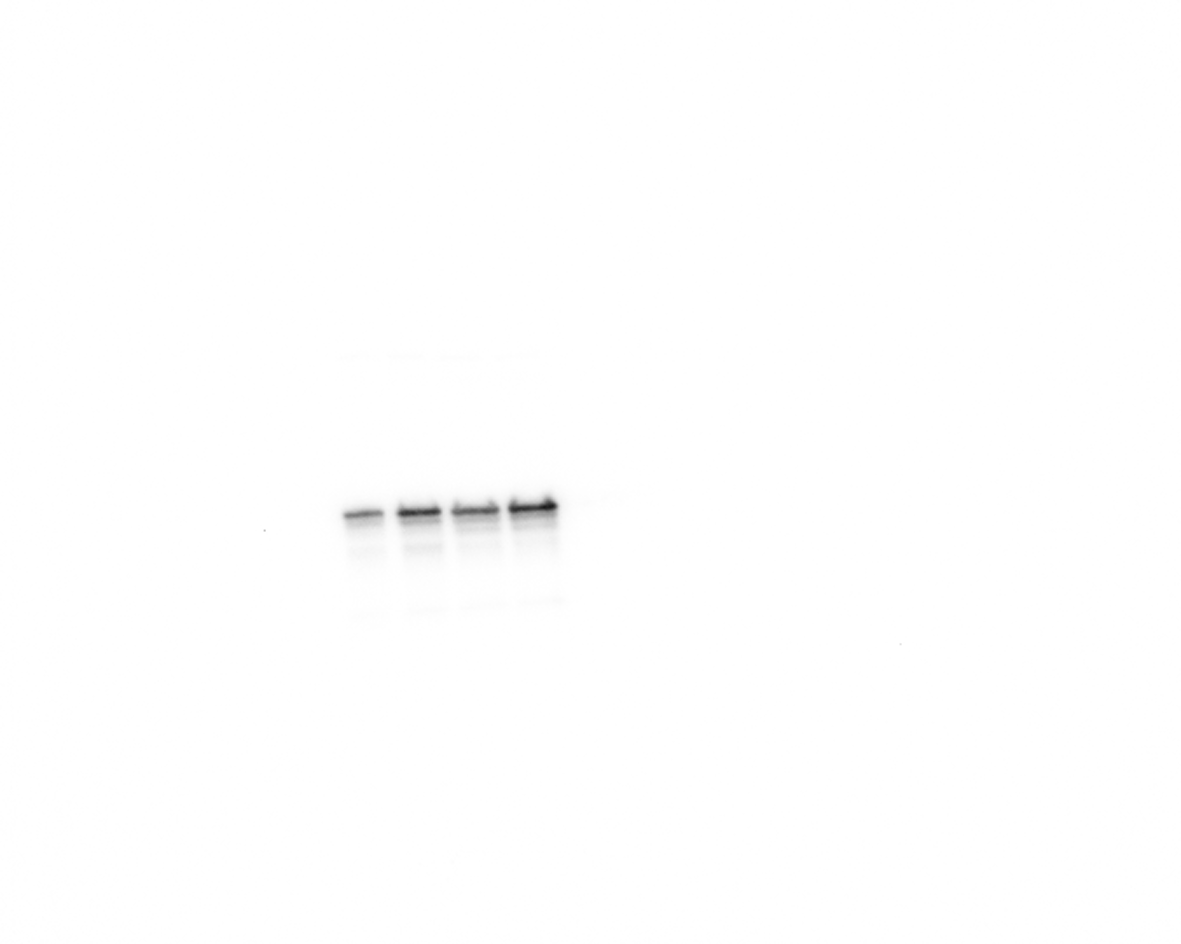

Supplement: Supplementary file 5 — Source data Fig. 3 [file 44318_2024_319_MOESM5_ESM.zip › EMBOJ-2024-117498-T-SourceDataForFigure3A-J/Figure 3 B/HT29_biological replicate n3/HT29_MHC I_western.tif]

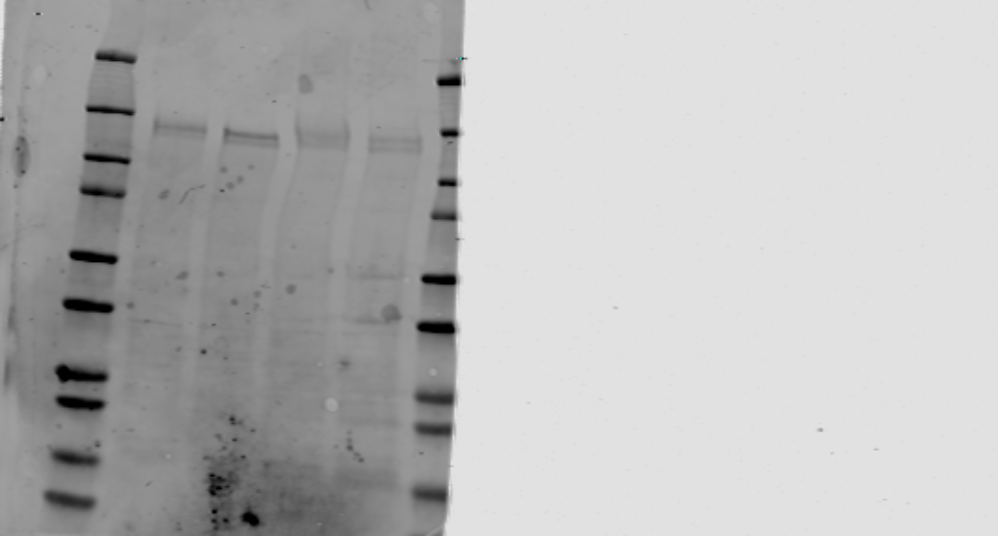

Supplement: Supplementary file 5 — Source data Fig. 3 [file 44318_2024_319_MOESM5_ESM.zip › EMBOJ-2024-117498-T-SourceDataForFigure3A-J/Figure 3 B/HT29_biological replicate n3/HT29_PERK_western.tif.tif]

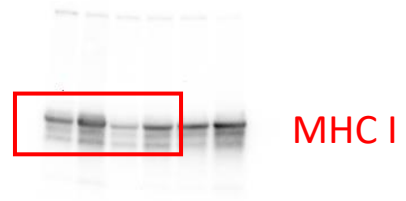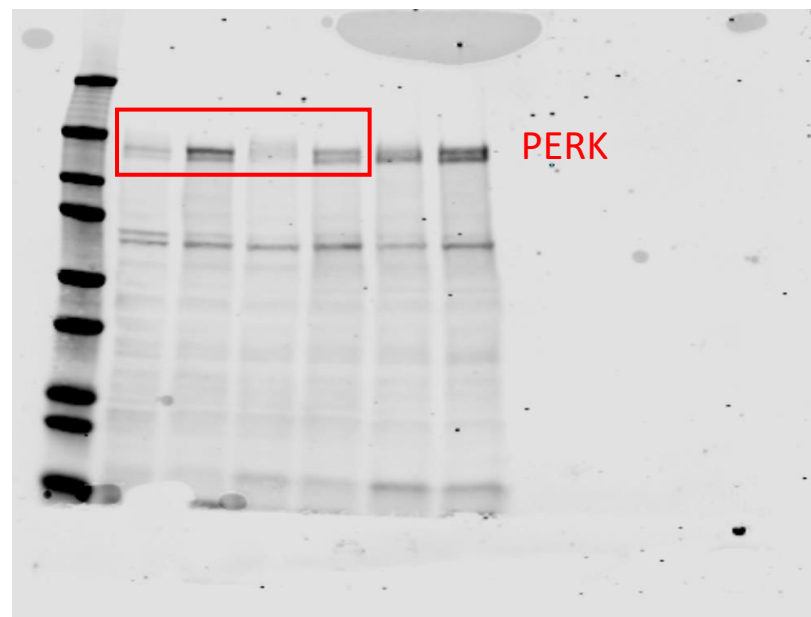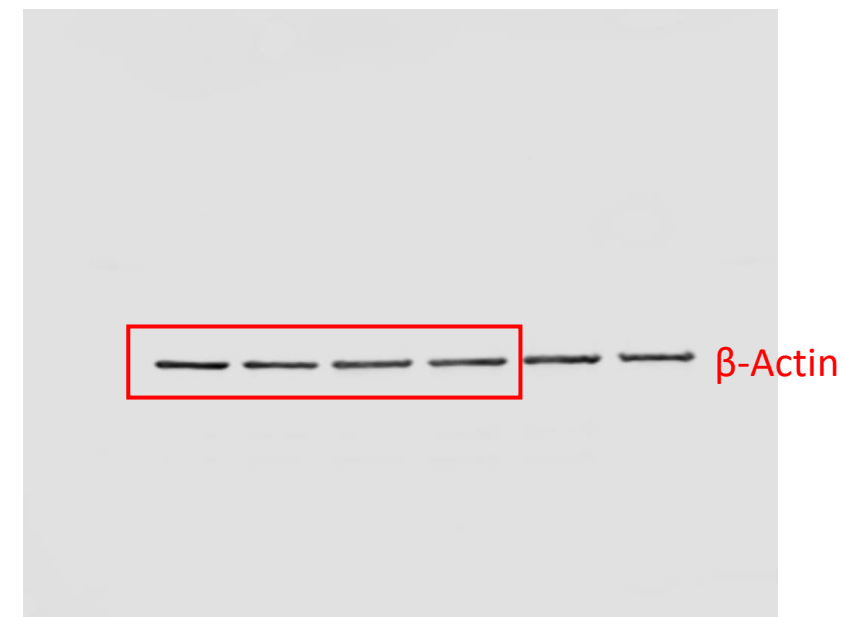

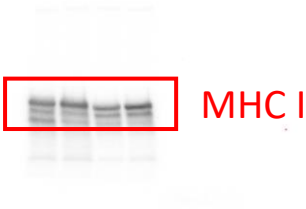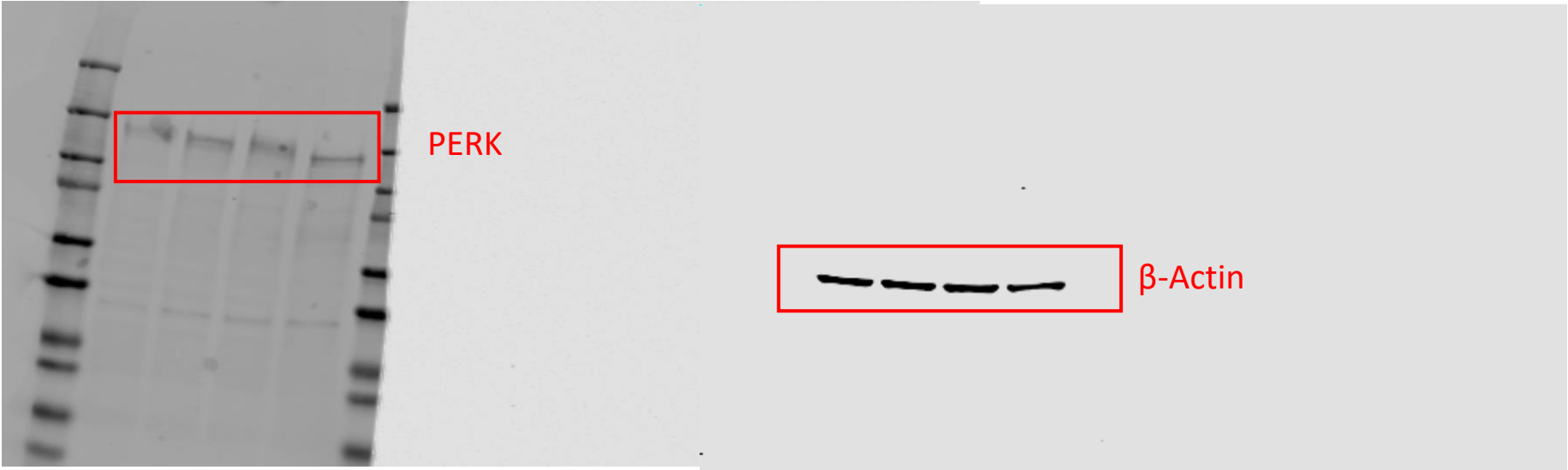

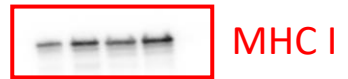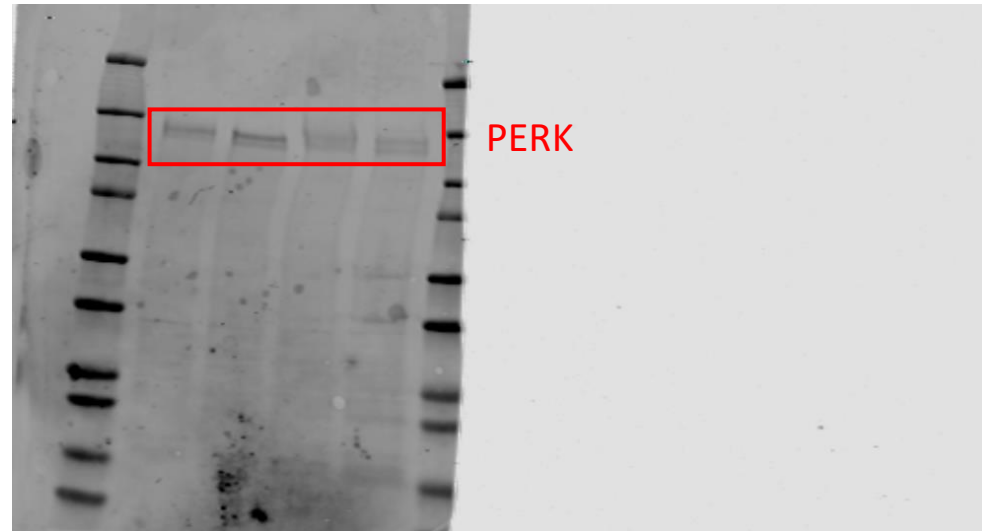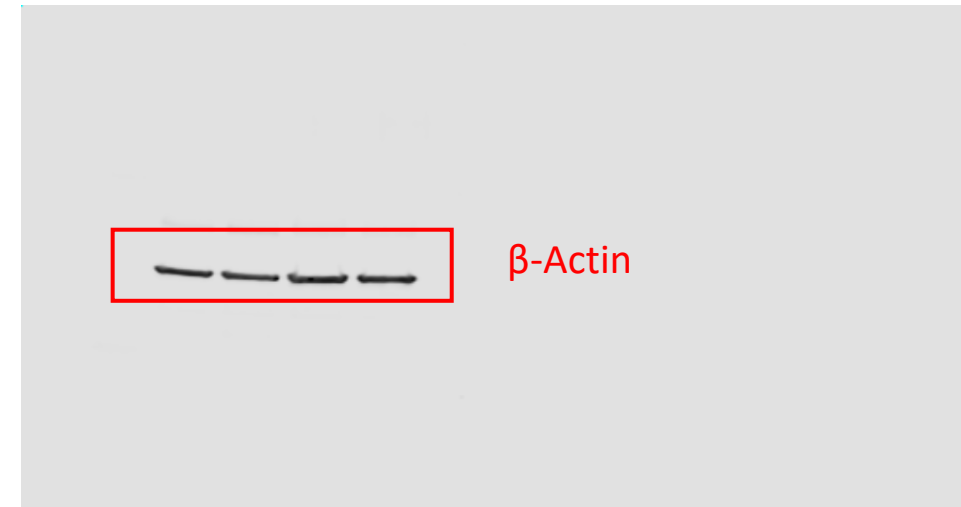

Supplement: Supplementary file 5 — Source data Fig. 3 [file 44318_2024_319_MOESM5_ESM.zip › EMBOJ-2024-117498-T-SourceDataForFigure3A-J/Figure 3 B/README/HT29_ biological replicates_western.pdf]

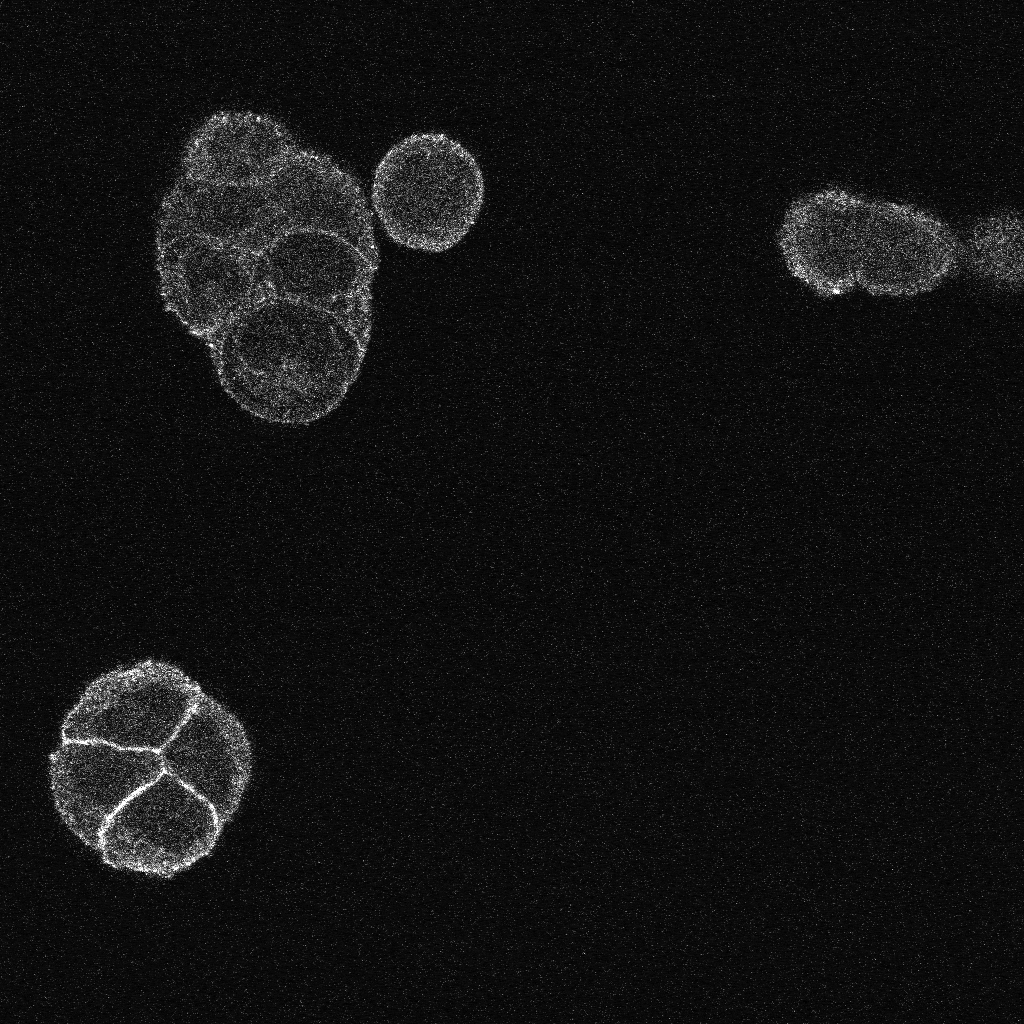

Supplement: Supplementary file 5 — Source data Fig. 3 [file 44318_2024_319_MOESM5_ESM.zip › EMBOJ-2024-117498-T-SourceDataForFigure3A-J/Figure 3 D/Hypoxia veh 0% 24h HT29 MHC I staining .tif]

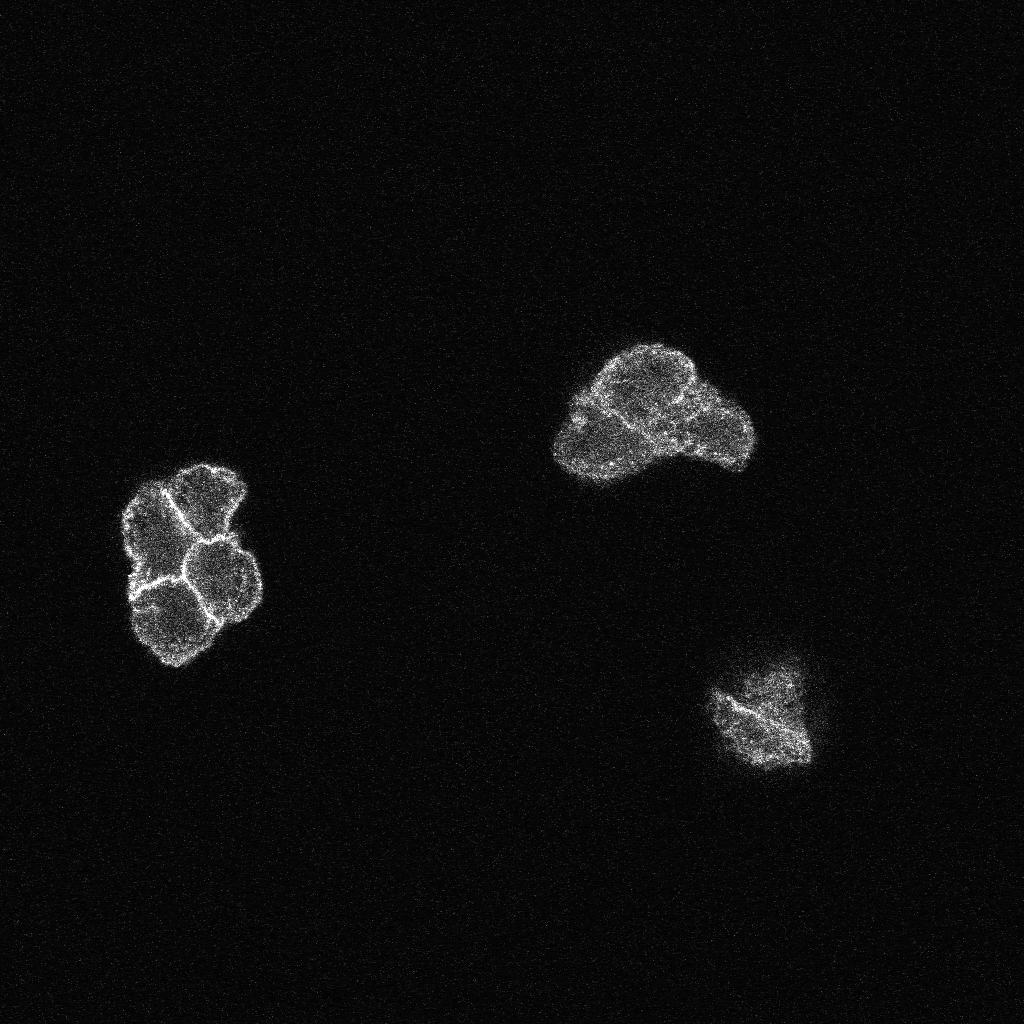

Supplement: Supplementary file 5 — Source data Fig. 3 [file 44318_2024_319_MOESM5_ESM.zip › EMBOJ-2024-117498-T-SourceDataForFigure3A-J/Figure 3 D/Hypoxia veh 0% 24h PERKi HT29 MHC I staining .tif]

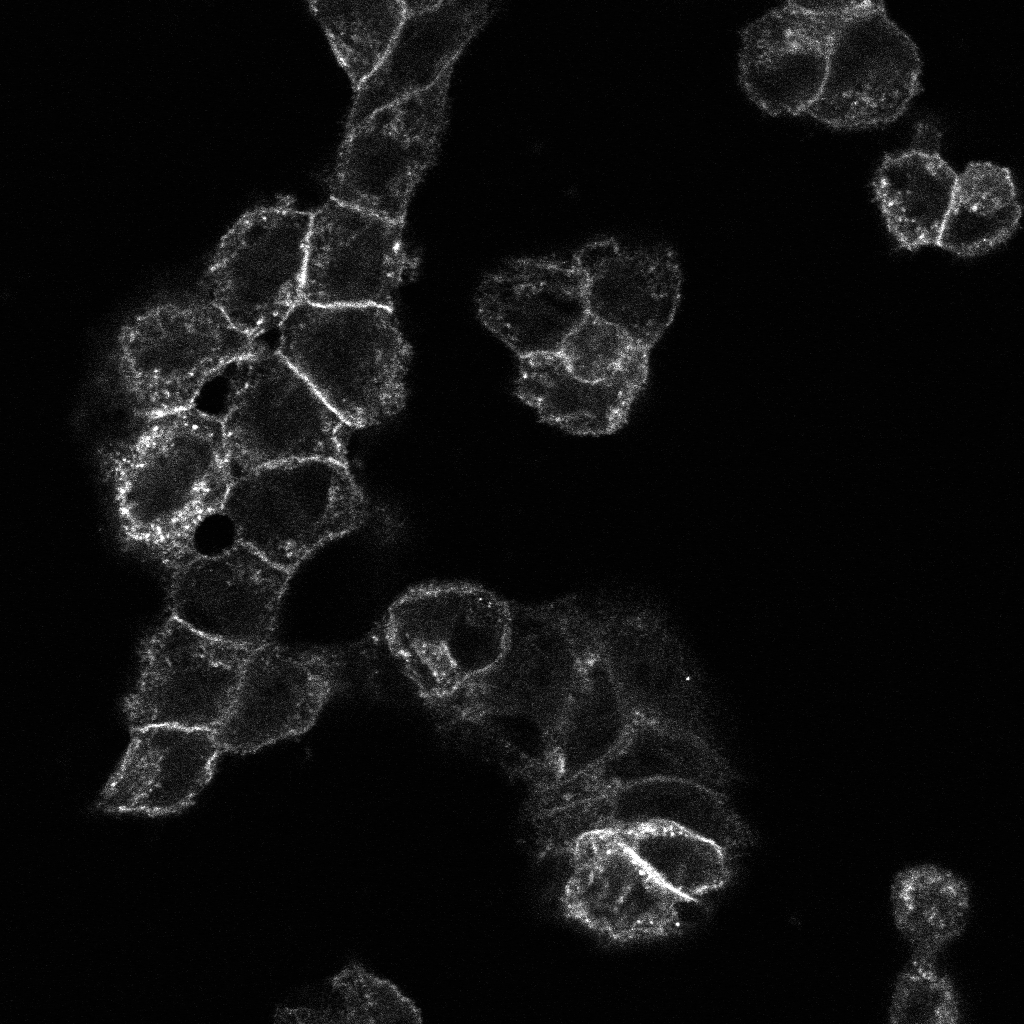

Supplement: Supplementary file 5 — Source data Fig. 3 [file 44318_2024_319_MOESM5_ESM.zip › EMBOJ-2024-117498-T-SourceDataForFigure3A-J/Figure 3 D/normoxia PERKi HT29 MHC I staining.tif]

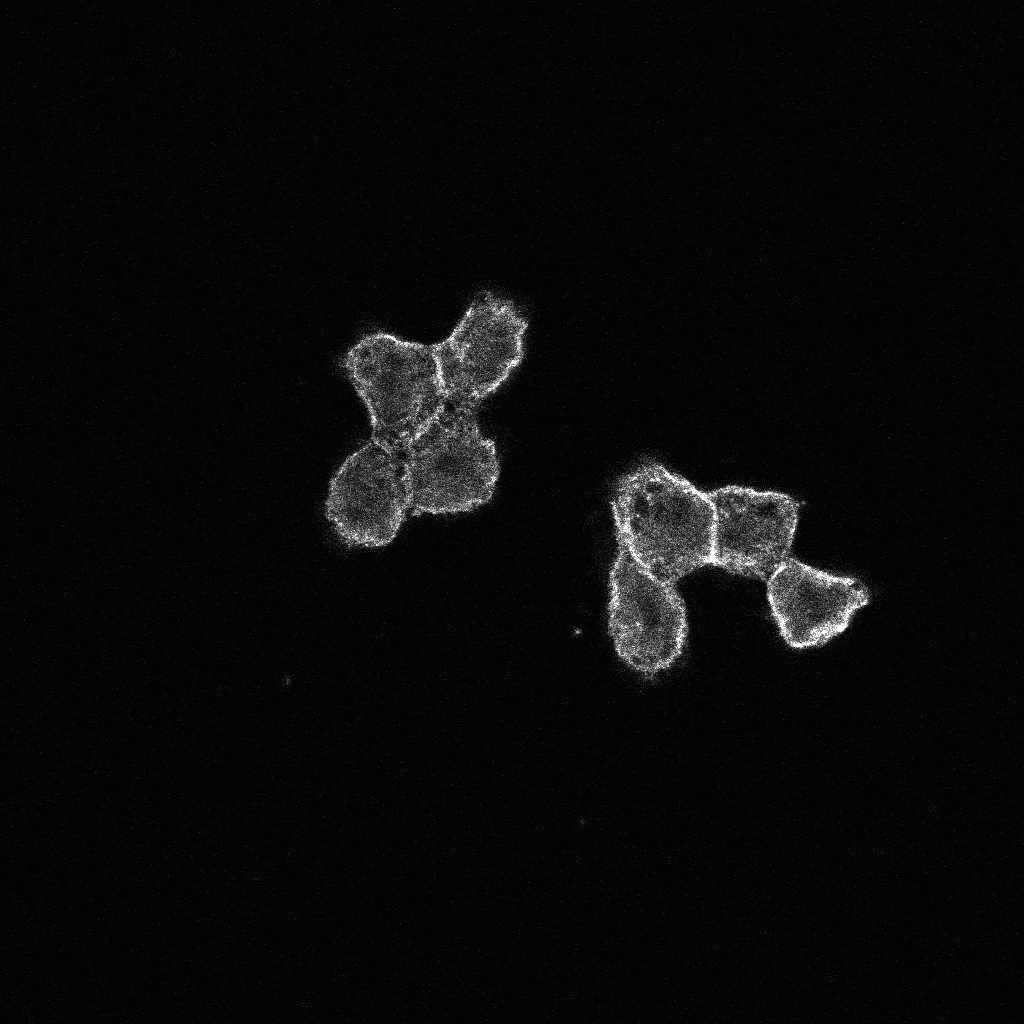

Supplement: Supplementary file 5 — Source data Fig. 3 [file 44318_2024_319_MOESM5_ESM.zip › EMBOJ-2024-117498-T-SourceDataForFigure3A-J/Figure 3 D/normoxia veh HT29 MHC I staining.tif]

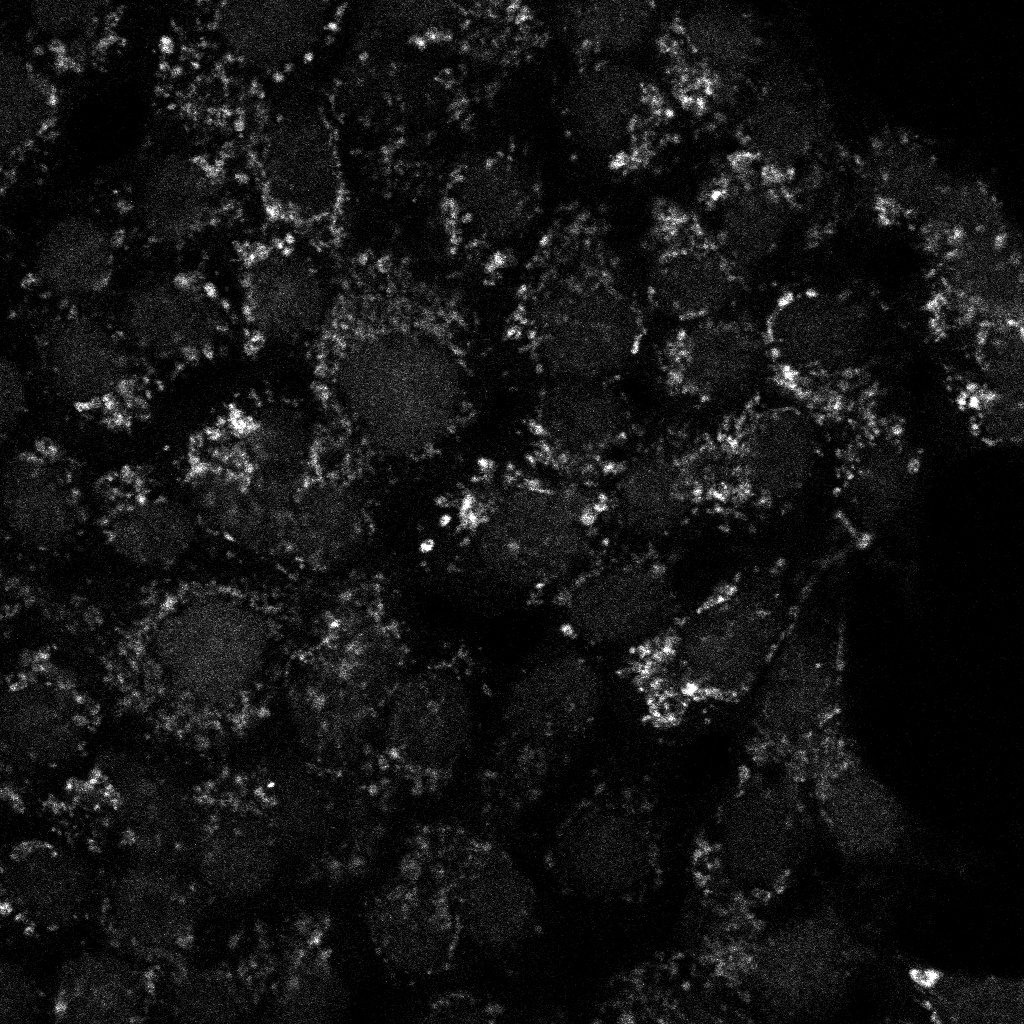

Supplement: Supplementary file 5 — Source data Fig. 3 [file 44318_2024_319_MOESM5_ESM.zip › EMBOJ-2024-117498-T-SourceDataForFigure3A-J/Figure 3 H/HT29 SUSD6 staining representative image hypoxia 0% 16h time point .tif]

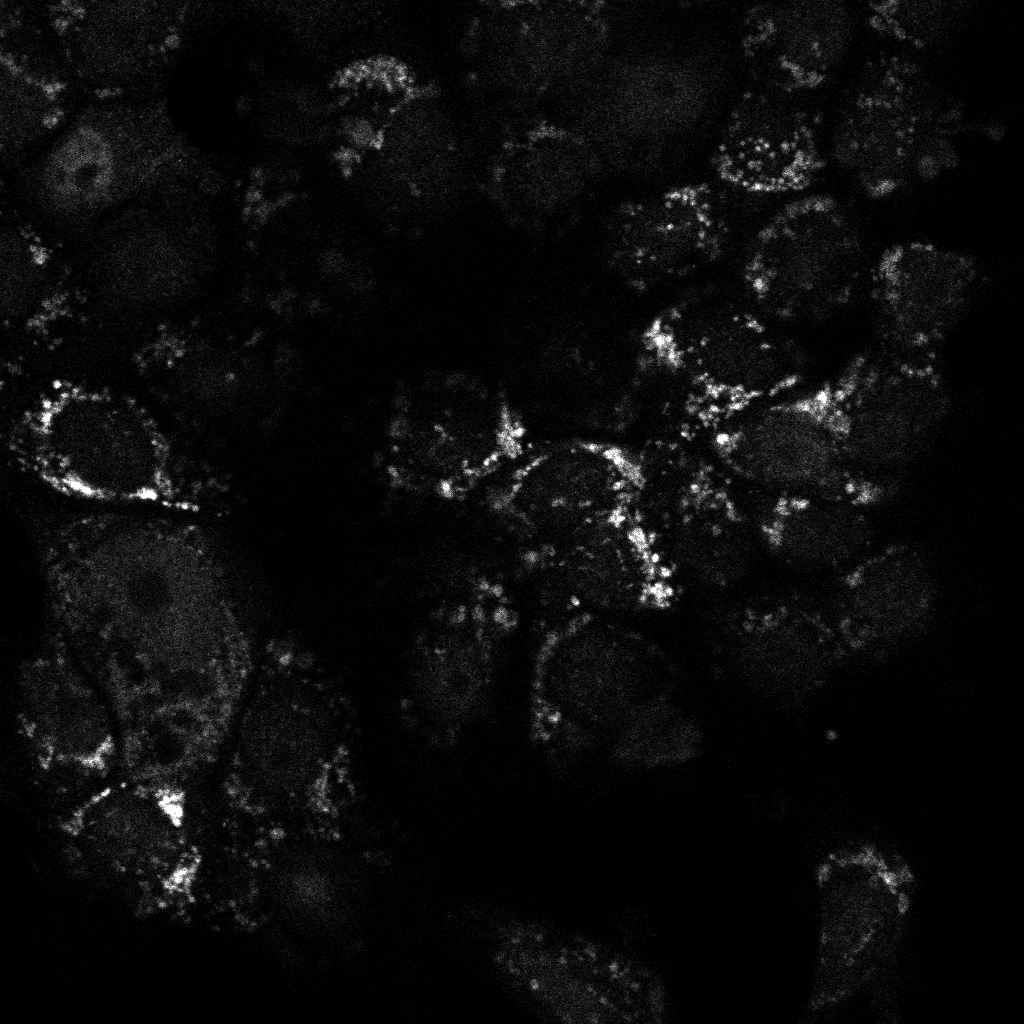

Supplement: Supplementary file 5 — Source data Fig. 3 [file 44318_2024_319_MOESM5_ESM.zip › EMBOJ-2024-117498-T-SourceDataForFigure3A-J/Figure 3 H/HT29 SUSD6 staining representative image hypoxia 0% 24h time point .tif]

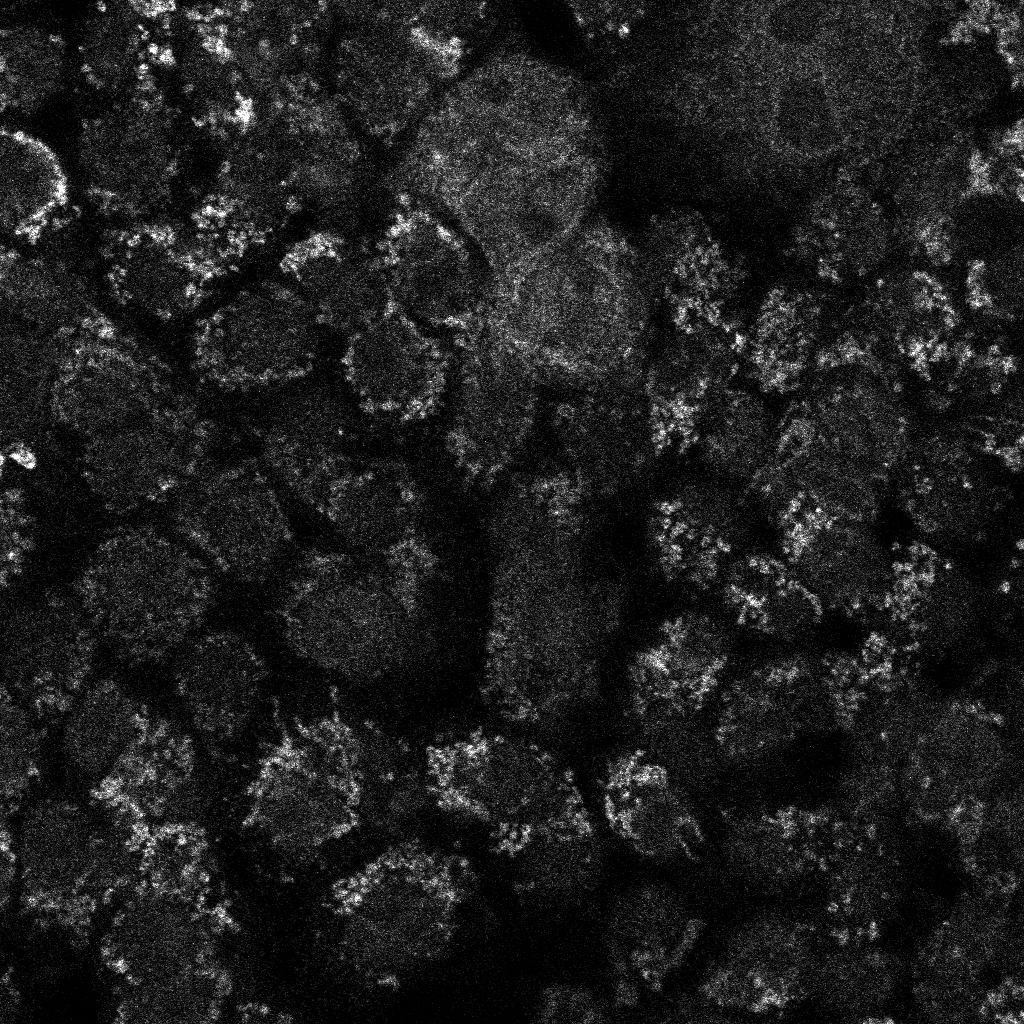

Supplement: Supplementary file 5 — Source data Fig. 3 [file 44318_2024_319_MOESM5_ESM.zip › EMBOJ-2024-117498-T-SourceDataForFigure3A-J/Figure 3 H/HT29 SUSD6 staining representative image normoxia.tif]

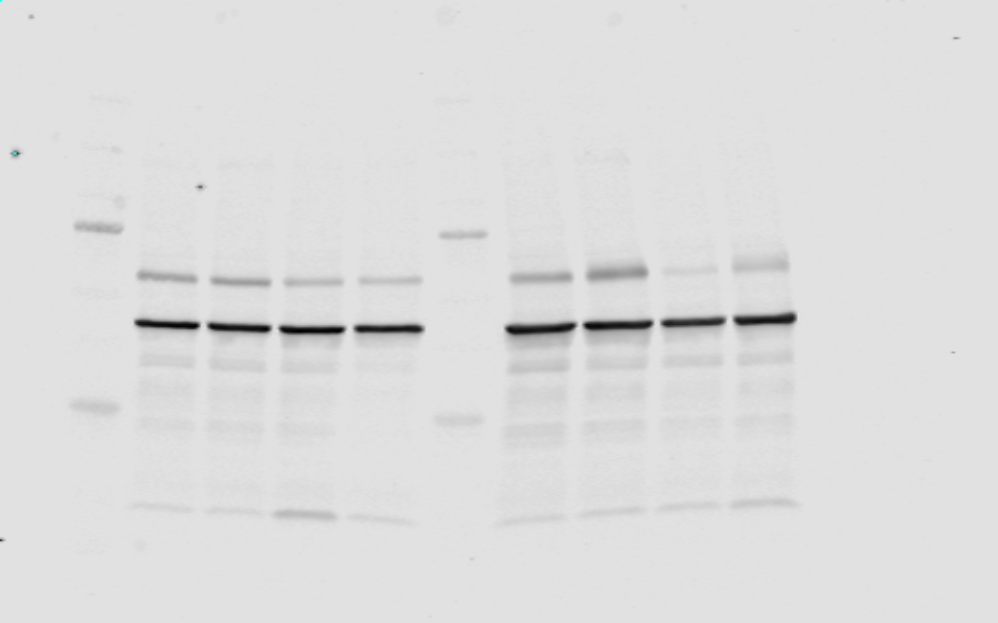

Supplement: Supplementary file 6 — Source data Fig. 4 [file 44318_2024_319_MOESM6_ESM.zip › EMBOJ-2024-117498-T-SourceDataForFigure4A-H/Figure 4 A/HT29_Biological replicate n1/HT29_Actin_Western.tif.tif]

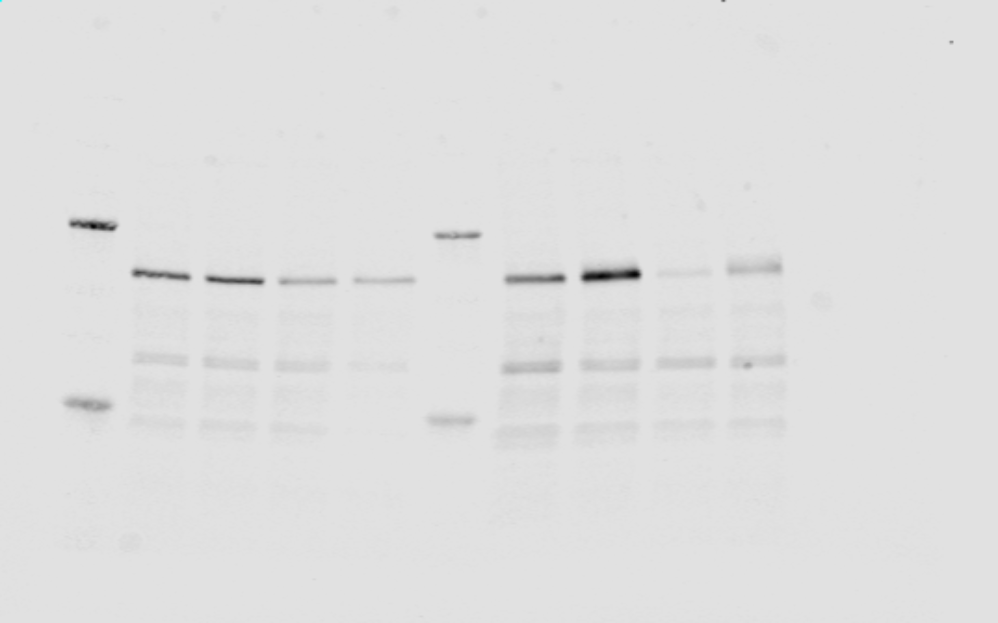

Supplement: Supplementary file 6 — Source data Fig. 4 [file 44318_2024_319_MOESM6_ESM.zip › EMBOJ-2024-117498-T-SourceDataForFigure4A-H/Figure 4 A/HT29_Biological replicate n1/HT29_p62_Western.tif]

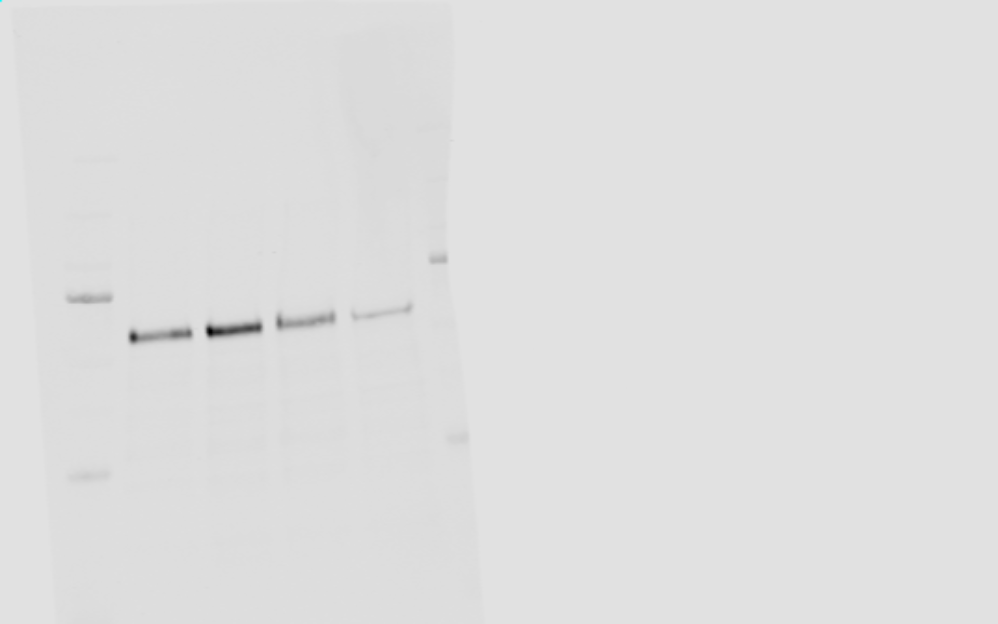

Supplement: Supplementary file 6 — Source data Fig. 4 [file 44318_2024_319_MOESM6_ESM.zip › EMBOJ-2024-117498-T-SourceDataForFigure4A-H/Figure 4 A/HT29_Biological replicate n2/HT29_p62_Western.tif.tif]

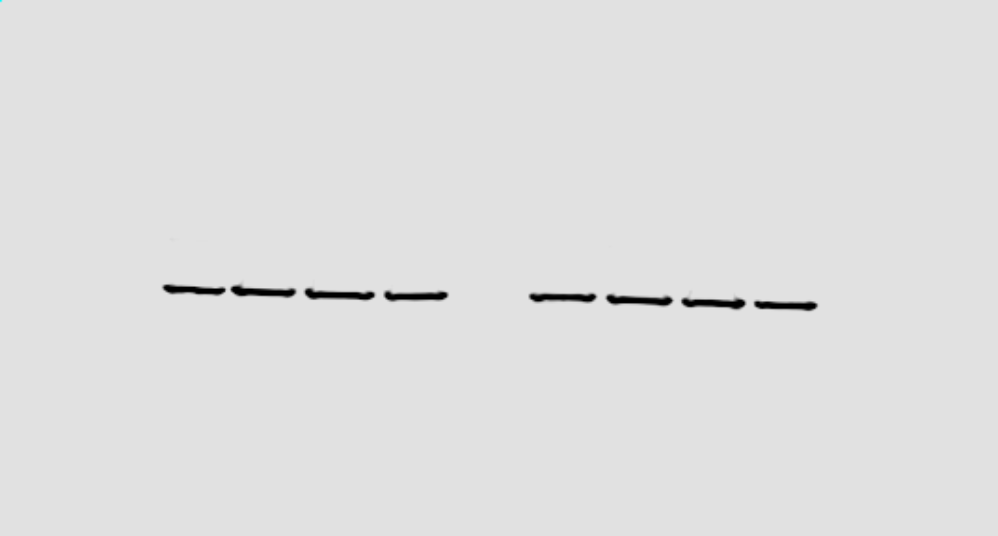

Supplement: Supplementary file 6 — Source data Fig. 4 [file 44318_2024_319_MOESM6_ESM.zip › EMBOJ-2024-117498-T-SourceDataForFigure4A-H/Figure 4 A/HT29_Biological replicate n3/HT29_actin_western.tif.tif]

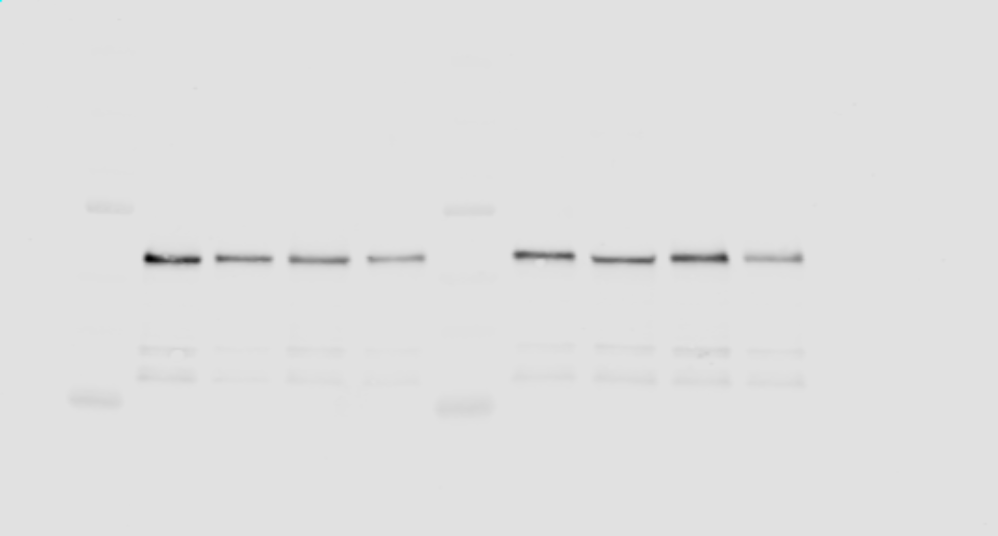

Supplement: Supplementary file 6 — Source data Fig. 4 [file 44318_2024_319_MOESM6_ESM.zip › EMBOJ-2024-117498-T-SourceDataForFigure4A-H/Figure 4 A/HT29_Biological replicate n3/HT29_p62_western.tif]

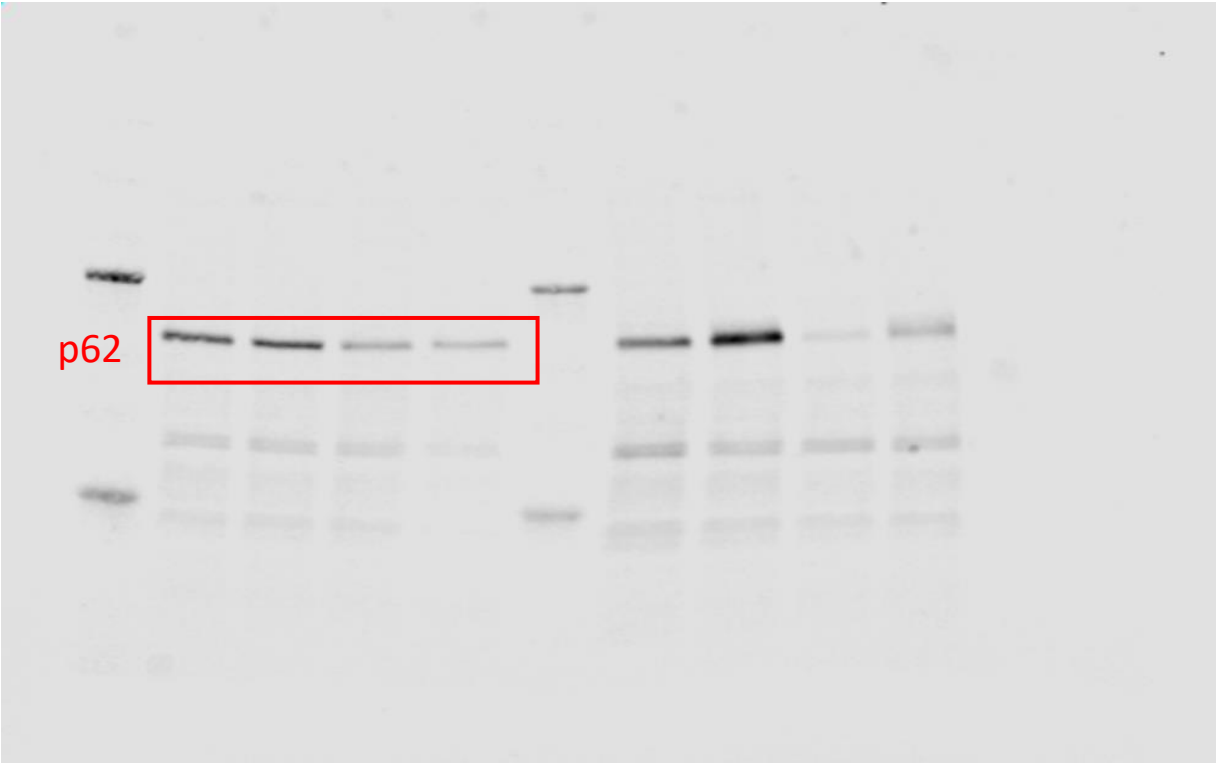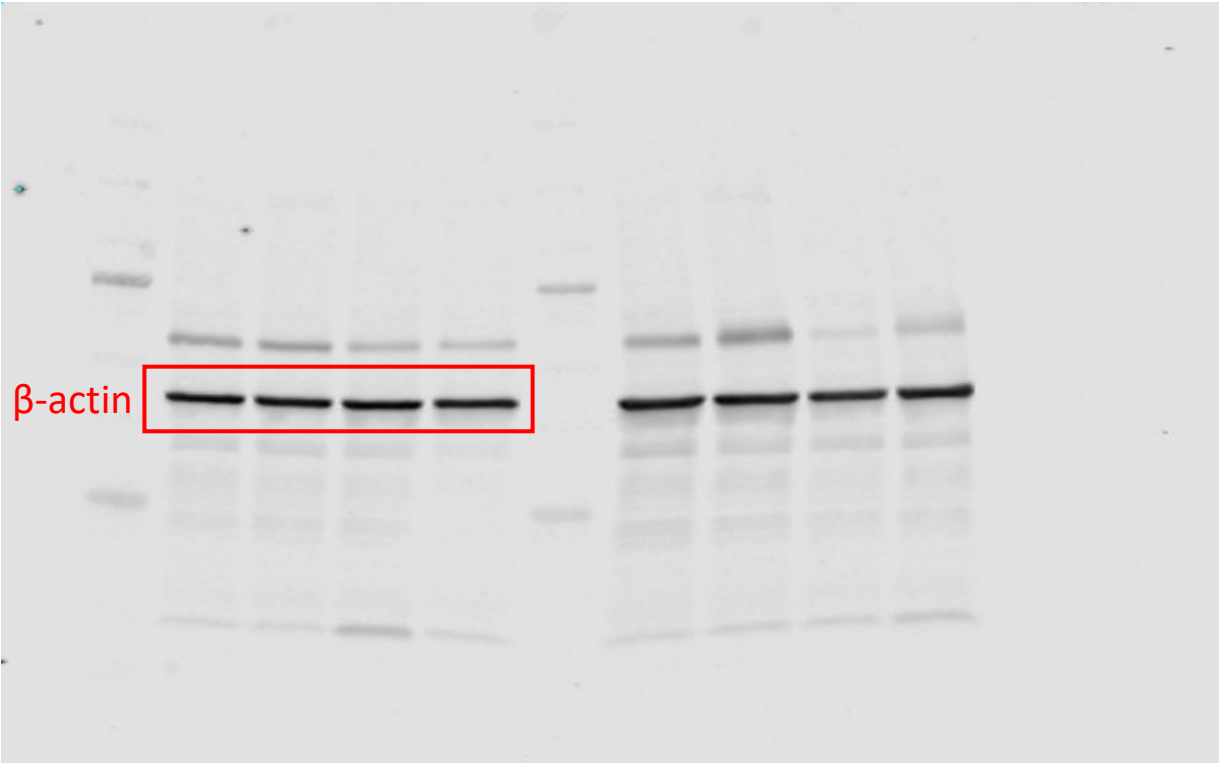

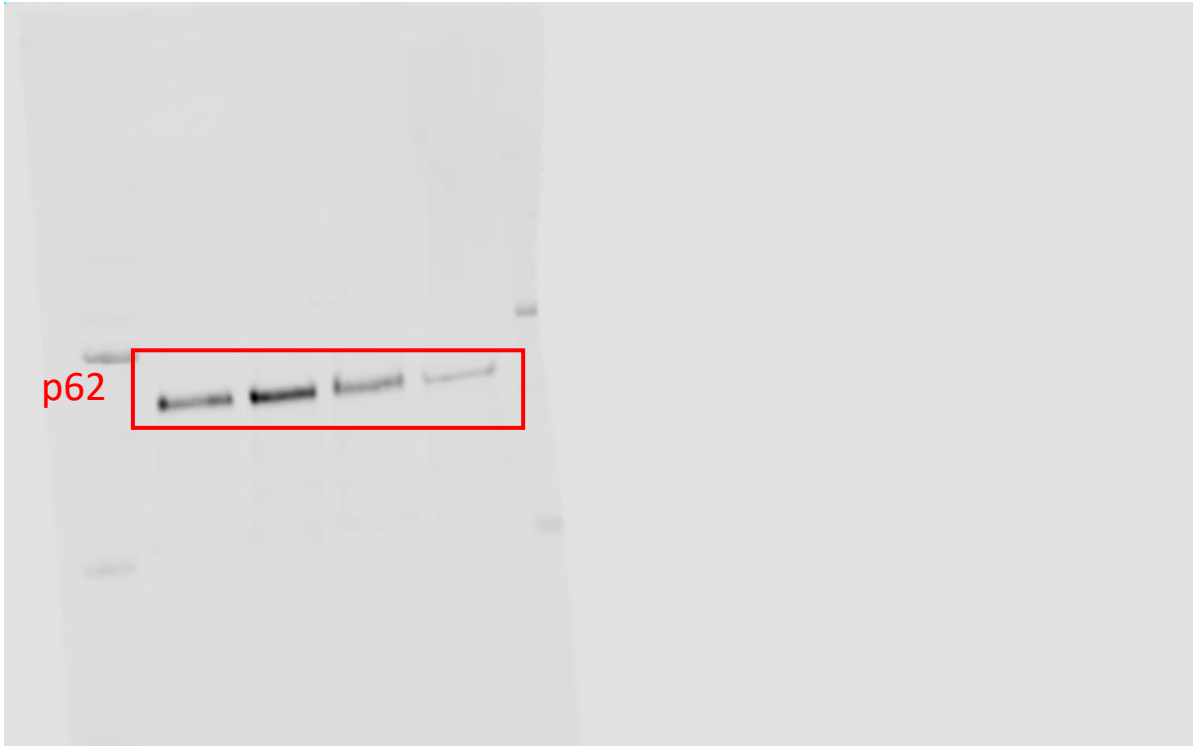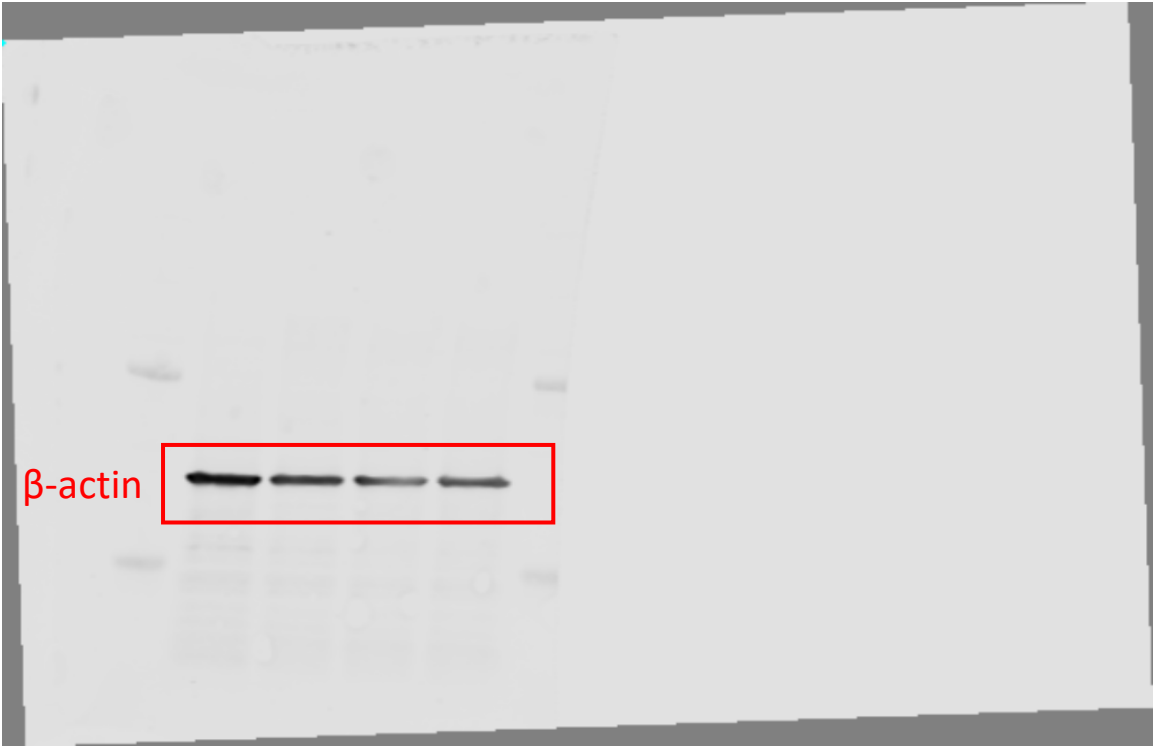

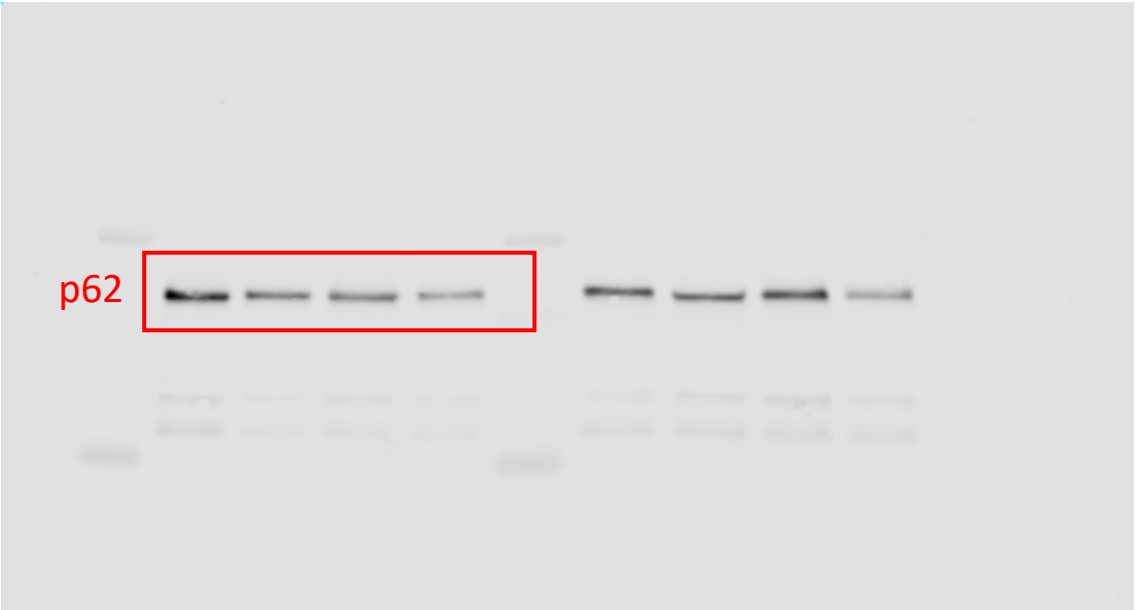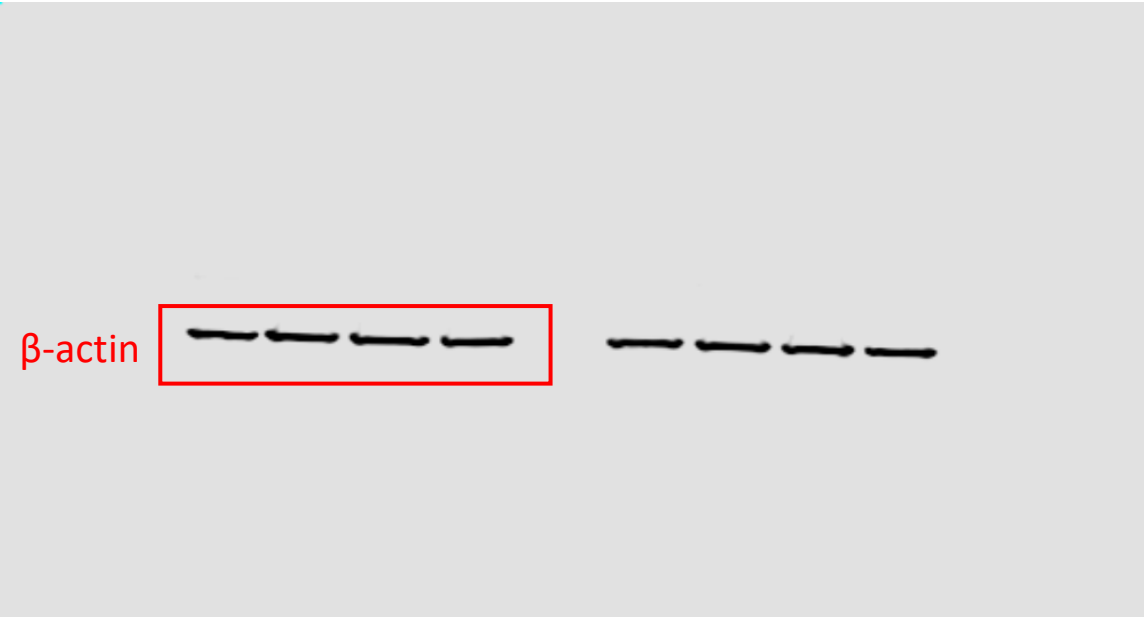

Supplement: Supplementary file 6 — Source data Fig. 4 [file 44318_2024_319_MOESM6_ESM.zip › EMBOJ-2024-117498-T-SourceDataForFigure4A-H/Figure 4 A/README/HT29_western_Biological replicates .pdf]

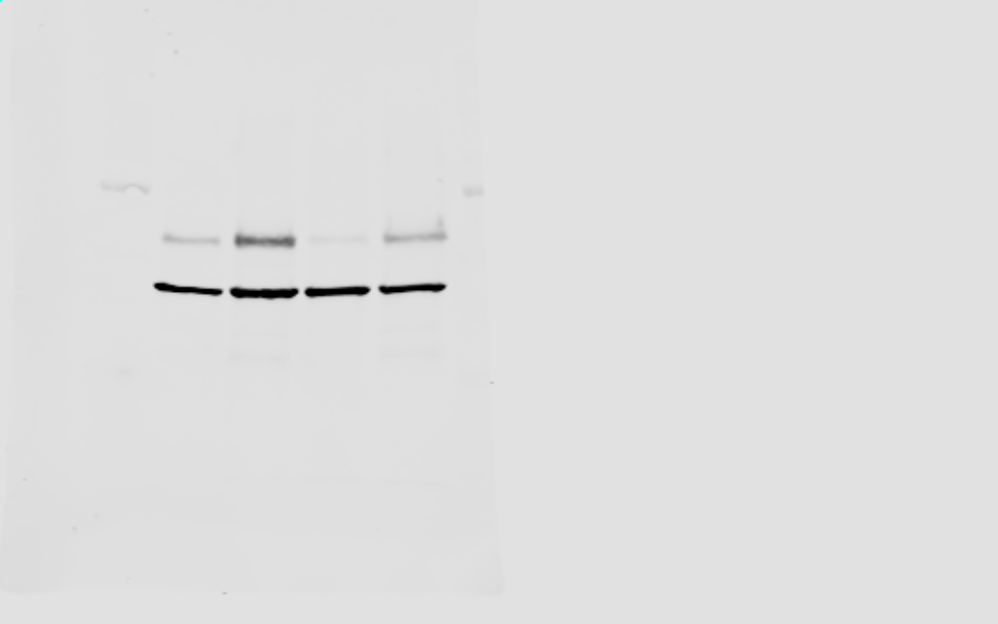

Supplement: Supplementary file 6 — Source data Fig. 4 [file 44318_2024_319_MOESM6_ESM.zip › EMBOJ-2024-117498-T-SourceDataForFigure4A-H/Figure 4 B/HT29_Biological replicate n1/HT29_Actin-p62_Western.tif.tif]

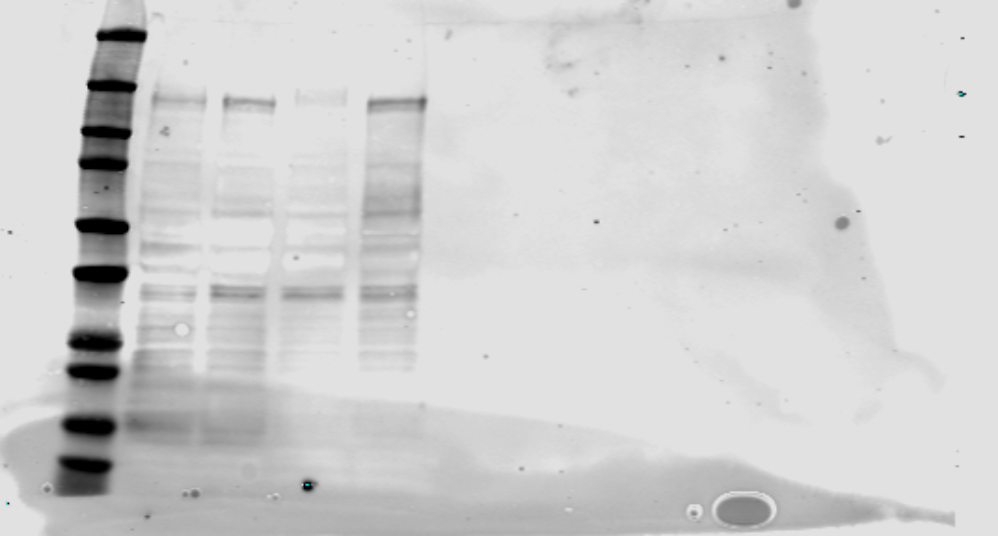

Supplement: Supplementary file 6 — Source data Fig. 4 [file 44318_2024_319_MOESM6_ESM.zip › EMBOJ-2024-117498-T-SourceDataForFigure4A-H/Figure 4 B/HT29_Biological replicate n1/HT29_PERK_western.tif.tif]

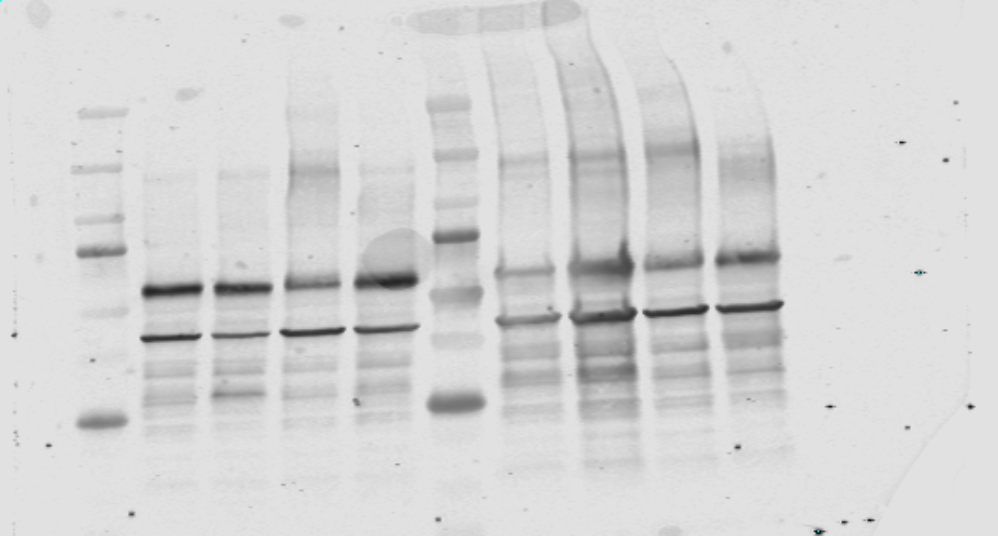

Supplement: Supplementary file 6 — Source data Fig. 4 [file 44318_2024_319_MOESM6_ESM.zip › EMBOJ-2024-117498-T-SourceDataForFigure4A-H/Figure 4 B/HT29_Biological replicate n2/HT29_actin_western.tif]

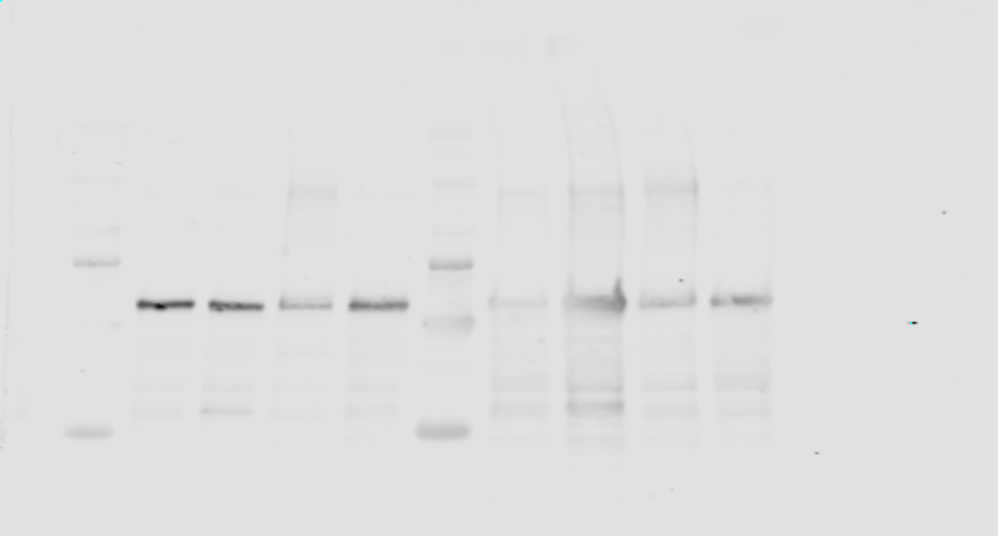

Supplement: Supplementary file 6 — Source data Fig. 4 [file 44318_2024_319_MOESM6_ESM.zip › EMBOJ-2024-117498-T-SourceDataForFigure4A-H/Figure 4 B/HT29_Biological replicate n2/HT29_p62_western.tif]

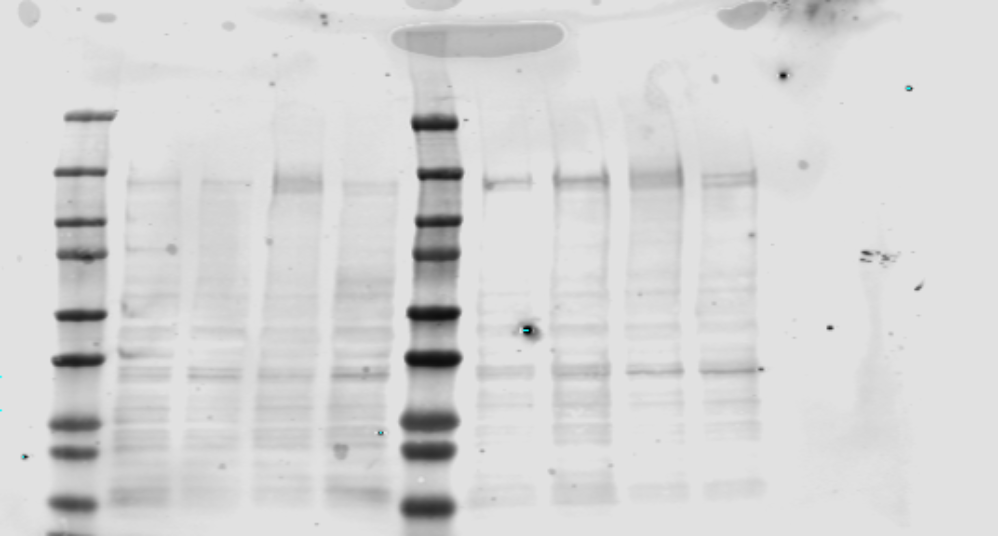

Supplement: Supplementary file 6 — Source data Fig. 4 [file 44318_2024_319_MOESM6_ESM.zip › EMBOJ-2024-117498-T-SourceDataForFigure4A-H/Figure 4 B/HT29_Biological replicate n2/HT29_PERK_western.tif.tif]

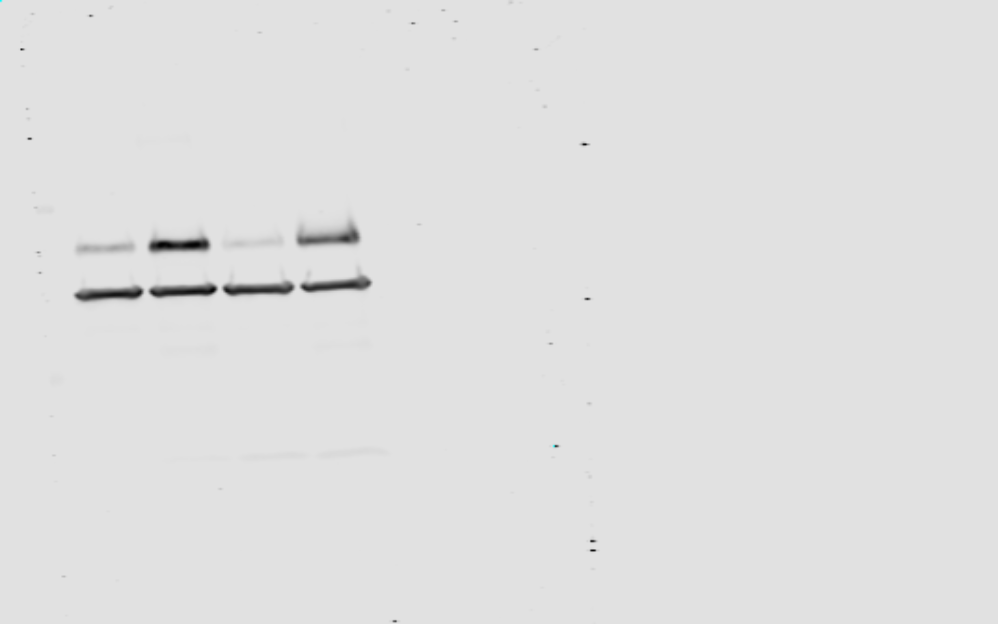

Supplement: Supplementary file 6 — Source data Fig. 4 [file 44318_2024_319_MOESM6_ESM.zip › EMBOJ-2024-117498-T-SourceDataForFigure4A-H/Figure 4 B/HT29_Biological replicate n3/HT29_Actin-p62_western.tif.tif]

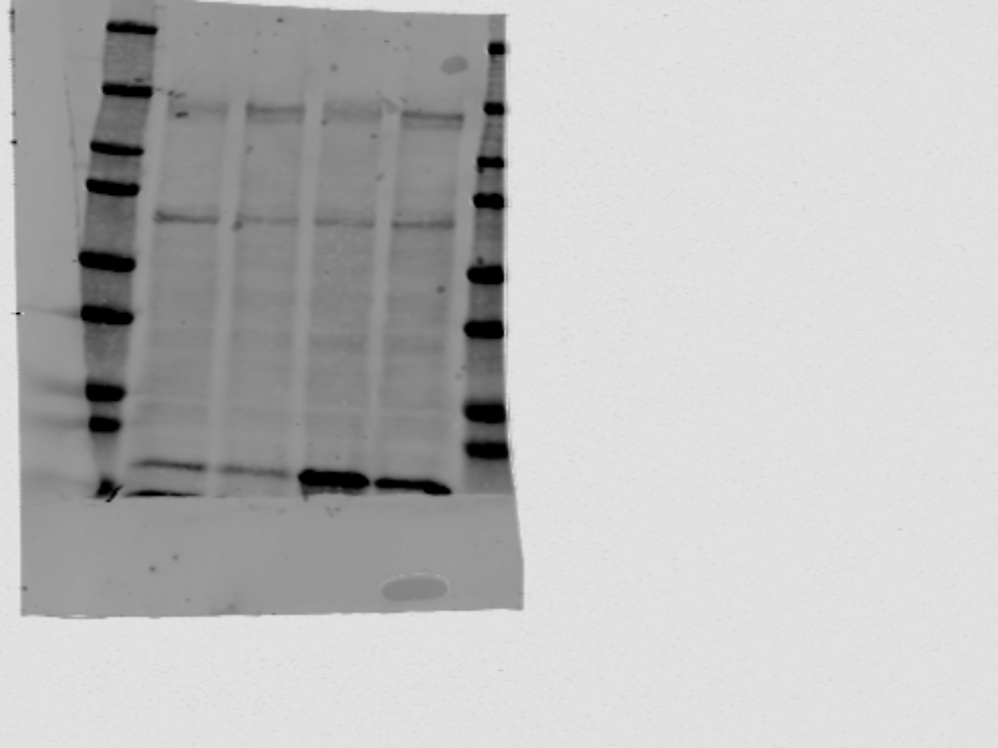

Supplement: Supplementary file 6 — Source data Fig. 4 [file 44318_2024_319_MOESM6_ESM.zip › EMBOJ-2024-117498-T-SourceDataForFigure4A-H/Figure 4 B/HT29_Biological replicate n3/HT29_PERK_Western.tif.tif]

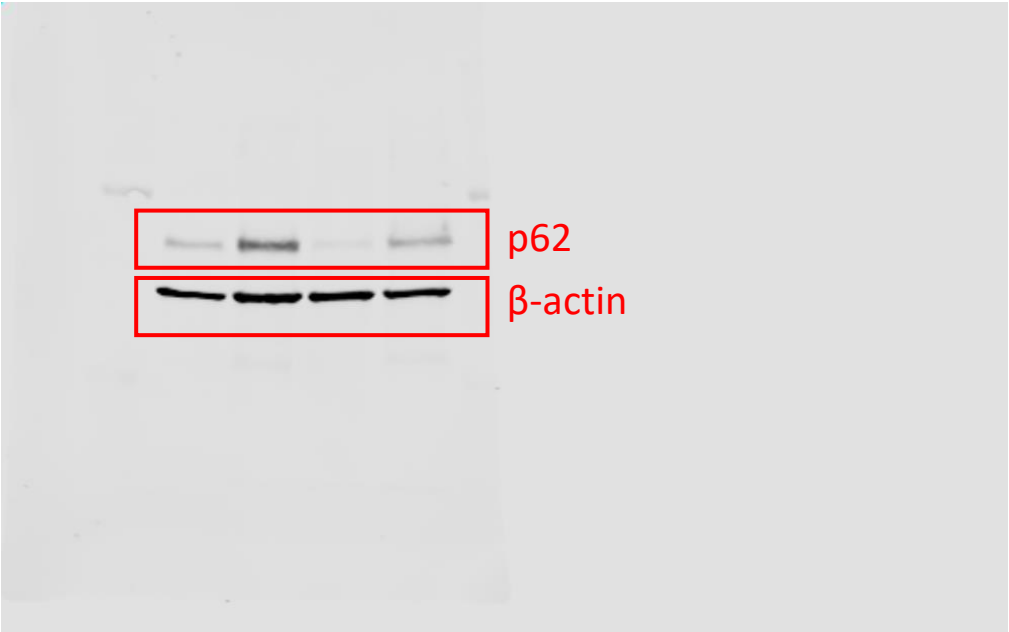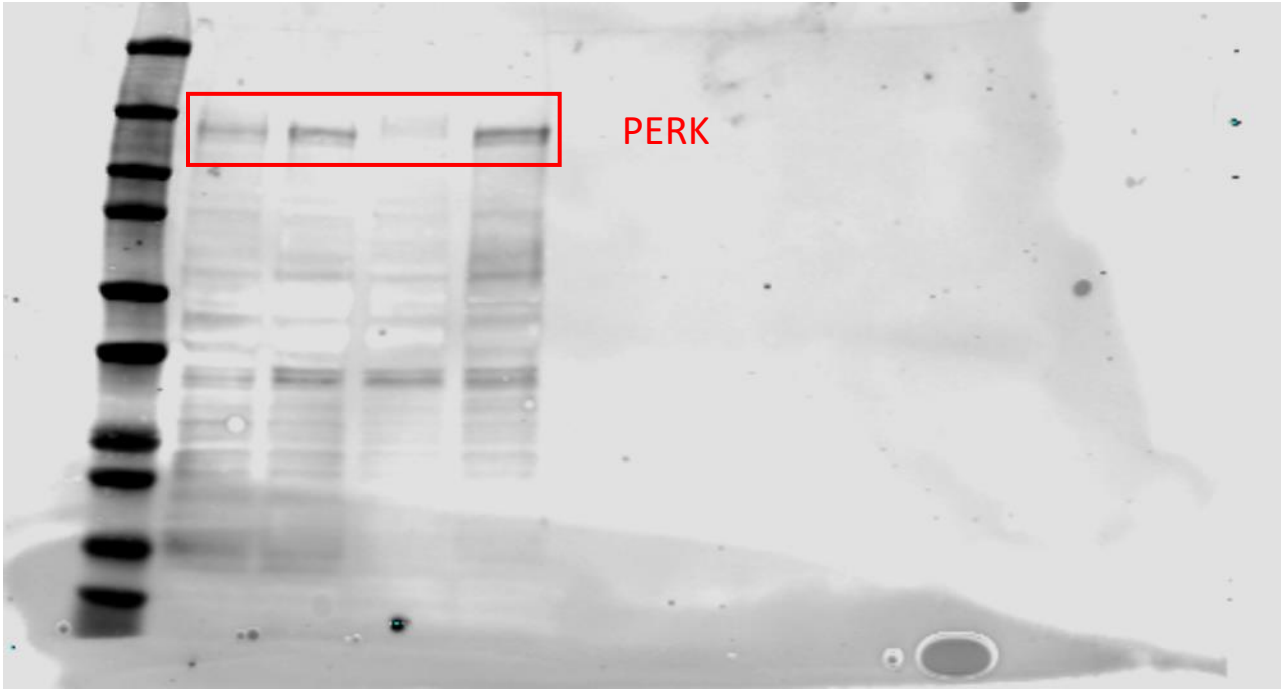

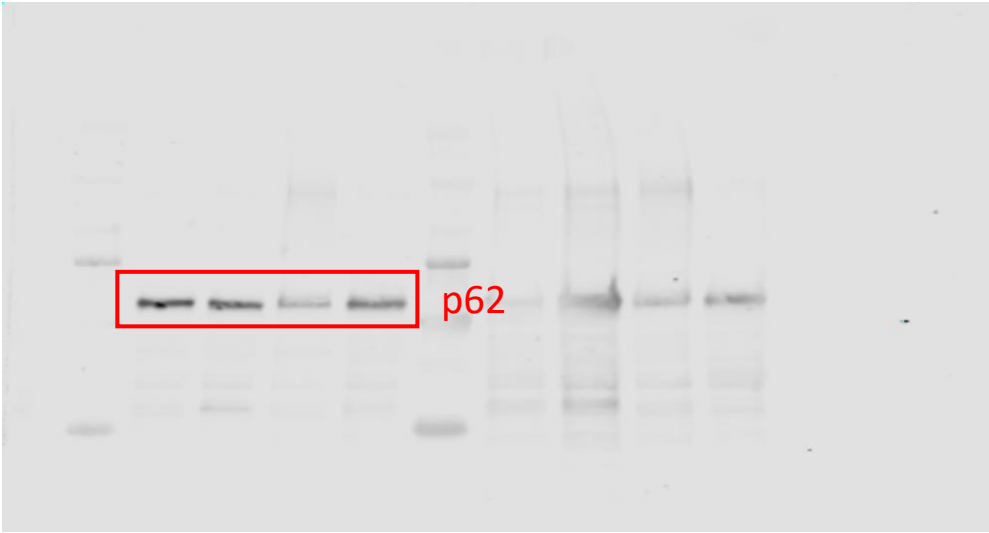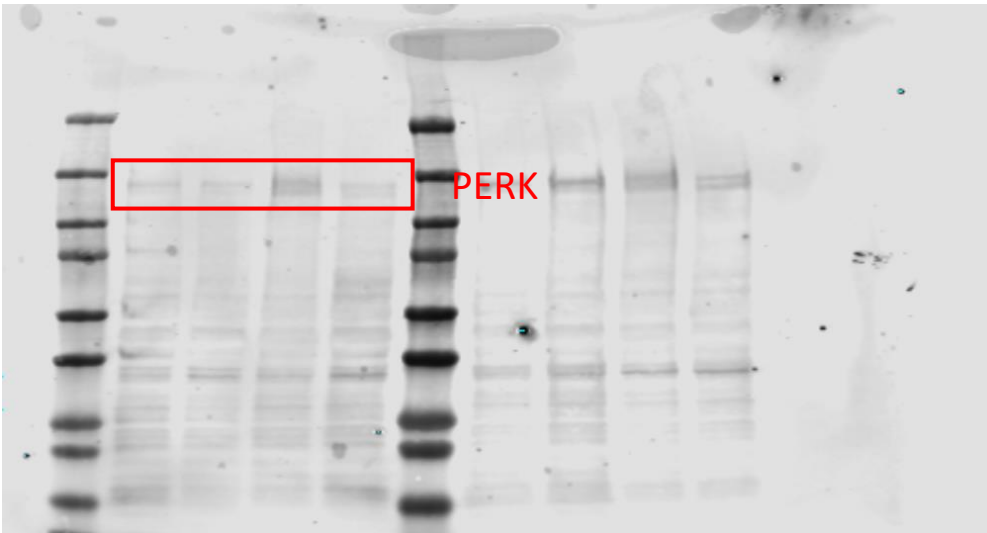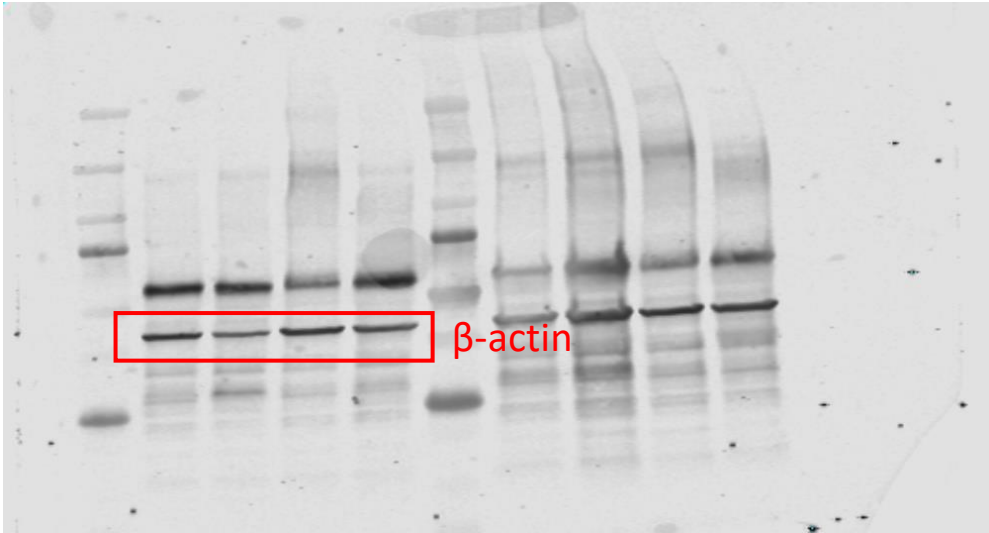

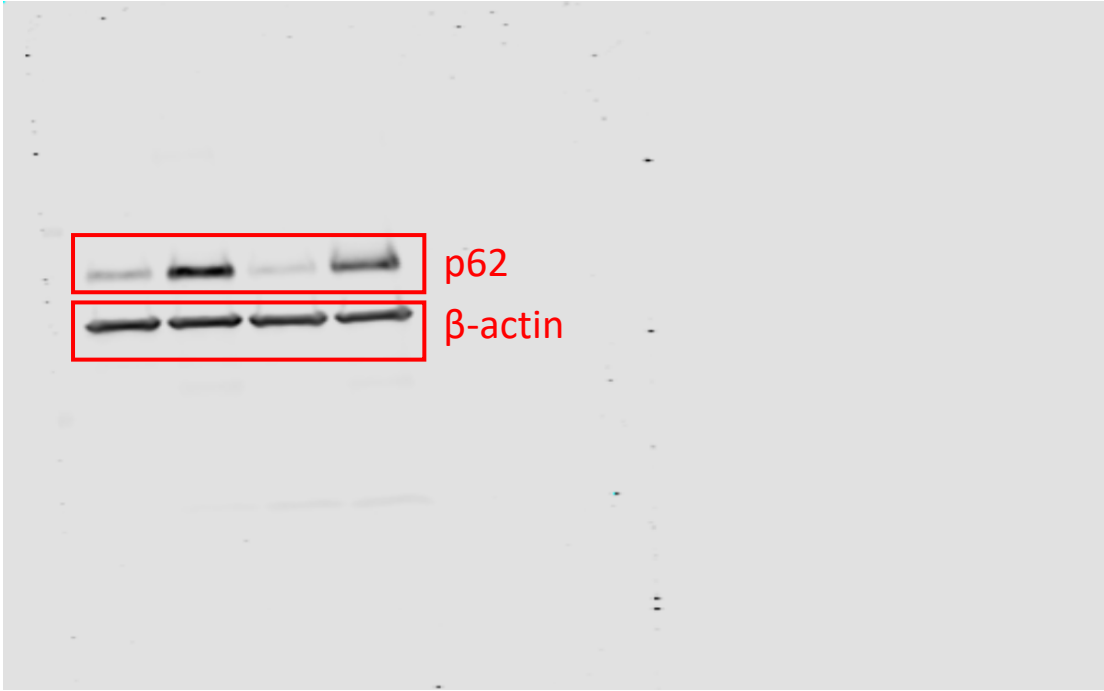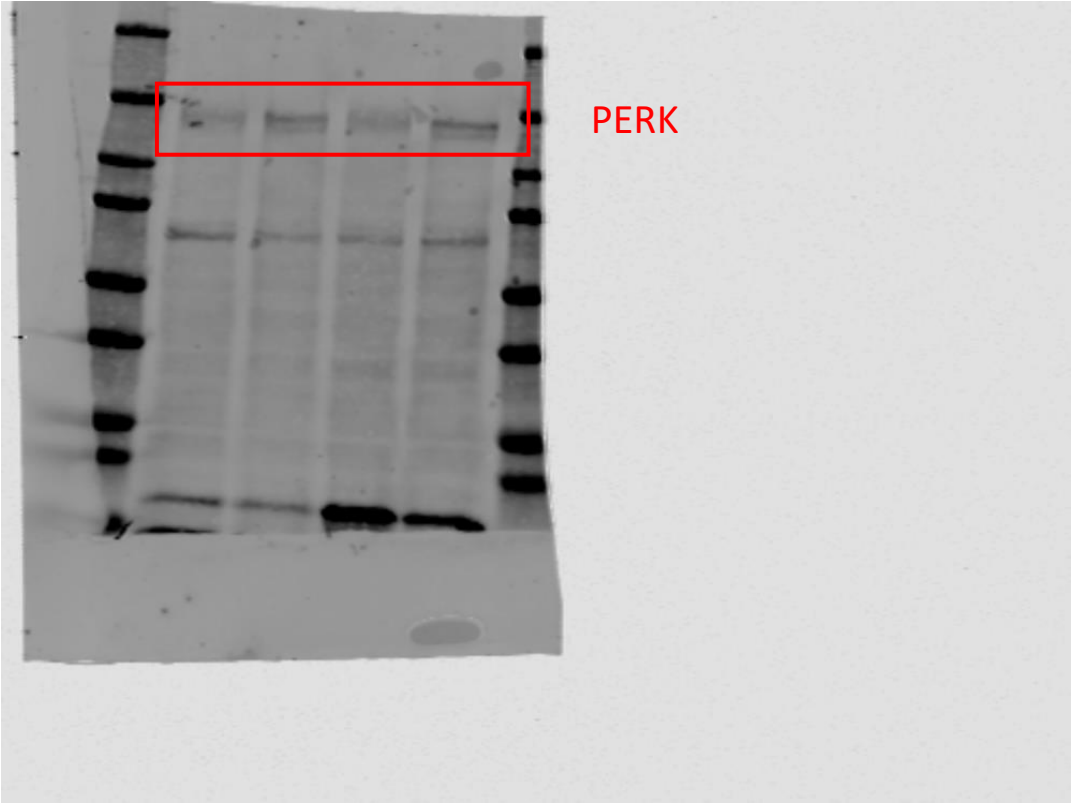

Supplement: Supplementary file 6 — Source data Fig. 4 [file 44318_2024_319_MOESM6_ESM.zip › EMBOJ-2024-117498-T-SourceDataForFigure4A-H/Figure 4 B/README/HT29_western_all biological replicates .pdf]

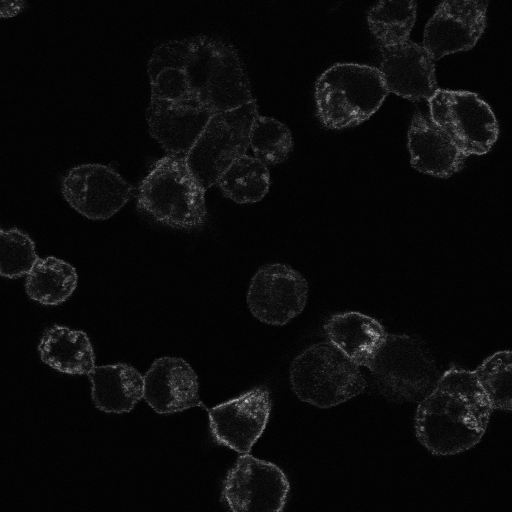

Supplement: Supplementary file 6 — Source data Fig. 4 [file 44318_2024_319_MOESM6_ESM.zip › EMBOJ-2024-117498-T-SourceDataForFigure4A-H/Figure 4 D/HT29 MHC I staining hypoxia 16h time point Baf A1.tif]

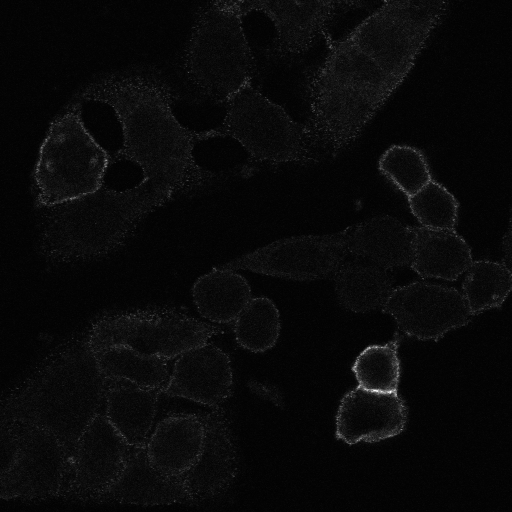

Supplement: Supplementary file 6 — Source data Fig. 4 [file 44318_2024_319_MOESM6_ESM.zip › EMBOJ-2024-117498-T-SourceDataForFigure4A-H/Figure 4 D/HT29 MHC I staining hypoxia 16h time point veh.tif]

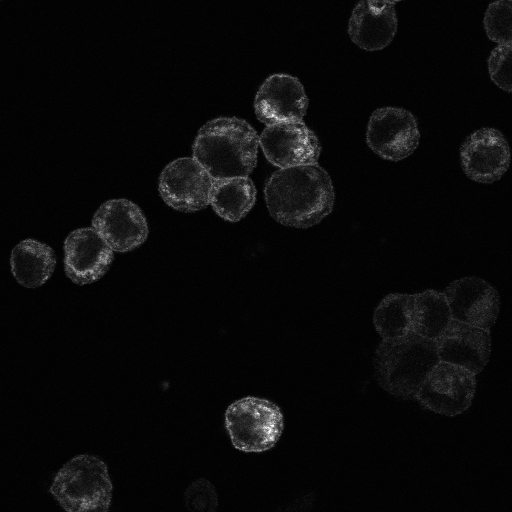

Supplement: Supplementary file 6 — Source data Fig. 4 [file 44318_2024_319_MOESM6_ESM.zip › EMBOJ-2024-117498-T-SourceDataForFigure4A-H/Figure 4 D/HT29 MHC I staining hypoxia 24h time point Baf A1.tif]

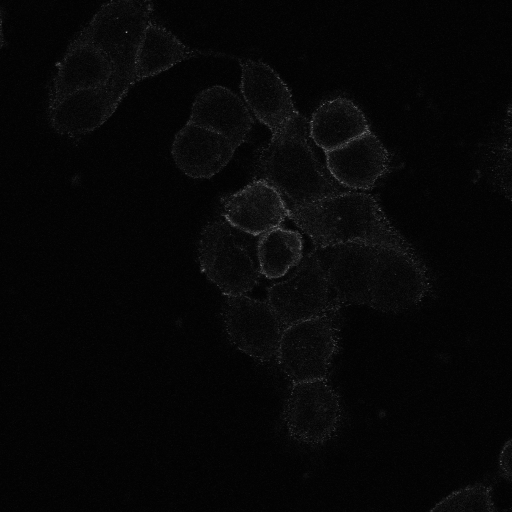

Supplement: Supplementary file 6 — Source data Fig. 4 [file 44318_2024_319_MOESM6_ESM.zip › EMBOJ-2024-117498-T-SourceDataForFigure4A-H/Figure 4 D/HT29 MHC I staining hypoxia 24h time point veh.tif]

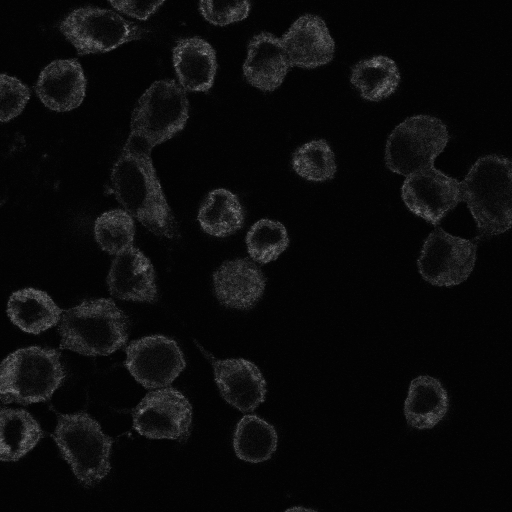

Supplement: Supplementary file 6 — Source data Fig. 4 [file 44318_2024_319_MOESM6_ESM.zip › EMBOJ-2024-117498-T-SourceDataForFigure4A-H/Figure 4 D/HT29 MHC I staining normoxia Baf A1.tif]

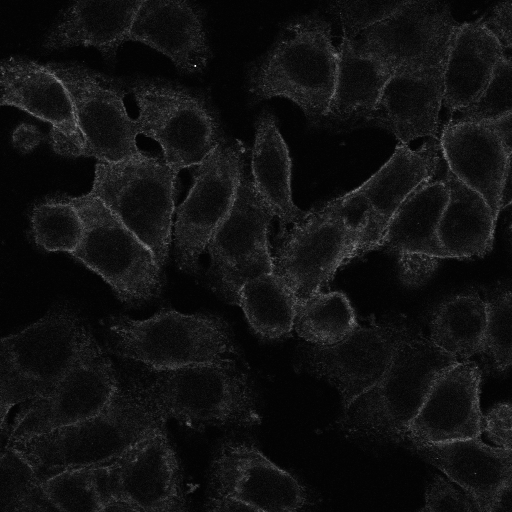

Supplement: Supplementary file 6 — Source data Fig. 4 [file 44318_2024_319_MOESM6_ESM.zip › EMBOJ-2024-117498-T-SourceDataForFigure4A-H/Figure 4 D/HT29 MHC I staining normoxia veh.tif]

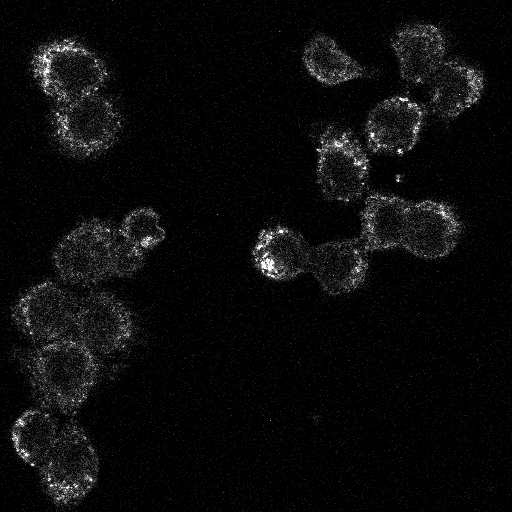

Supplement: Supplementary file 6 — Source data Fig. 4 [file 44318_2024_319_MOESM6_ESM.zip › EMBOJ-2024-117498-T-SourceDataForFigure4A-H/Figure 4 F/HT29 MHC I LAMP1 staining normoxia Baf A1.tif]

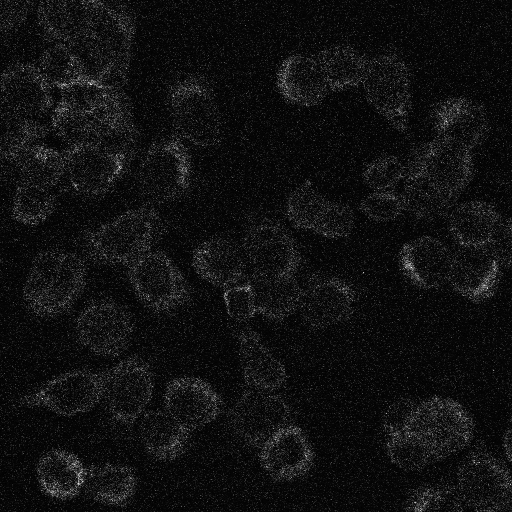

Supplement: Supplementary file 6 — Source data Fig. 4 [file 44318_2024_319_MOESM6_ESM.zip › EMBOJ-2024-117498-T-SourceDataForFigure4A-H/Figure 4 F/HT29 MHC I LAMP1 staining normoxia veh.tif]

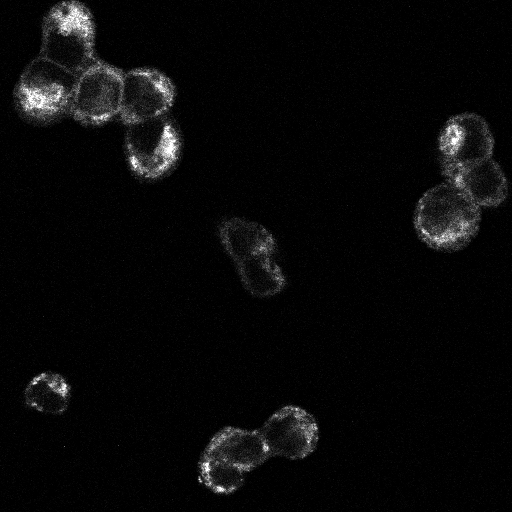

Supplement: Supplementary file 6 — Source data Fig. 4 [file 44318_2024_319_MOESM6_ESM.zip › EMBOJ-2024-117498-T-SourceDataForFigure4A-H/Figure 4 F/HT29 MHC I LAMP1 staining hypoxia 0.1% 16h time point Baf A1.tif]

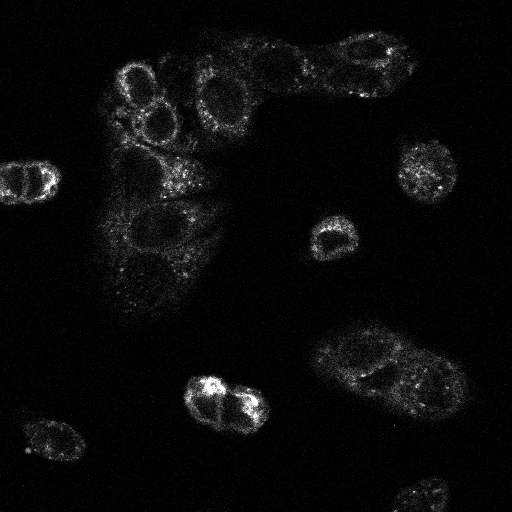

Supplement: Supplementary file 6 — Source data Fig. 4 [file 44318_2024_319_MOESM6_ESM.zip › EMBOJ-2024-117498-T-SourceDataForFigure4A-H/Figure 4 F/HT29 MHC I LAMP1 staining hypoxia 0.1% 16h time point veh.tif]

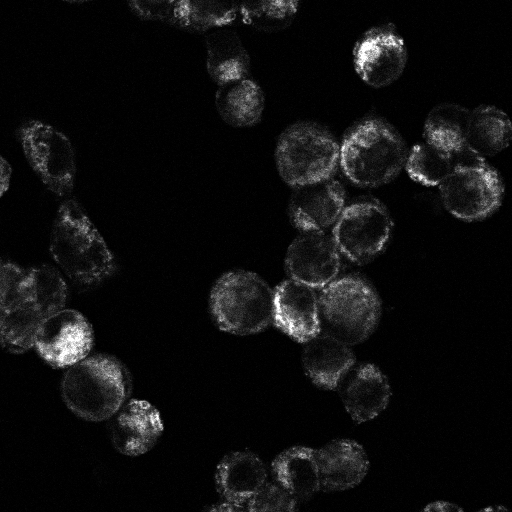

Supplement: Supplementary file 6 — Source data Fig. 4 [file 44318_2024_319_MOESM6_ESM.zip › EMBOJ-2024-117498-T-SourceDataForFigure4A-H/Figure 4 F/HT29 MHC I LAMP1 staining hypoxia 0.1% 24h time point Baf A1.tif]

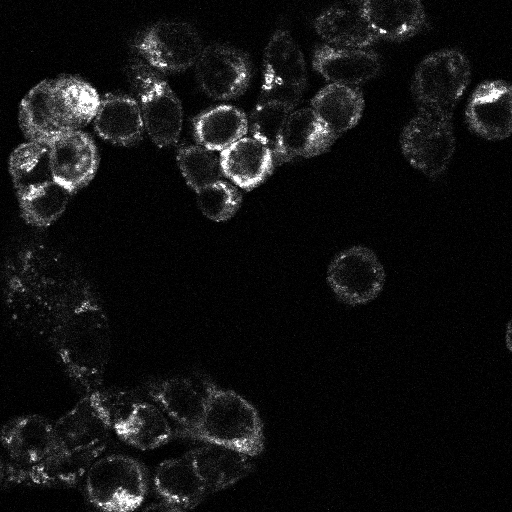

Supplement: Supplementary file 6 — Source data Fig. 4 [file 44318_2024_319_MOESM6_ESM.zip › EMBOJ-2024-117498-T-SourceDataForFigure4A-H/Figure 4 F/HT29 MHC I LAMP1 staining hypoxia 0.1% 24h time point veh.tif]

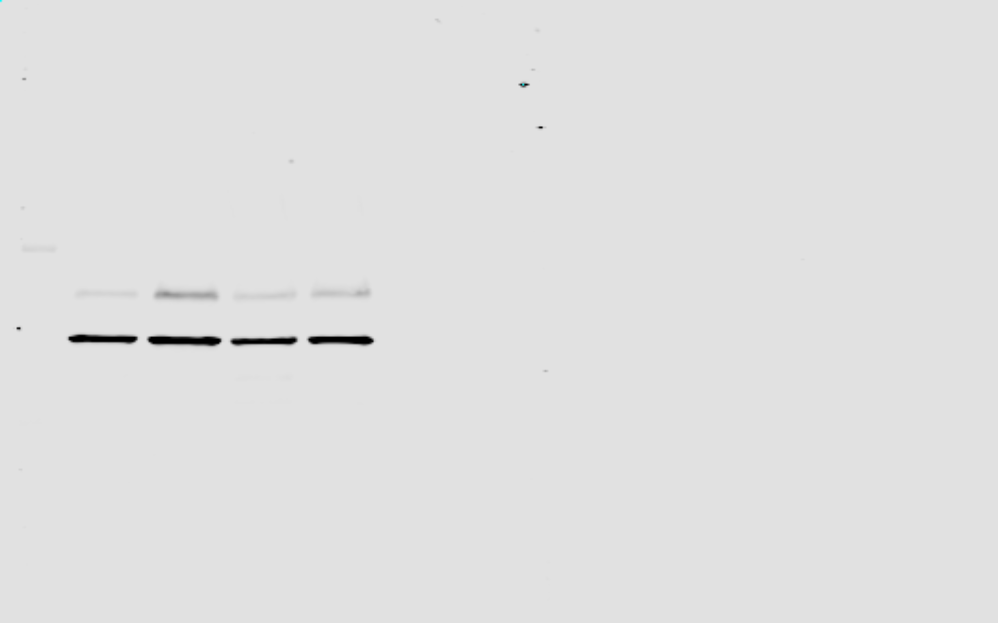

Supplement: Supplementary file 6 — Source data Fig. 4 [file 44318_2024_319_MOESM6_ESM.zip › EMBOJ-2024-117498-T-SourceDataForFigure4A-H/Figure 4 H/HT29_biological replicate n1/HT29_actin_Western.tif]

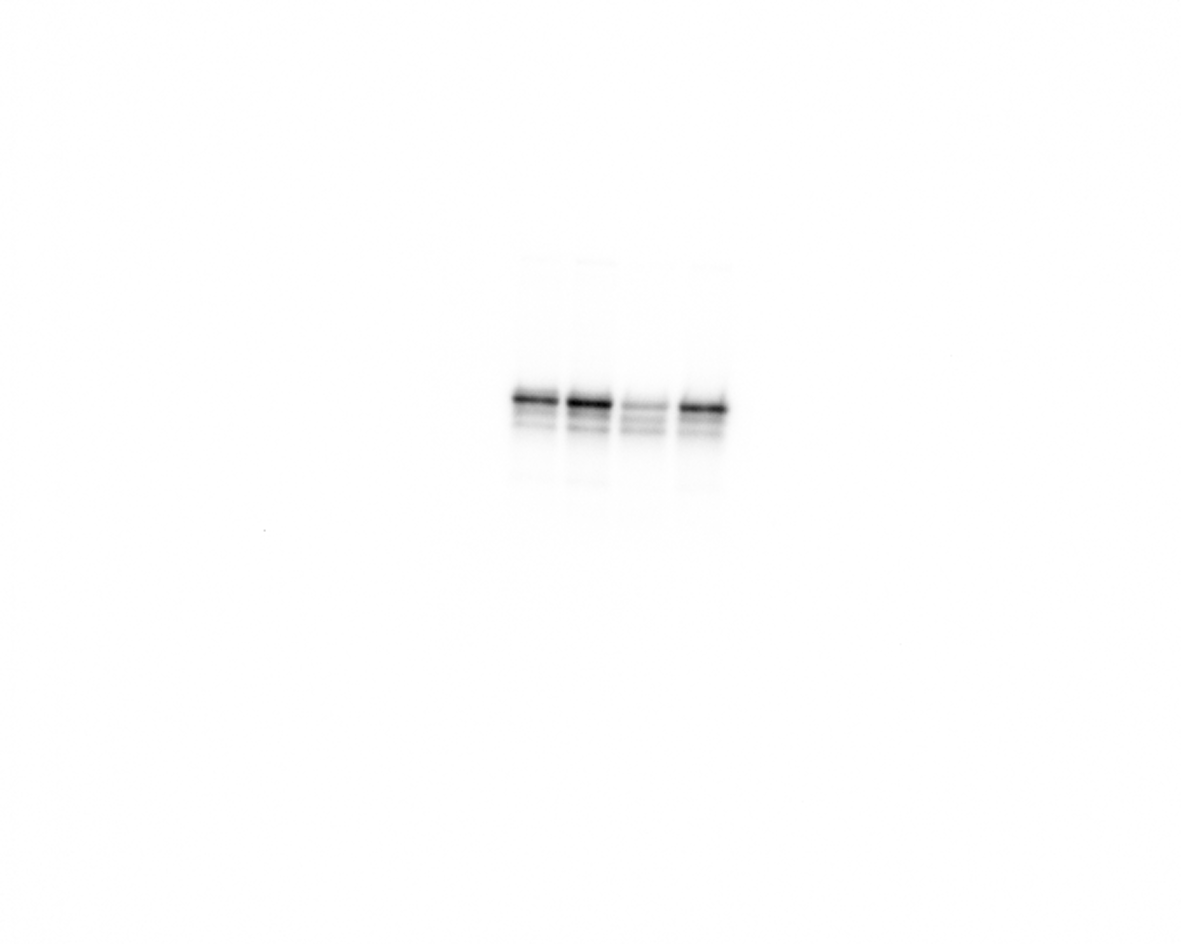

Supplement: Supplementary file 6 — Source data Fig. 4 [file 44318_2024_319_MOESM6_ESM.zip › EMBOJ-2024-117498-T-SourceDataForFigure4A-H/Figure 4 H/HT29_biological replicate n1/HT29_MHC I_western.tif]

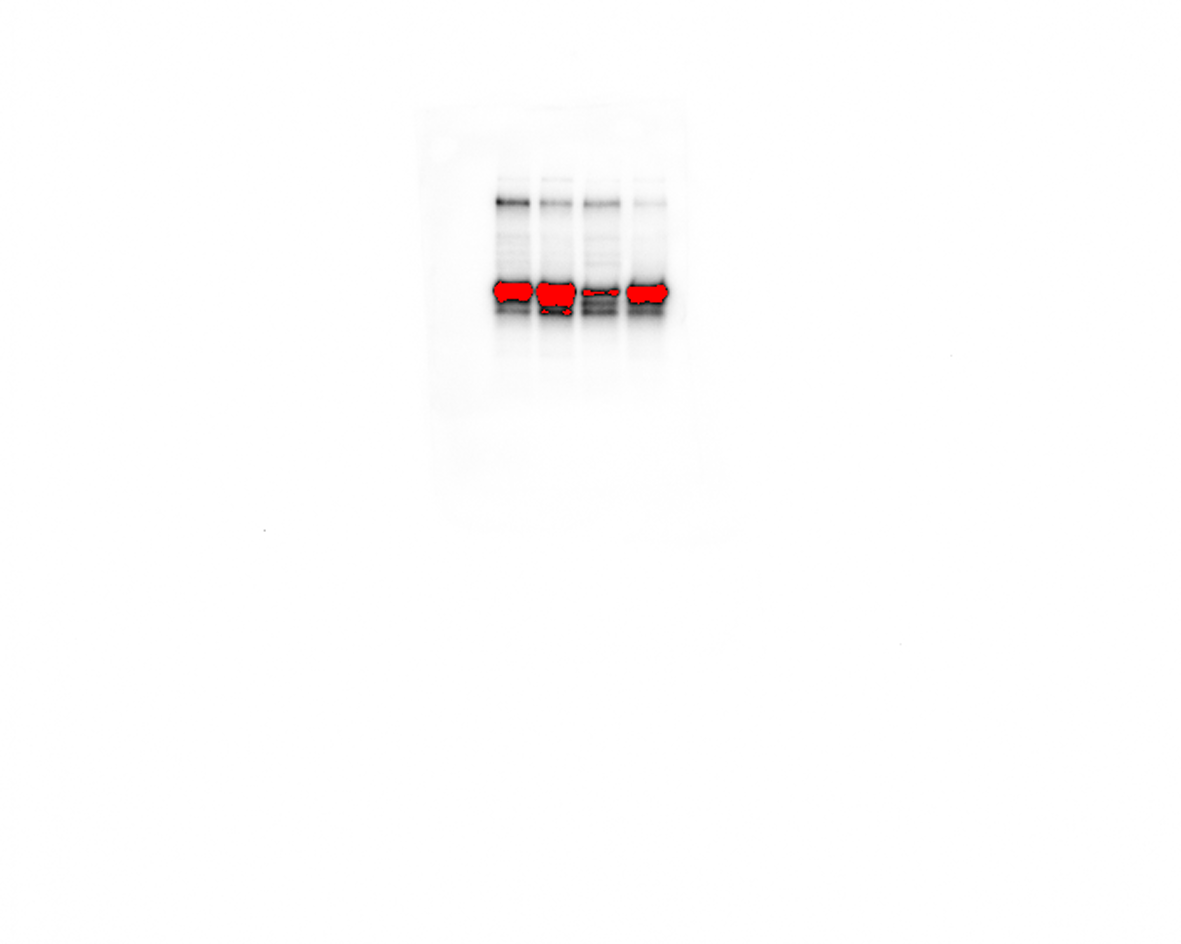

Supplement: Supplementary file 6 — Source data Fig. 4 [file 44318_2024_319_MOESM6_ESM.zip › EMBOJ-2024-117498-T-SourceDataForFigure4A-H/Figure 4 H/HT29_biological replicate n1/HT29_NBR1_western.tif]

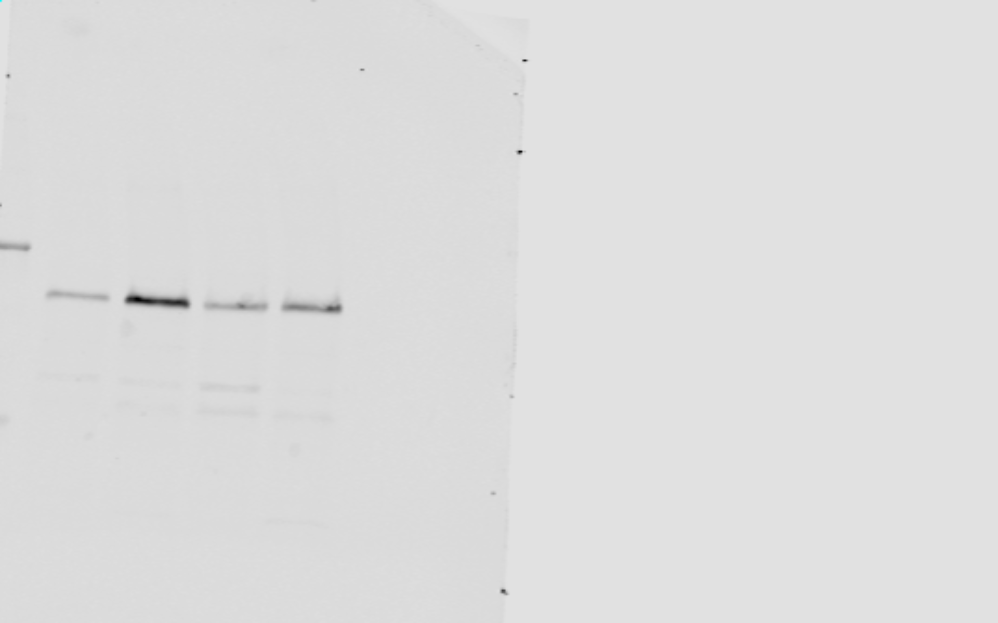

Supplement: Supplementary file 6 — Source data Fig. 4 [file 44318_2024_319_MOESM6_ESM.zip › EMBOJ-2024-117498-T-SourceDataForFigure4A-H/Figure 4 H/HT29_biological replicate n1/HT29_p62_Western.tif.tif]

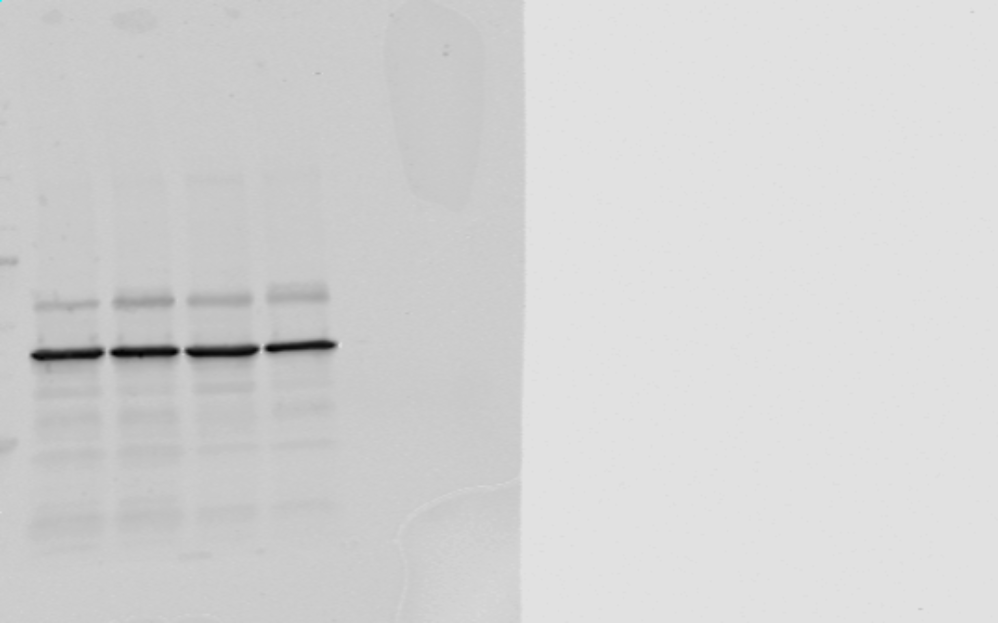

Supplement: Supplementary file 6 — Source data Fig. 4 [file 44318_2024_319_MOESM6_ESM.zip › EMBOJ-2024-117498-T-SourceDataForFigure4A-H/Figure 4 H/HT29_biological replicate n2/HT29_actin_Western.tif.tif]

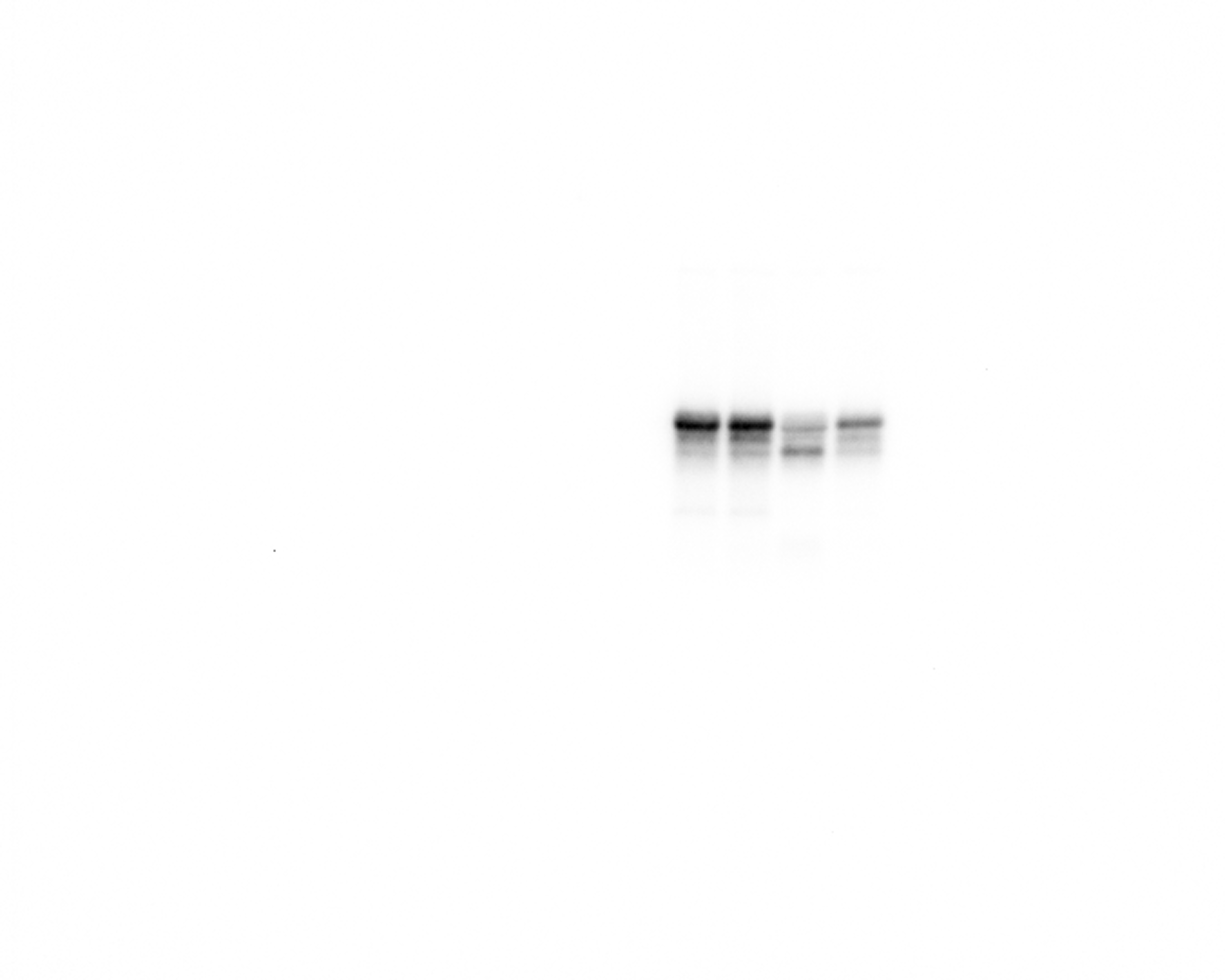

Supplement: Supplementary file 6 — Source data Fig. 4 [file 44318_2024_319_MOESM6_ESM.zip › EMBOJ-2024-117498-T-SourceDataForFigure4A-H/Figure 4 H/HT29_biological replicate n2/HT29_MHC I_western.tif]

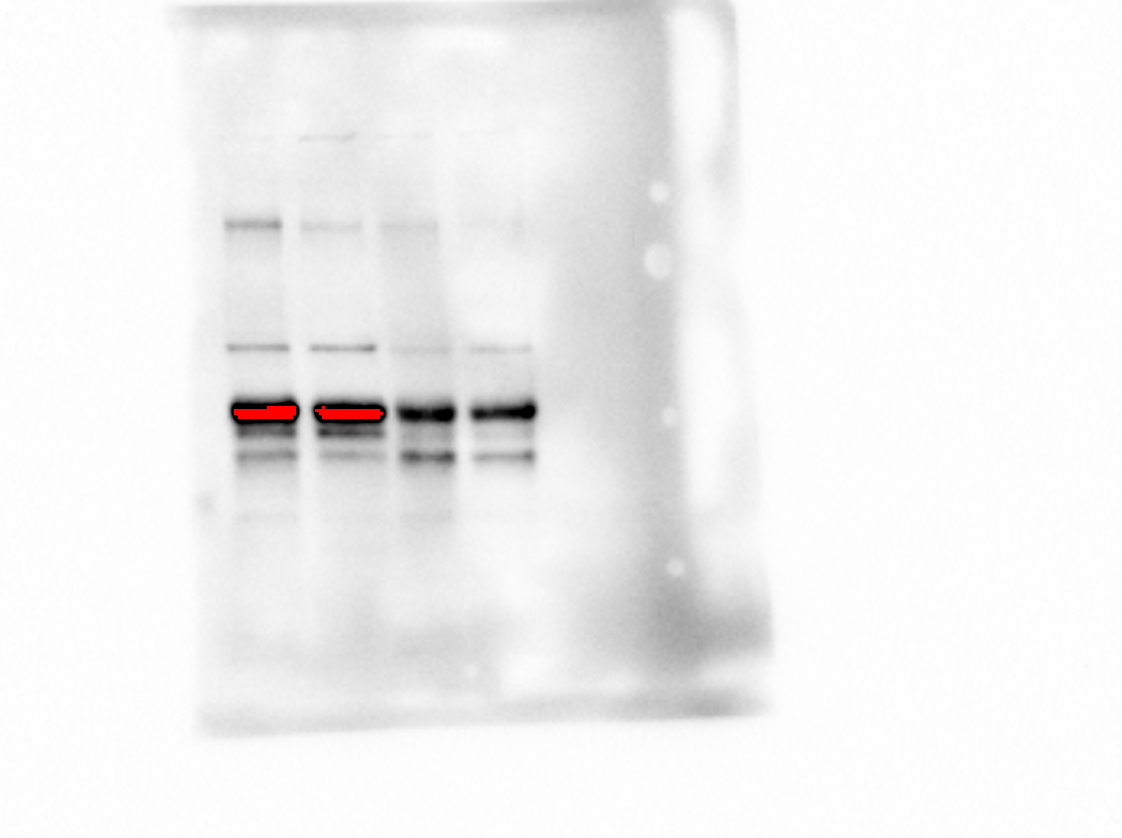

Supplement: Supplementary file 6 — Source data Fig. 4 [file 44318_2024_319_MOESM6_ESM.zip › EMBOJ-2024-117498-T-SourceDataForFigure4A-H/Figure 4 H/HT29_biological replicate n2/HT29_NBR1_western.tif]

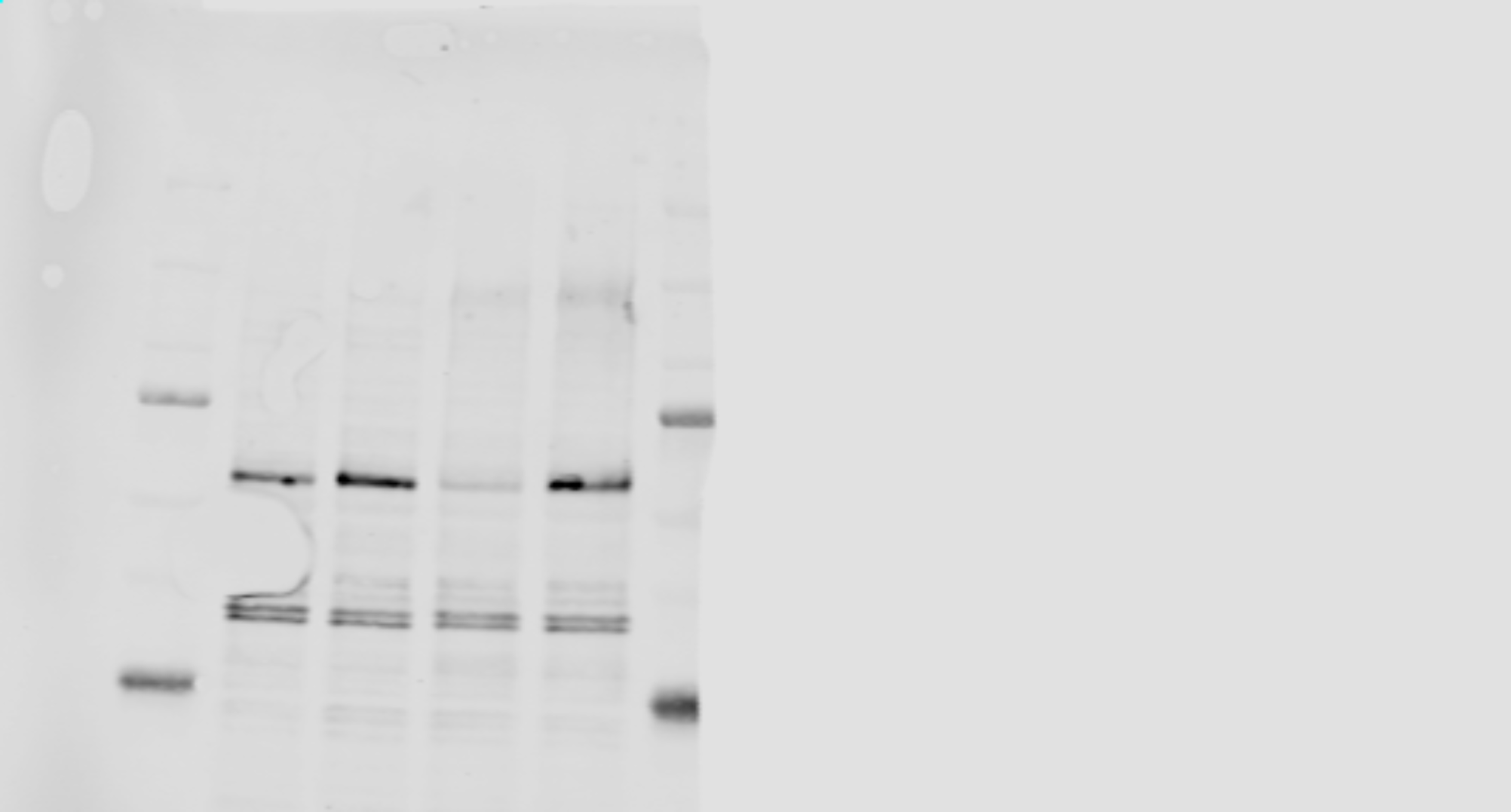

Supplement: Supplementary file 6 — Source data Fig. 4 [file 44318_2024_319_MOESM6_ESM.zip › EMBOJ-2024-117498-T-SourceDataForFigure4A-H/Figure 4 H/HT29_biological replicate n2/HT29_p62_western.tif]

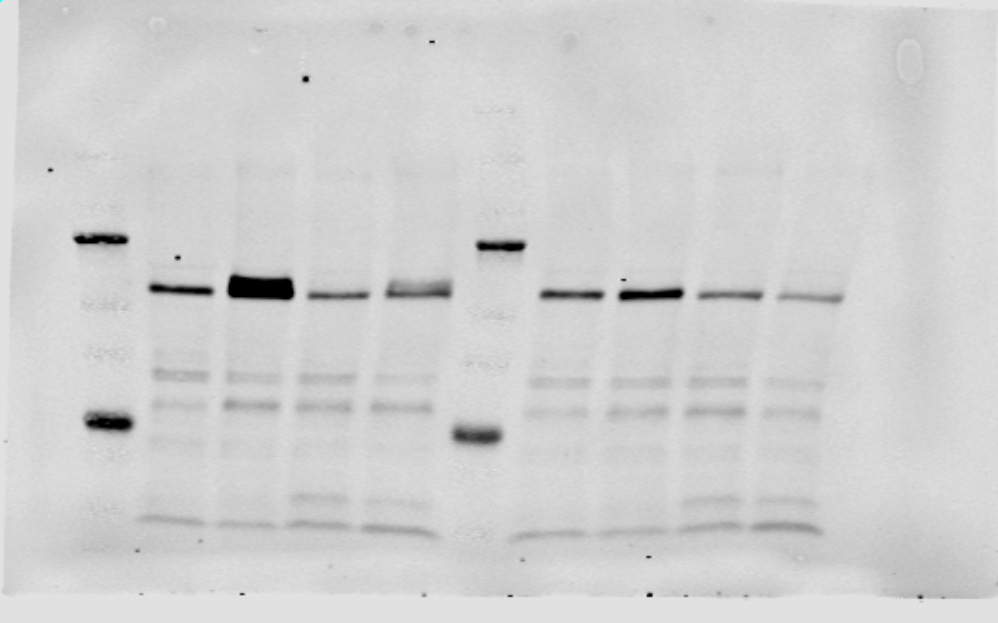

Supplement: Supplementary file 6 — Source data Fig. 4 [file 44318_2024_319_MOESM6_ESM.zip › EMBOJ-2024-117498-T-SourceDataForFigure4A-H/Figure 4 H/HT29_biological replicate n2/HT29_p62_Western.tif.tif]

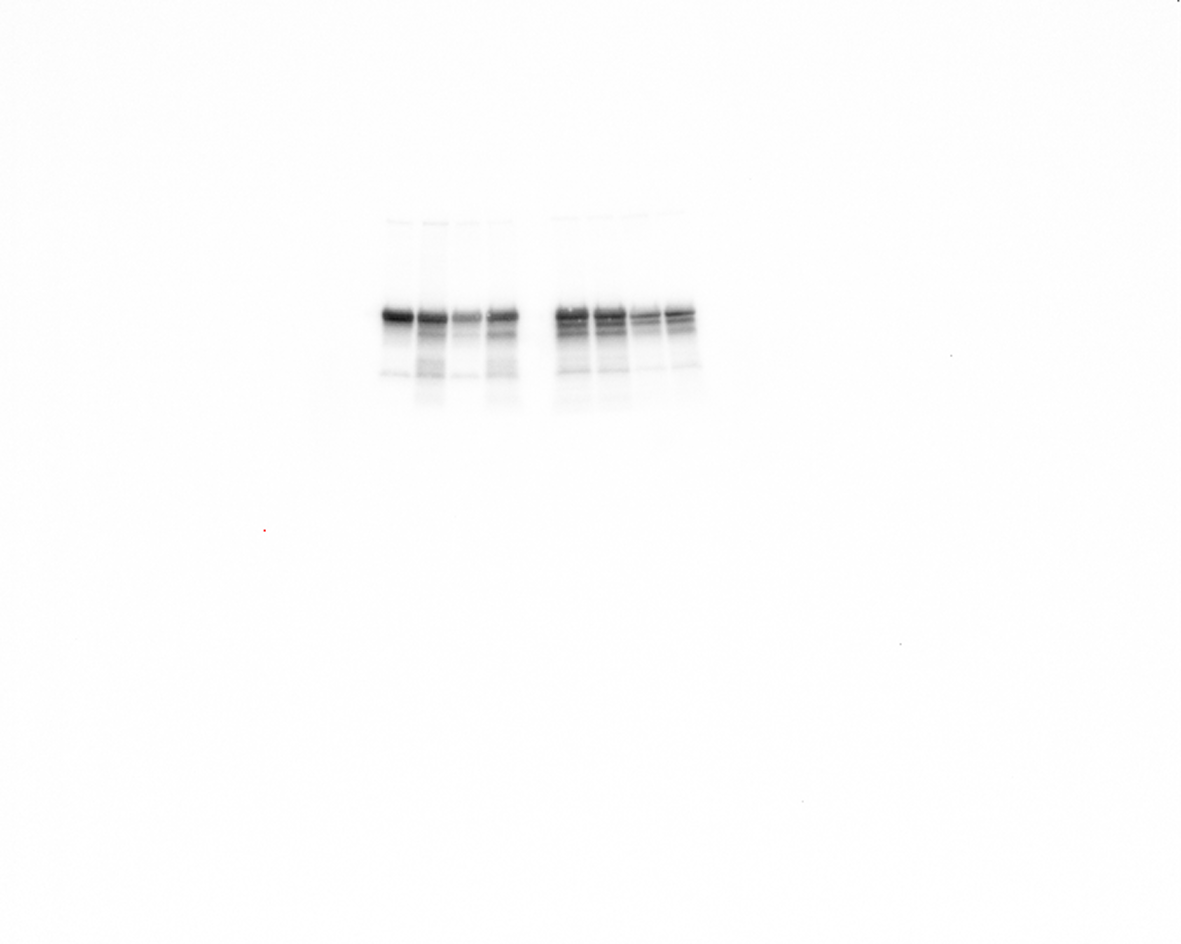

Supplement: Supplementary file 6 — Source data Fig. 4 [file 44318_2024_319_MOESM6_ESM.zip › EMBOJ-2024-117498-T-SourceDataForFigure4A-H/Figure 4 H/HT29_biological replicate n3/HT29_MHC I_western.tif]

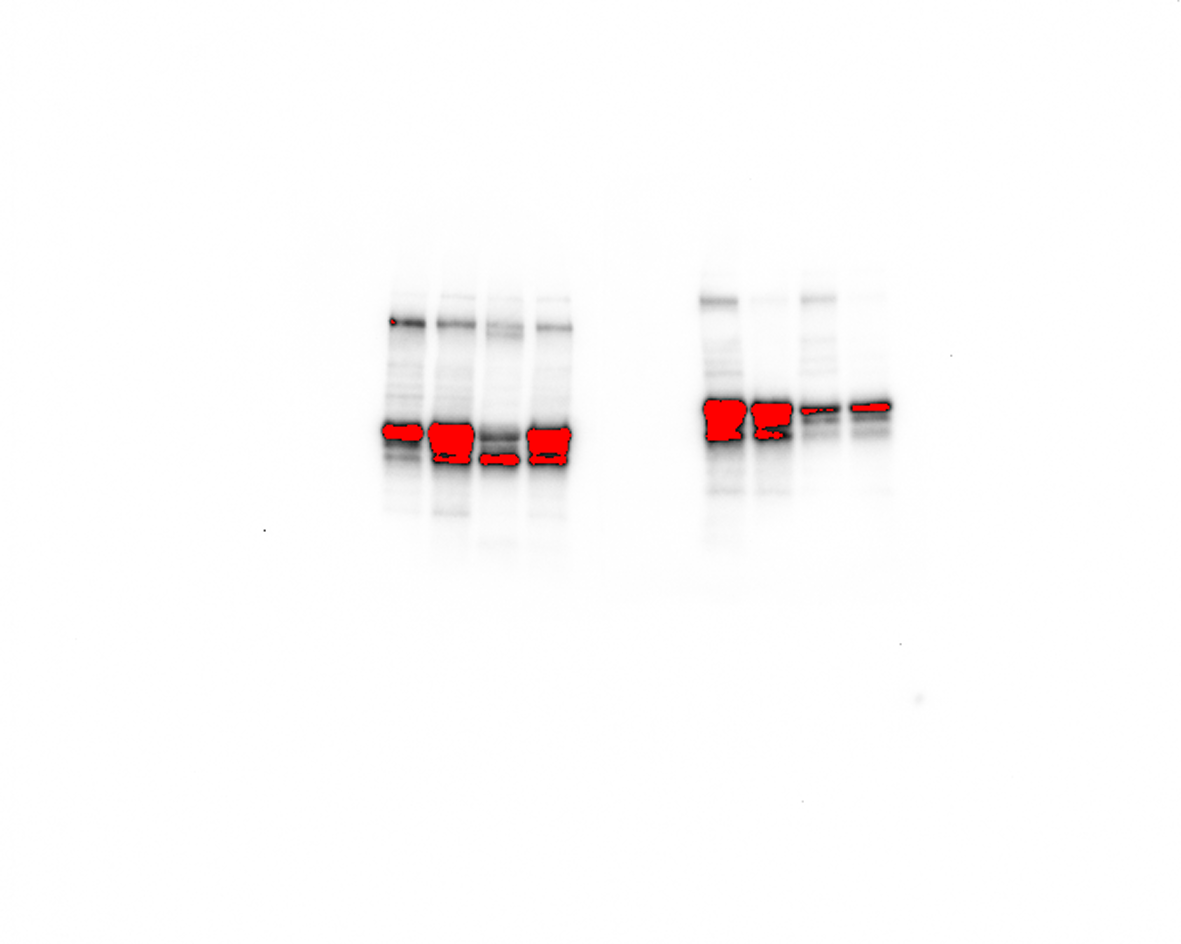

Supplement: Supplementary file 6 — Source data Fig. 4 [file 44318_2024_319_MOESM6_ESM.zip › EMBOJ-2024-117498-T-SourceDataForFigure4A-H/Figure 4 H/HT29_biological replicate n3/HT29_NBR1_western.tif]

Biological replicate n1

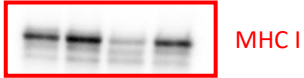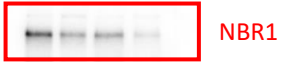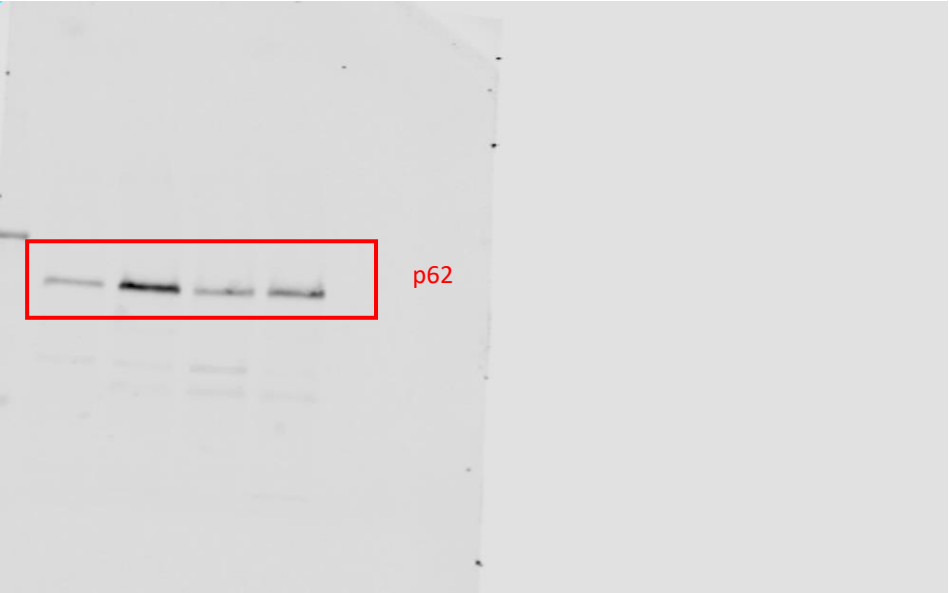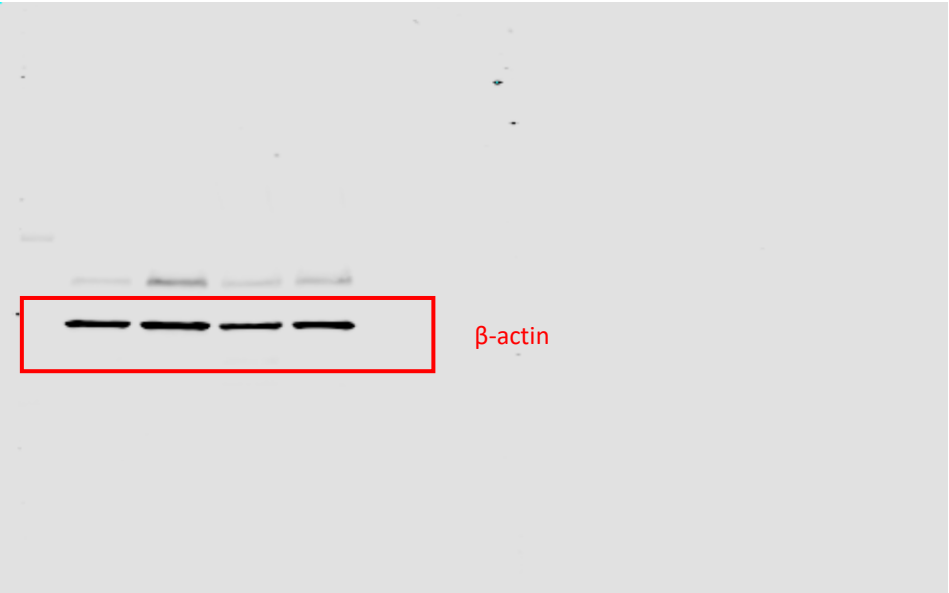

Biological replicate n2

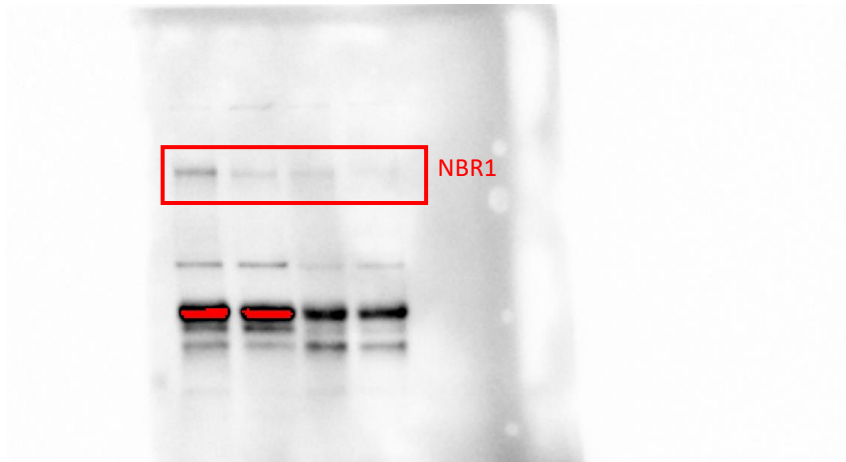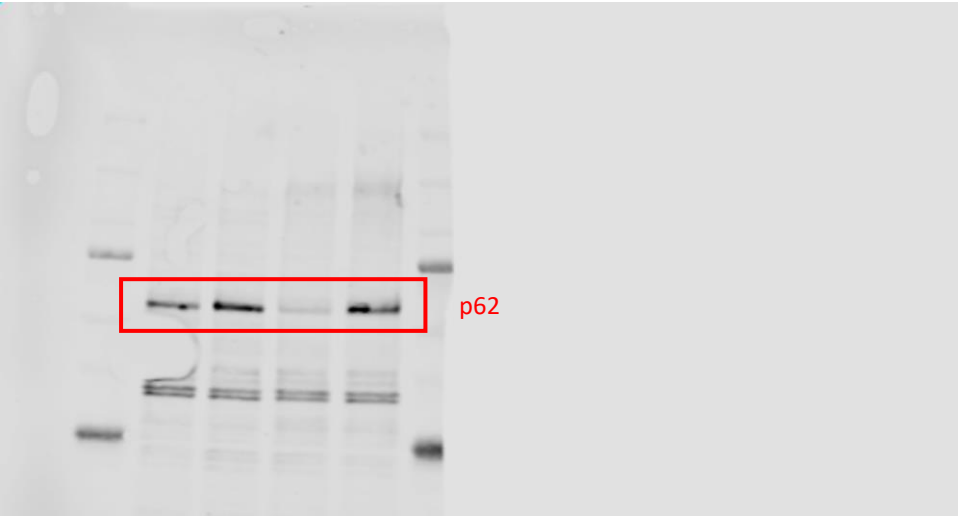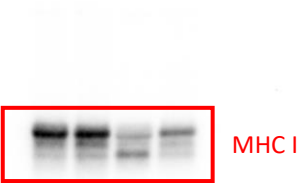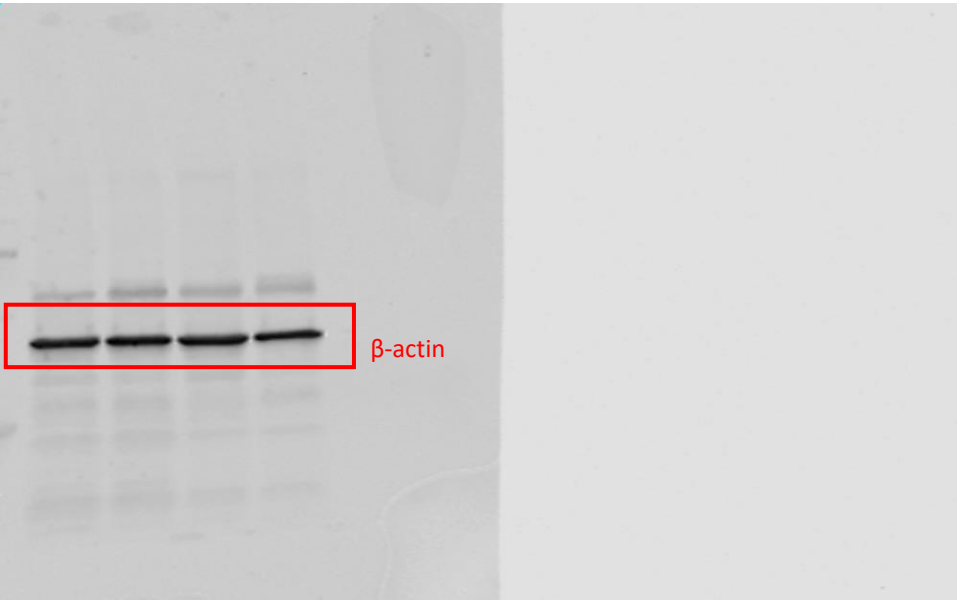

Biological replicate n3

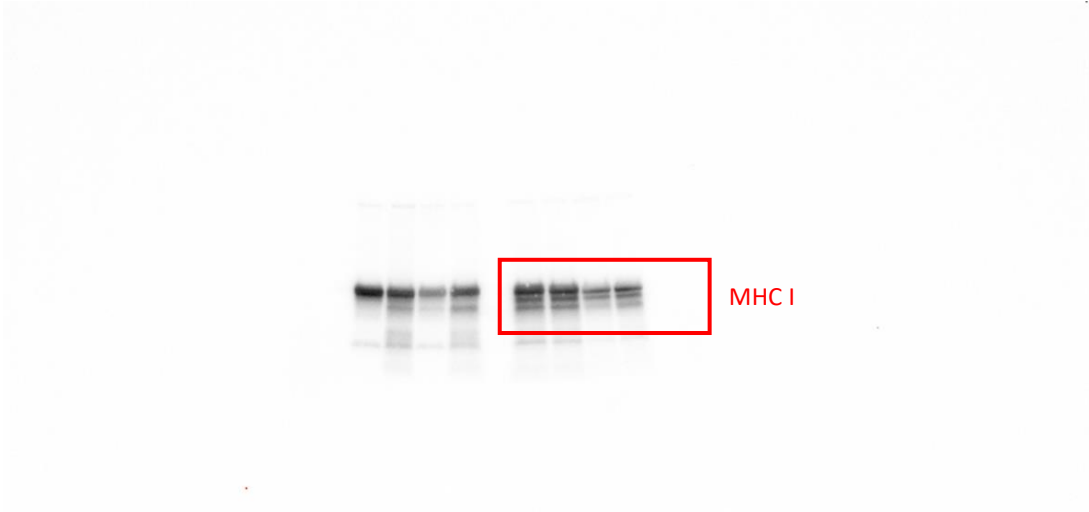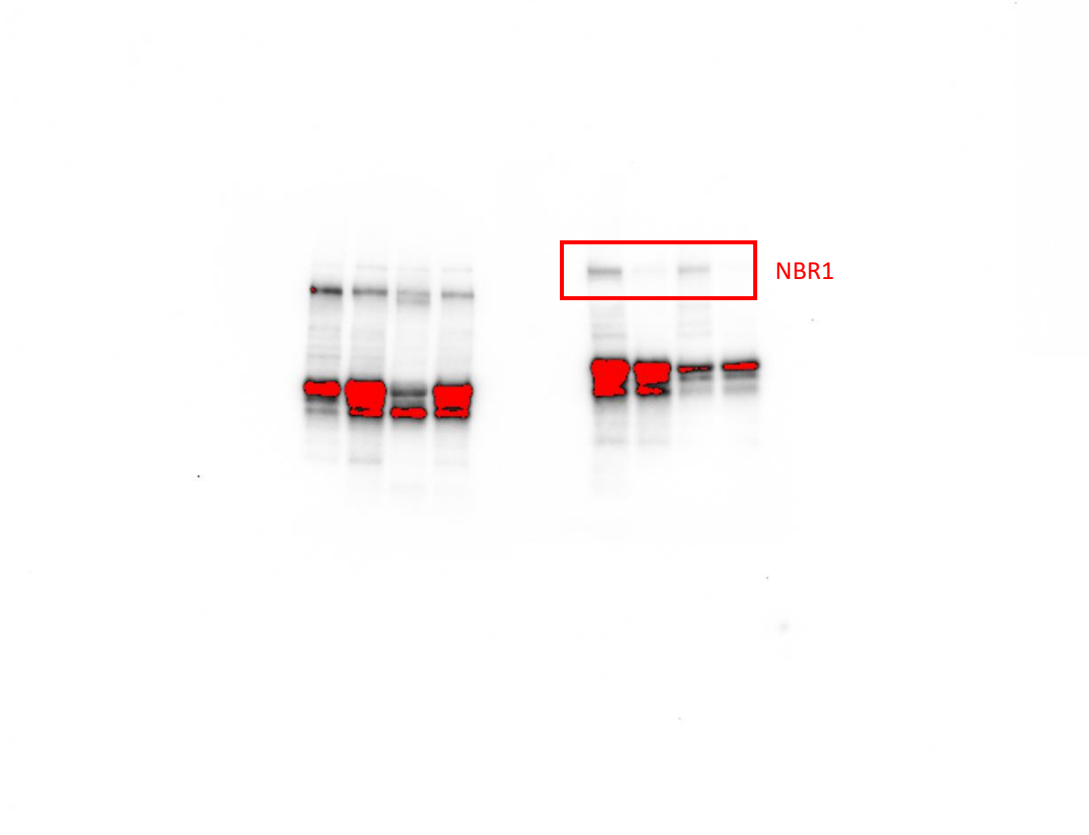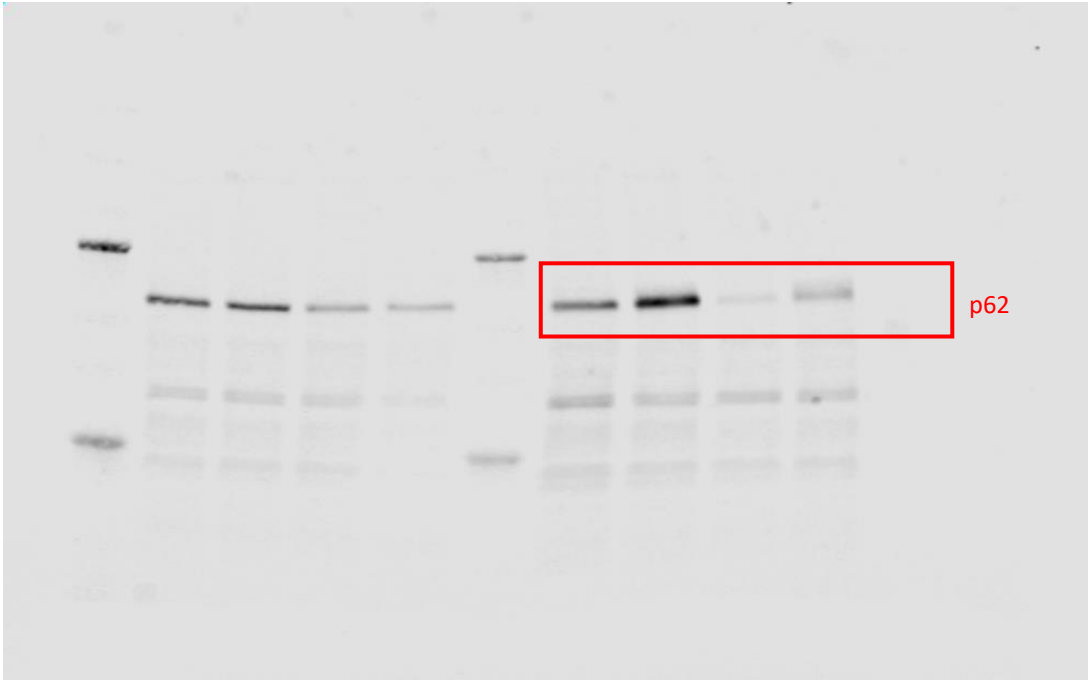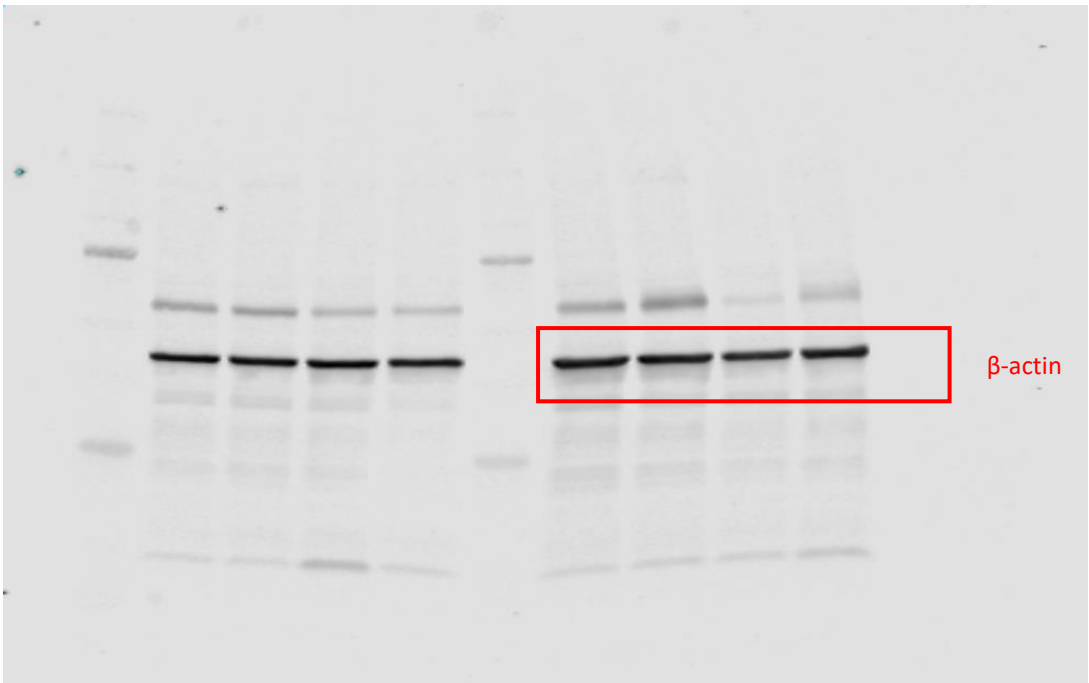

Supplement: Supplementary file 6 — Source data Fig. 4 [file 44318_2024_319_MOESM6_ESM.zip › EMBOJ-2024-117498-T-SourceDataForFigure4A-H/Figure 4 H/README/HT29_western_biological replicates.pdf]
