# Supplementary material for: Hypoxia promotes tumor immune evasion by suppressing MHC-I expression and antigen presentation
Source: EMBO J. 2025 Jan 3;44(3):903–22. doi: 10.1038/s44318-024-00319-7 (PMC11790895; doi:10.1038/s44318-024-00319-7)
Supplement: Supplementary file 4 — Source data Fig. 2 [file 44318_2024_319_MOESM4_ESM.zip › EMBOJ-2024-117498-T-SourceDataForFigure2B-J/Figure 2 D/README/HT29_western_Biological replicates.pptx]

## Slide 1
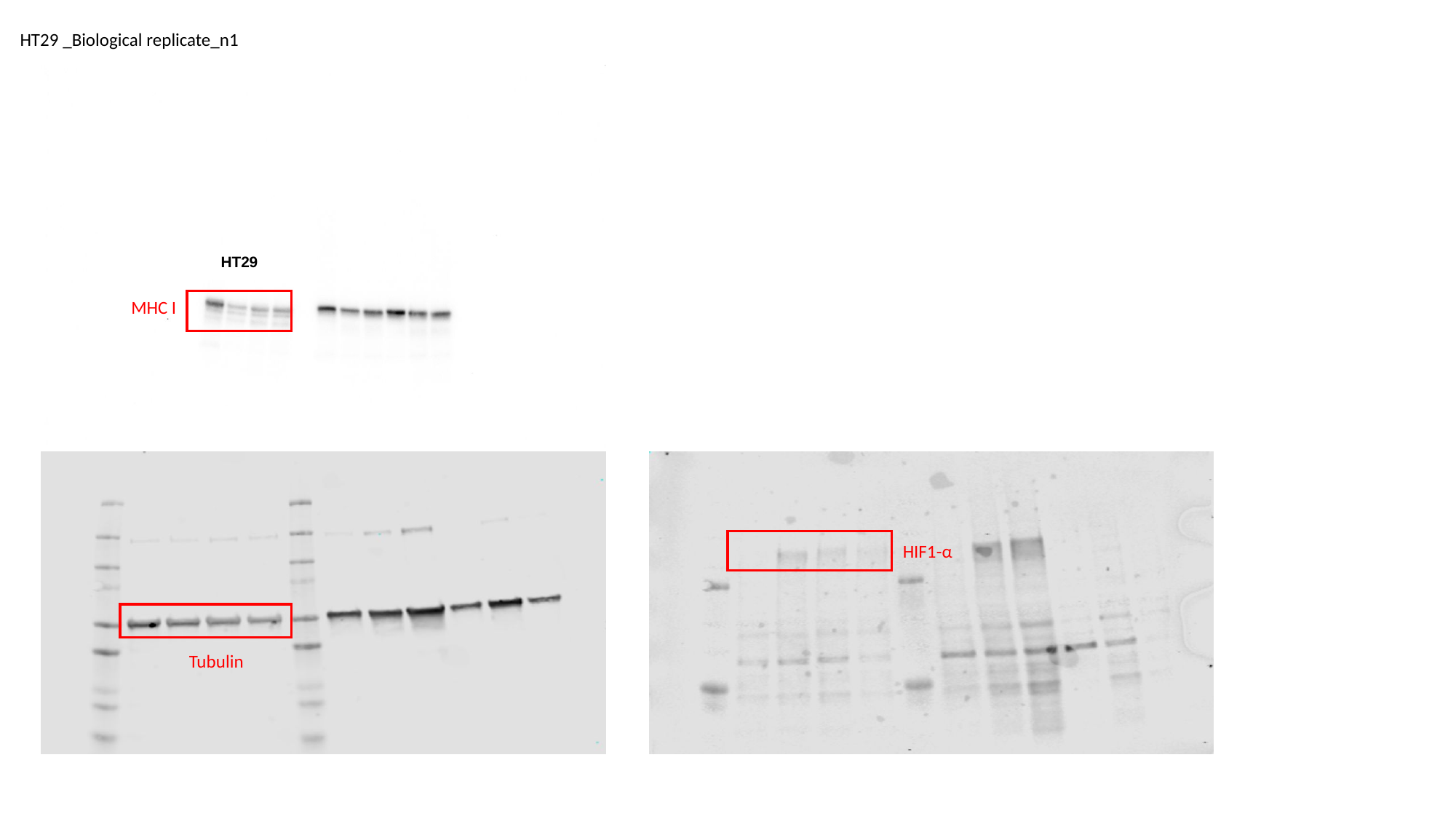

HT29 _Biological replicate_n1
HT29
MHC I
HIF1-α
Tubulin

## Slide 2
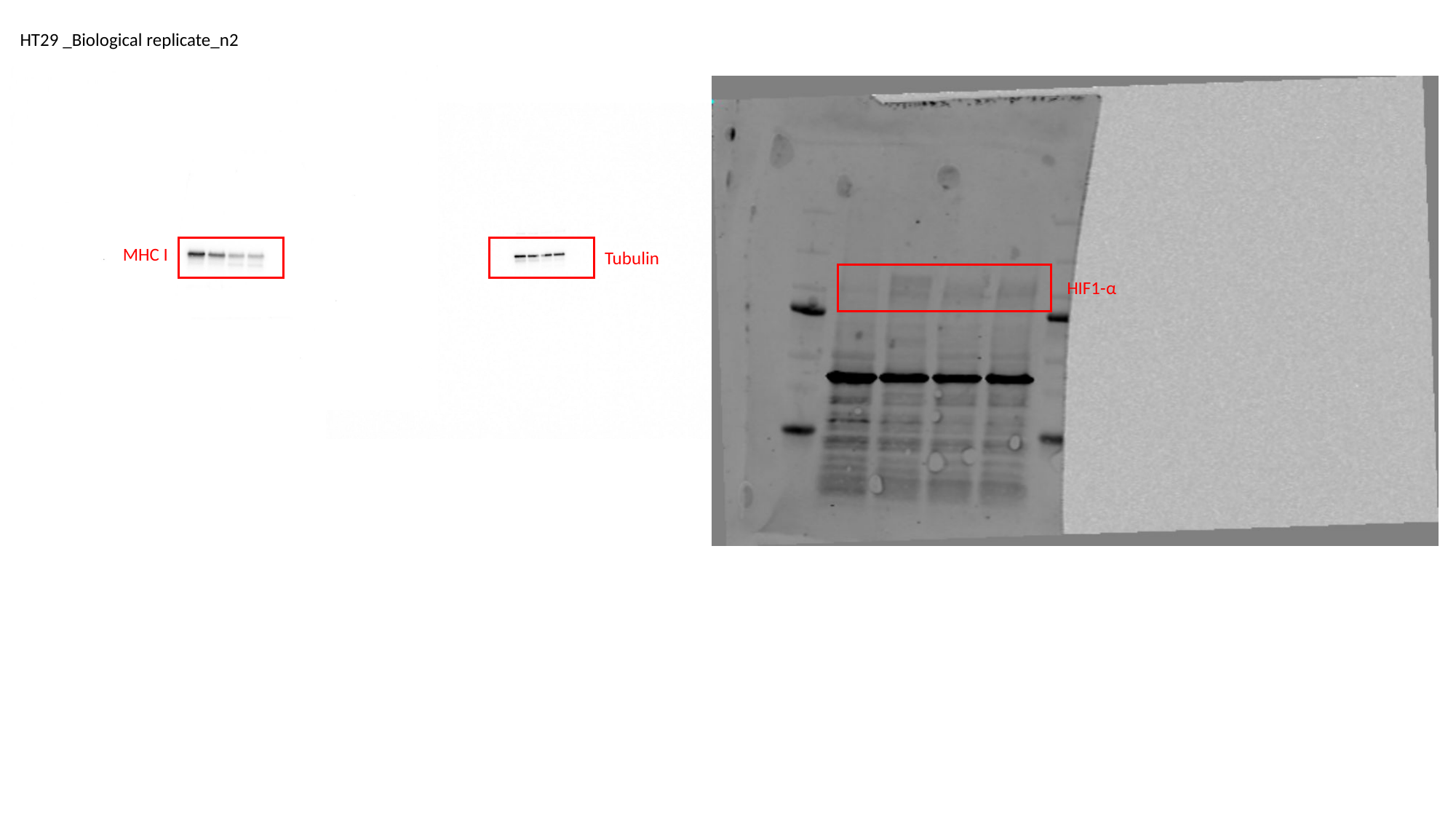

HT29 _Biological replicate_n2
MHC I
Tubulin
HIF1-α

## Slide 3
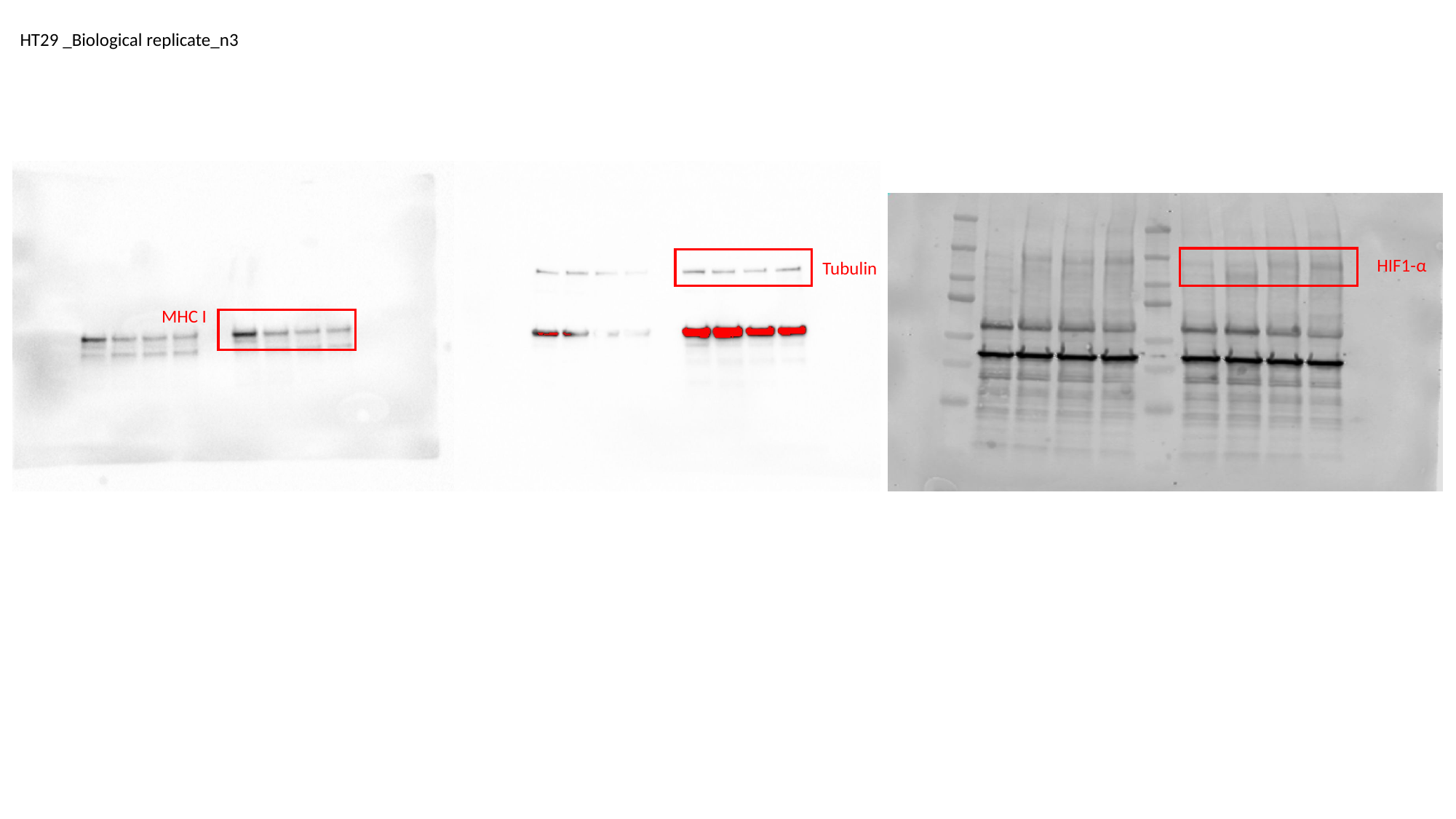

HT29 _Biological replicate_n3
HIF1-α
Tubulin
MHC I
