# Supplementary material for: Hypoxia promotes tumor immune evasion by suppressing MHC-I expression and antigen presentation
Source: EMBO J. 2025 Jan 3;44(3):903–22. doi: 10.1038/s44318-024-00319-7 (PMC11790895; doi:10.1038/s44318-024-00319-7)
Supplement: Supplementary file 5 — Source data Fig. 3 [file 44318_2024_319_MOESM5_ESM.zip › EMBOJ-2024-117498-T-SourceDataForFigure3A-J/Figure 3 B/README/HT29_ biological replicates_western.pptx]

## Slide 1
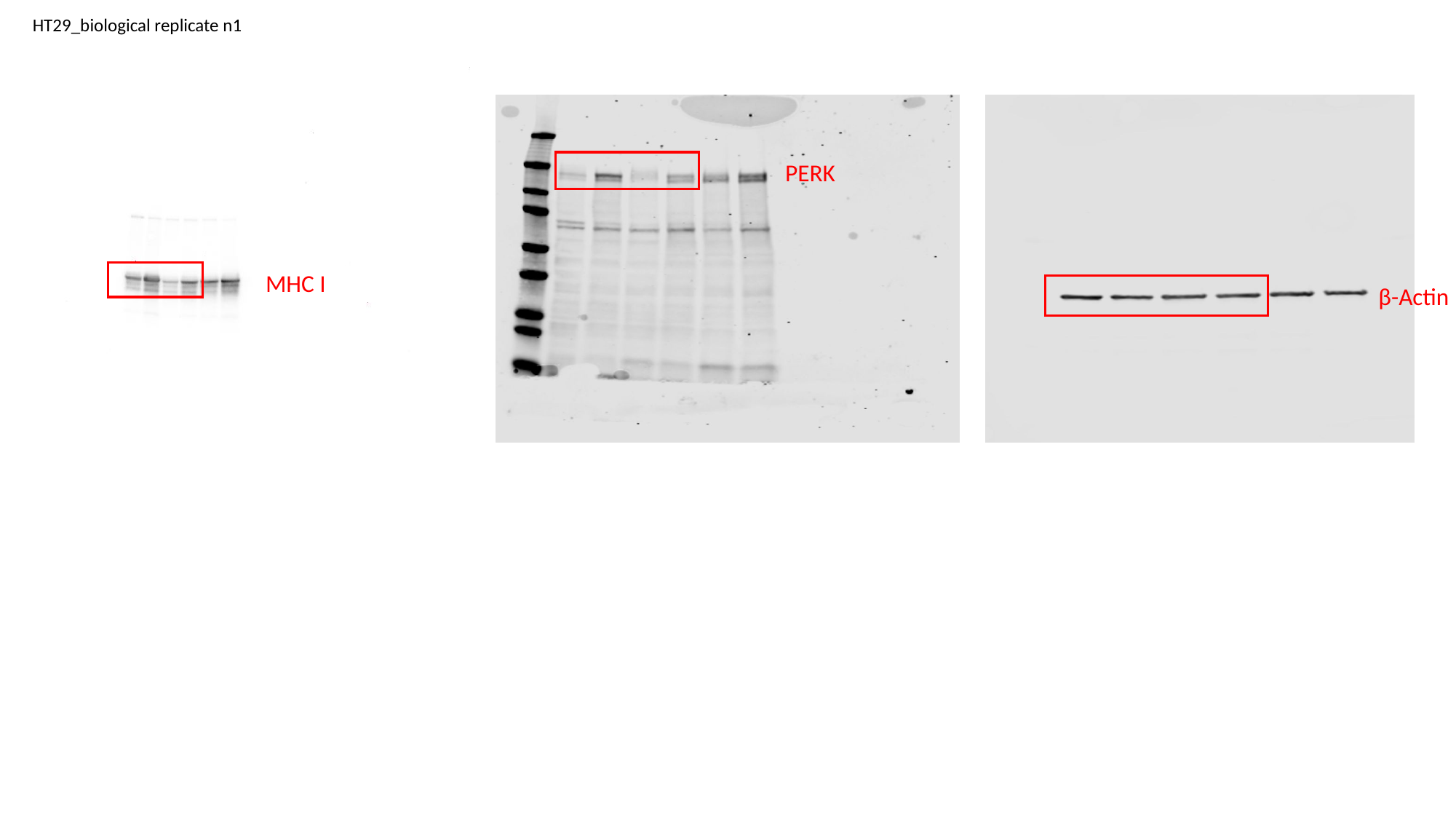

HT29_biological replicate n1
PERK
MHC I
β-Actin

## Slide 2
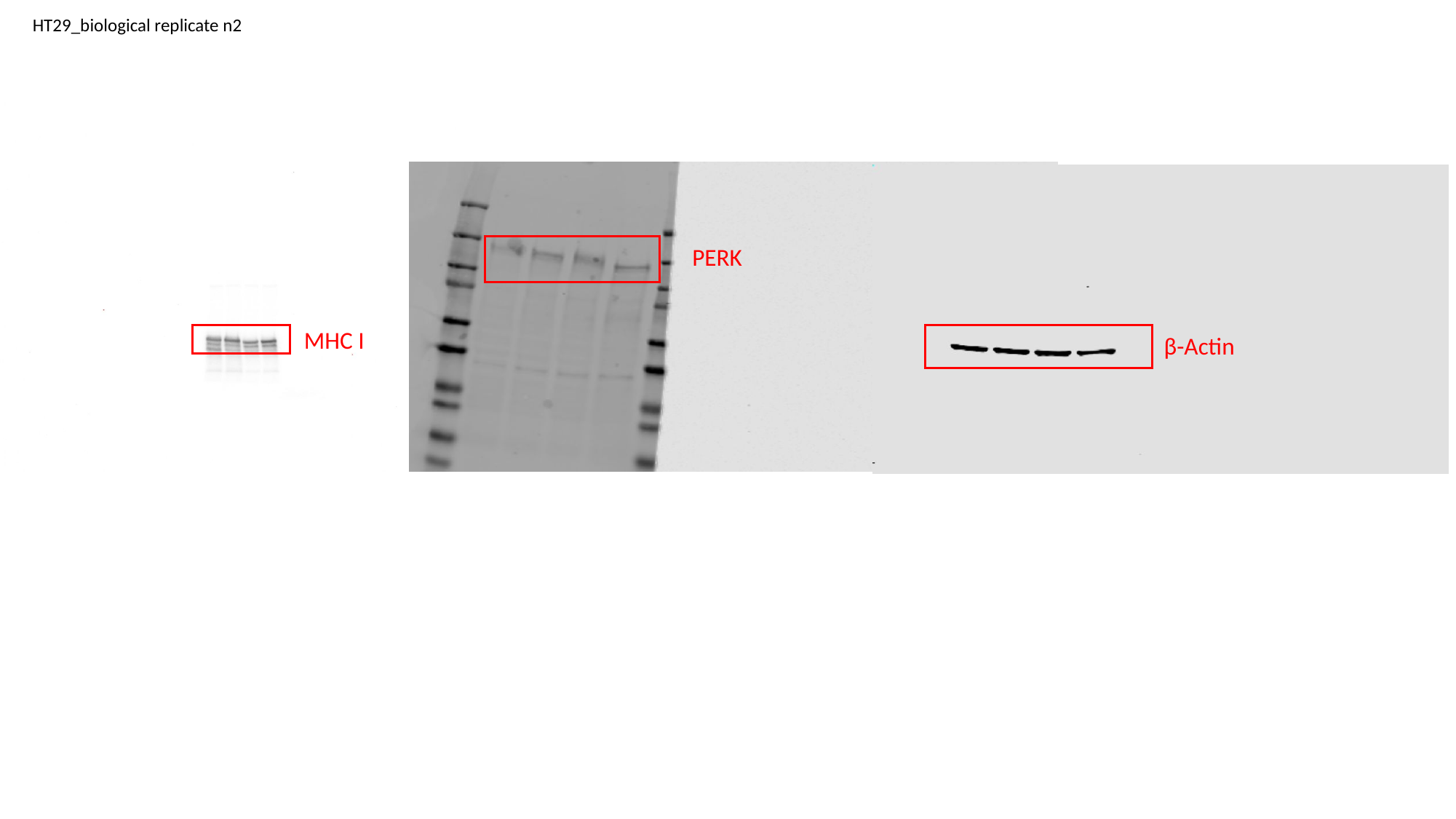

HT29_biological replicate n2
MHC I
PERK
PERK
β-Actin

## Slide 3
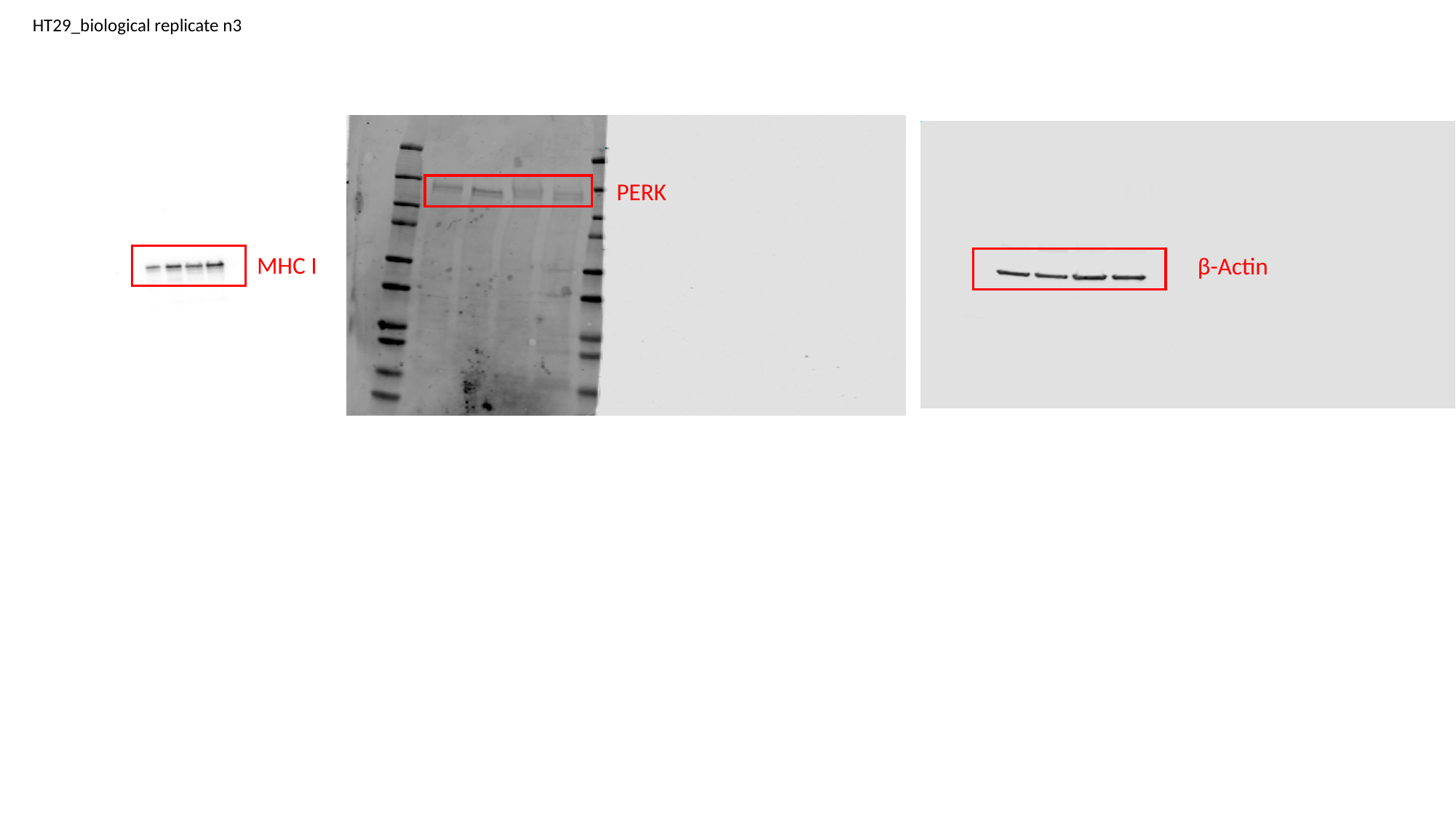

HT29_biological replicate n3
PERK
MHC I
β-Actin
