# Supplementary material for: Hypoxia promotes tumor immune evasion by suppressing MHC-I expression and antigen presentation
Source: EMBO J. 2025 Jan 3;44(3):903–22. doi: 10.1038/s44318-024-00319-7 (PMC11790895; doi:10.1038/s44318-024-00319-7)
Supplement: Supplementary file 6 — Source data Fig. 4 [file 44318_2024_319_MOESM6_ESM.zip › EMBOJ-2024-117498-T-SourceDataForFigure4A-H/Figure 4 B/README/HT29_western_all biological replicates .pptx]

## Slide 1
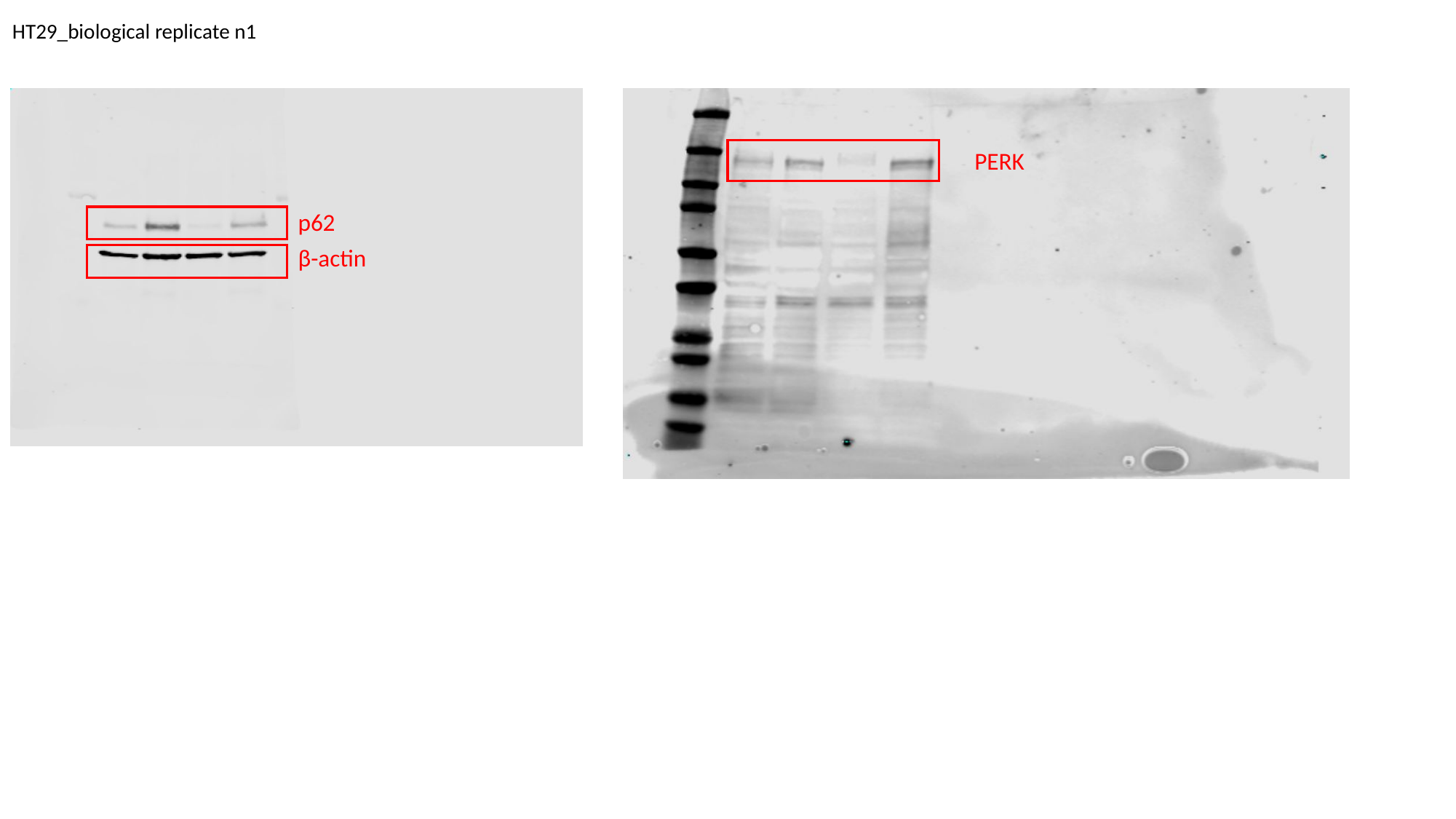

HT29_biological replicate n1
PERK
p62
β-actin

## Slide 2
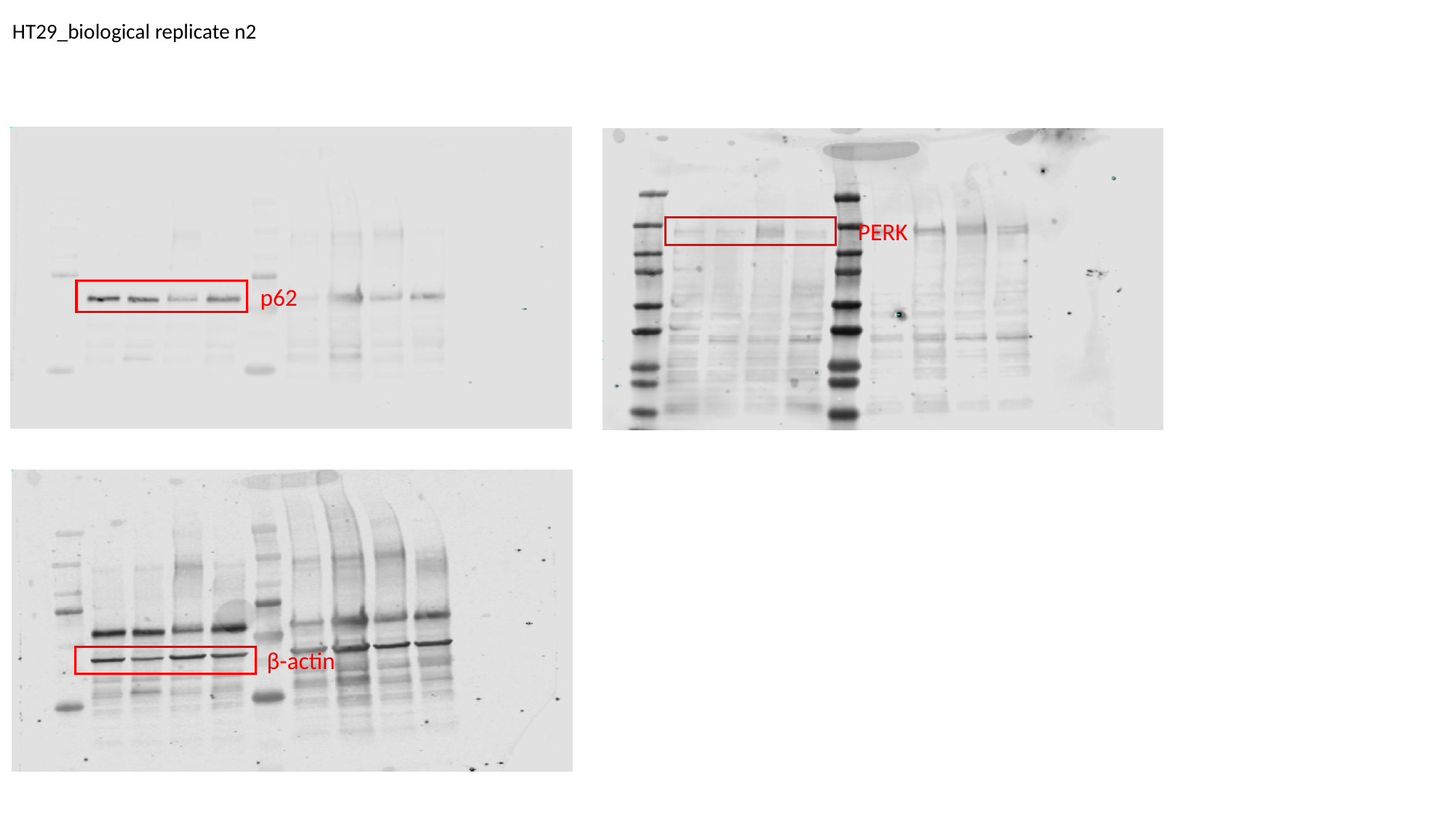

HT29_biological replicate n2
PERK
p62
β-actin

## Slide 3
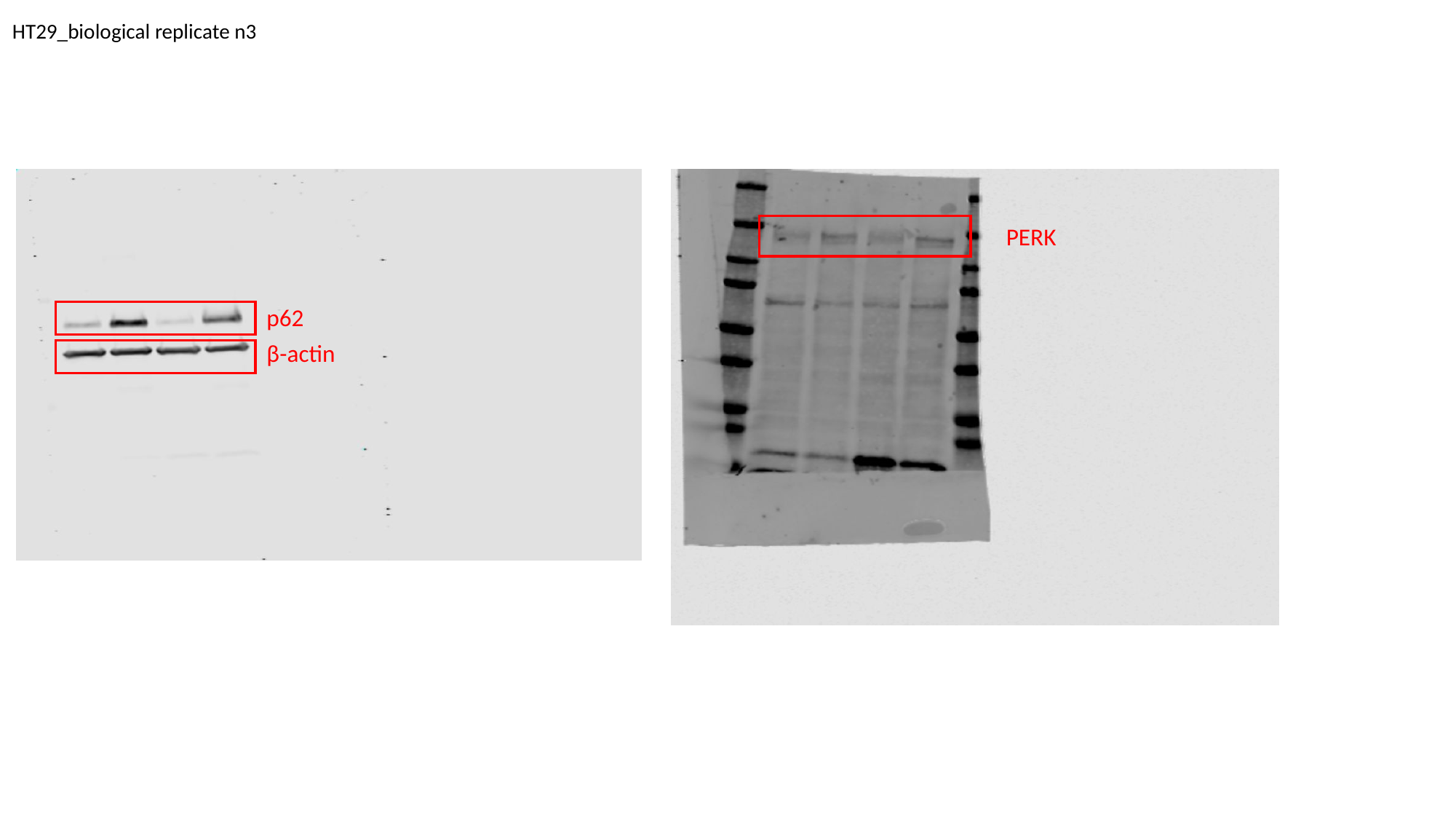

HT29_biological replicate n3
PERK
p62
β-actin
