# Supplementary material for: Hypoxia promotes tumor immune evasion by suppressing MHC-I expression and antigen presentation
Source: EMBO J. 2025 Jan 3;44(3):903–22. doi: 10.1038/s44318-024-00319-7 (PMC11790895; doi:10.1038/s44318-024-00319-7)
Supplement: Supplementary file 6 — Source data Fig. 4 [file 44318_2024_319_MOESM6_ESM.zip › EMBOJ-2024-117498-T-SourceDataForFigure4A-H/Figure 4 H/README/HT29_western_biological replicates.pptx]

## Slide 1
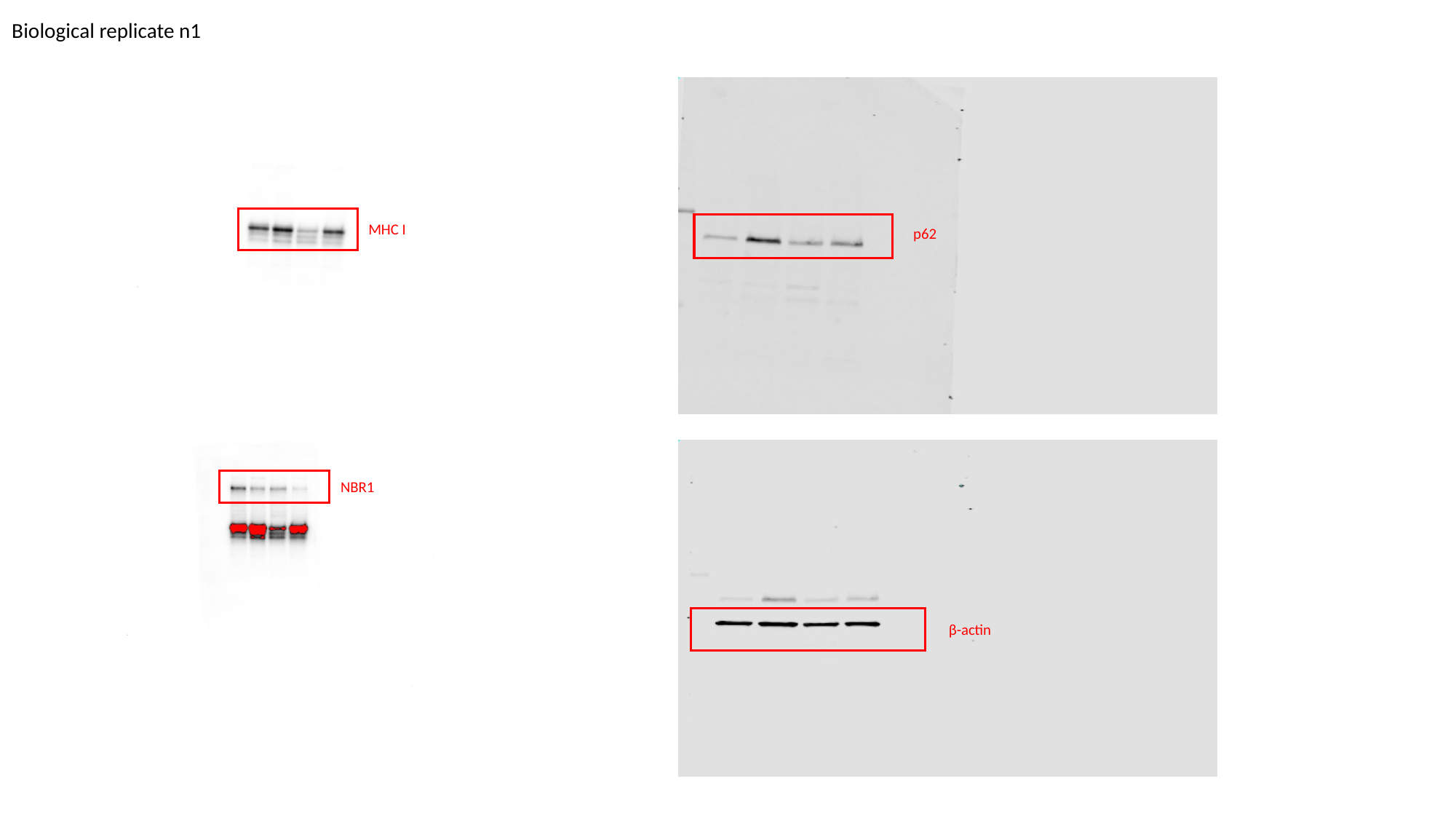

Biological replicate n1
MHC I
p62
NBR1
β-actin

## Slide 2
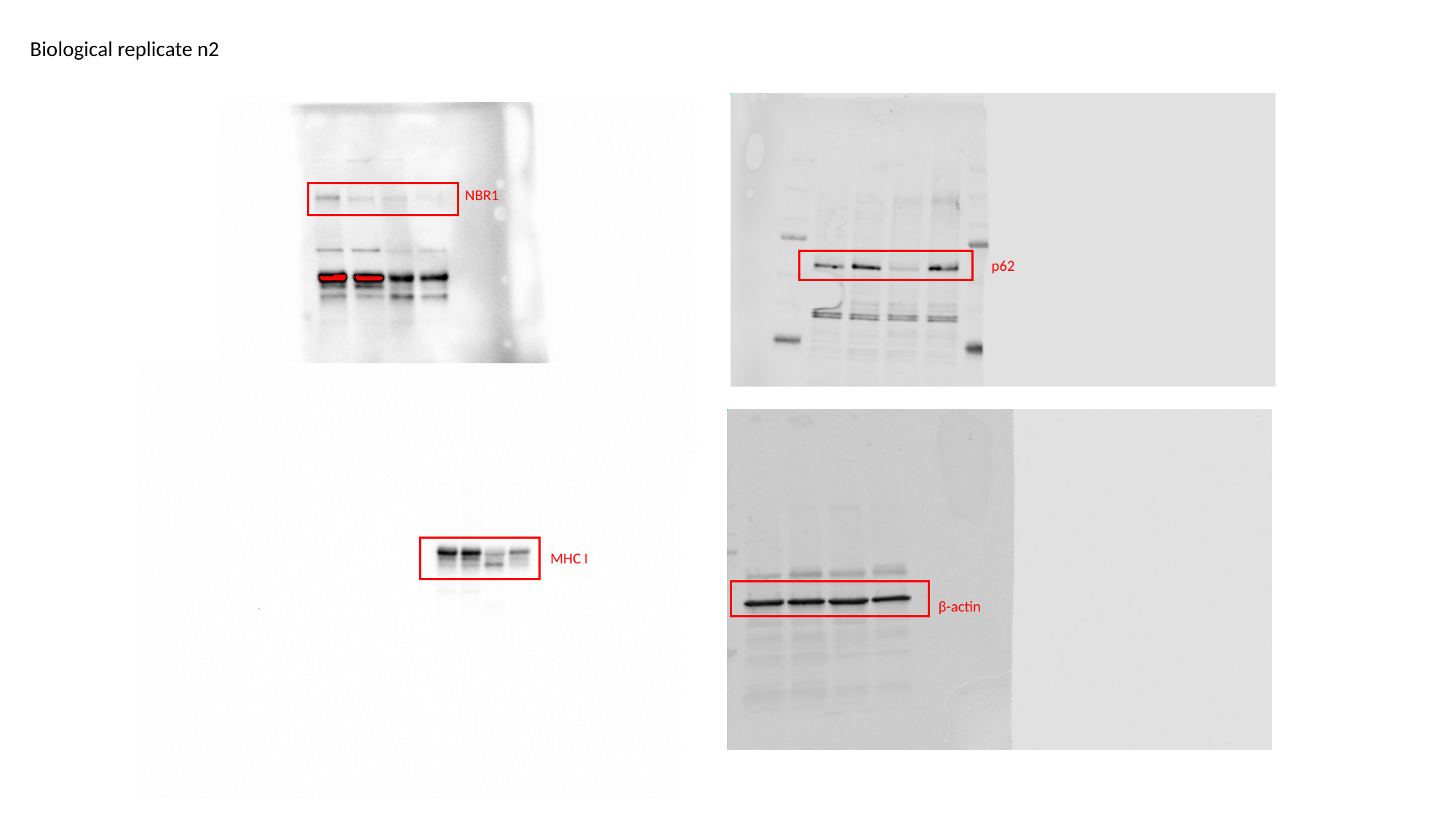

Biological replicate n2
NBR1
p62
MHC I
β-actin

## Slide 3
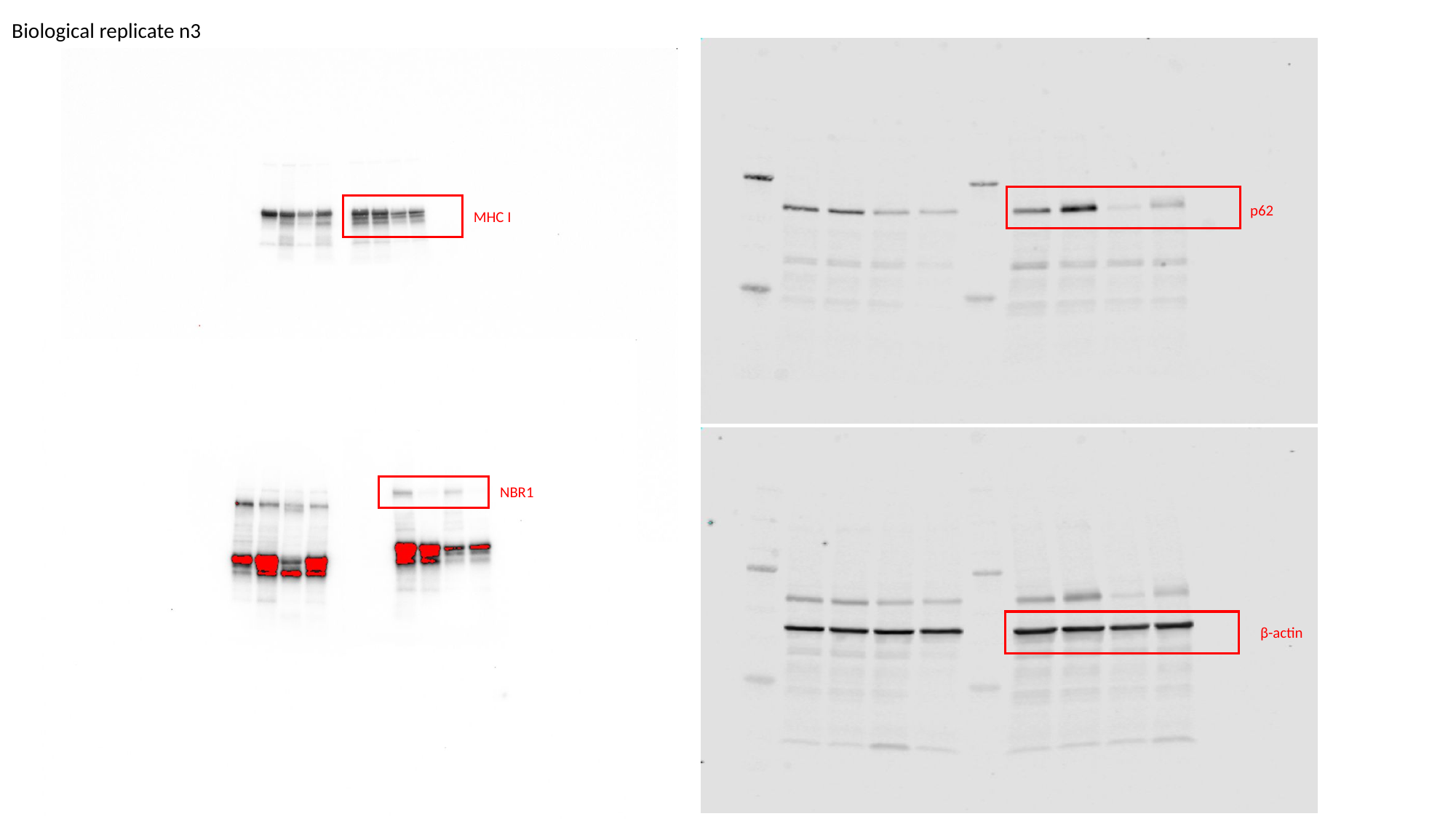

Biological replicate n3
p62
MHC I
NBR1
β-actin
