# Supplementary material for: Hypoxia promotes tumor immune evasion by suppressing MHC-I expression and antigen presentation
Source: EMBO J. 2025 Jan 3;44(3):903–22. doi: 10.1038/s44318-024-00319-7 (PMC11790895; doi:10.1038/s44318-024-00319-7)
Supplement: Supplementary file 7 — Source data Fig. 5 [file 44318_2024_319_MOESM7_ESM.zip › EMBOJ-2024-117498-T-SourceDataForFigure5B-H/Figure 5 F/README/HT29_western_all biological repeats.pptx]

## Slide 1
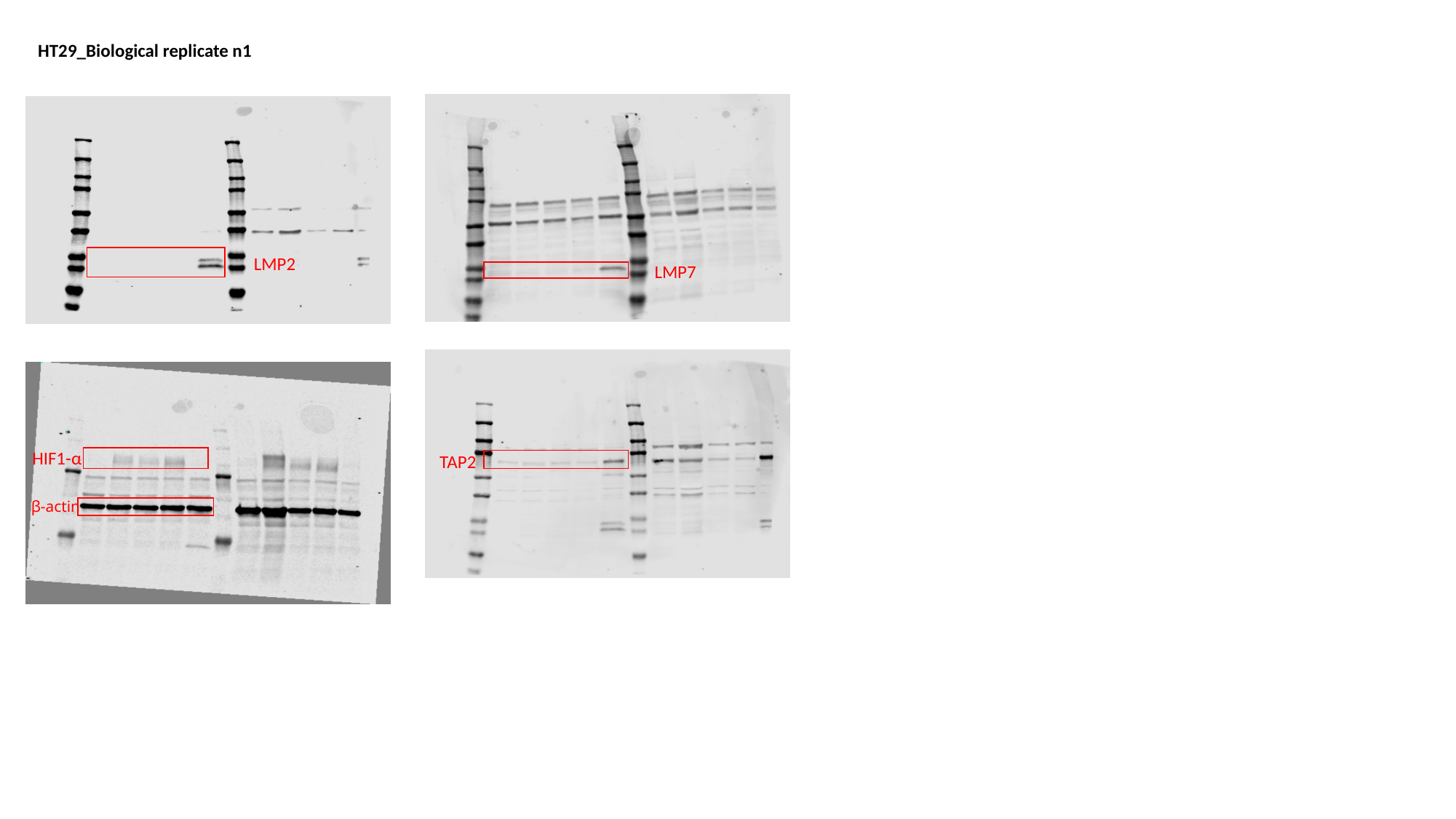

HT29_Biological replicate n1
LMP2
LMP7
HIF1-α
TAP2
β-actin

## Slide 2
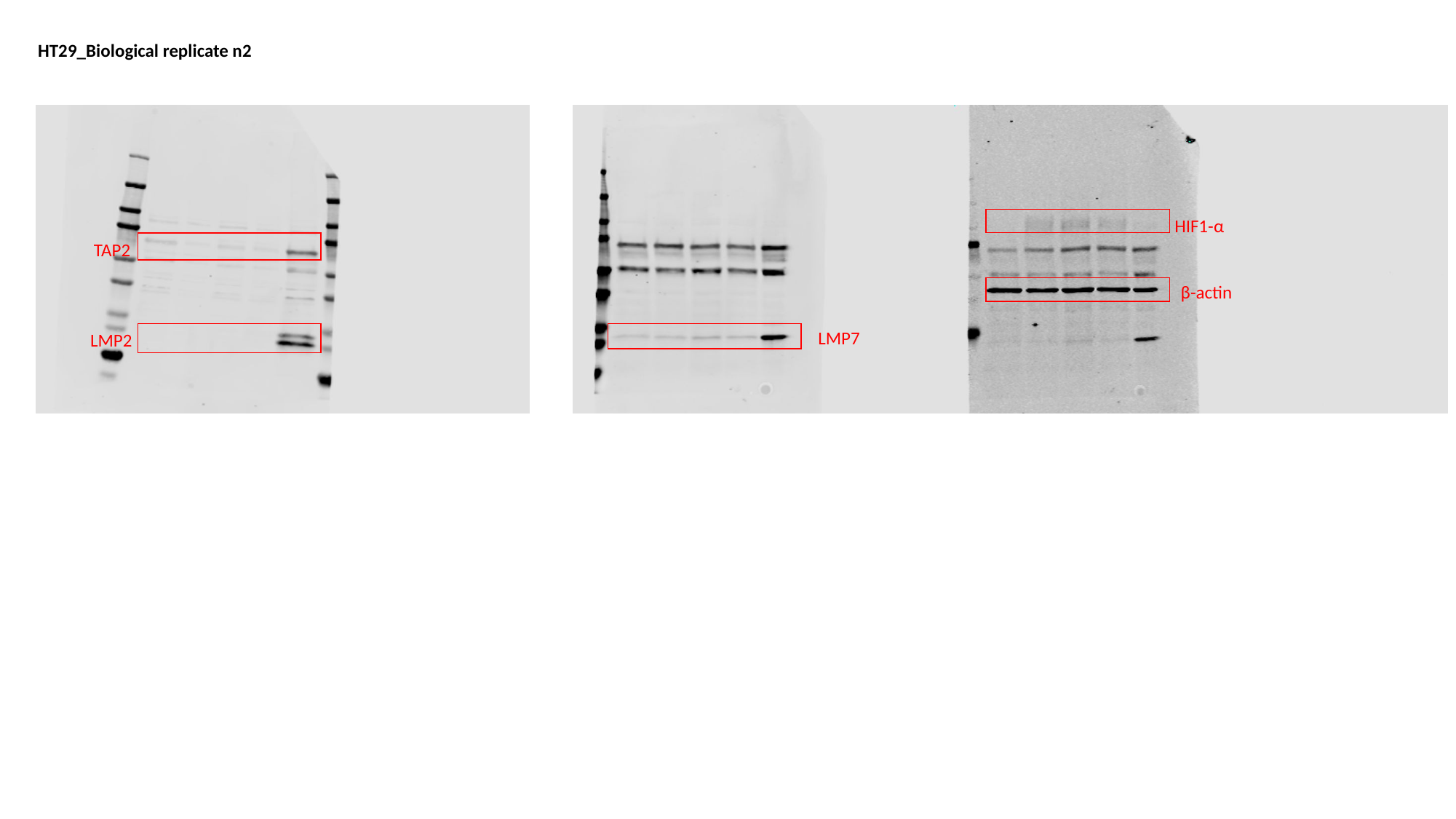

HT29_Biological replicate n2
TAP2
LMP2
HIF1-α
β-actin
LMP7

## Slide 3
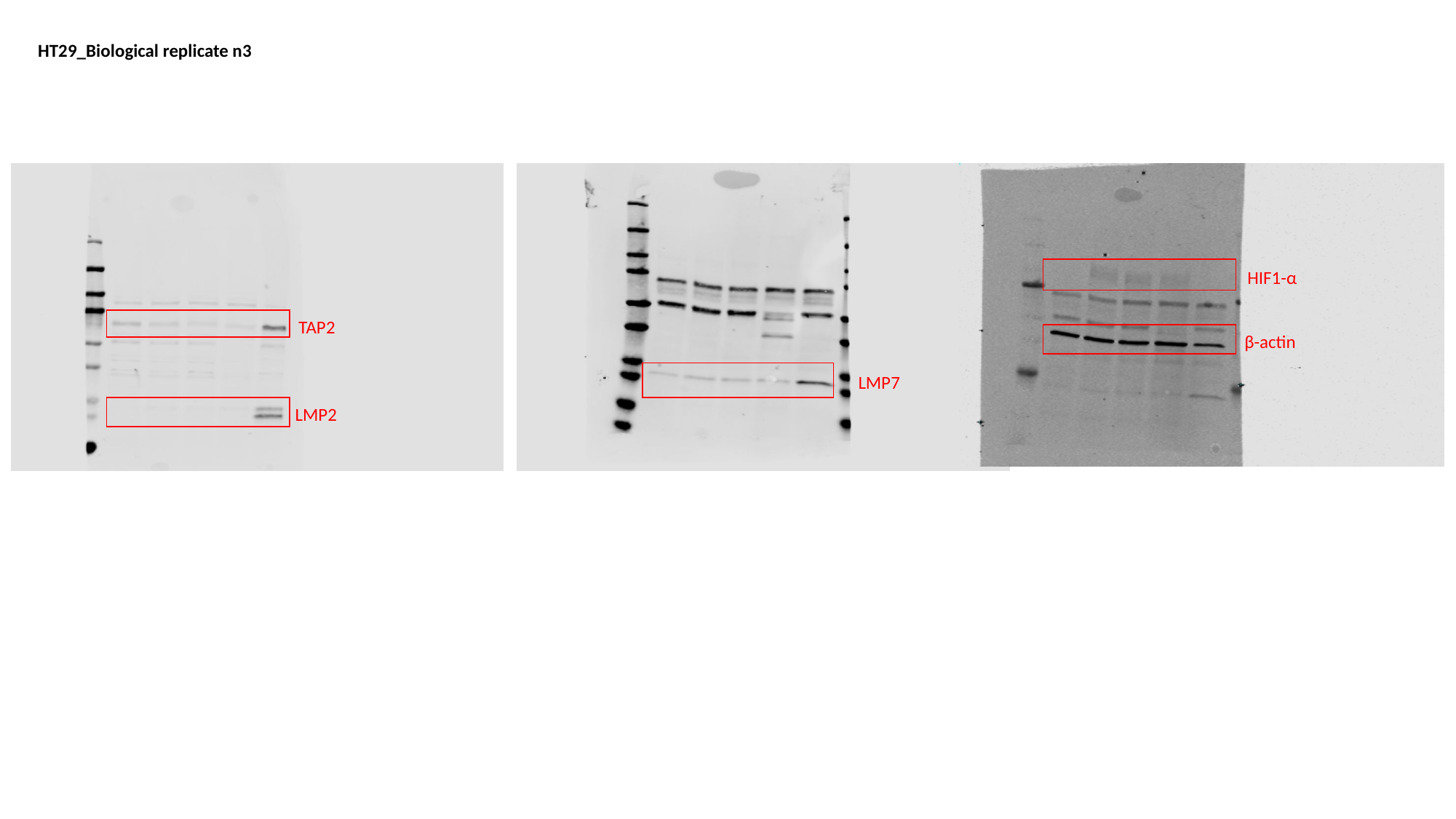

HT29_Biological replicate n3
HIF1-α
TAP2
β-actin
LMP7
LMP2
