# Supplementary figures and images for: Hypoxia promotes tumor immune evasion by suppressing MHC-I expression and antigen presentation (part 2 of 2)
Source: EMBO J. 2025 Jan 3;44(3):903–22. doi: 10.1038/s44318-024-00319-7 (PMC11790895; doi:10.1038/s44318-024-00319-7)

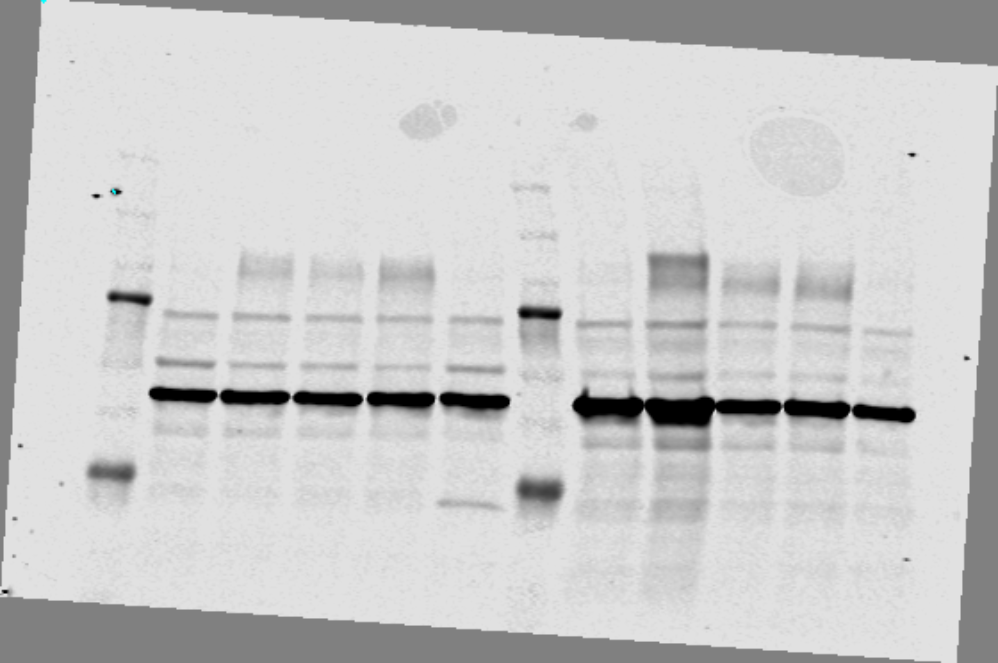

Supplement: Supplementary file 7 — Source data Fig. 5 [file 44318_2024_319_MOESM7_ESM.zip › EMBOJ-2024-117498-T-SourceDataForFigure5B-H/Figure 5 F/HT29_Biological replicate_n1_western/HT29_HIF1 actin_Western1.tif]

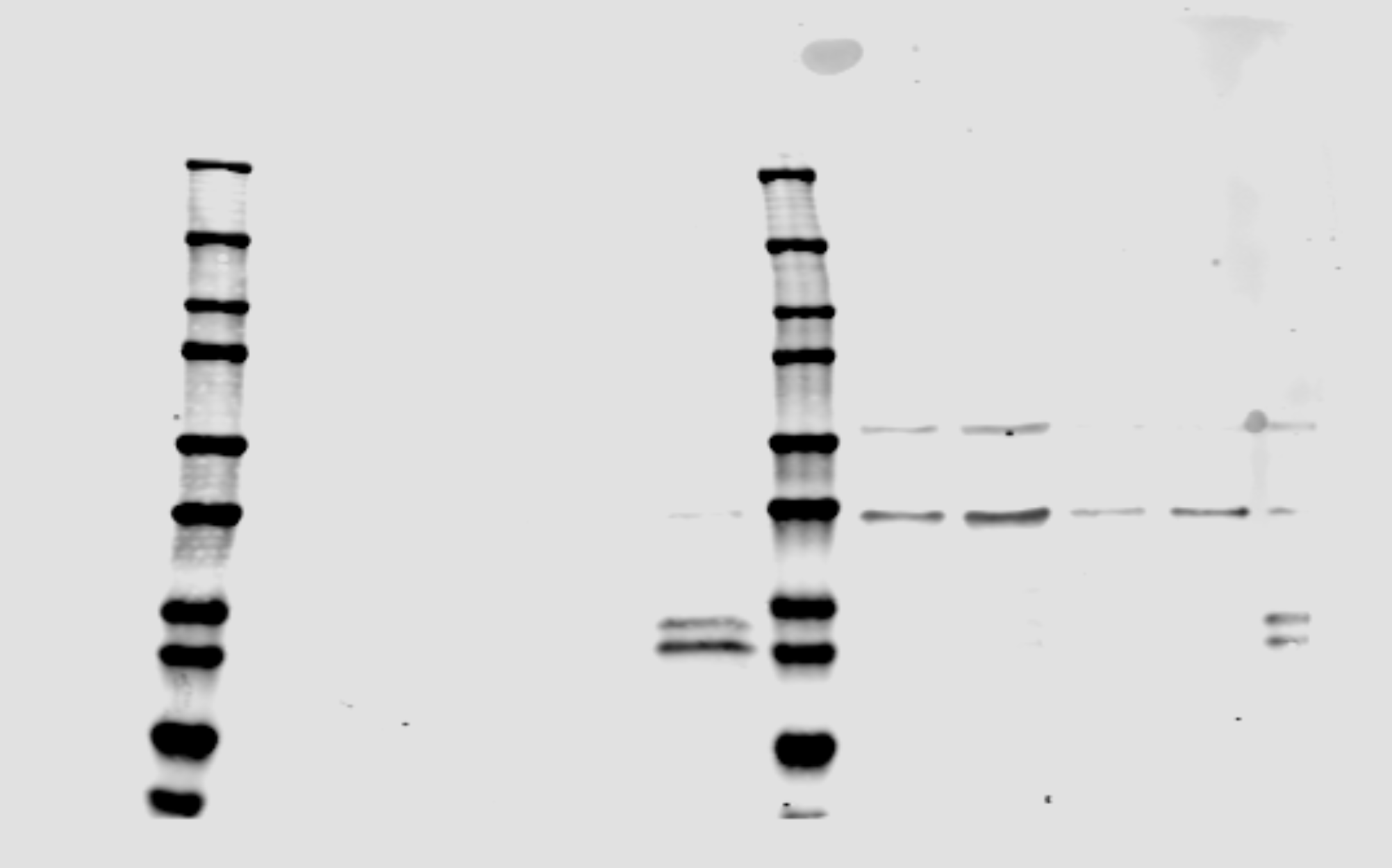

Supplement: Supplementary file 7 — Source data Fig. 5 [file 44318_2024_319_MOESM7_ESM.zip › EMBOJ-2024-117498-T-SourceDataForFigure5B-H/Figure 5 F/HT29_Biological replicate_n1_western/HT29_LMP2_Western.tif]

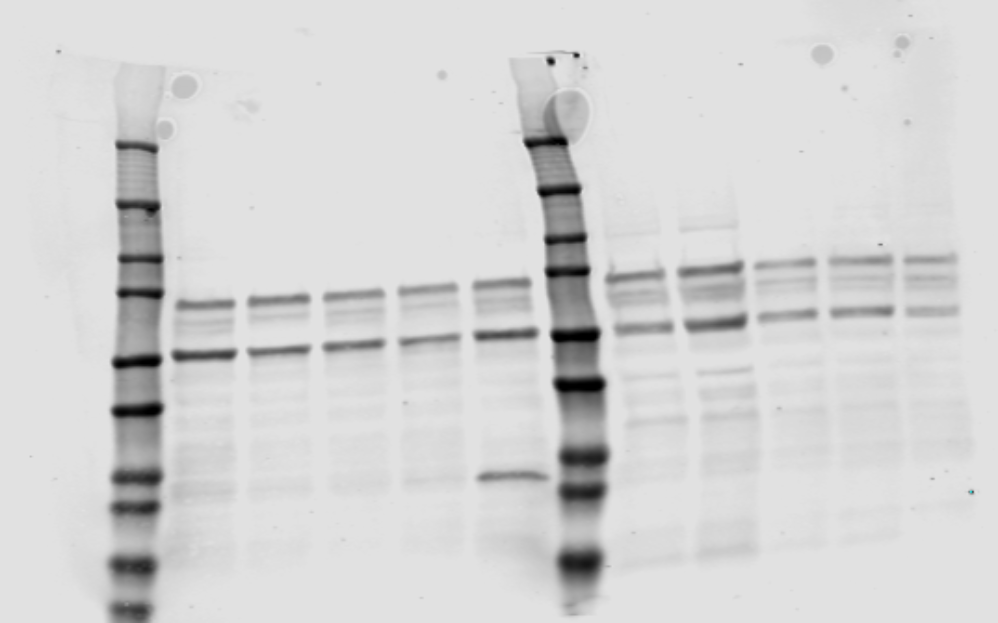

Supplement: Supplementary file 7 — Source data Fig. 5 [file 44318_2024_319_MOESM7_ESM.zip › EMBOJ-2024-117498-T-SourceDataForFigure5B-H/Figure 5 F/HT29_Biological replicate_n1_western/HT29_LMP7_Western.tif]

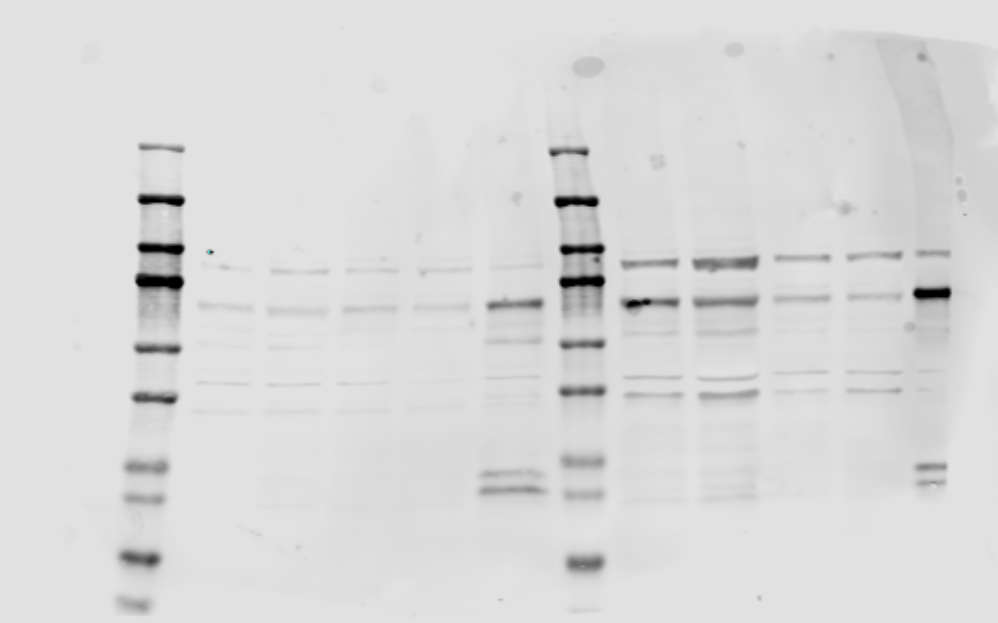

Supplement: Supplementary file 7 — Source data Fig. 5 [file 44318_2024_319_MOESM7_ESM.zip › EMBOJ-2024-117498-T-SourceDataForFigure5B-H/Figure 5 F/HT29_Biological replicate_n1_western/HT29_TAP2_Western.tif]

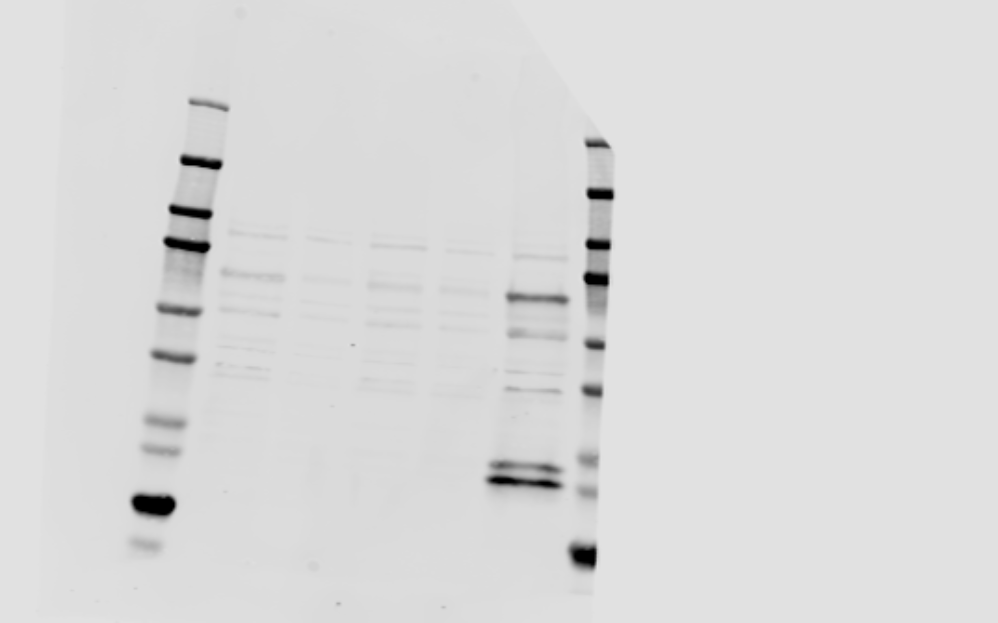

Supplement: Supplementary file 7 — Source data Fig. 5 [file 44318_2024_319_MOESM7_ESM.zip › EMBOJ-2024-117498-T-SourceDataForFigure5B-H/Figure 5 F/HT29_Biological replicate_n2_western/HT29_ LMP2 TAP2_Western.tif]

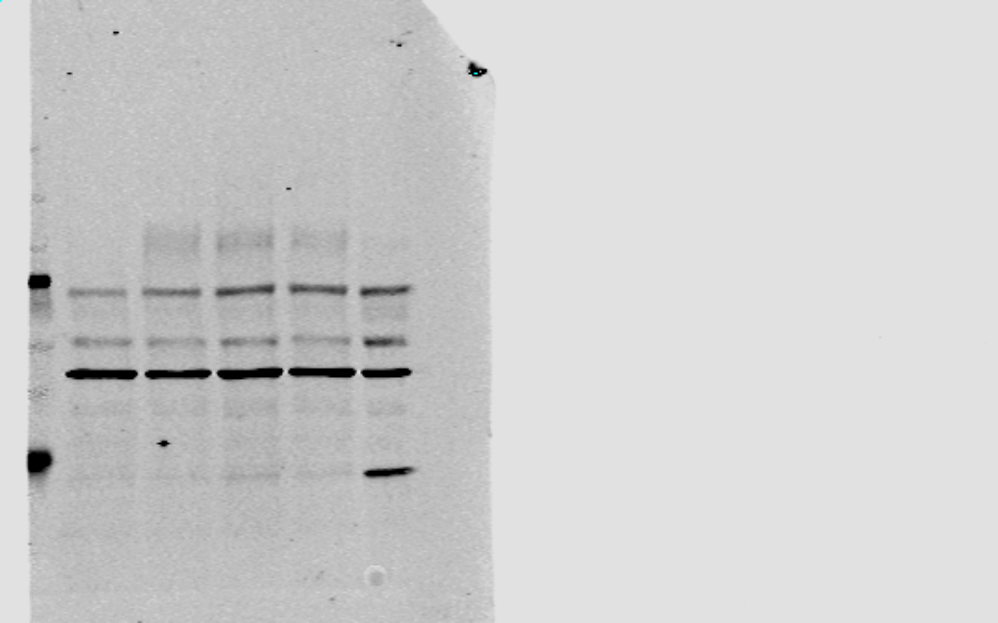

Supplement: Supplementary file 7 — Source data Fig. 5 [file 44318_2024_319_MOESM7_ESM.zip › EMBOJ-2024-117498-T-SourceDataForFigure5B-H/Figure 5 F/HT29_Biological replicate_n2_western/HT29_HIF1 actin_Western.tif]

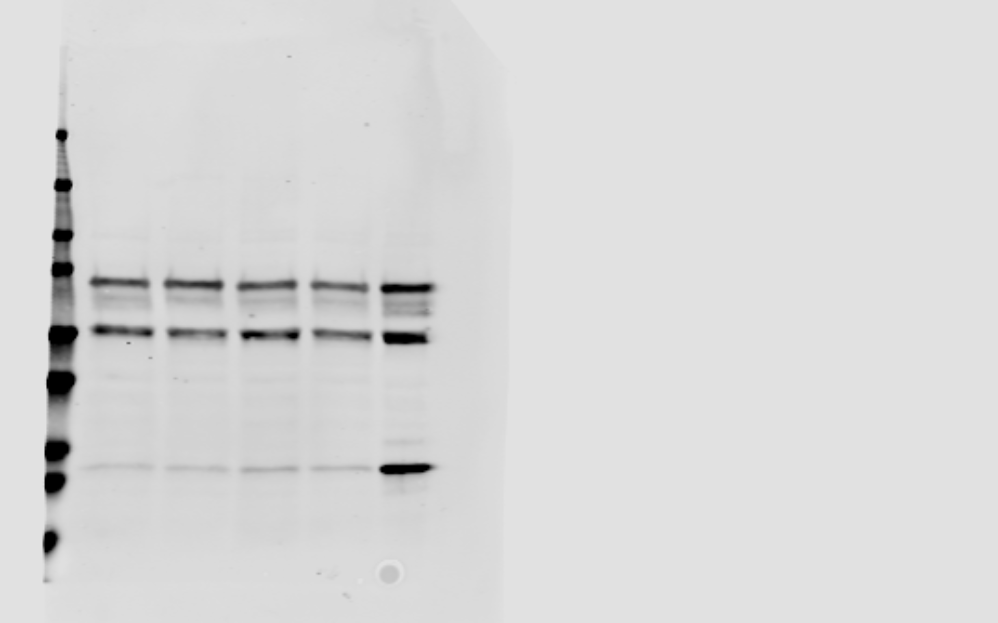

Supplement: Supplementary file 7 — Source data Fig. 5 [file 44318_2024_319_MOESM7_ESM.zip › EMBOJ-2024-117498-T-SourceDataForFigure5B-H/Figure 5 F/HT29_Biological replicate_n2_western/HT29_LMP7_Western.tif]

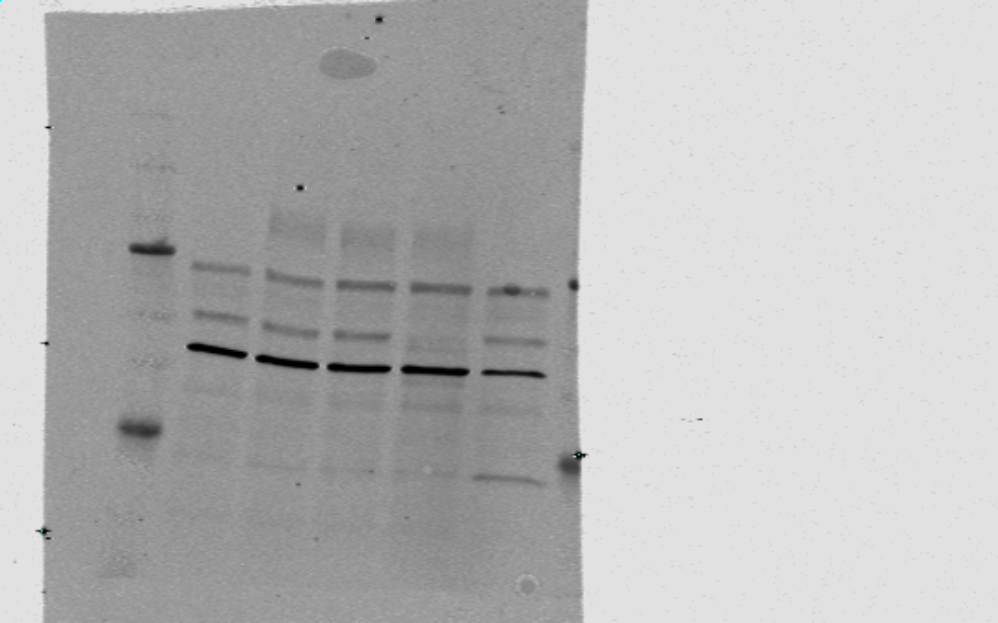

Supplement: Supplementary file 7 — Source data Fig. 5 [file 44318_2024_319_MOESM7_ESM.zip › EMBOJ-2024-117498-T-SourceDataForFigure5B-H/Figure 5 F/HT29_Biological replicate_n3_western/HT29_HIF1 actin_Western.tif]

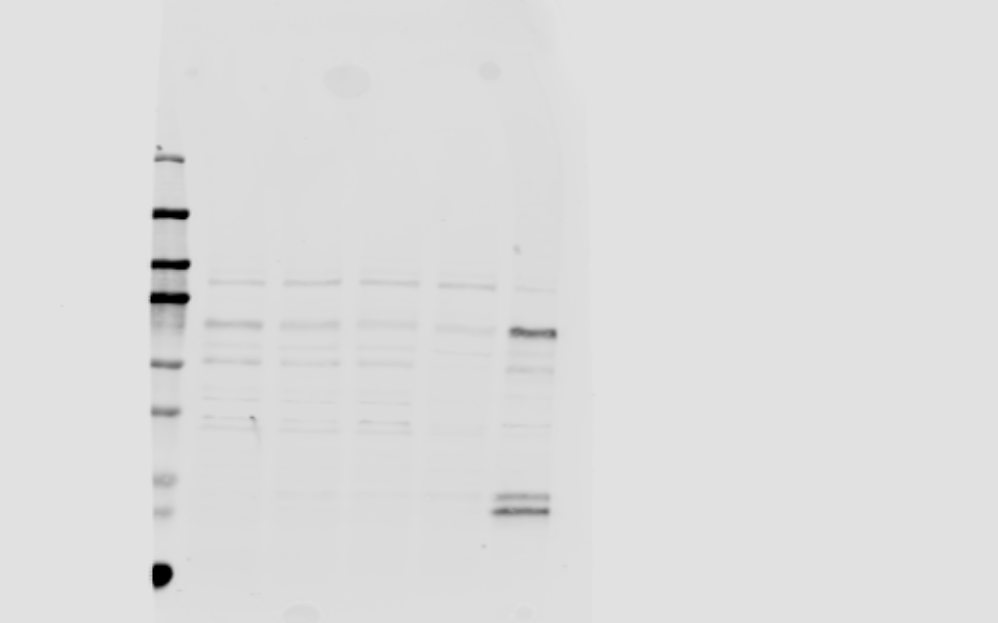

Supplement: Supplementary file 7 — Source data Fig. 5 [file 44318_2024_319_MOESM7_ESM.zip › EMBOJ-2024-117498-T-SourceDataForFigure5B-H/Figure 5 F/HT29_Biological replicate_n3_western/HT29_LMP2 TAP2_Western.png.tif]

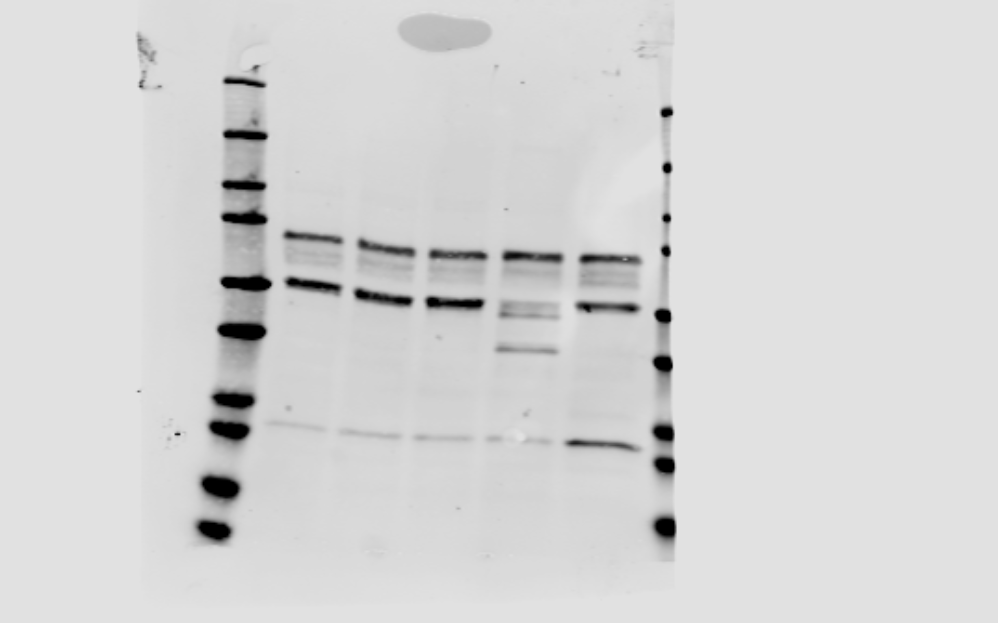

Supplement: Supplementary file 7 — Source data Fig. 5 [file 44318_2024_319_MOESM7_ESM.zip › EMBOJ-2024-117498-T-SourceDataForFigure5B-H/Figure 5 F/HT29_Biological replicate_n3_western/HT29_LMP7_Western.tif]

HT29\_Biological replicate n1

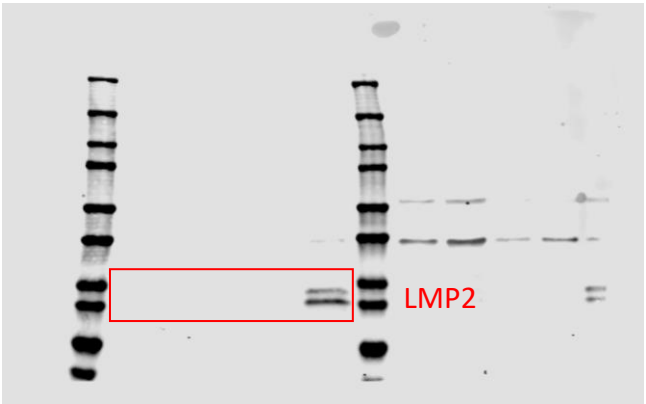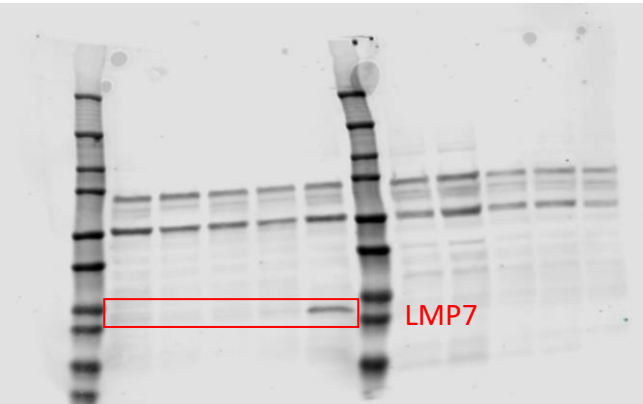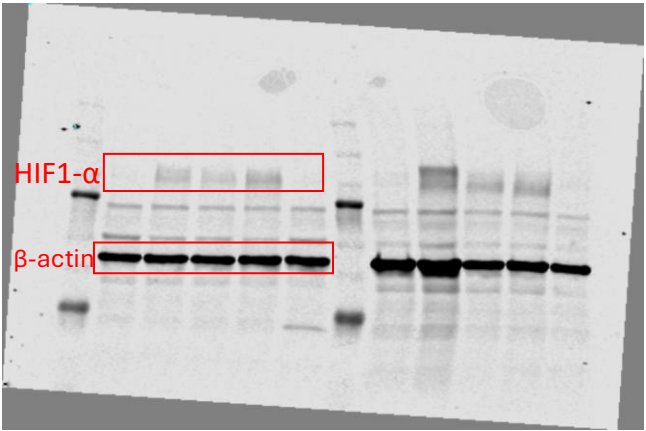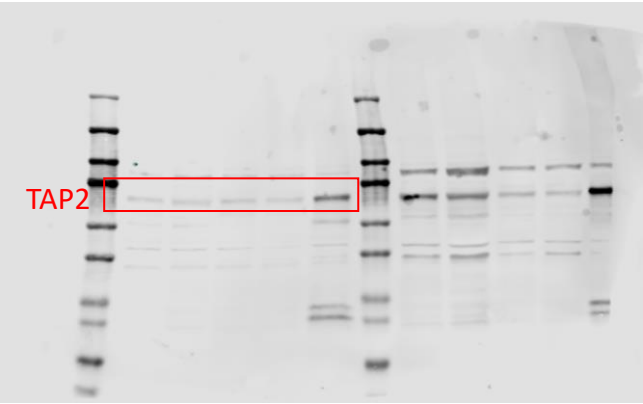

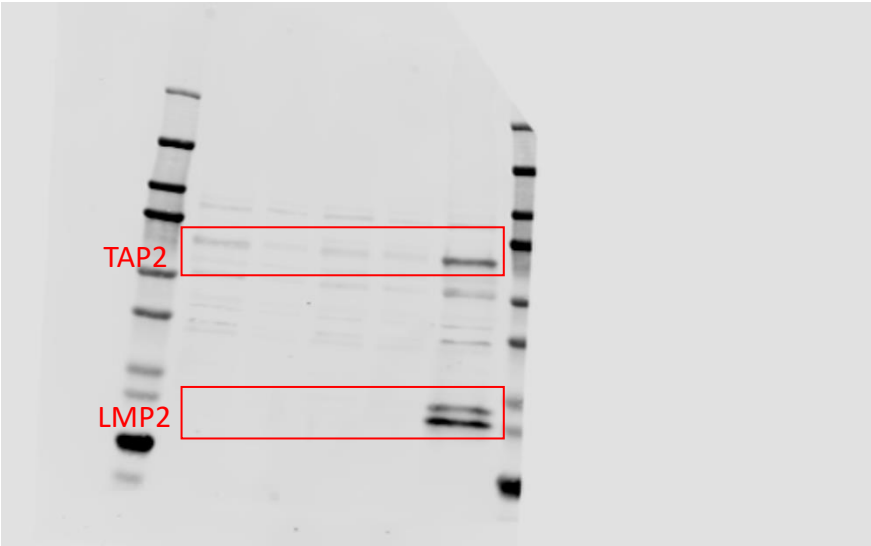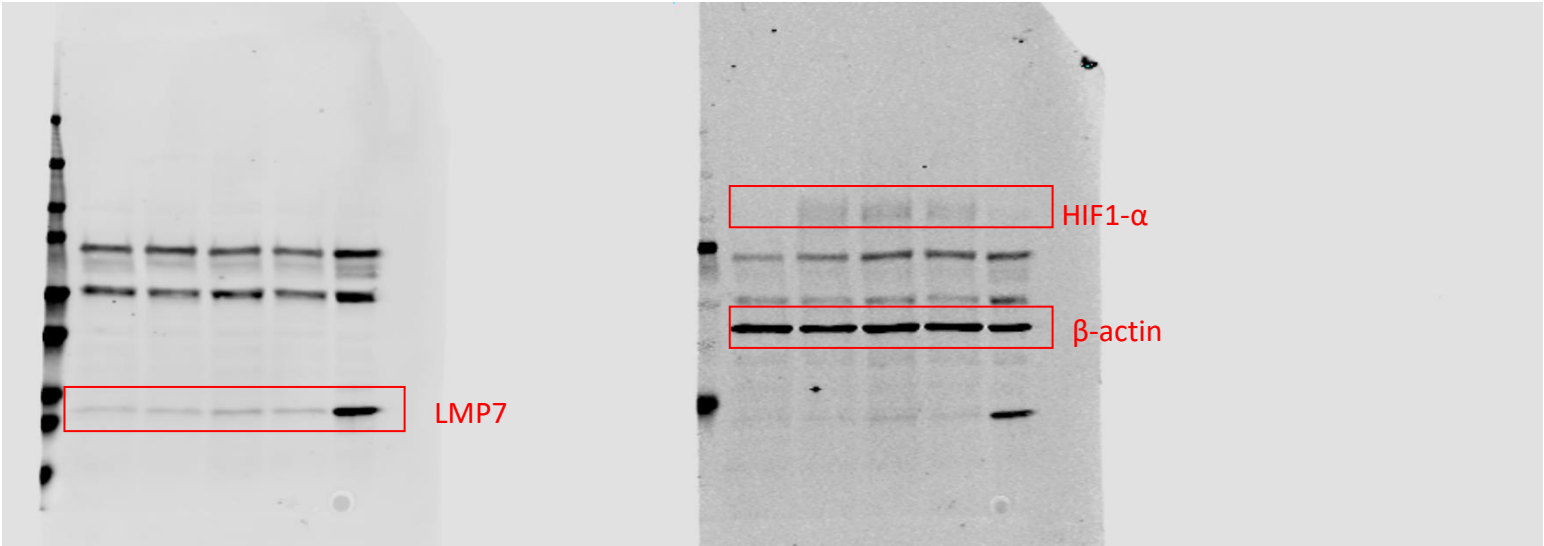

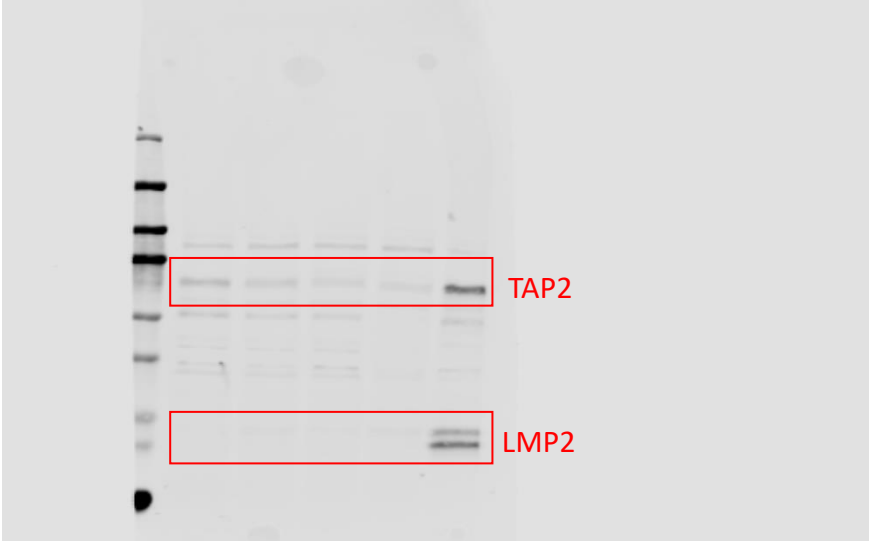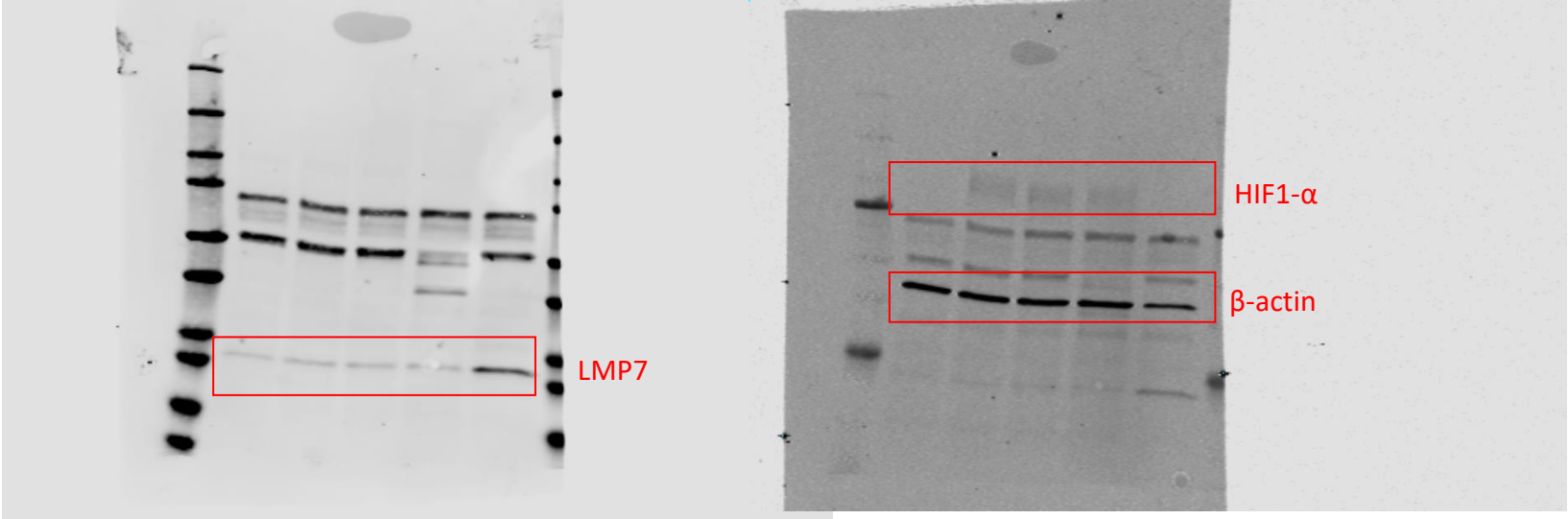

Supplement: Supplementary file 7 — Source data Fig. 5 [file 44318_2024_319_MOESM7_ESM.zip › EMBOJ-2024-117498-T-SourceDataForFigure5B-H/Figure 5 F/README/HT29_western_all biological repeats.pdf]

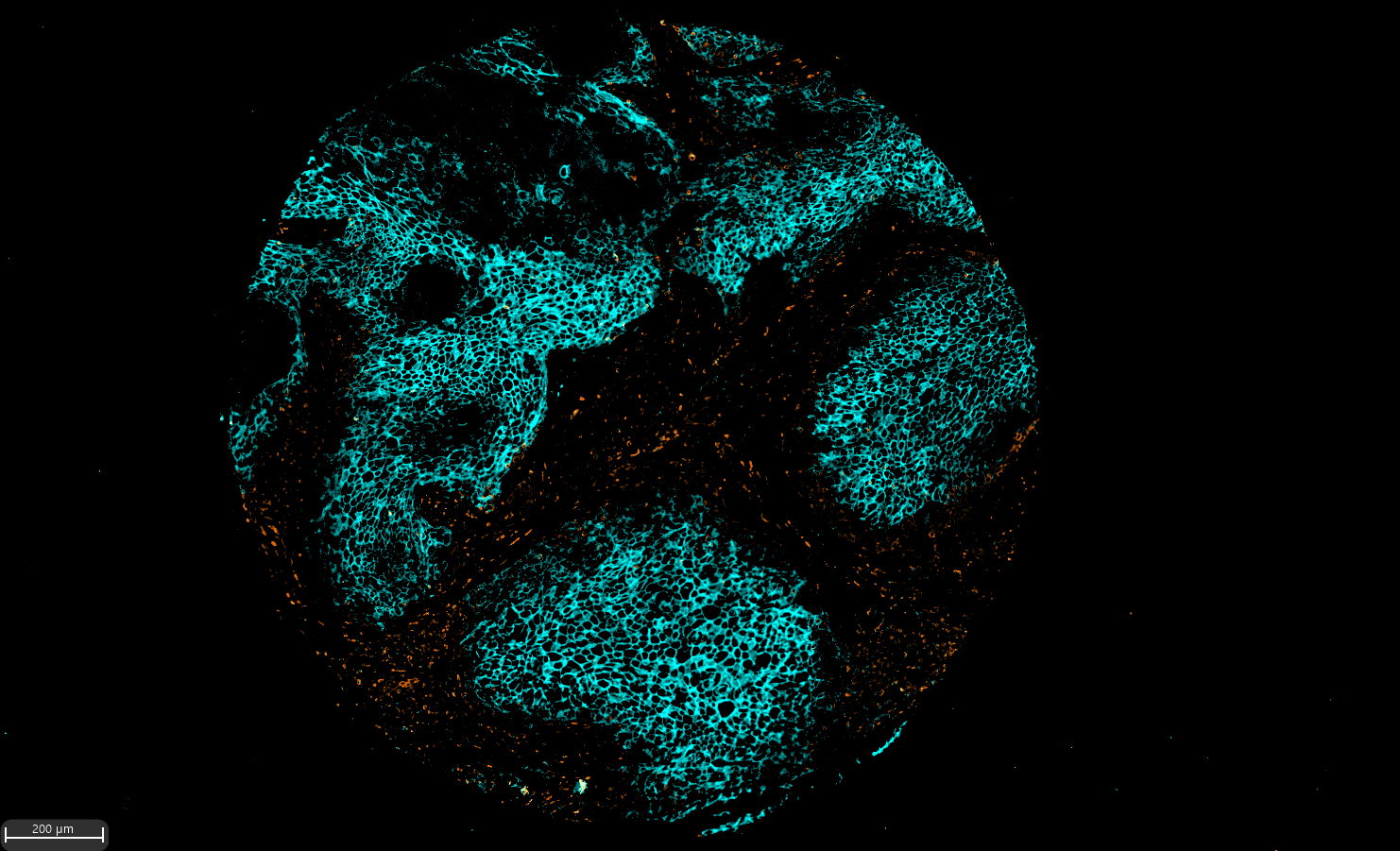

Supplement: Supplementary file 7 — Source data Fig. 5 [file 44318_2024_319_MOESM7_ESM.zip › EMBOJ-2024-117498-T-SourceDataForFigure5B-H/Figure 5 G/Core 1 TAP2 LMP2 CAIX.tiff]

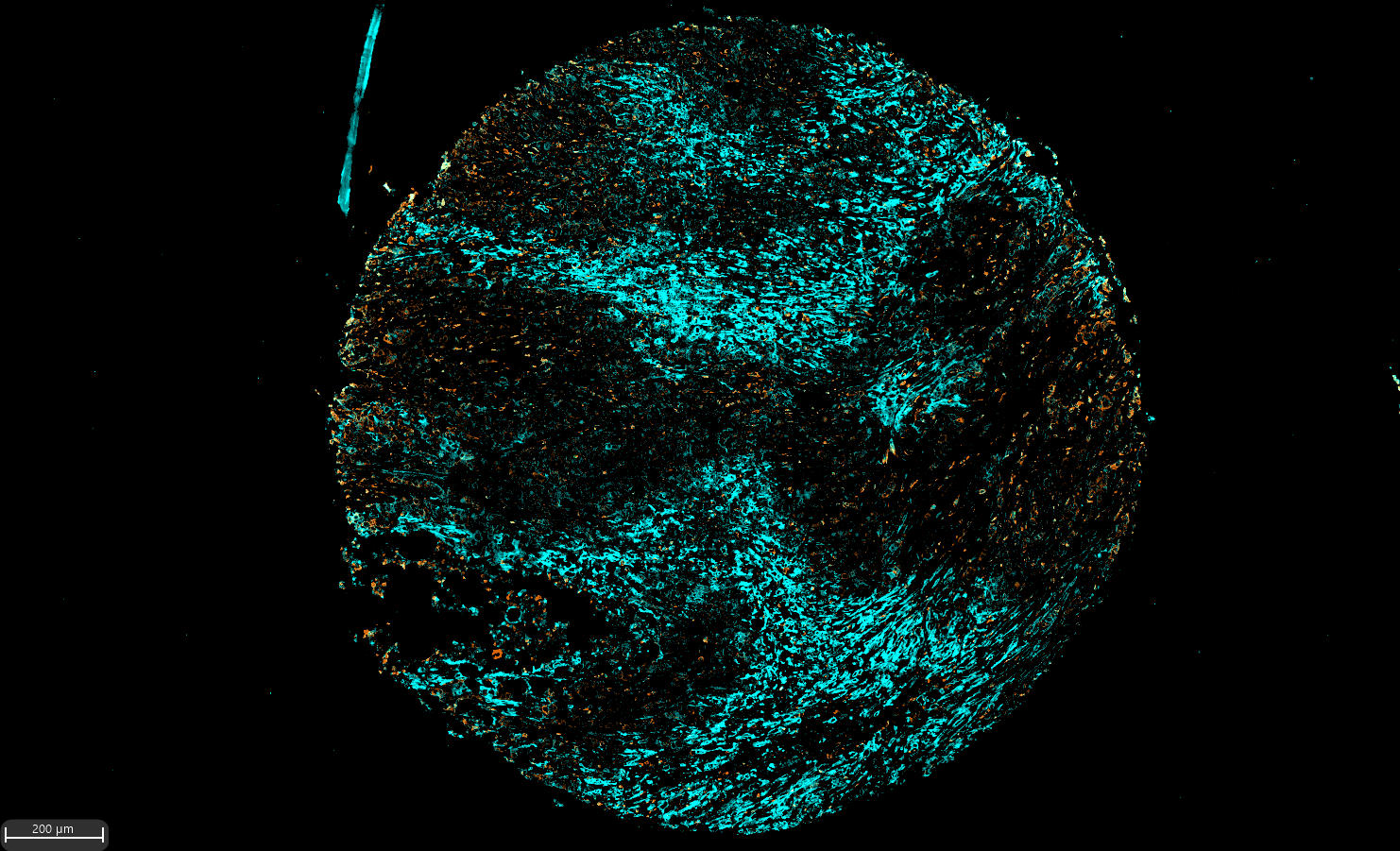

Supplement: Supplementary file 7 — Source data Fig. 5 [file 44318_2024_319_MOESM7_ESM.zip › EMBOJ-2024-117498-T-SourceDataForFigure5B-H/Figure 5 G/Core 2 TAP2 LMP2 CAIX.tiff]

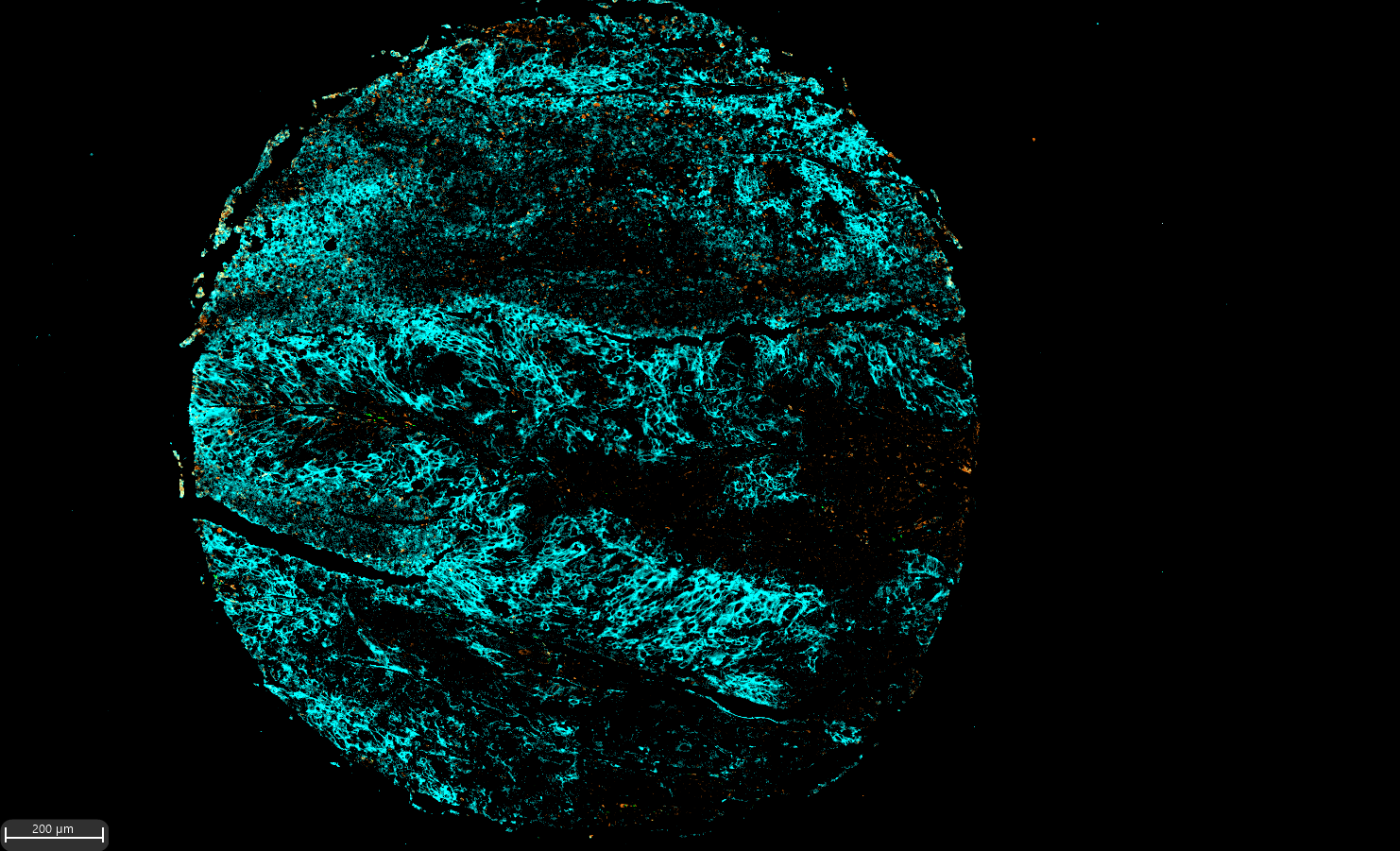

Supplement: Supplementary file 7 — Source data Fig. 5 [file 44318_2024_319_MOESM7_ESM.zip › EMBOJ-2024-117498-T-SourceDataForFigure5B-H/Figure 5 G/Core 3 TAP2 LMP2 CAIX.tiff]

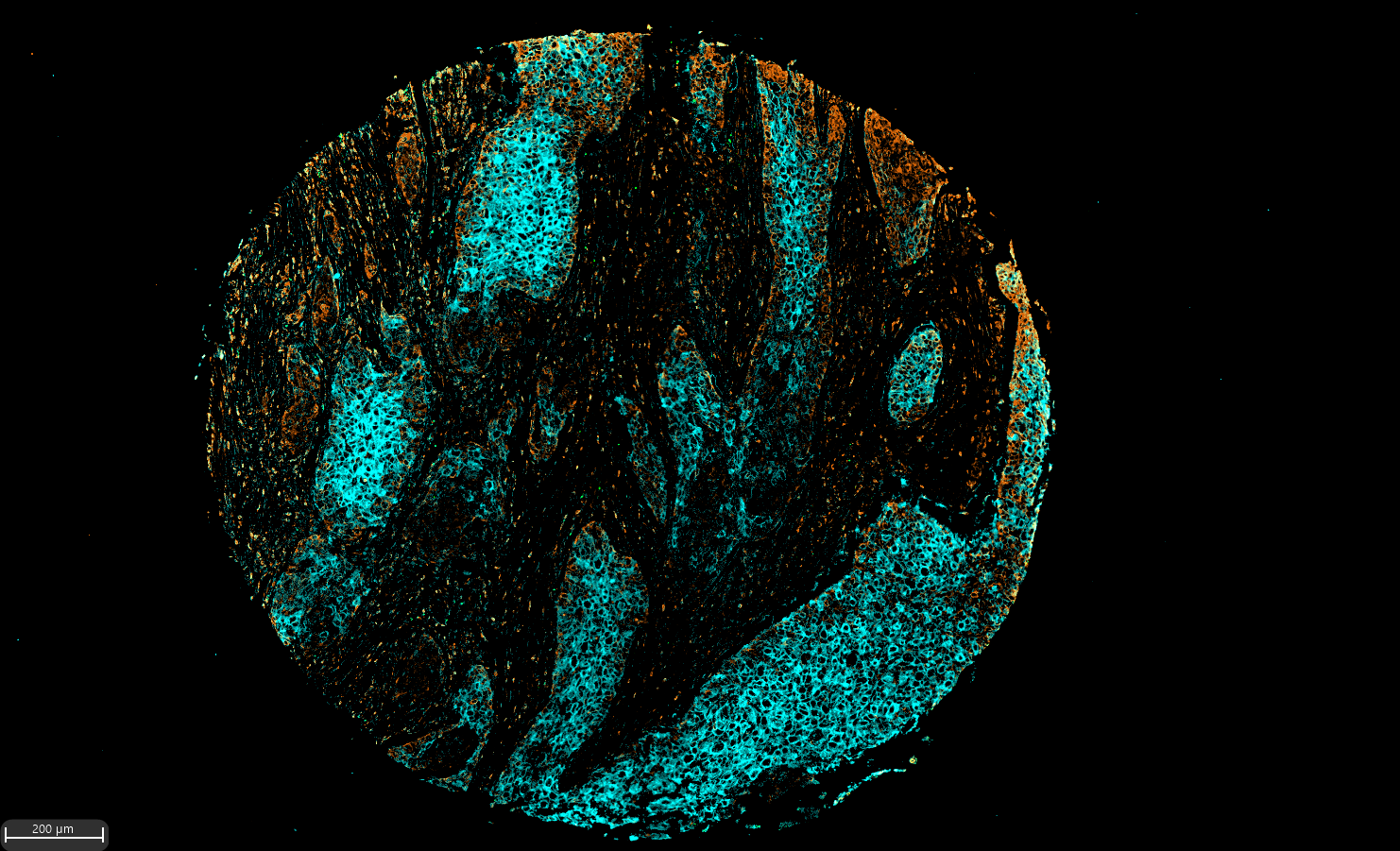

Supplement: Supplementary file 7 — Source data Fig. 5 [file 44318_2024_319_MOESM7_ESM.zip › EMBOJ-2024-117498-T-SourceDataForFigure5B-H/Figure 5 G/Core 4 TAP2 LMP2 CAIX.tiff]

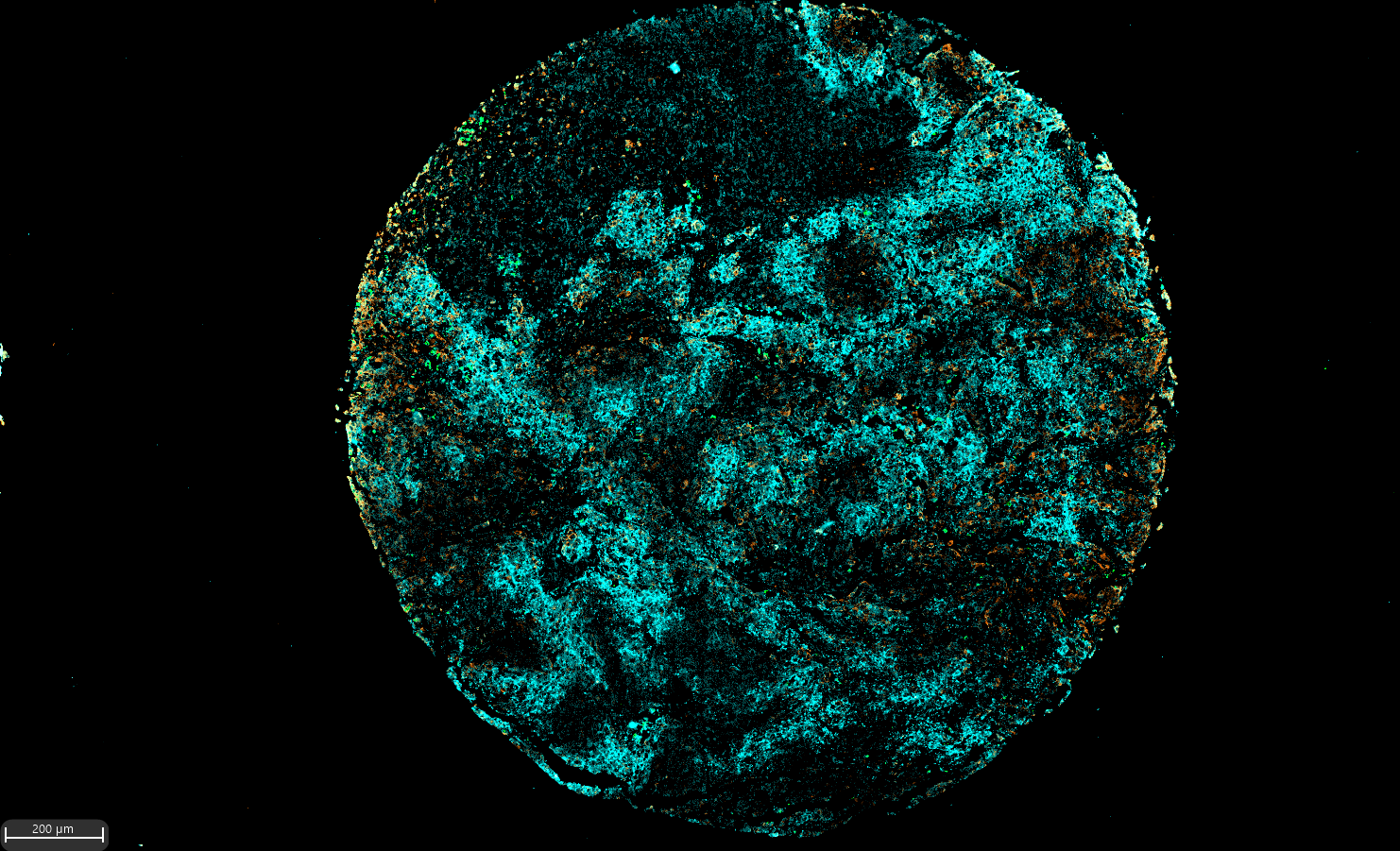

Supplement: Supplementary file 7 — Source data Fig. 5 [file 44318_2024_319_MOESM7_ESM.zip › EMBOJ-2024-117498-T-SourceDataForFigure5B-H/Figure 5 G/Core 5 TAP2 LMP2 CAIX.tiff]

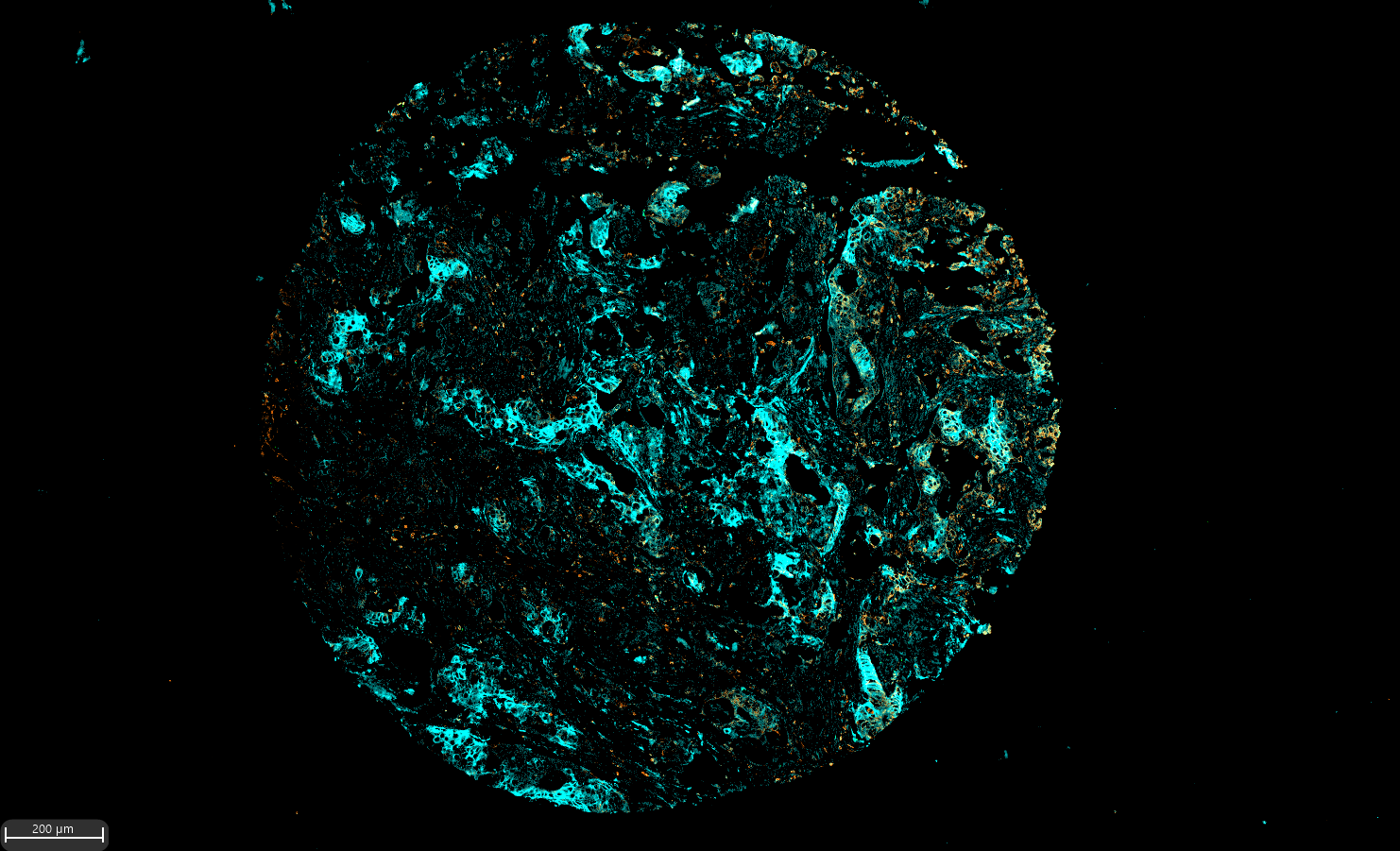

Supplement: Supplementary file 7 — Source data Fig. 5 [file 44318_2024_319_MOESM7_ESM.zip › EMBOJ-2024-117498-T-SourceDataForFigure5B-H/Figure 5 G/Core 6 TAP2 LMP2 CAIX.tiff]

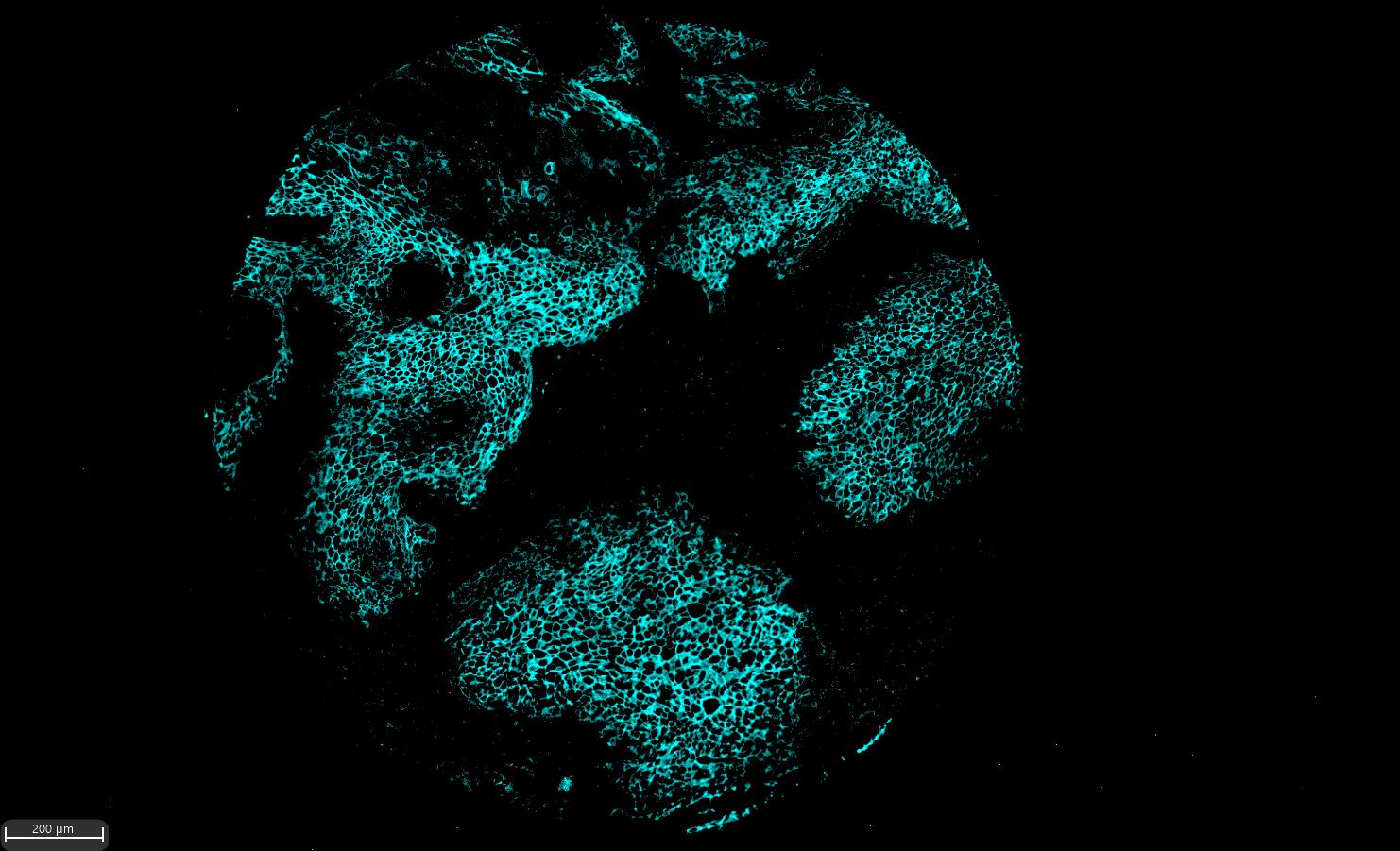

Supplement: Supplementary file 7 — Source data Fig. 5 [file 44318_2024_319_MOESM7_ESM.zip › EMBOJ-2024-117498-T-SourceDataForFigure5B-H/Figure 5 G/README/CAIX staining only/Core 1 CAIX.tiff]

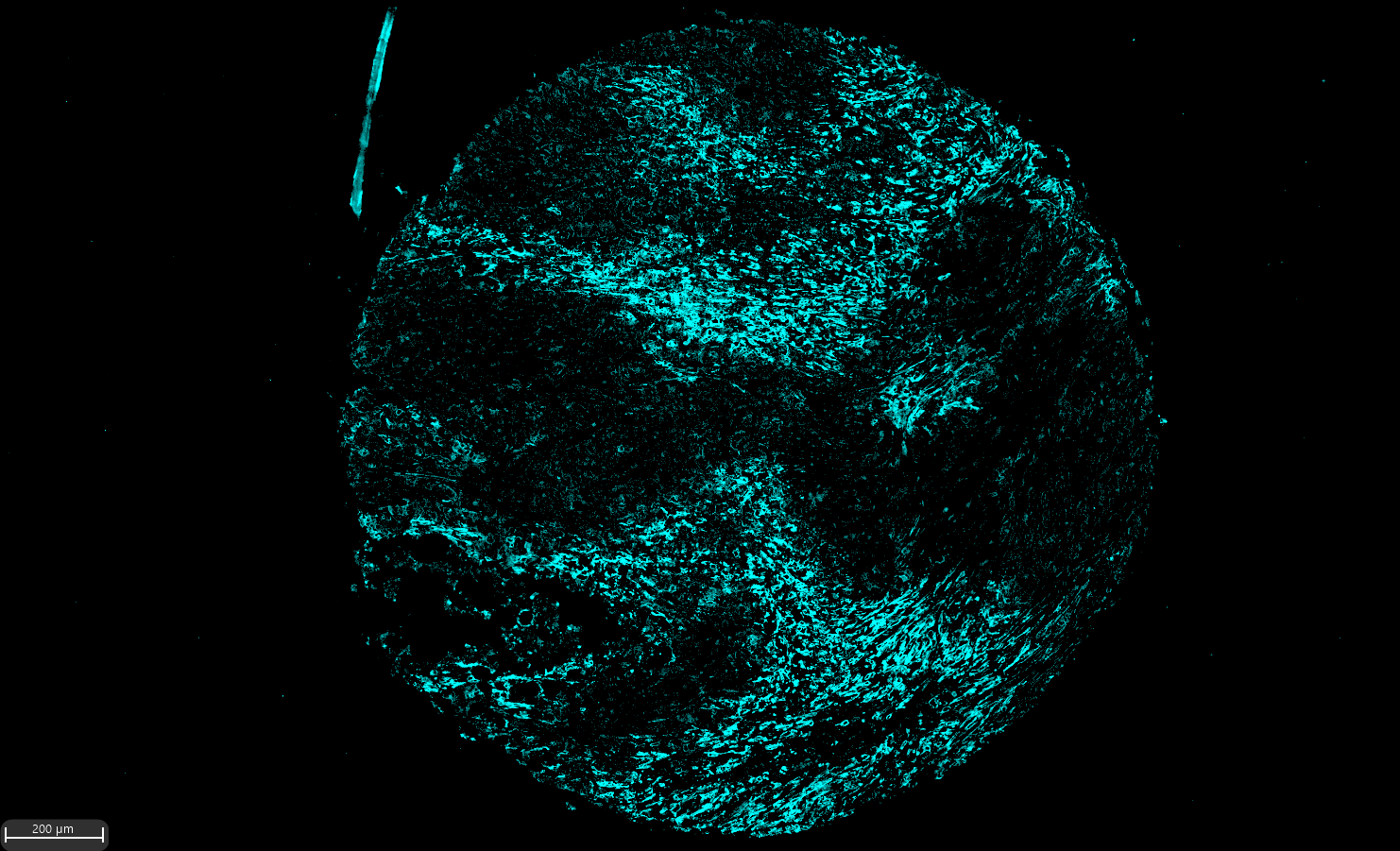

Supplement: Supplementary file 7 — Source data Fig. 5 [file 44318_2024_319_MOESM7_ESM.zip › EMBOJ-2024-117498-T-SourceDataForFigure5B-H/Figure 5 G/README/CAIX staining only/Core 2 CAIX.tiff]

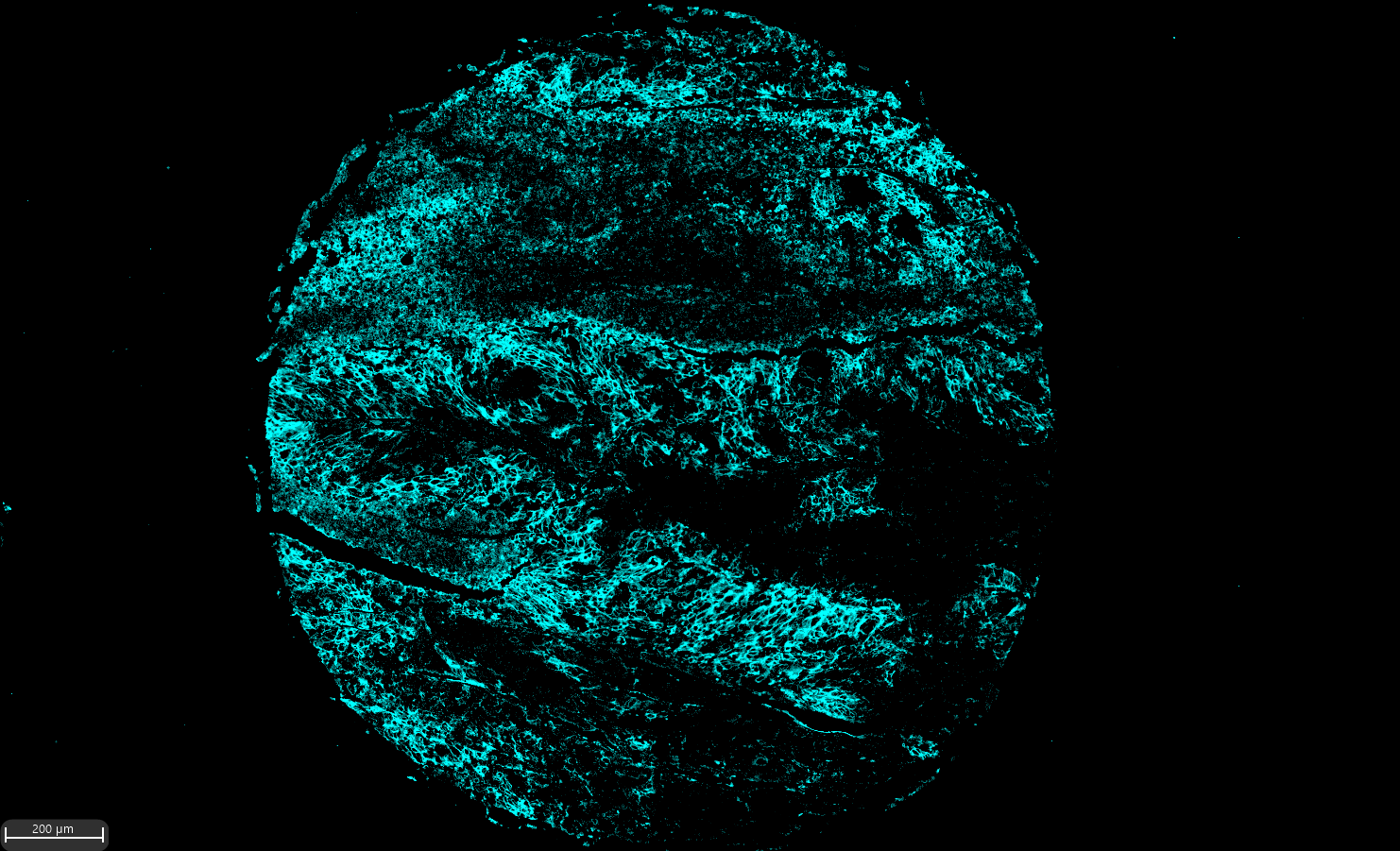

Supplement: Supplementary file 7 — Source data Fig. 5 [file 44318_2024_319_MOESM7_ESM.zip › EMBOJ-2024-117498-T-SourceDataForFigure5B-H/Figure 5 G/README/CAIX staining only/Core 3 CAIX .tiff]

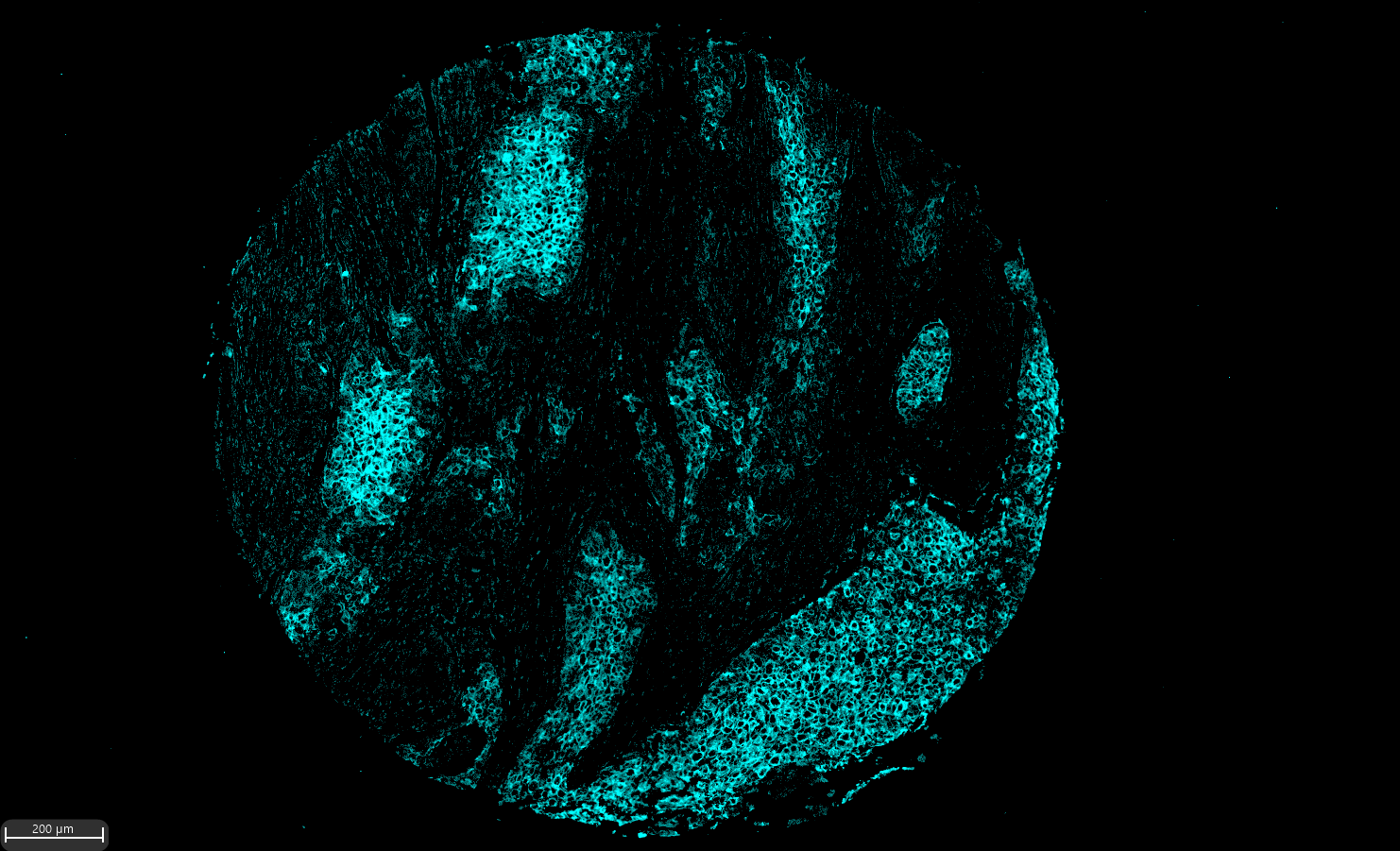

Supplement: Supplementary file 7 — Source data Fig. 5 [file 44318_2024_319_MOESM7_ESM.zip › EMBOJ-2024-117498-T-SourceDataForFigure5B-H/Figure 5 G/README/CAIX staining only/Core 4 CAIX.tiff]

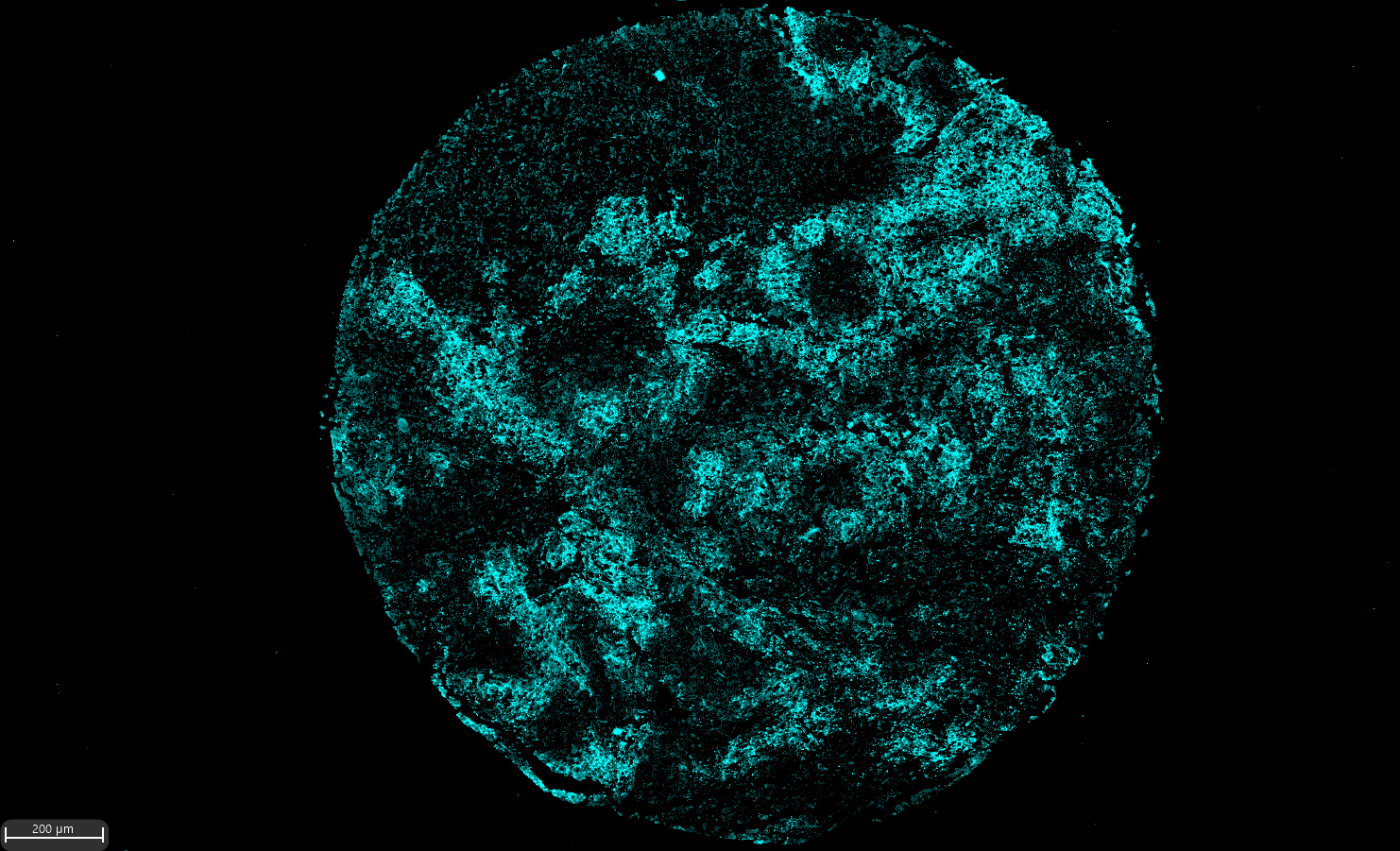

Supplement: Supplementary file 7 — Source data Fig. 5 [file 44318_2024_319_MOESM7_ESM.zip › EMBOJ-2024-117498-T-SourceDataForFigure5B-H/Figure 5 G/README/CAIX staining only/Core 5 CAIX.tiff]

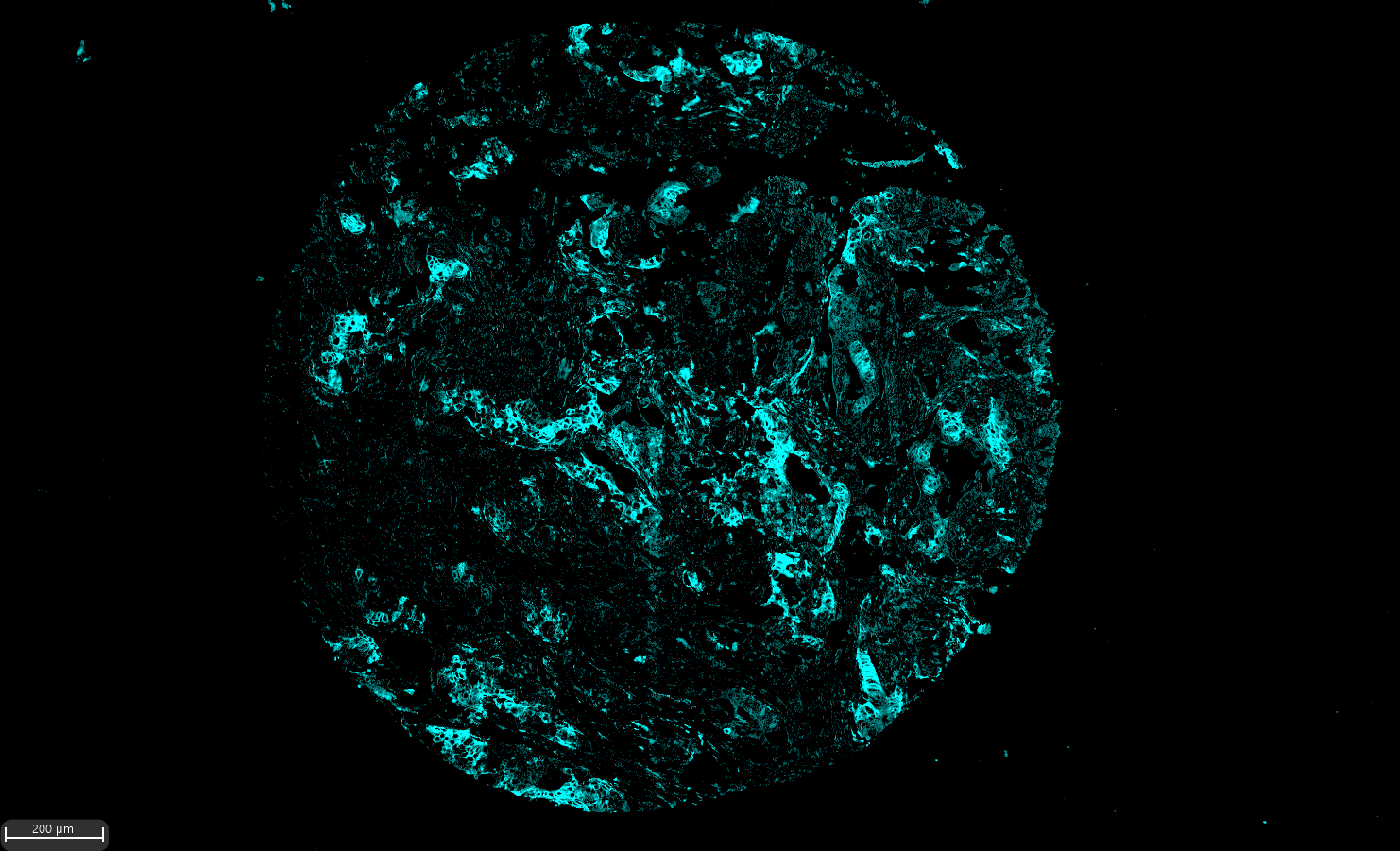

Supplement: Supplementary file 7 — Source data Fig. 5 [file 44318_2024_319_MOESM7_ESM.zip › EMBOJ-2024-117498-T-SourceDataForFigure5B-H/Figure 5 G/README/CAIX staining only/Core 6 CAIX.tiff]

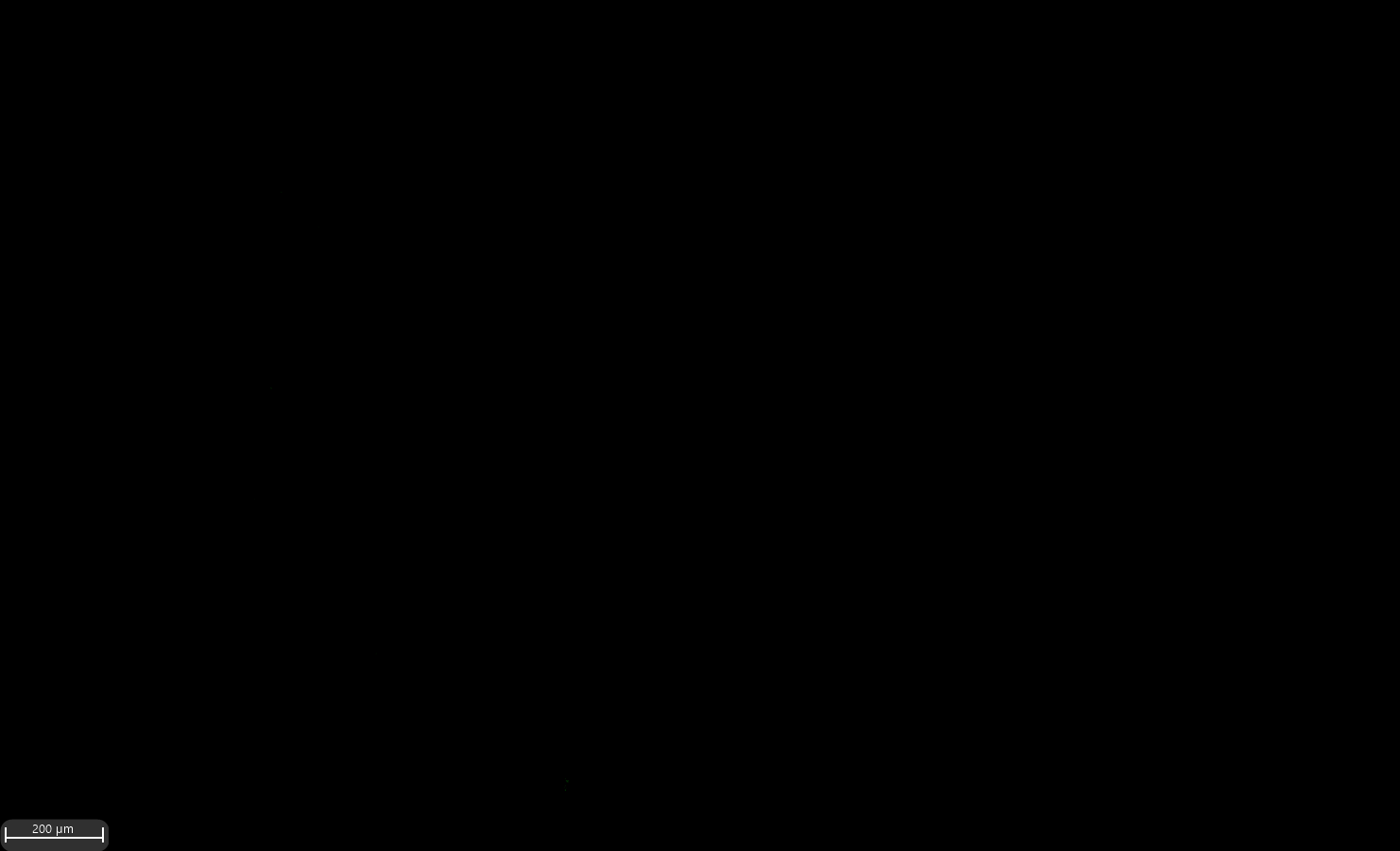

Supplement: Supplementary file 7 — Source data Fig. 5 [file 44318_2024_319_MOESM7_ESM.zip › EMBOJ-2024-117498-T-SourceDataForFigure5B-H/Figure 5 G/README/LMP2 staining only/Core 1 LMP2.tiff]

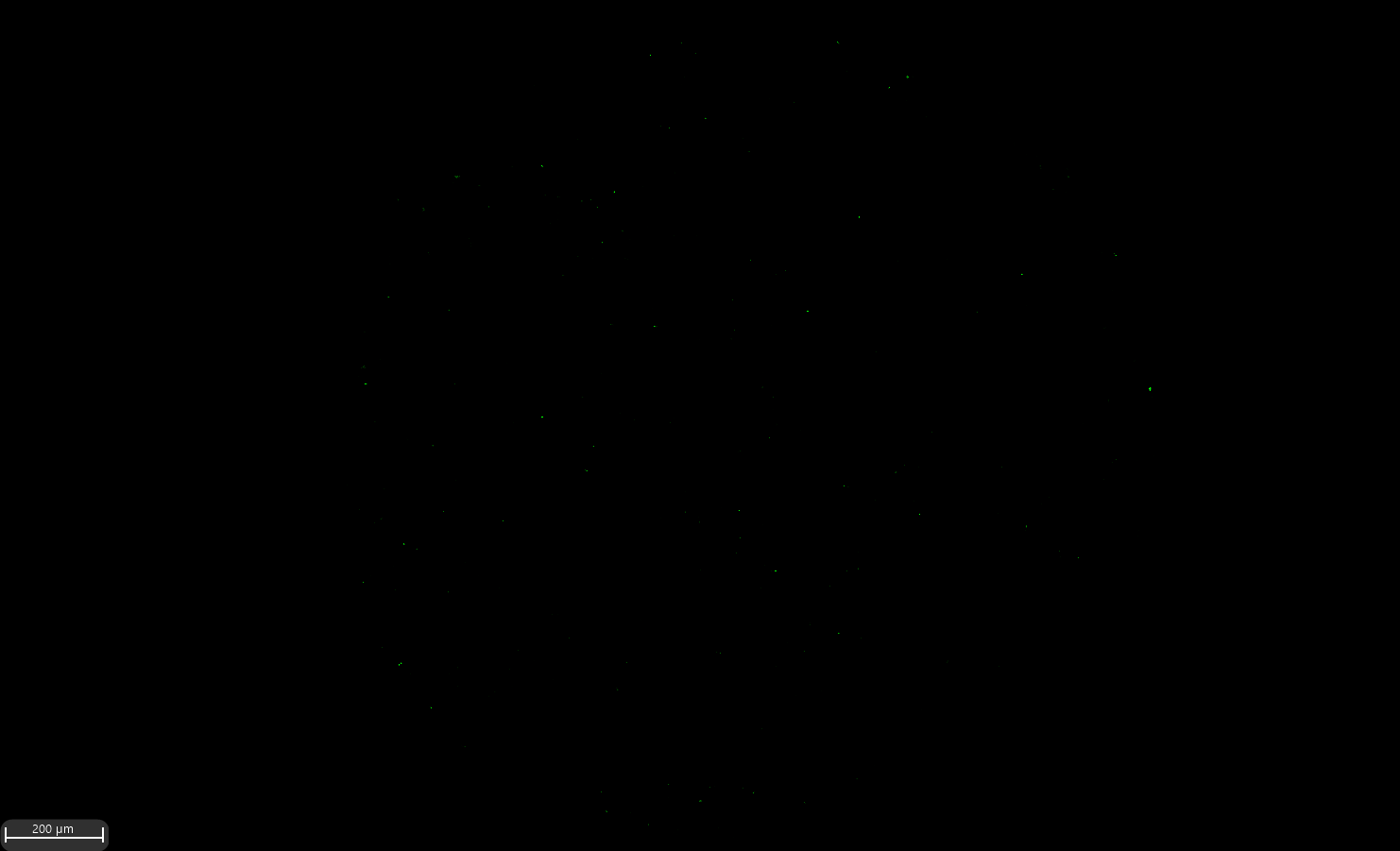

Supplement: Supplementary file 7 — Source data Fig. 5 [file 44318_2024_319_MOESM7_ESM.zip › EMBOJ-2024-117498-T-SourceDataForFigure5B-H/Figure 5 G/README/LMP2 staining only/Core 2 LMP2.tiff]

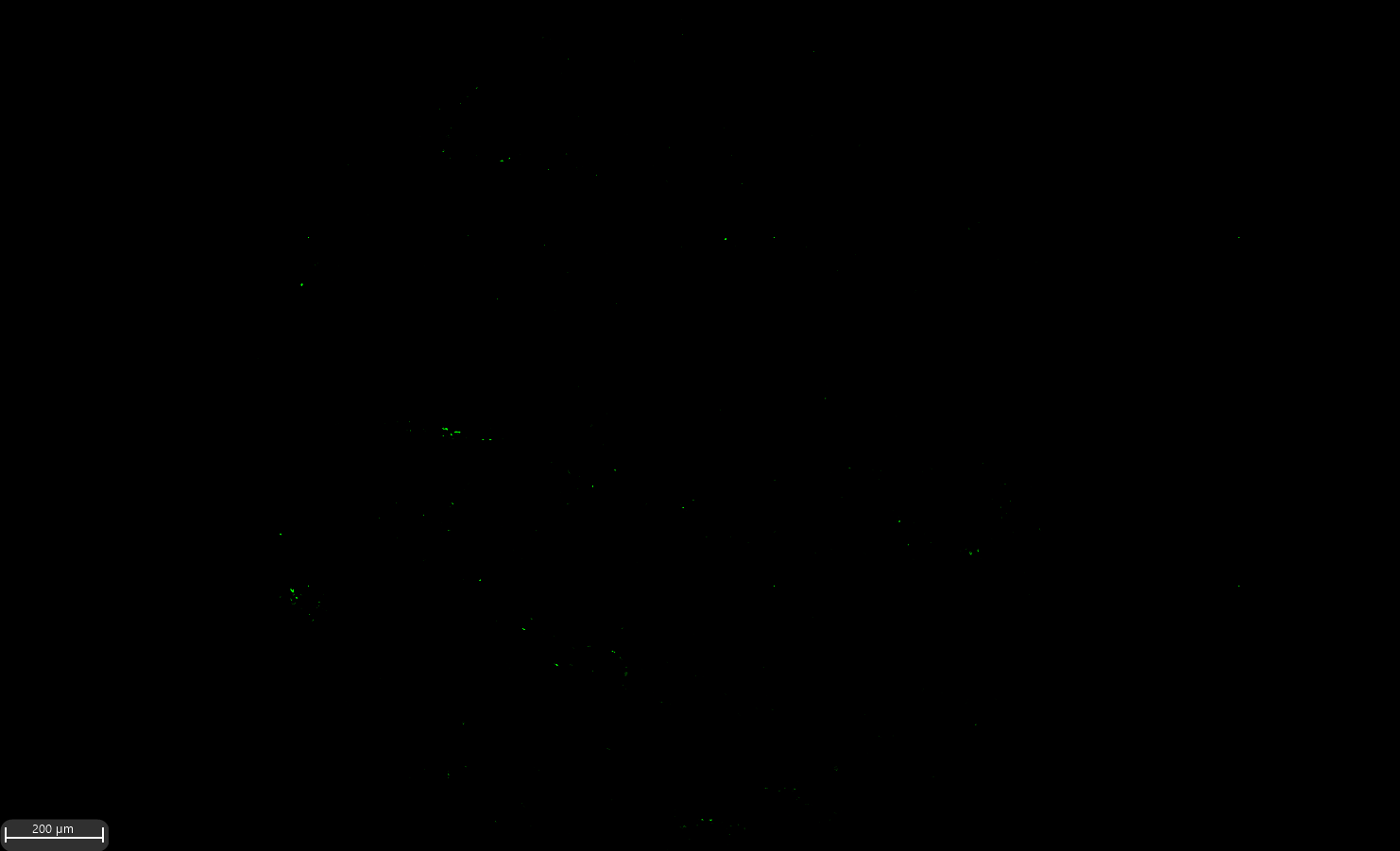

Supplement: Supplementary file 7 — Source data Fig. 5 [file 44318_2024_319_MOESM7_ESM.zip › EMBOJ-2024-117498-T-SourceDataForFigure5B-H/Figure 5 G/README/LMP2 staining only/Core 3 LMP2.tiff]

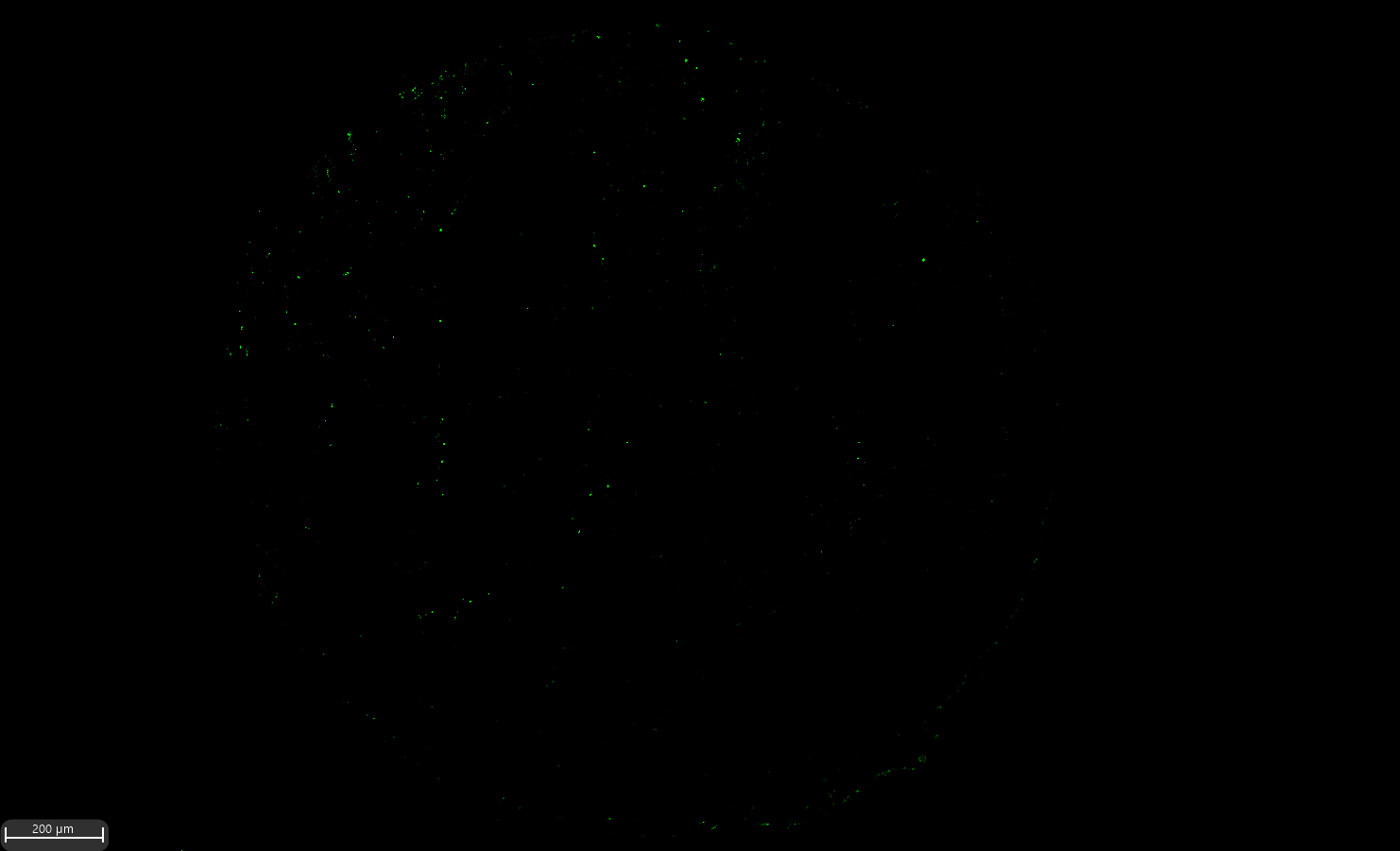

Supplement: Supplementary file 7 — Source data Fig. 5 [file 44318_2024_319_MOESM7_ESM.zip › EMBOJ-2024-117498-T-SourceDataForFigure5B-H/Figure 5 G/README/LMP2 staining only/Core 4 LMP2.tiff]

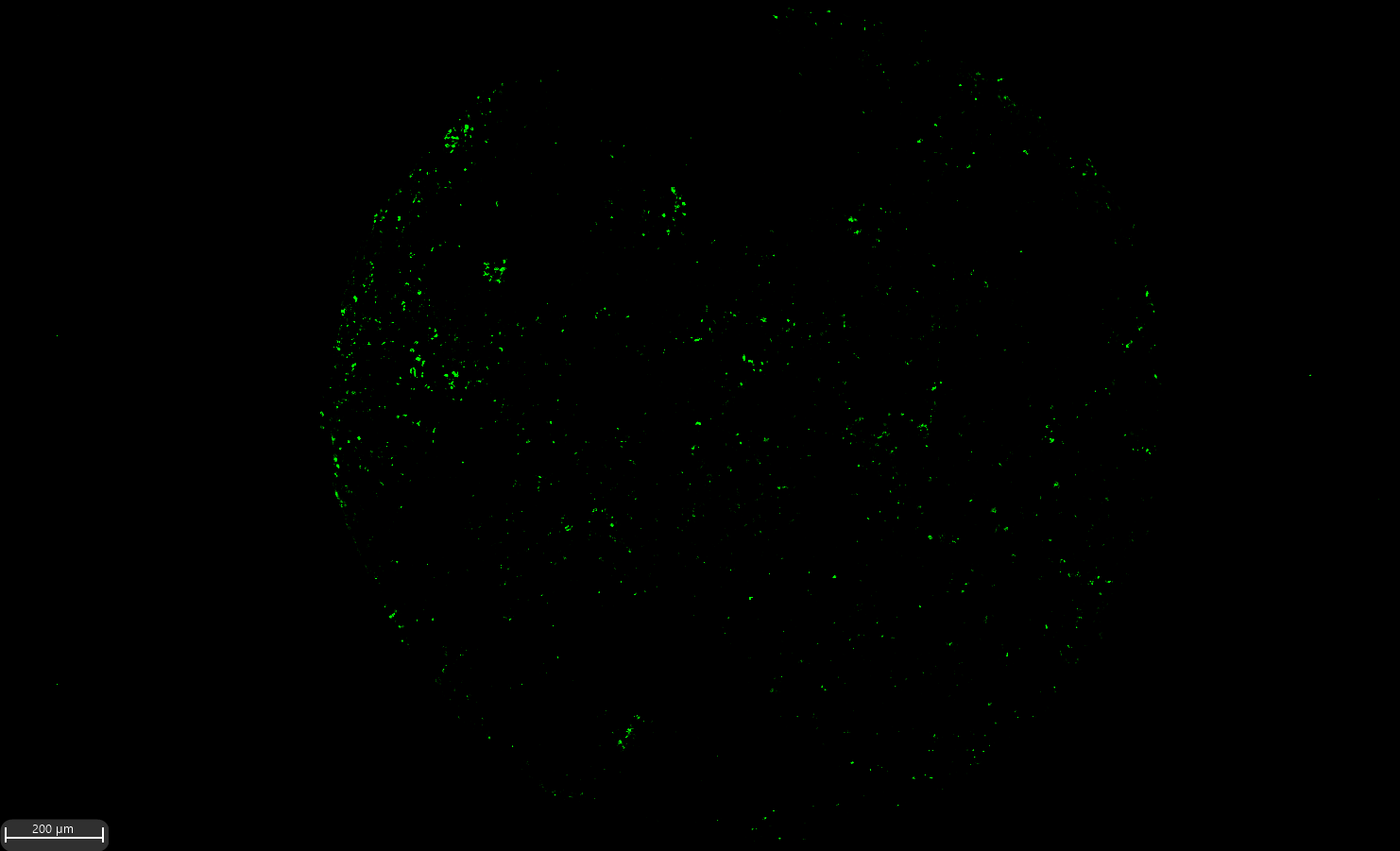

Supplement: Supplementary file 7 — Source data Fig. 5 [file 44318_2024_319_MOESM7_ESM.zip › EMBOJ-2024-117498-T-SourceDataForFigure5B-H/Figure 5 G/README/LMP2 staining only/Core 5 LMP2.tiff]

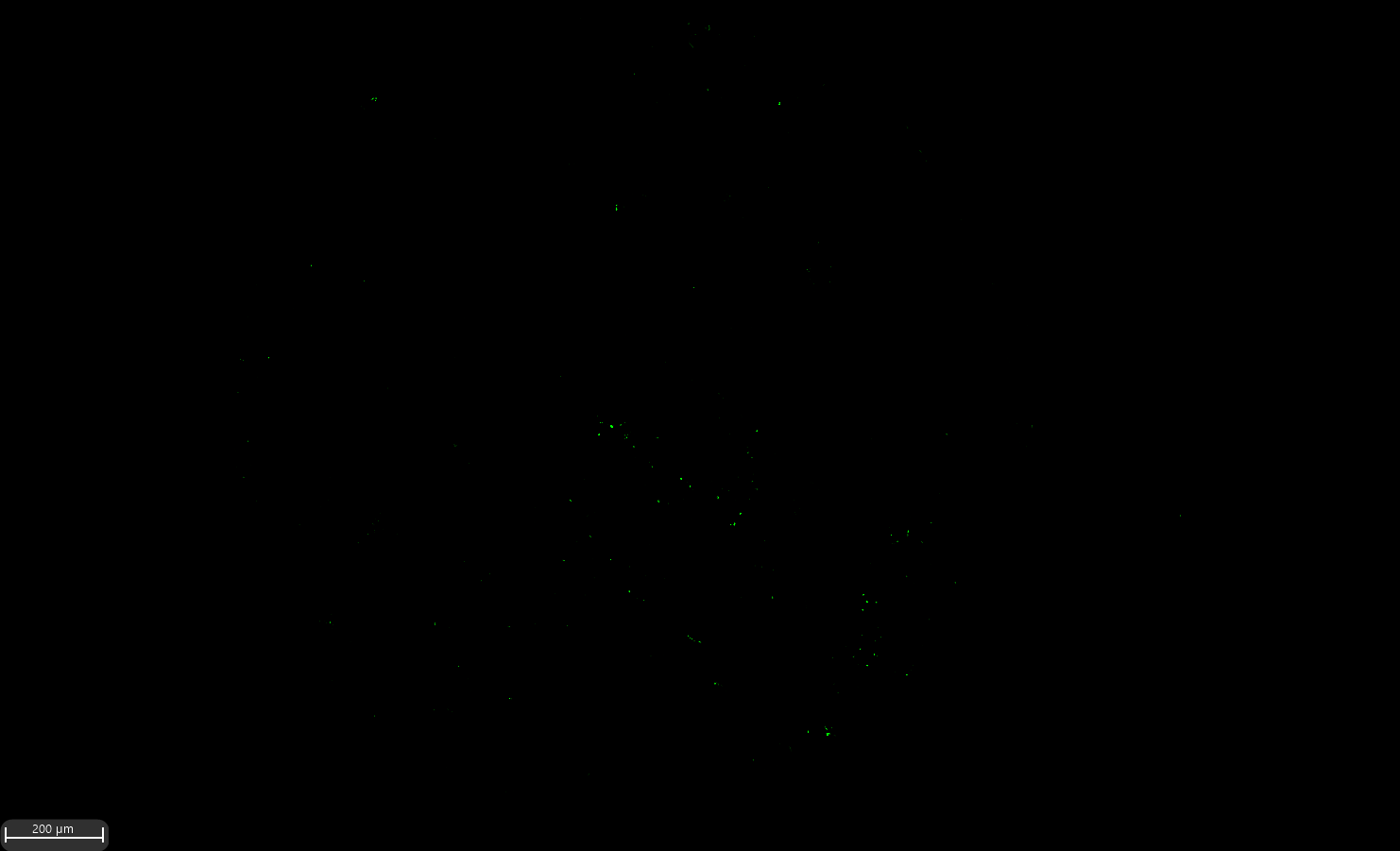

Supplement: Supplementary file 7 — Source data Fig. 5 [file 44318_2024_319_MOESM7_ESM.zip › EMBOJ-2024-117498-T-SourceDataForFigure5B-H/Figure 5 G/README/LMP2 staining only/Core 6 LMP2.tiff]

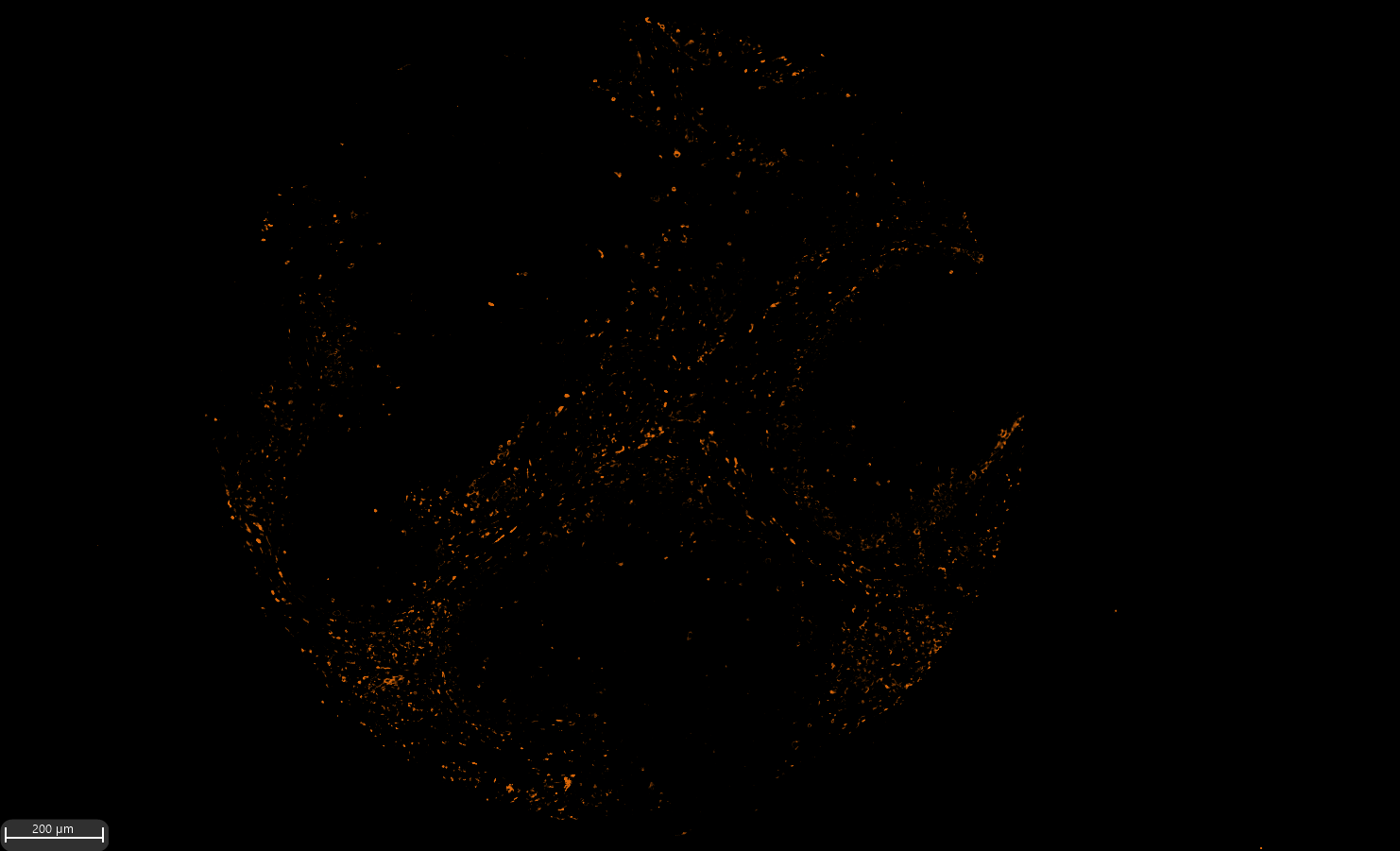

Supplement: Supplementary file 7 — Source data Fig. 5 [file 44318_2024_319_MOESM7_ESM.zip › EMBOJ-2024-117498-T-SourceDataForFigure5B-H/Figure 5 G/README/TAP2 staining only/Core 1 TAP2.tiff]

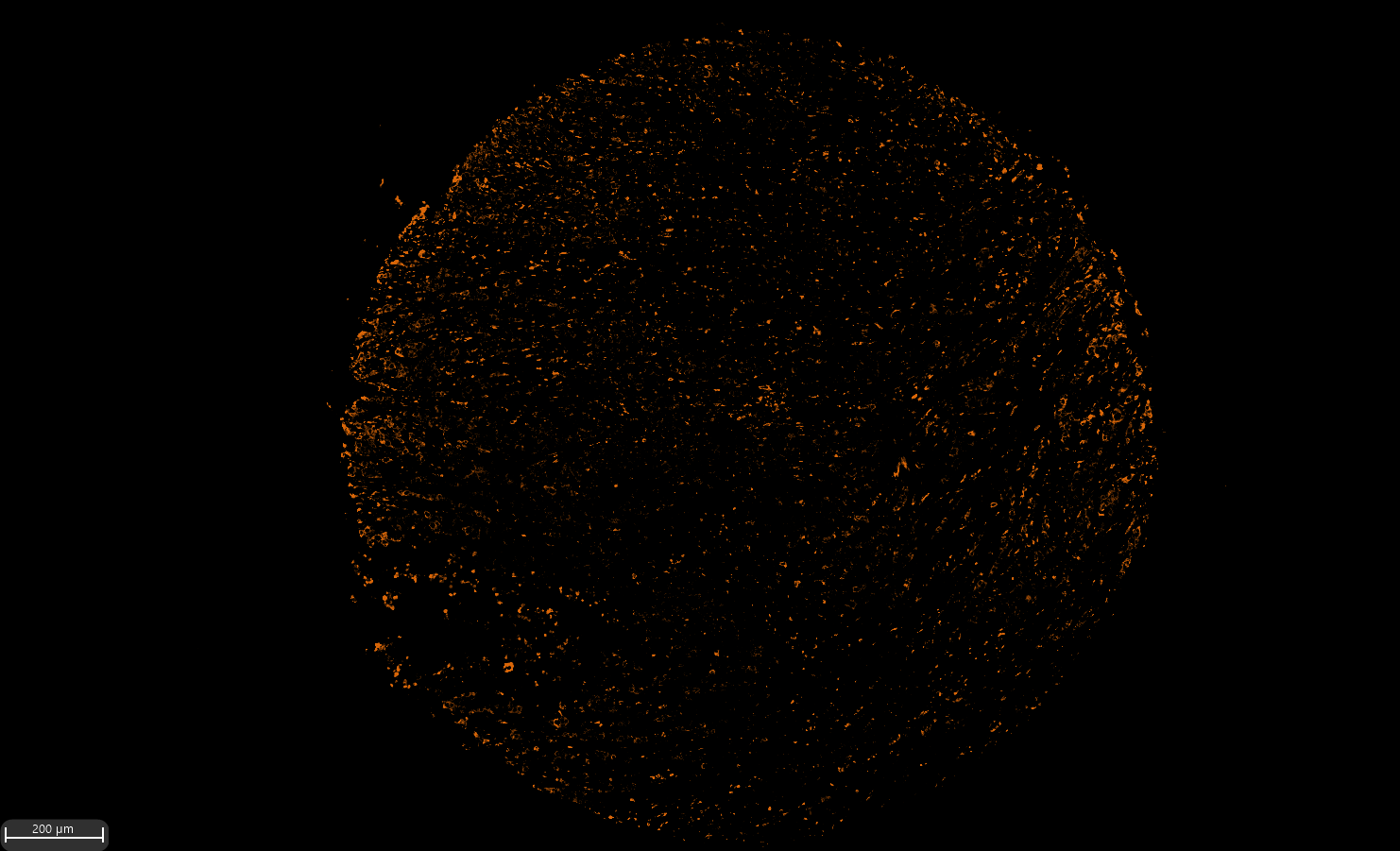

Supplement: Supplementary file 7 — Source data Fig. 5 [file 44318_2024_319_MOESM7_ESM.zip › EMBOJ-2024-117498-T-SourceDataForFigure5B-H/Figure 5 G/README/TAP2 staining only/Core 2 TAP2.tiff]

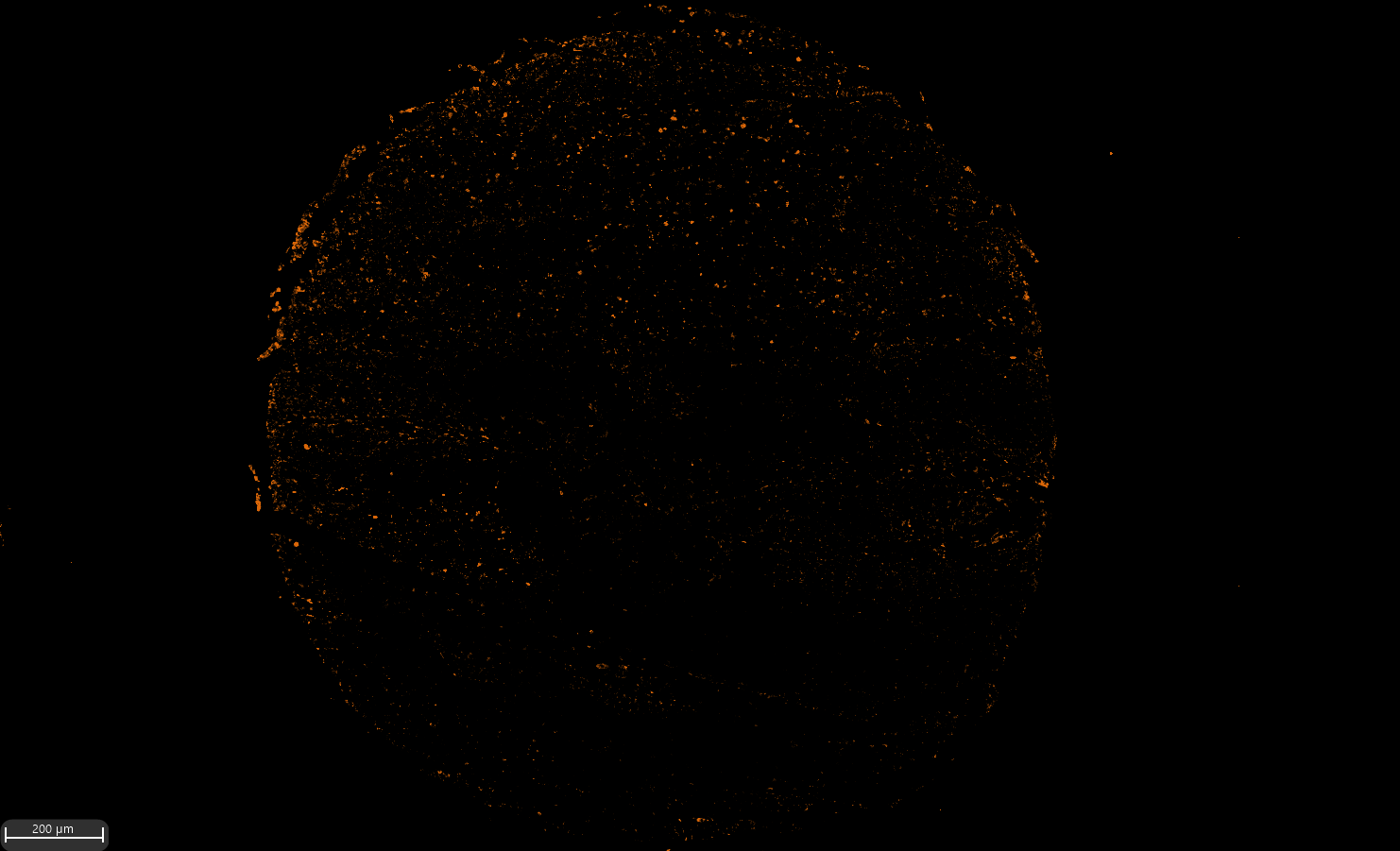

Supplement: Supplementary file 7 — Source data Fig. 5 [file 44318_2024_319_MOESM7_ESM.zip › EMBOJ-2024-117498-T-SourceDataForFigure5B-H/Figure 5 G/README/TAP2 staining only/Core 3 TAP2.tiff]

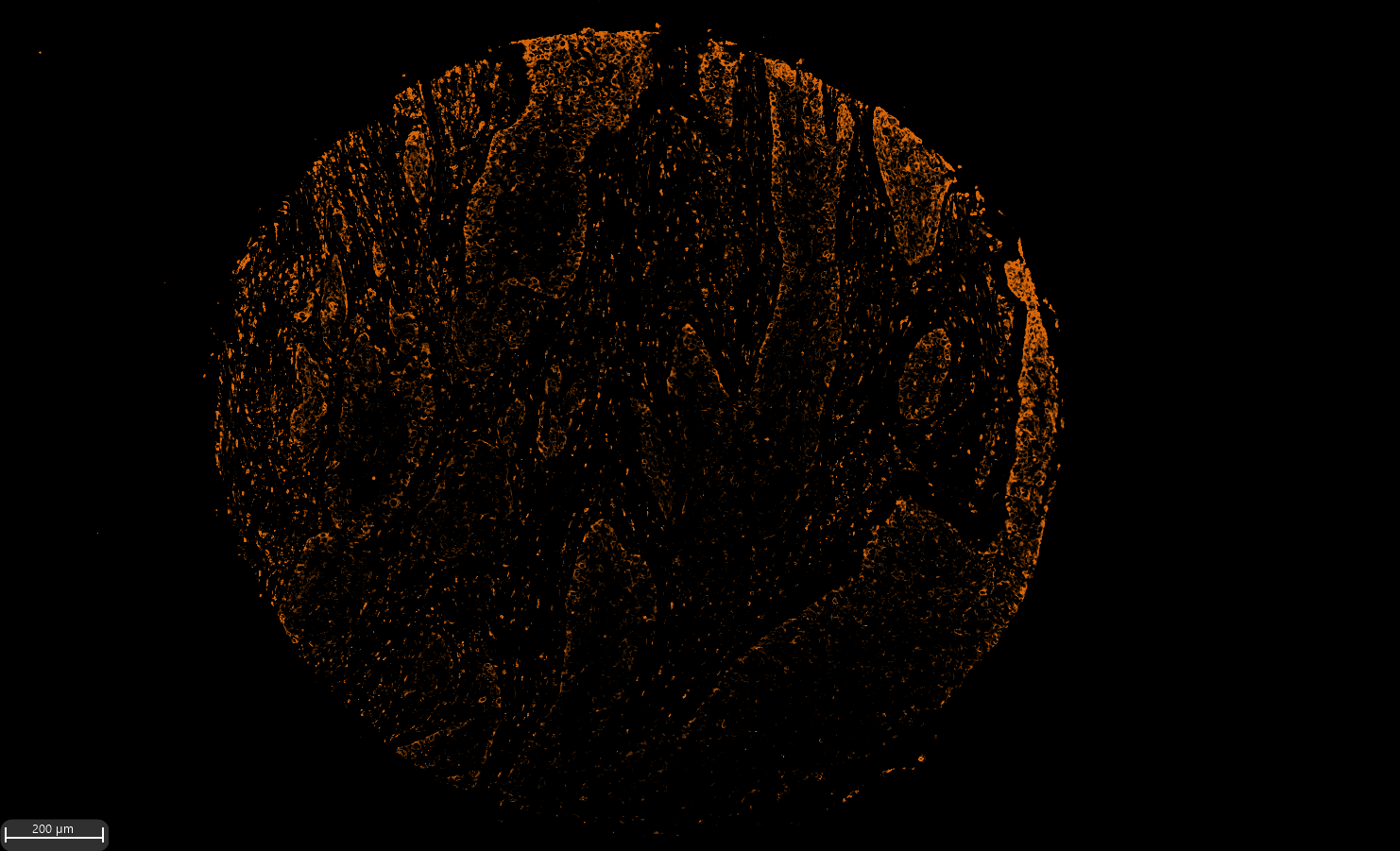

Supplement: Supplementary file 7 — Source data Fig. 5 [file 44318_2024_319_MOESM7_ESM.zip › EMBOJ-2024-117498-T-SourceDataForFigure5B-H/Figure 5 G/README/TAP2 staining only/Core 4 TAP2.tiff]

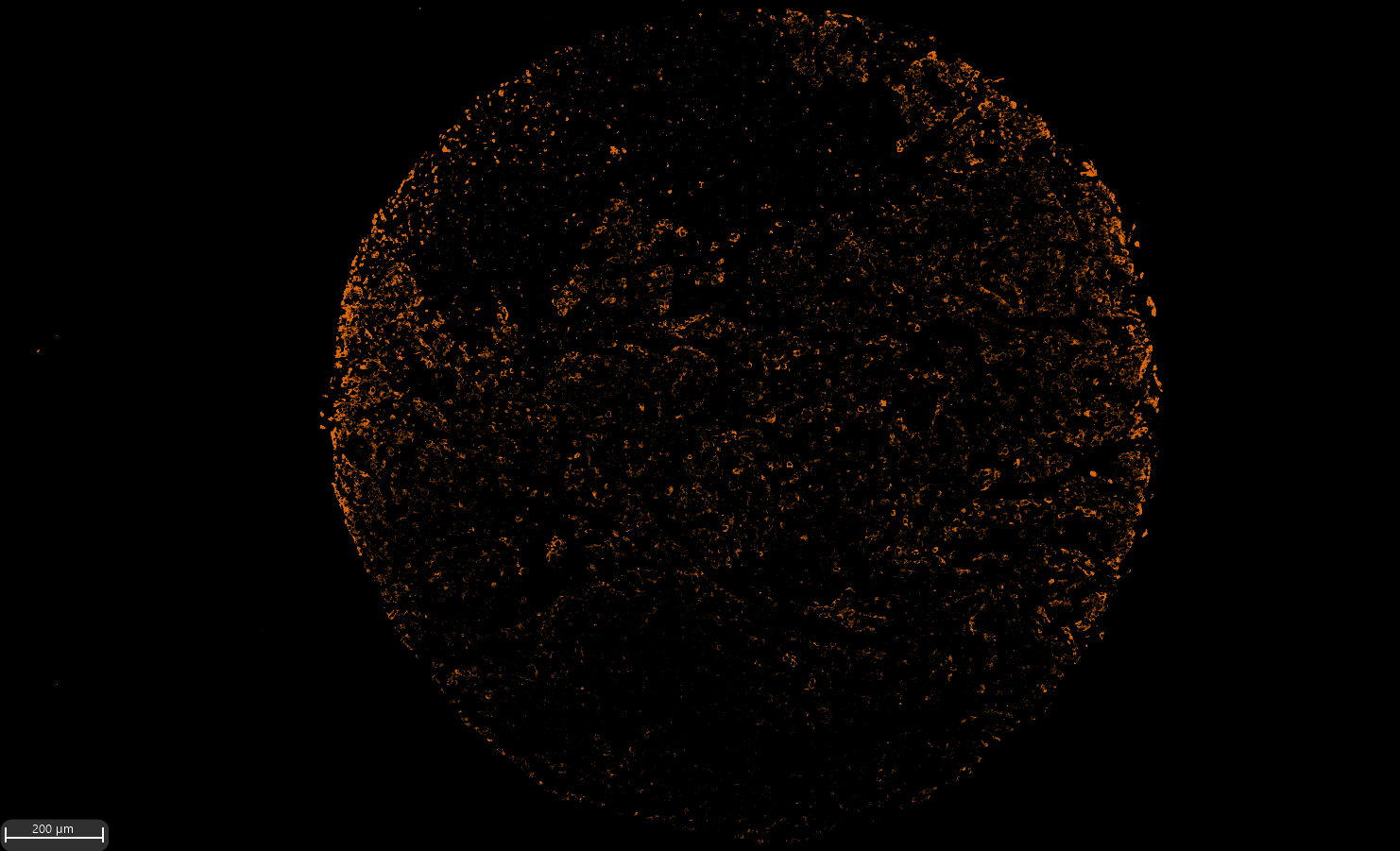

Supplement: Supplementary file 7 — Source data Fig. 5 [file 44318_2024_319_MOESM7_ESM.zip › EMBOJ-2024-117498-T-SourceDataForFigure5B-H/Figure 5 G/README/TAP2 staining only/Core 5 TAP2.tiff]

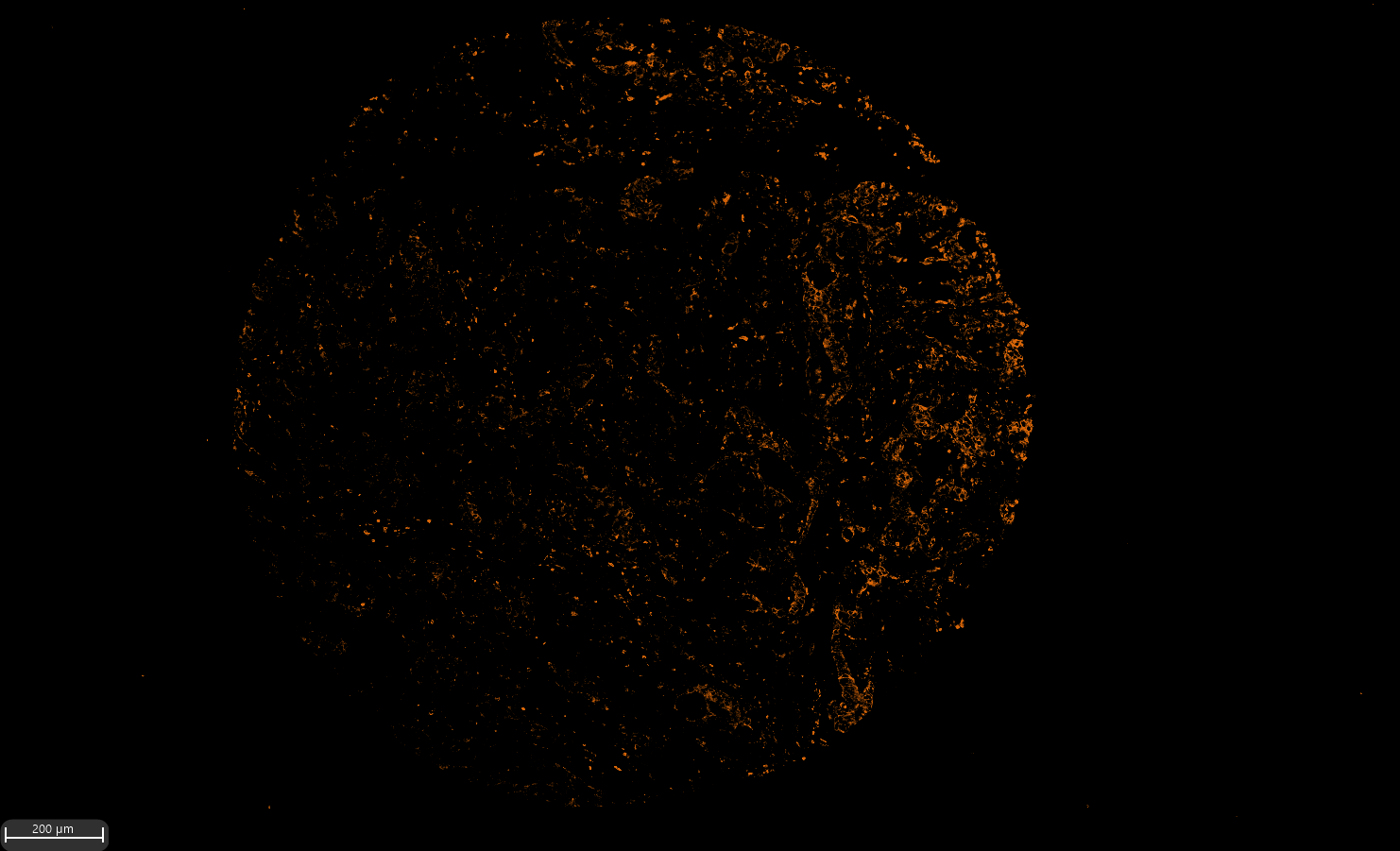

Supplement: Supplementary file 7 — Source data Fig. 5 [file 44318_2024_319_MOESM7_ESM.zip › EMBOJ-2024-117498-T-SourceDataForFigure5B-H/Figure 5 G/README/TAP2 staining only/Core 6 TAP2.tiff]

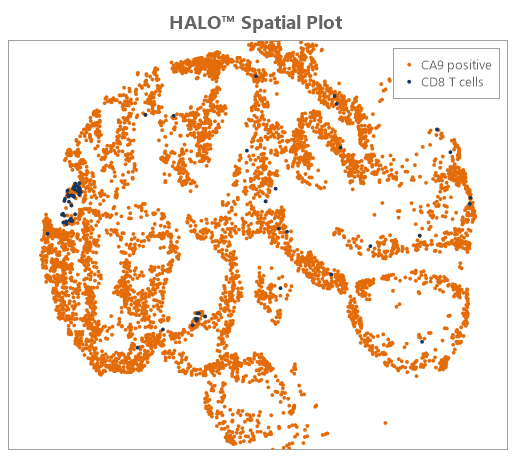

Supplement: Supplementary file 10 — Appendix Figure Source Data [file 44318_2024_319_MOESM10_ESM.zip › EMBOJ-2024-117498-T_SourceDataForAppendix/EMBOJ-2024-117498-T_SourceDataForAppendixFig. S1/Supplementary Figure 1A/Core 1.png]

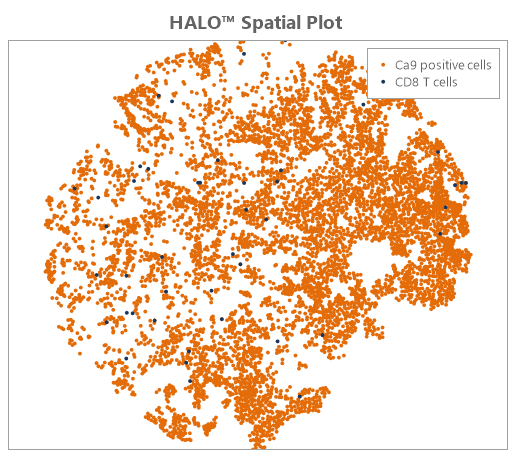

Supplement: Supplementary file 10 — Appendix Figure Source Data [file 44318_2024_319_MOESM10_ESM.zip › EMBOJ-2024-117498-T_SourceDataForAppendix/EMBOJ-2024-117498-T_SourceDataForAppendixFig. S1/Supplementary Figure 1A/Core 2.png]

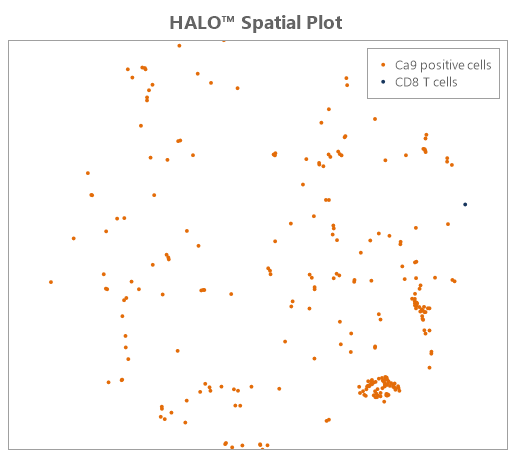

Supplement: Supplementary file 10 — Appendix Figure Source Data [file 44318_2024_319_MOESM10_ESM.zip › EMBOJ-2024-117498-T_SourceDataForAppendix/EMBOJ-2024-117498-T_SourceDataForAppendixFig. S1/Supplementary Figure 1A/Core 3.png]

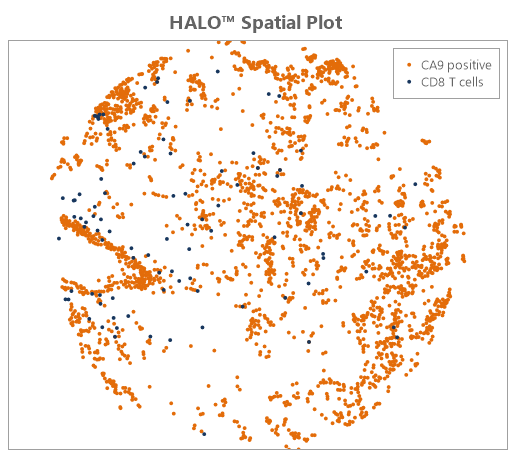

Supplement: Supplementary file 10 — Appendix Figure Source Data [file 44318_2024_319_MOESM10_ESM.zip › EMBOJ-2024-117498-T_SourceDataForAppendix/EMBOJ-2024-117498-T_SourceDataForAppendixFig. S1/Supplementary Figure 1A/Core 4.png]

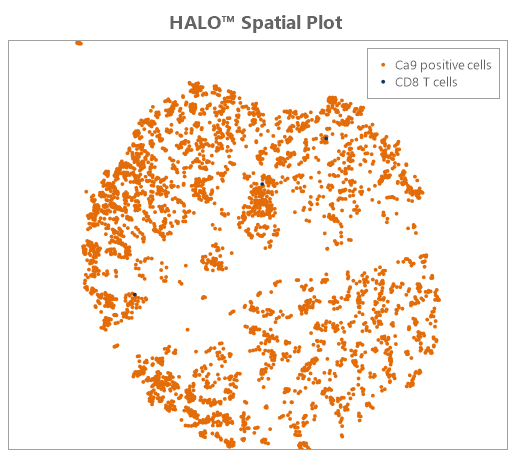

Supplement: Supplementary file 10 — Appendix Figure Source Data [file 44318_2024_319_MOESM10_ESM.zip › EMBOJ-2024-117498-T_SourceDataForAppendix/EMBOJ-2024-117498-T_SourceDataForAppendixFig. S1/Supplementary Figure 1A/Core 5.png]

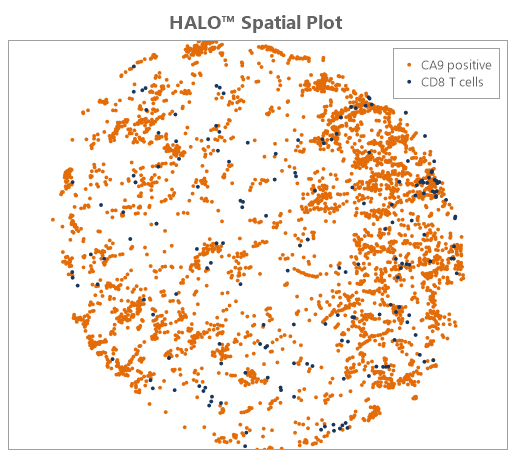

Supplement: Supplementary file 10 — Appendix Figure Source Data [file 44318_2024_319_MOESM10_ESM.zip › EMBOJ-2024-117498-T_SourceDataForAppendix/EMBOJ-2024-117498-T_SourceDataForAppendixFig. S1/Supplementary Figure 1A/Core 6.png]

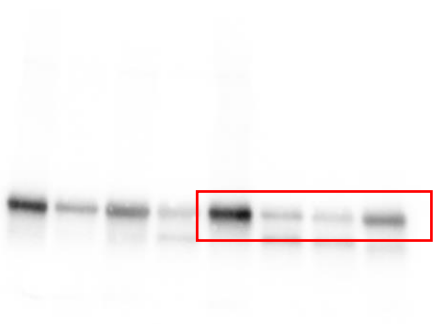

MHC I

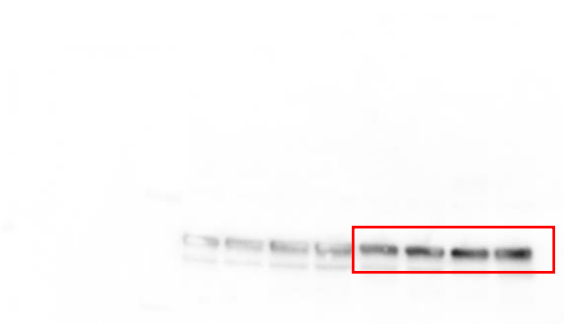

$\beta$ -tubulin

MHC I

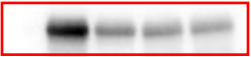

$\beta$ -tubulin

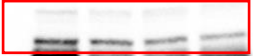

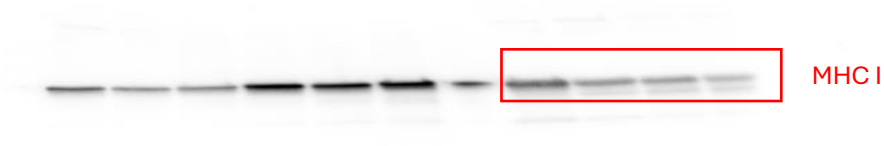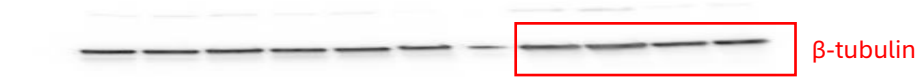

Supplement: Supplementary file 10 — Appendix Figure Source Data [file 44318_2024_319_MOESM10_ESM.zip › EMBOJ-2024-117498-T_SourceDataForAppendix/EMBOJ-2024-117498-T_SourceDataForAppendixFig. S2/Supplementary Figure 2C/README/HCT116_all biological replicates_western.pdf]

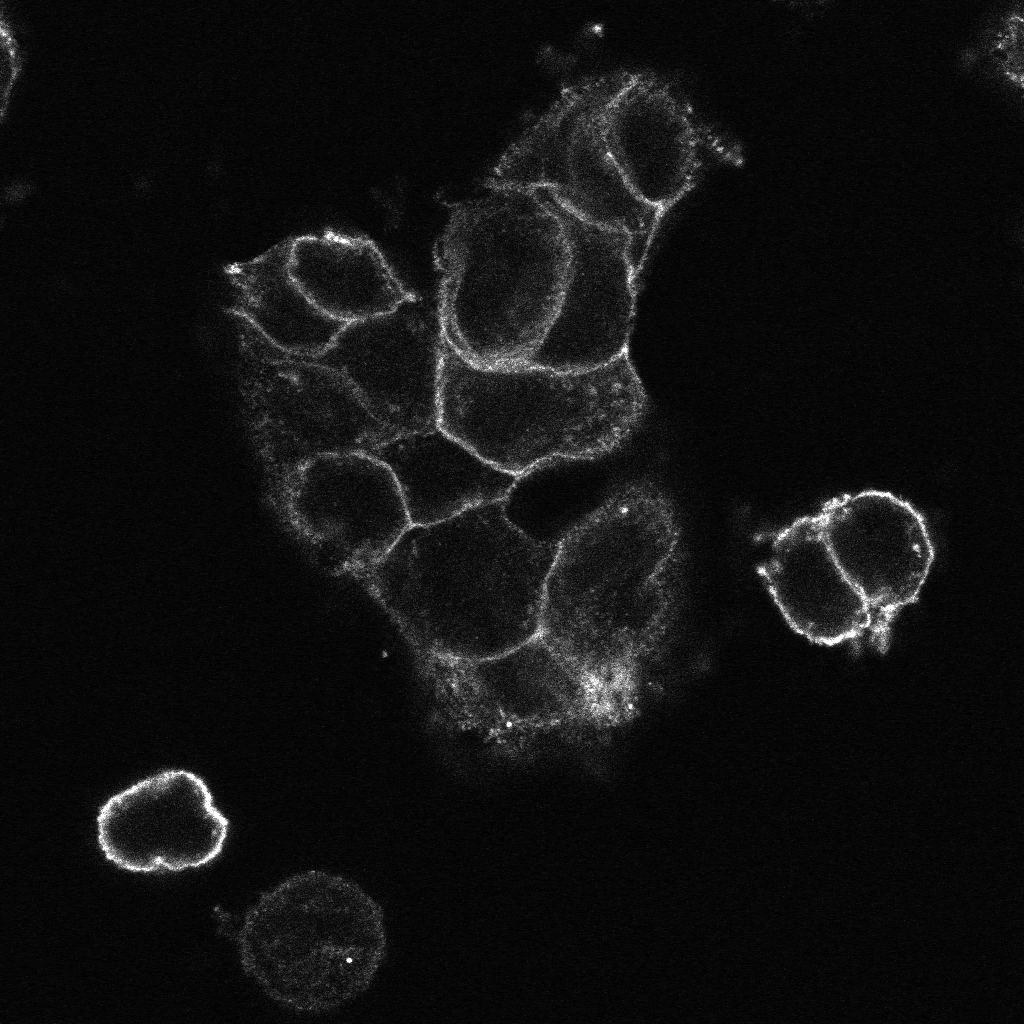

Supplement: Supplementary file 10 — Appendix Figure Source Data [file 44318_2024_319_MOESM10_ESM.zip › EMBOJ-2024-117498-T_SourceDataForAppendix/EMBOJ-2024-117498-T_SourceDataForAppendixFig. S2/Supplementary Figure 2D/HT29_MHC I Dapi IF staining_ hypoxia 16h time point .tif]

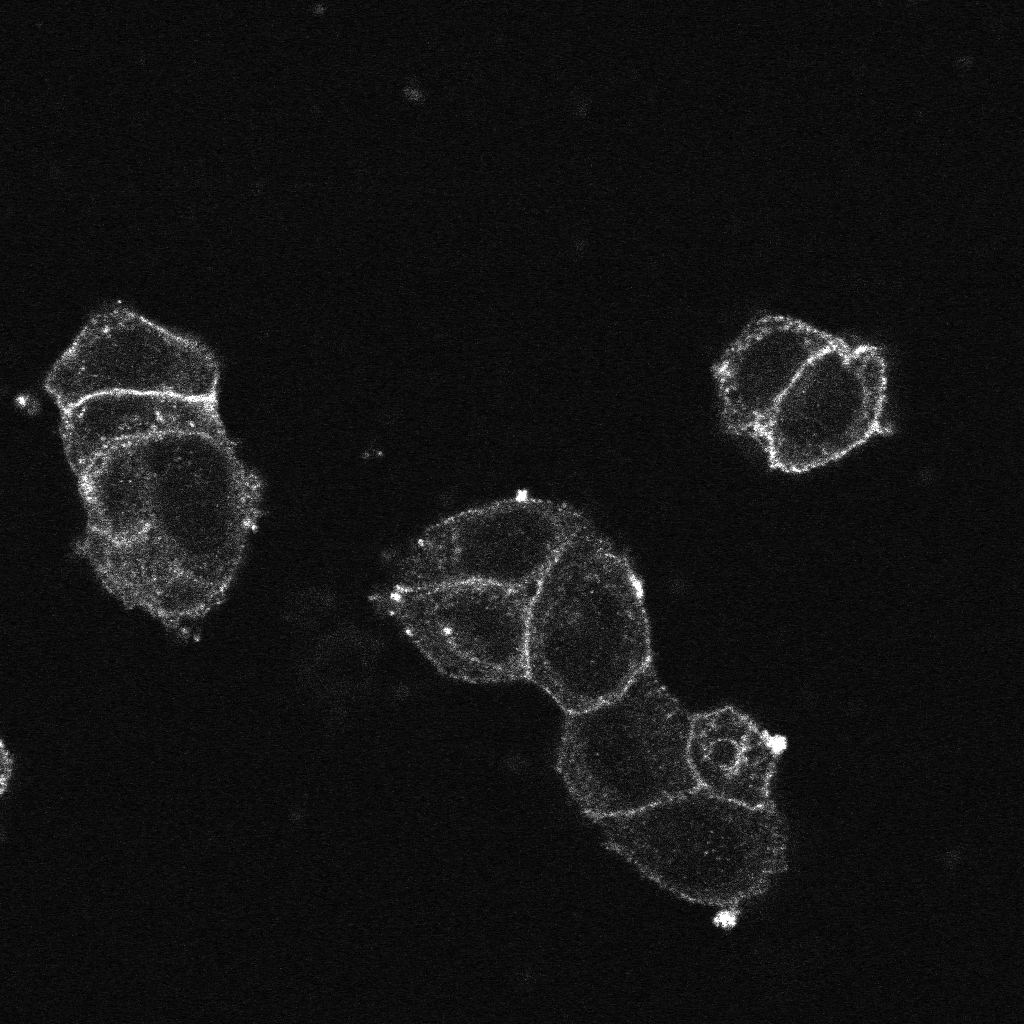

Supplement: Supplementary file 10 — Appendix Figure Source Data [file 44318_2024_319_MOESM10_ESM.zip › EMBOJ-2024-117498-T_SourceDataForAppendix/EMBOJ-2024-117498-T_SourceDataForAppendixFig. S2/Supplementary Figure 2D/HT29_MHC I Dapi IF staining_ hypoxia 24h time point .tif]

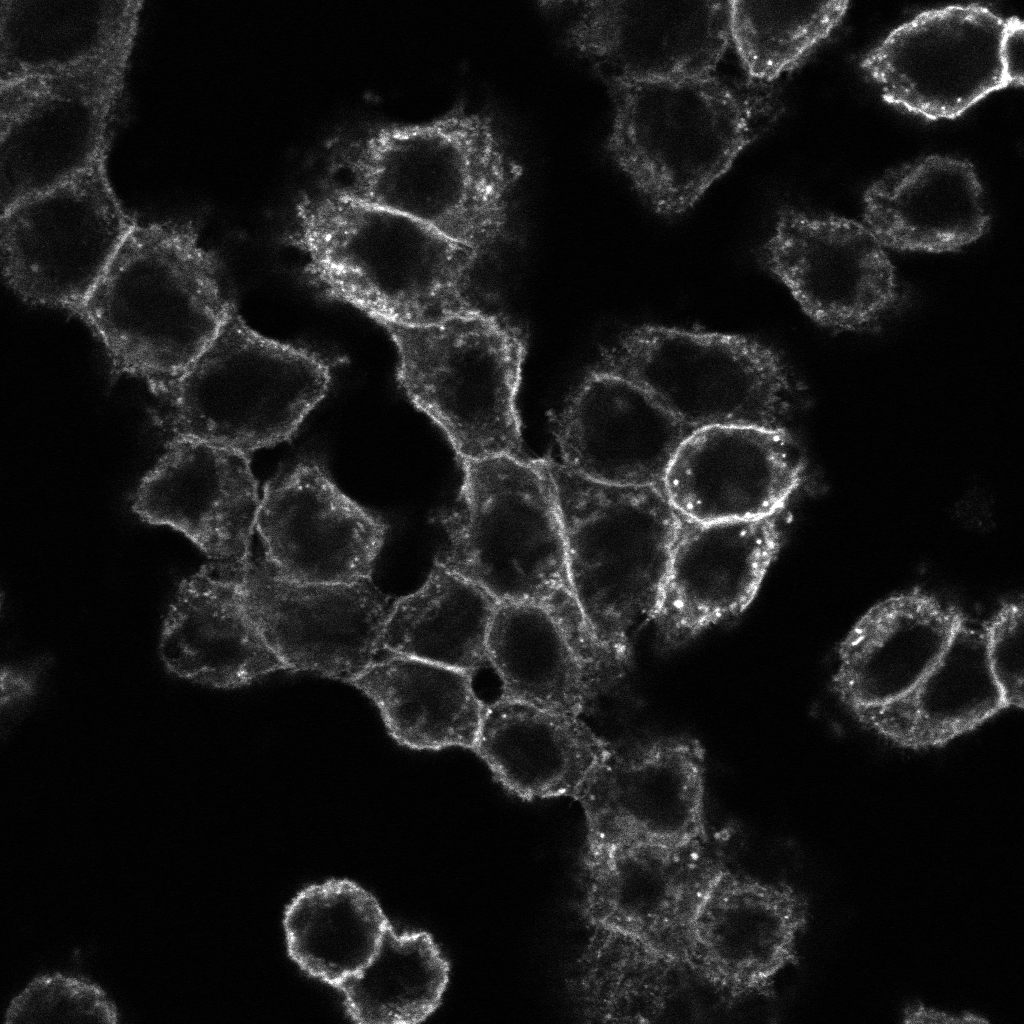

Supplement: Supplementary file 10 — Appendix Figure Source Data [file 44318_2024_319_MOESM10_ESM.zip › EMBOJ-2024-117498-T_SourceDataForAppendix/EMBOJ-2024-117498-T_SourceDataForAppendixFig. S2/Supplementary Figure 2D/HT29_MHC I Dapi IF staining_Normoxia .tif]

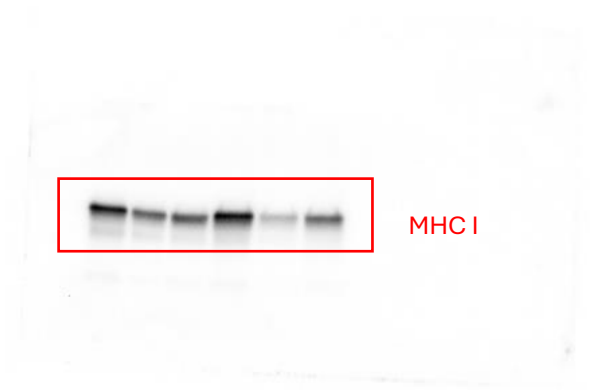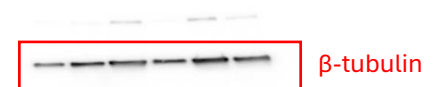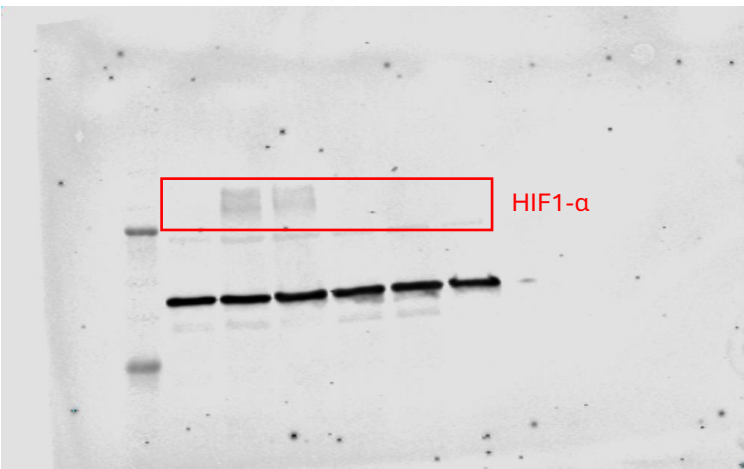

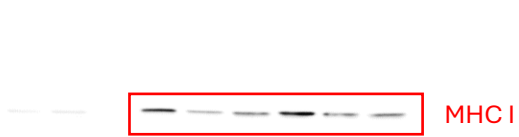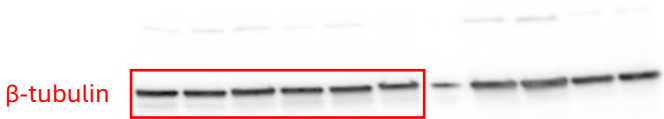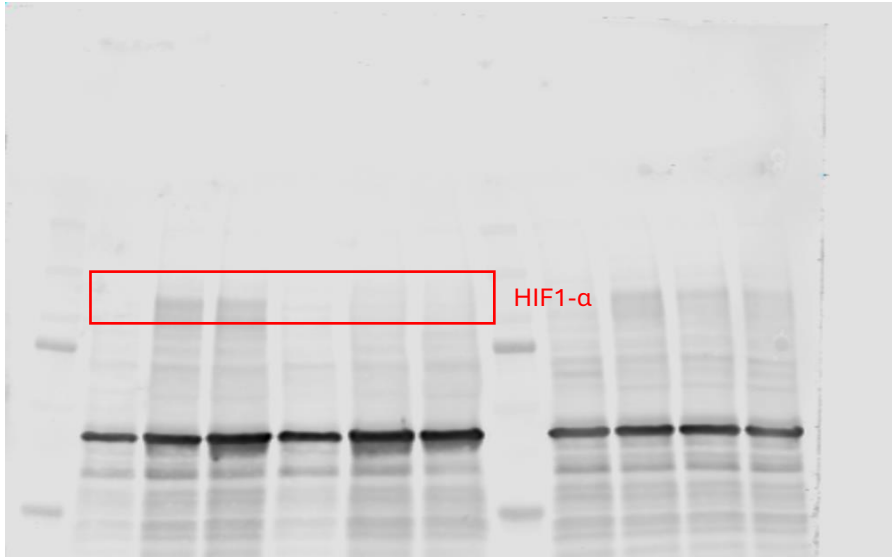

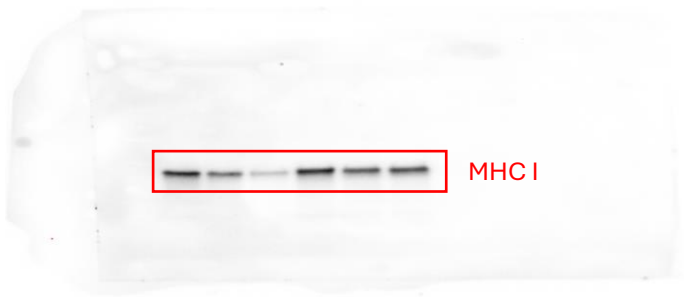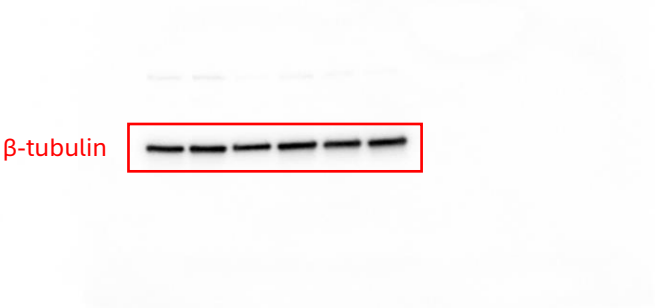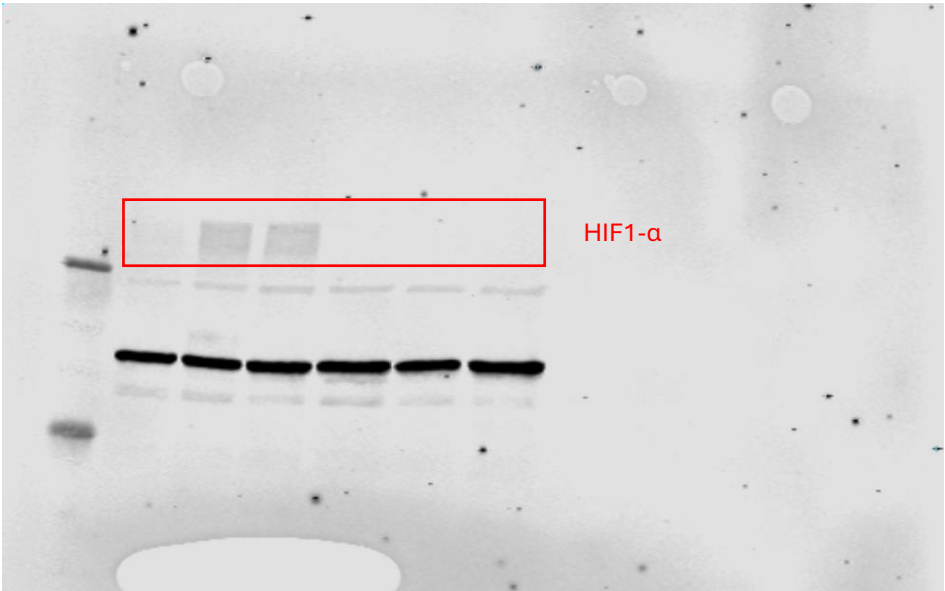

Supplement: Supplementary file 10 — Appendix Figure Source Data [file 44318_2024_319_MOESM10_ESM.zip › EMBOJ-2024-117498-T_SourceDataForAppendix/EMBOJ-2024-117498-T_SourceDataForAppendixFig. S2/Supplementary Figure 2H/README/RKO WT and HIF KO_all biological replicates_western.pdf]

MHC I

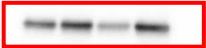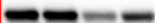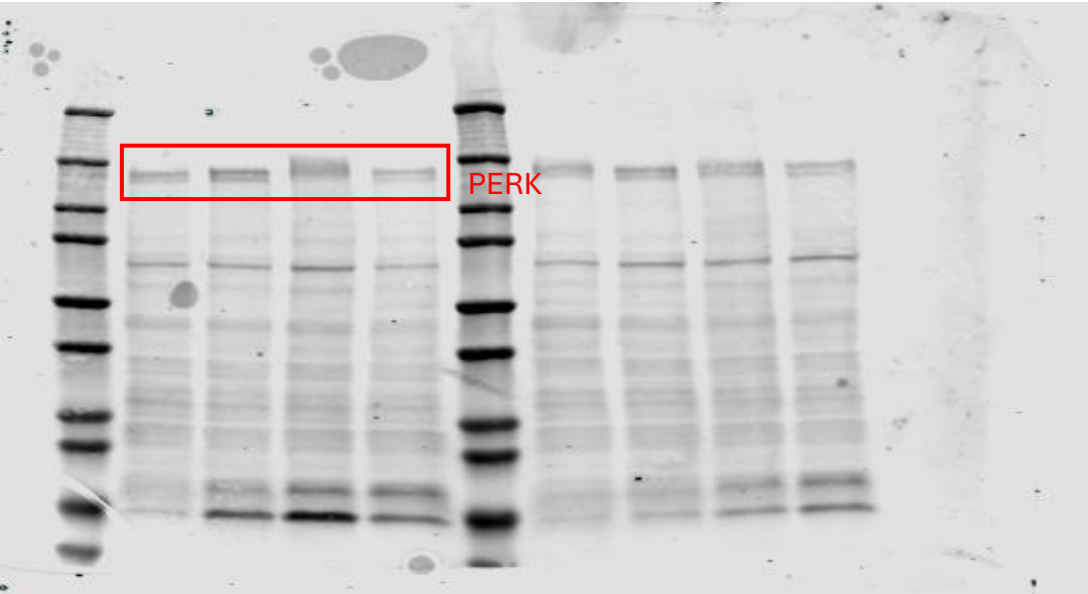

PERK

$\beta$ -actin

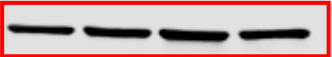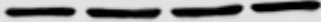

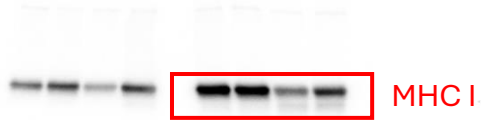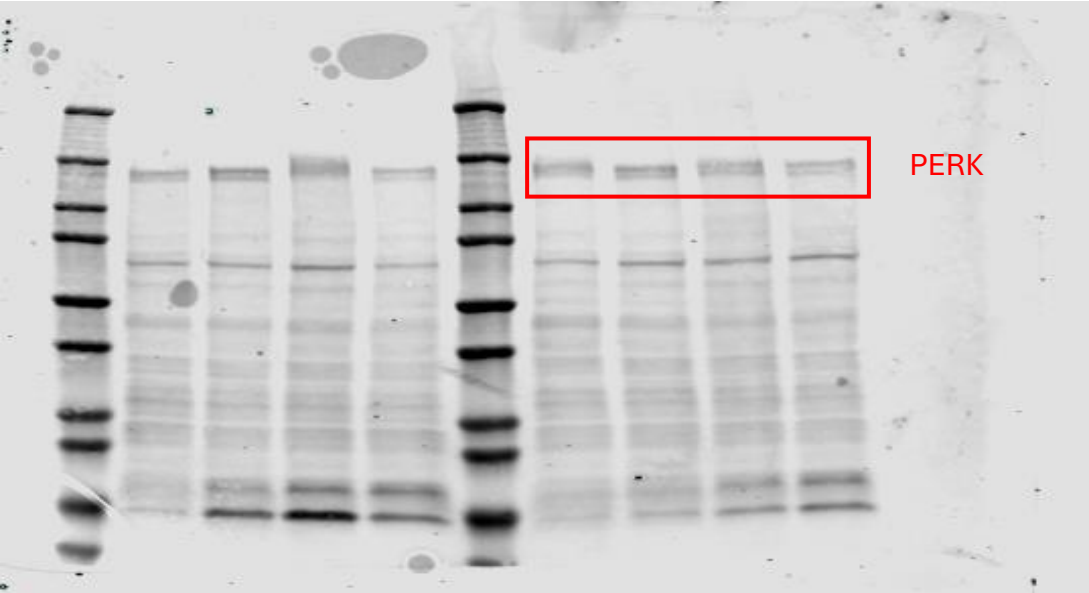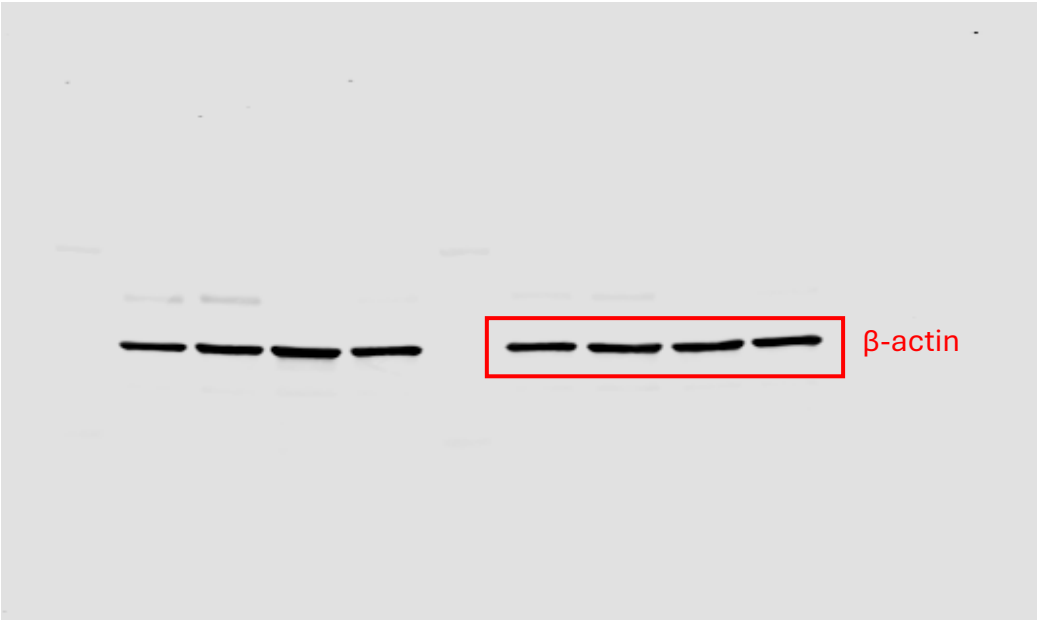

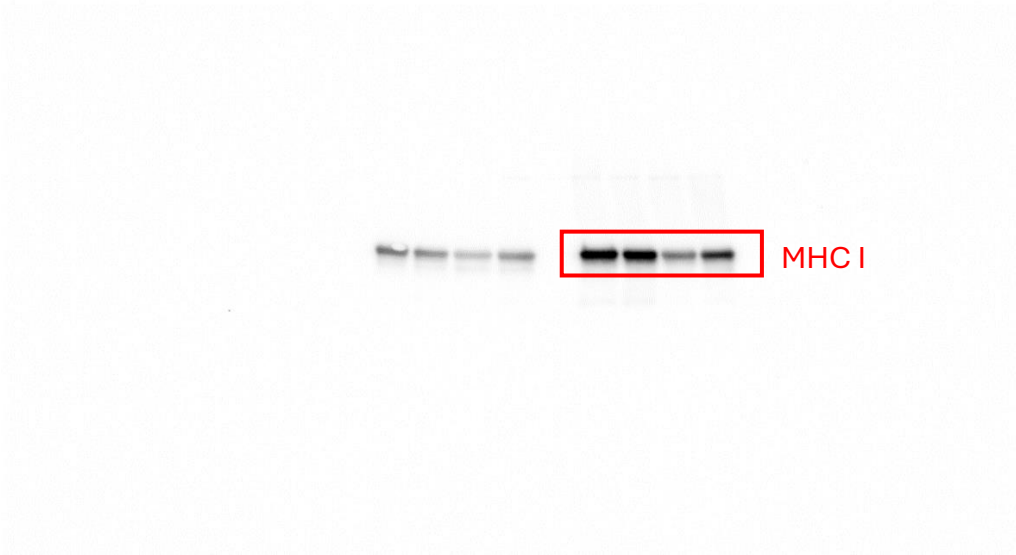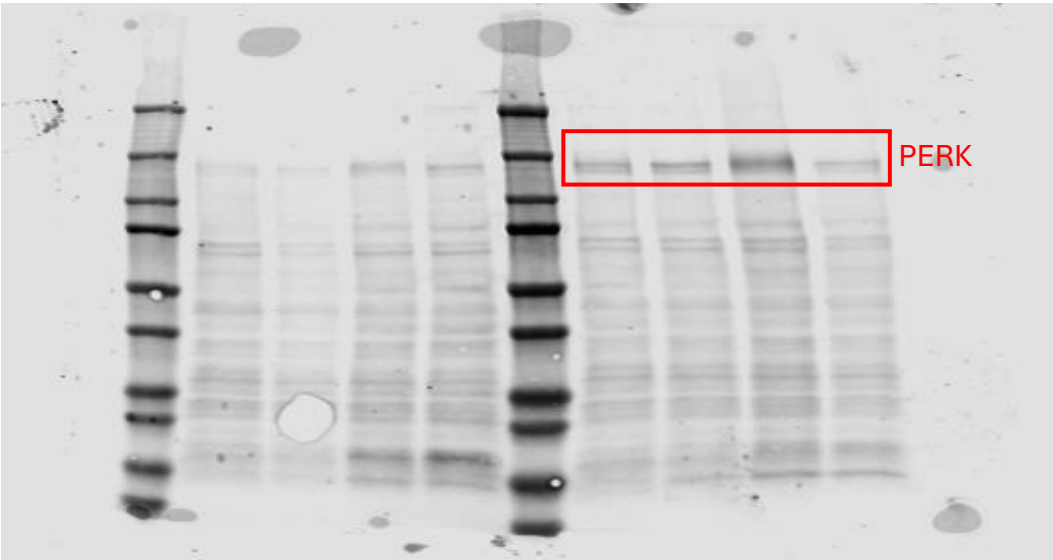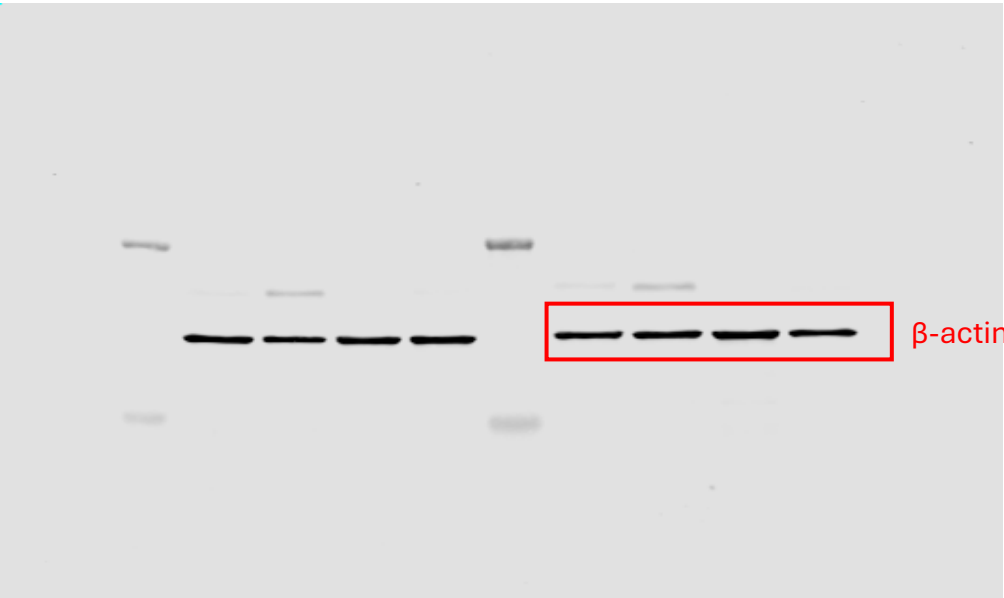

Supplement: Supplementary file 10 — Appendix Figure Source Data [file 44318_2024_319_MOESM10_ESM.zip › EMBOJ-2024-117498-T_SourceDataForAppendix/EMBOJ-2024-117498-T_SourceDataForAppendixFig. S3/Supplementary Figure 3A/README/HCT116_all biological replicates_Western.pdf]

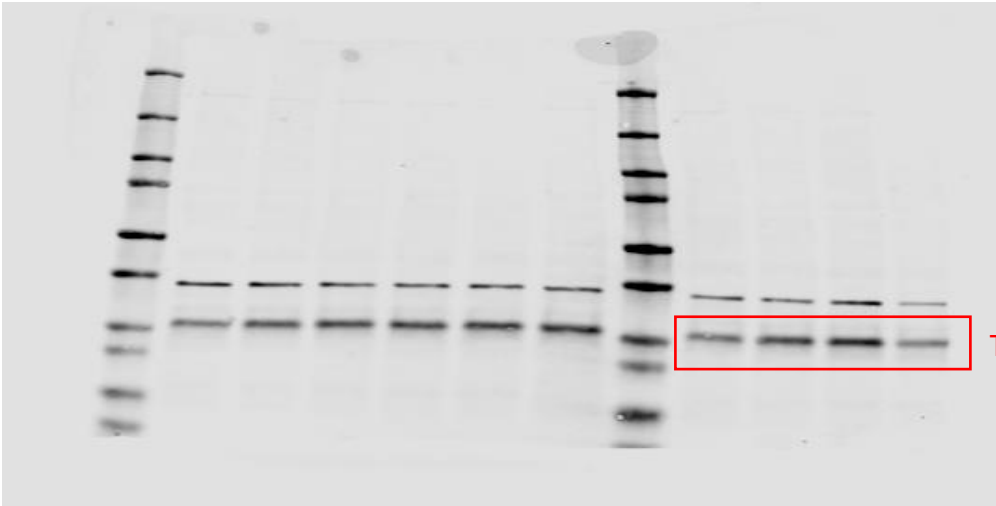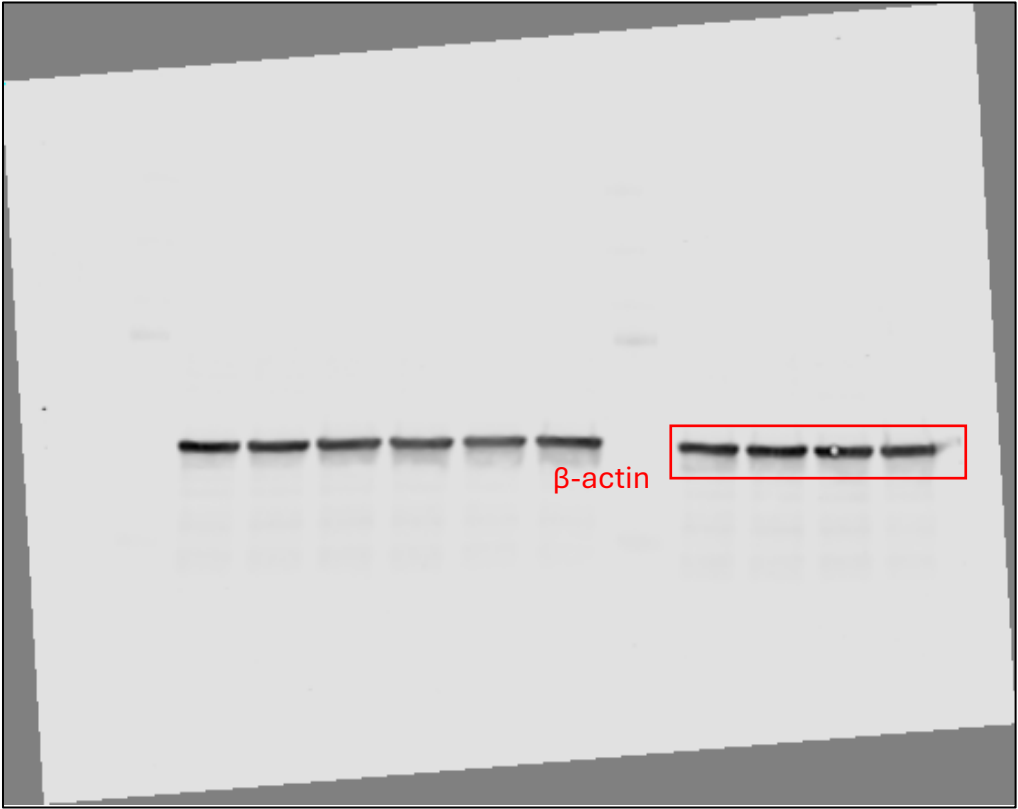

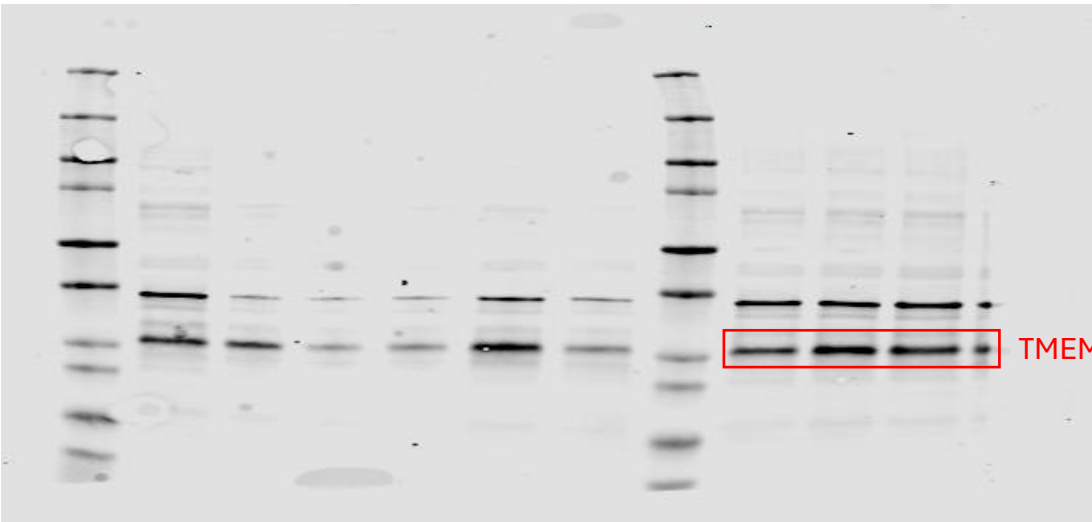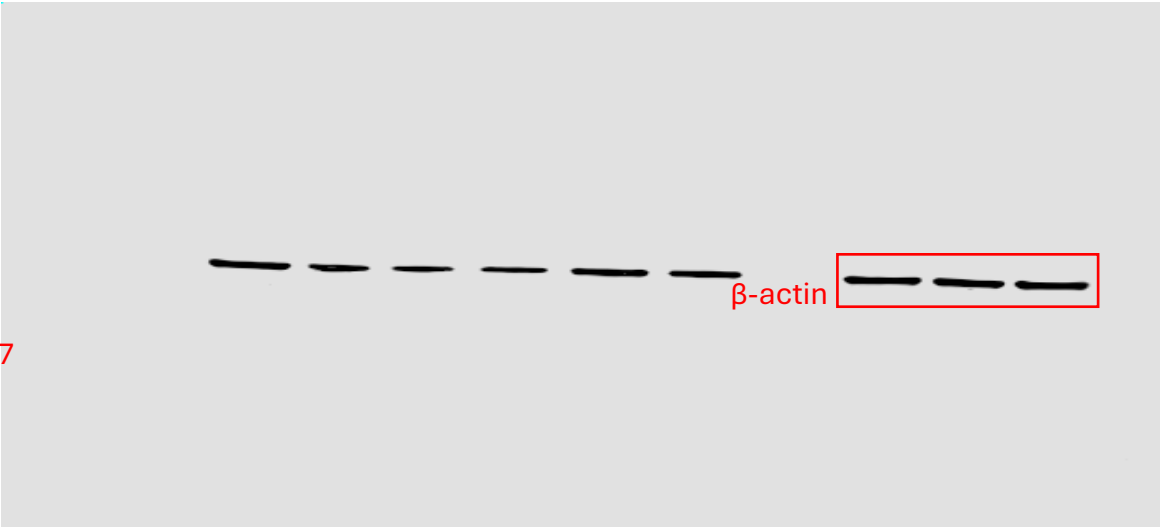

Supplement: Supplementary file 10 — Appendix Figure Source Data [file 44318_2024_319_MOESM10_ESM.zip › EMBOJ-2024-117498-T_SourceDataForAppendix/EMBOJ-2024-117498-T_SourceDataForAppendixFig. S4/Supplementary Figure 4C/README/HT29_all biological replicates_Western.pdf]

## Slide 1
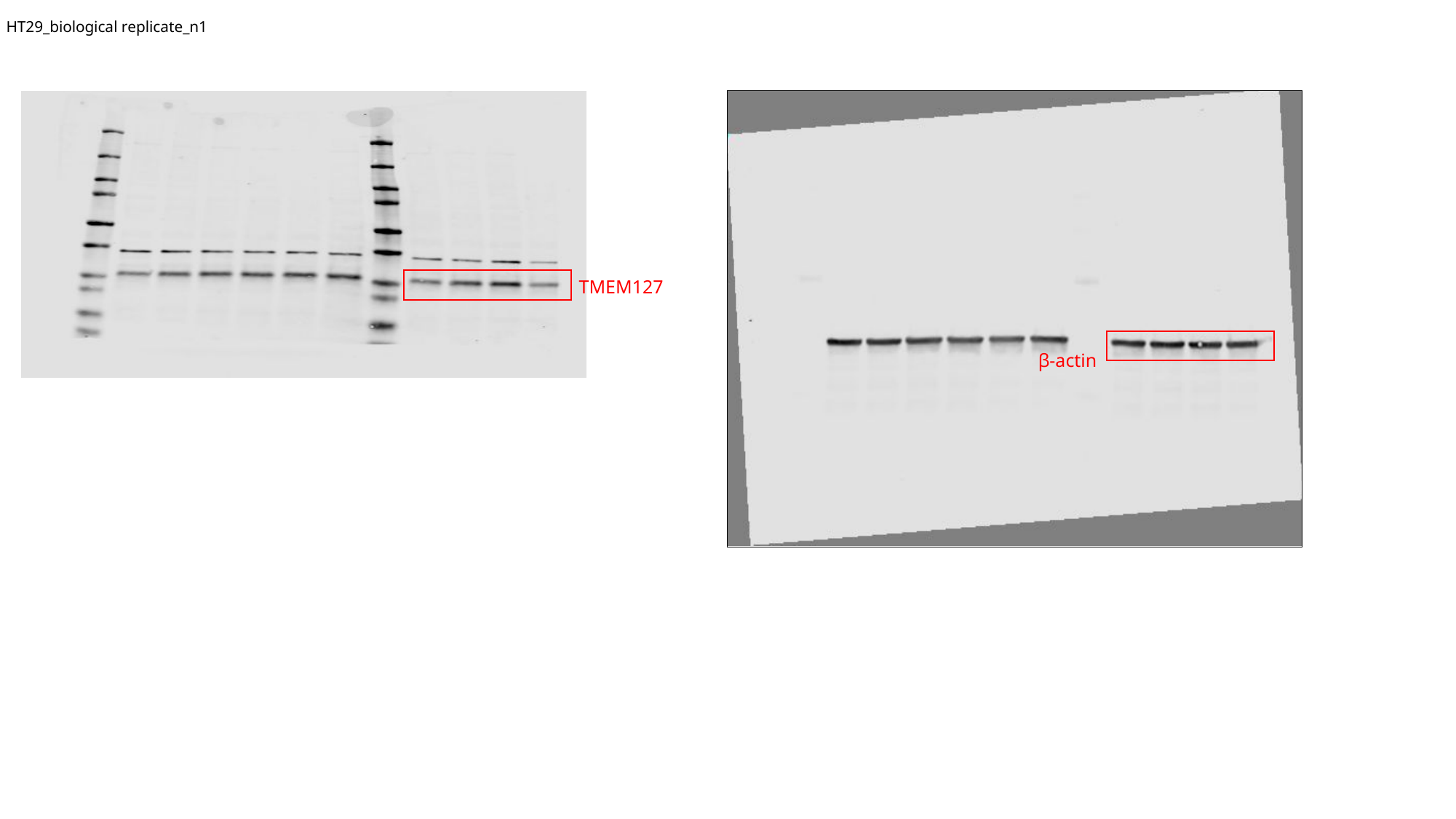

HT29_biological replicate_n1
TMEM127
β-actin

## Slide 2
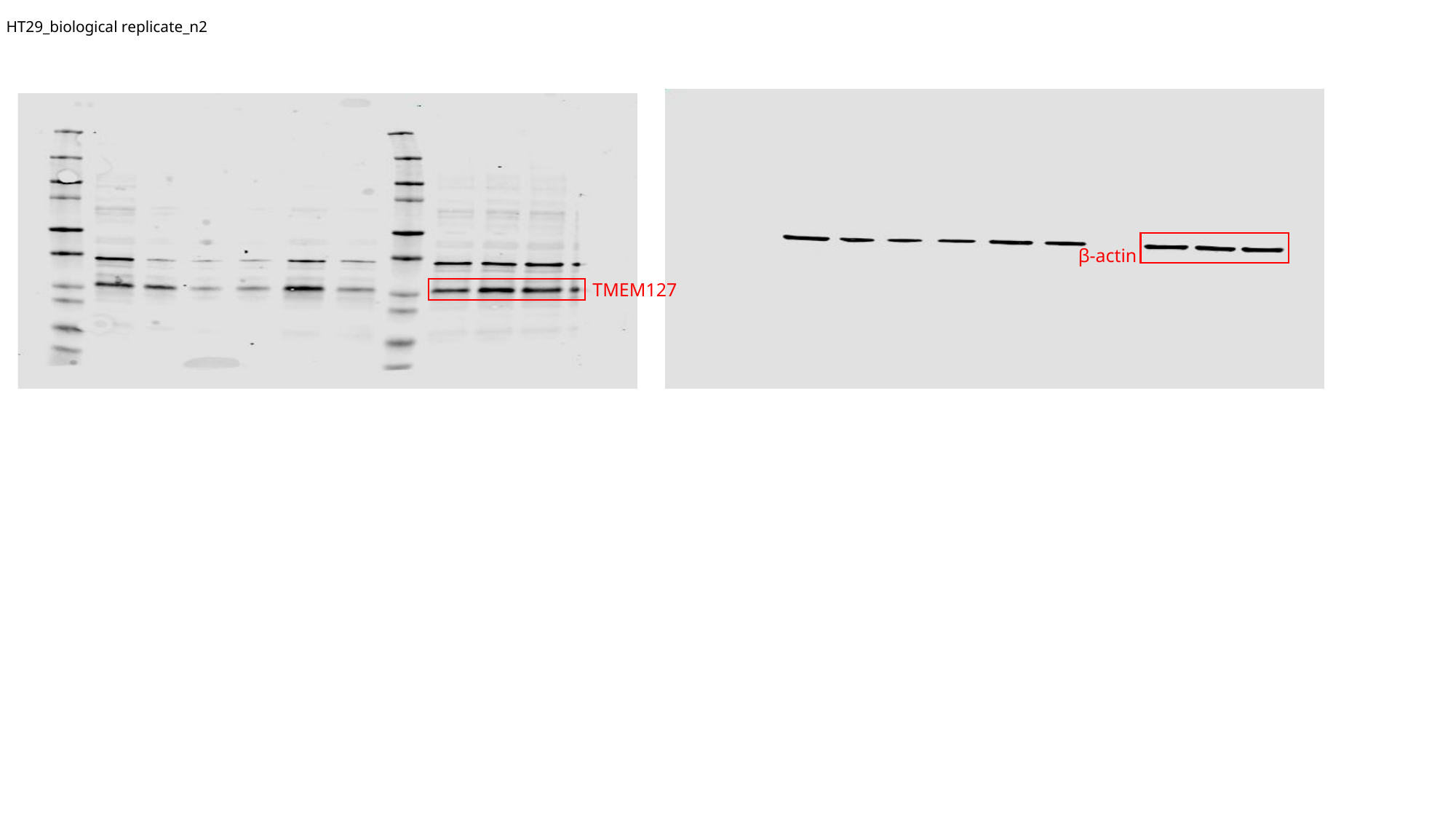

HT29_biological replicate_n2
β-actin
TMEM127

Supplement: Supplementary file 10 — Appendix Figure Source Data [file 44318_2024_319_MOESM10_ESM.zip › EMBOJ-2024-117498-T_SourceDataForAppendix/EMBOJ-2024-117498-T_SourceDataForAppendixFig. S4/Supplementary Figure 4C/README/HT29_all biological replicates_Western.pptx]

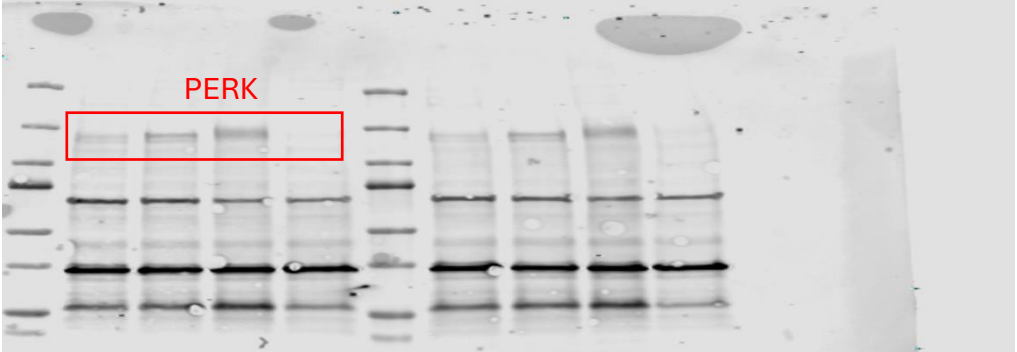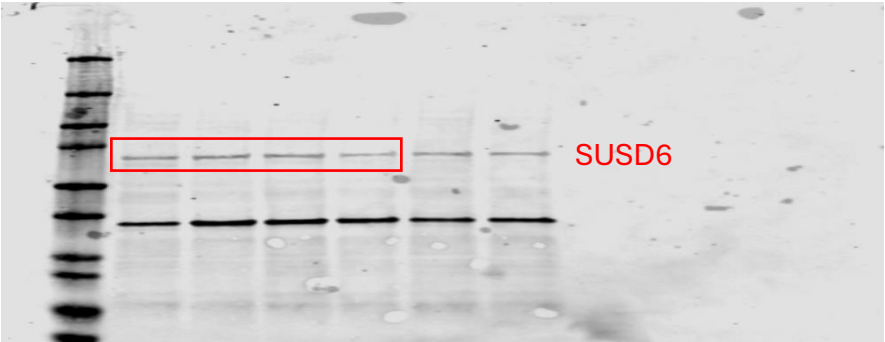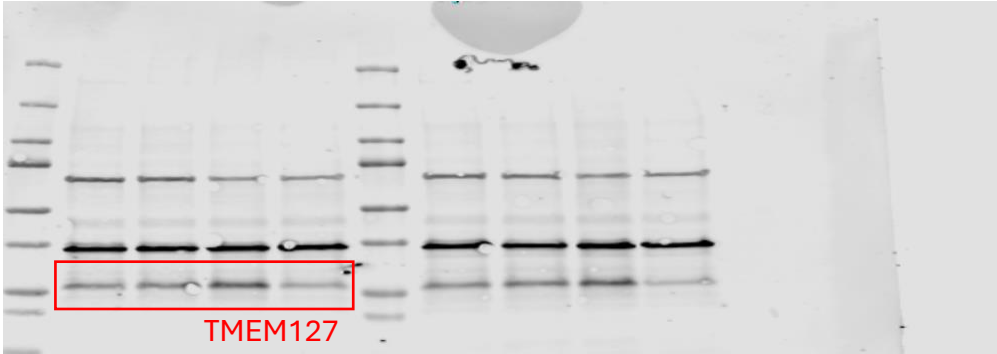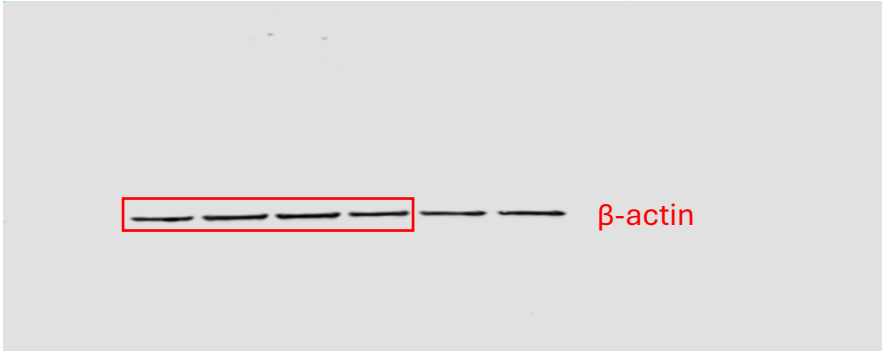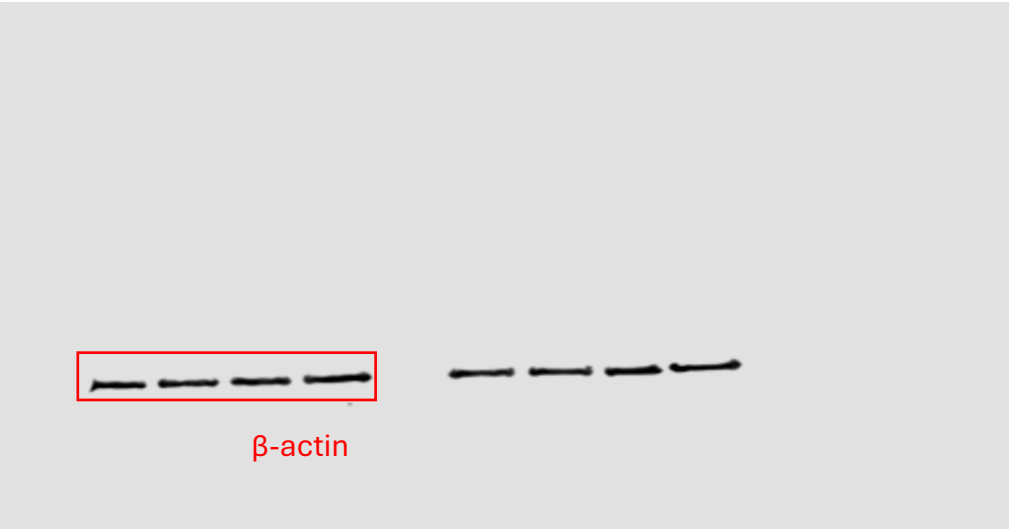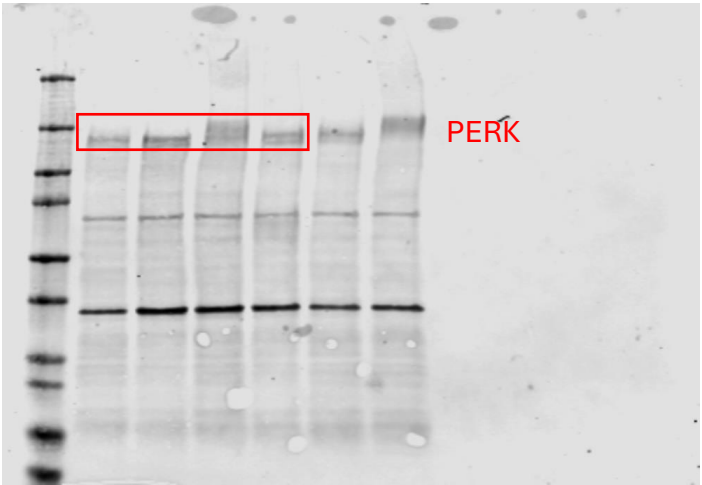

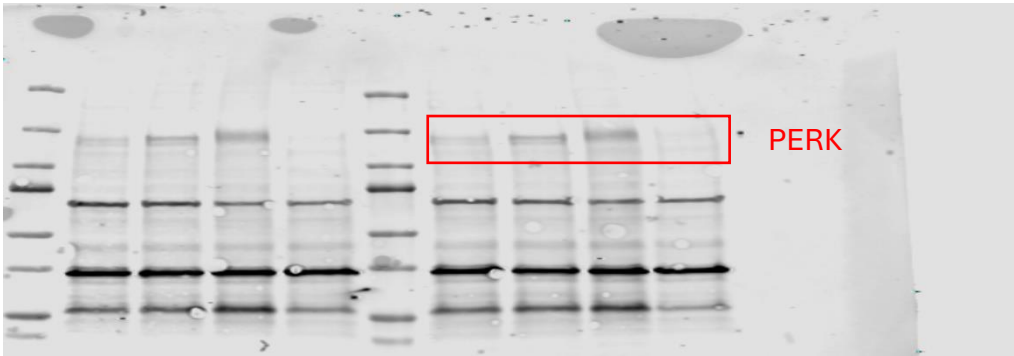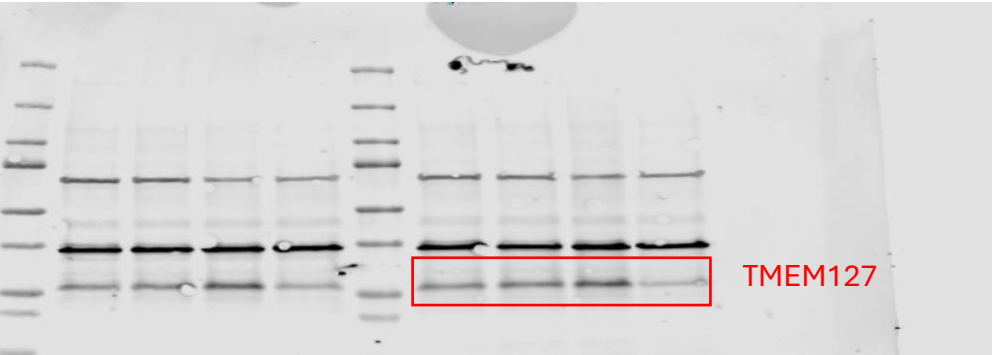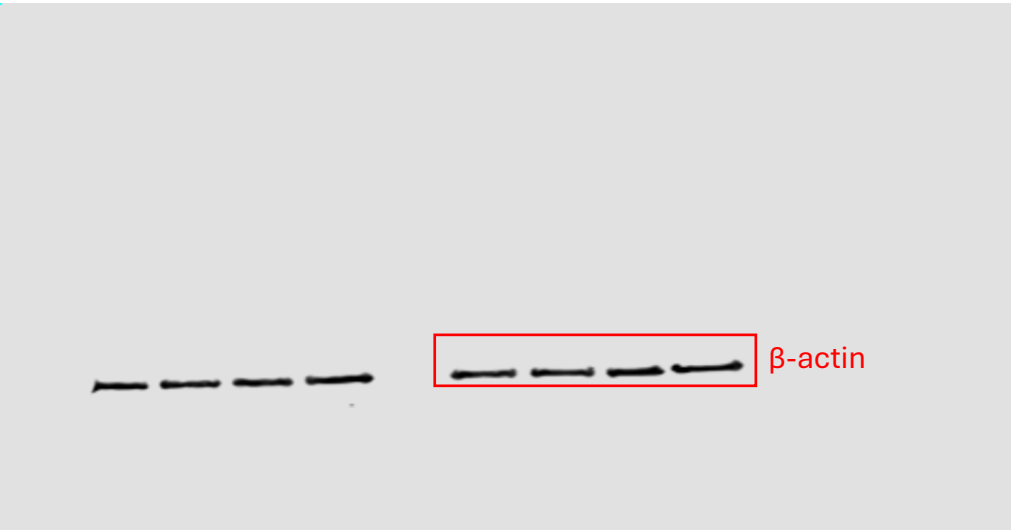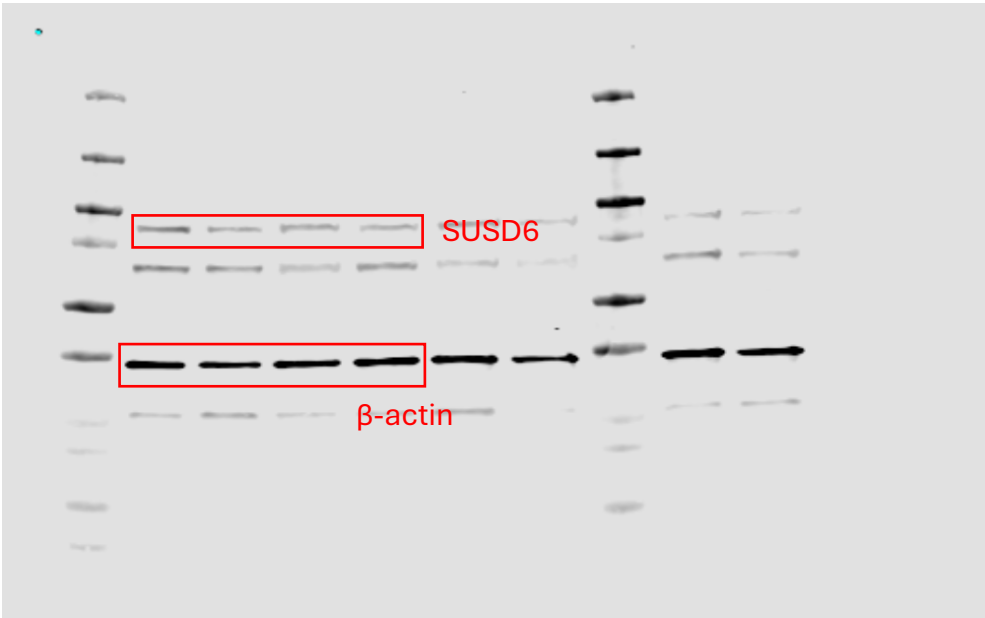

Supplement: Supplementary file 10 — Appendix Figure Source Data [file 44318_2024_319_MOESM10_ESM.zip › EMBOJ-2024-117498-T_SourceDataForAppendix/EMBOJ-2024-117498-T_SourceDataForAppendixFig. S4/Supplementary Figure 4E/HT29_all biological replicates_western.pdf]

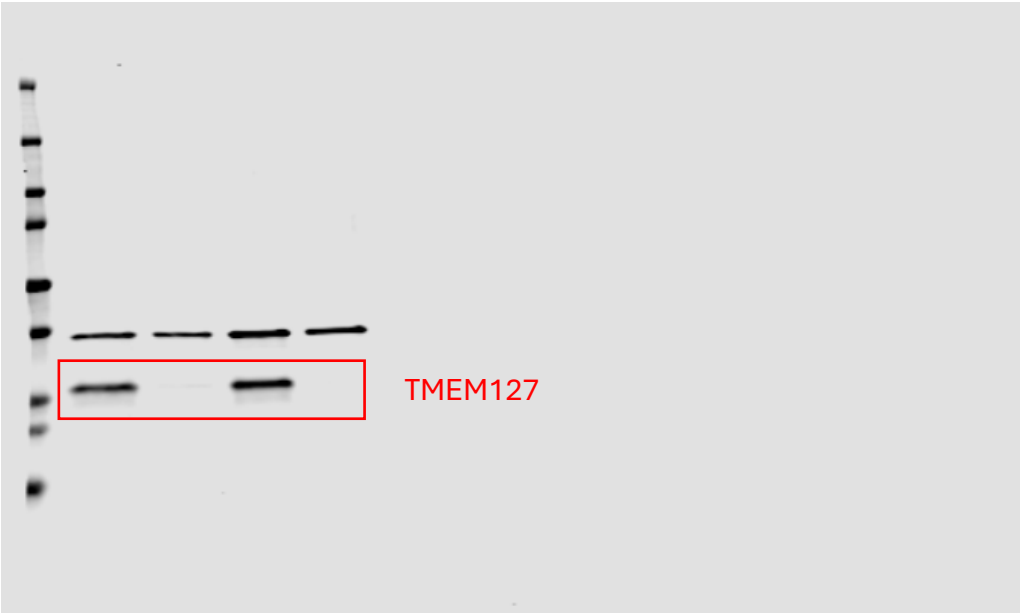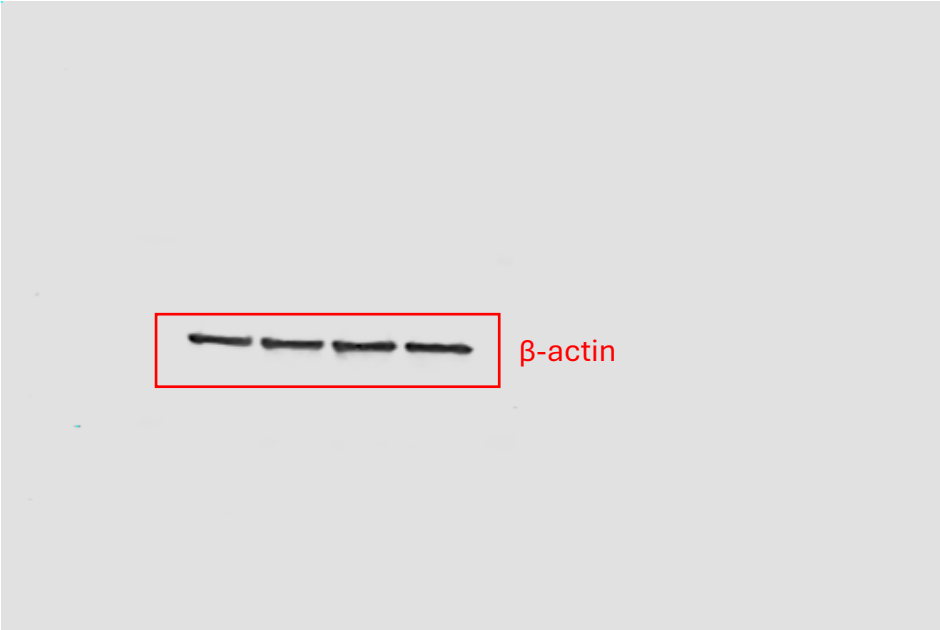

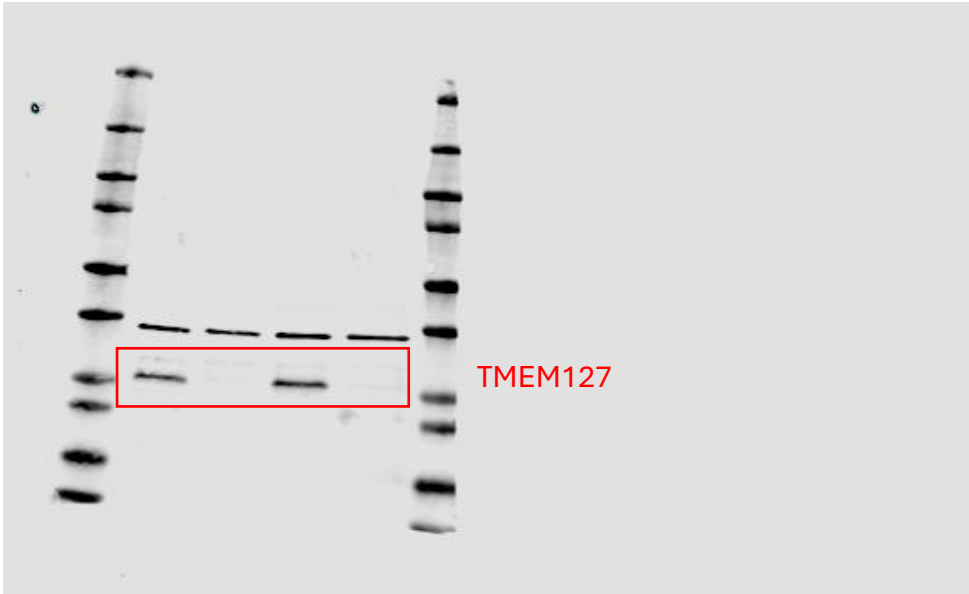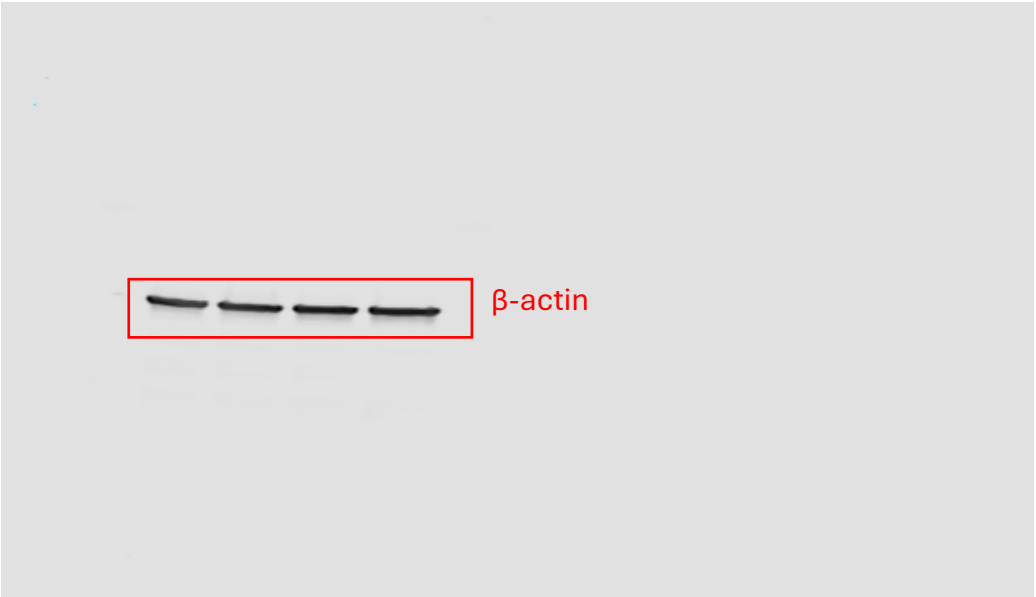

Supplement: Supplementary file 10 — Appendix Figure Source Data [file 44318_2024_319_MOESM10_ESM.zip › EMBOJ-2024-117498-T_SourceDataForAppendix/EMBOJ-2024-117498-T_SourceDataForAppendixFig. S4/Supplementary Figure 4H/README/HT29_all biological replicates_western.pdf]

## Slide 1
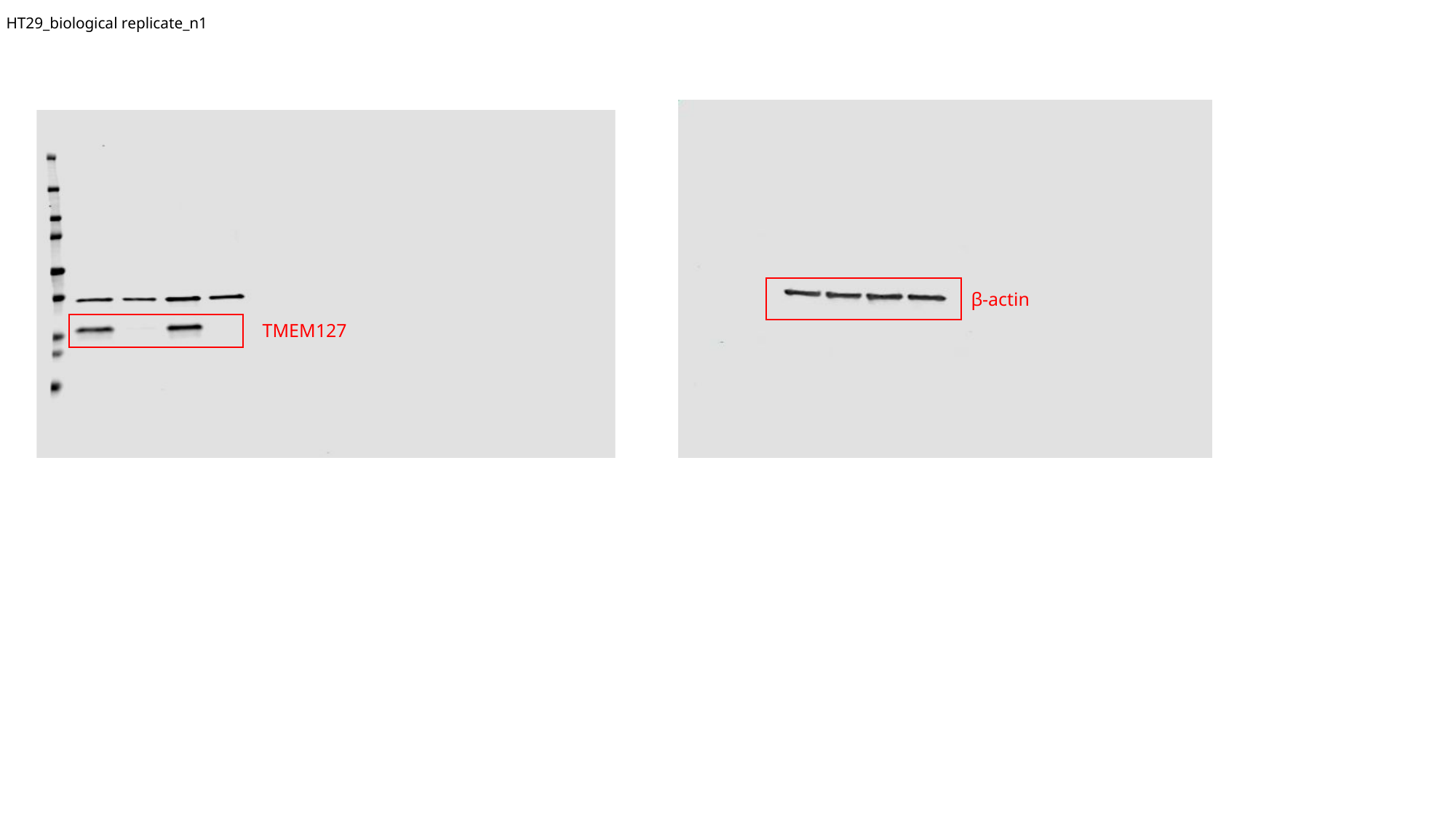

HT29_biological replicate_n1
β-actin
TMEM127

## Slide 2
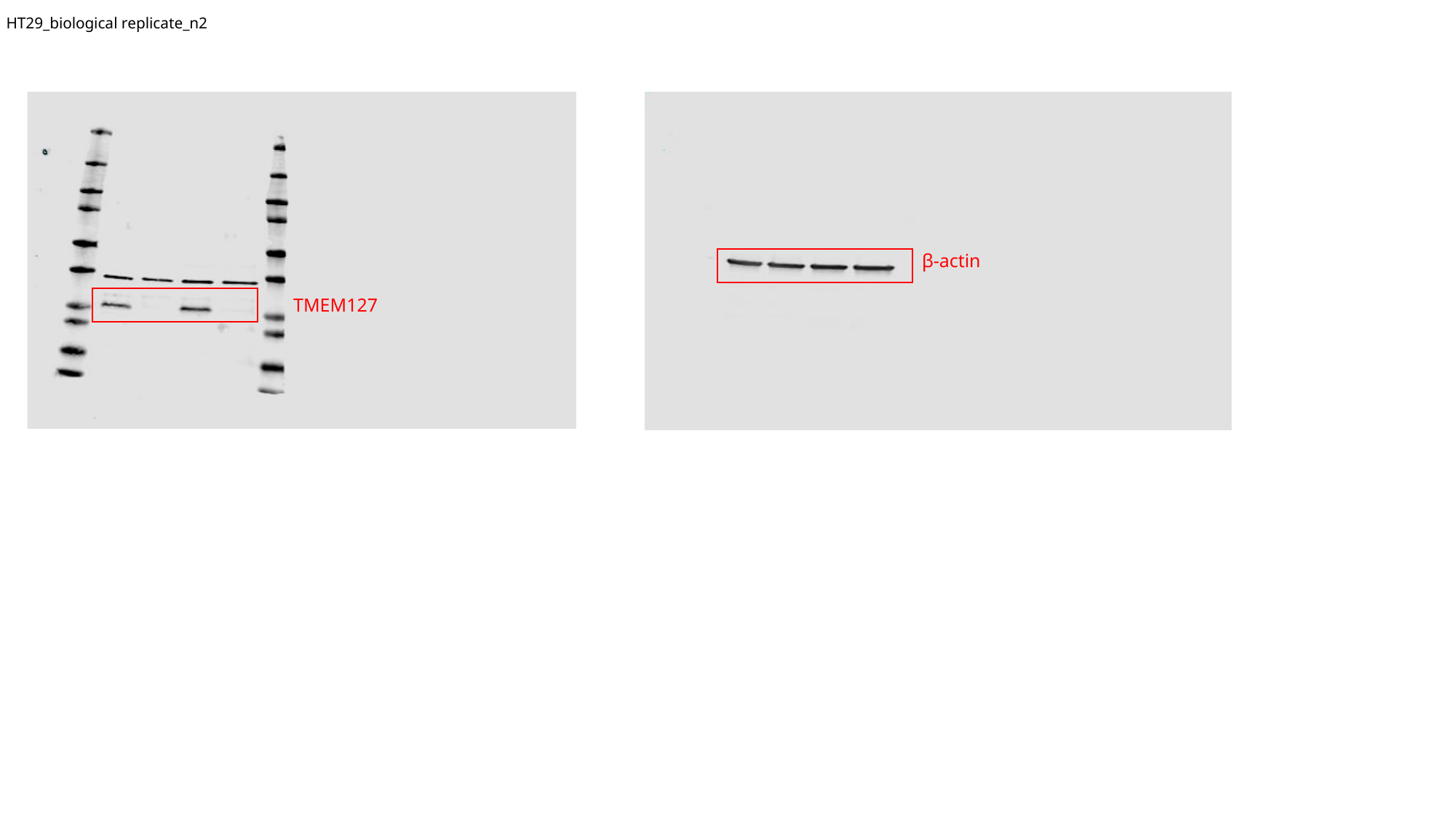

HT29_biological replicate_n2
β-actin
TMEM127

Supplement: Supplementary file 10 — Appendix Figure Source Data [file 44318_2024_319_MOESM10_ESM.zip › EMBOJ-2024-117498-T_SourceDataForAppendix/EMBOJ-2024-117498-T_SourceDataForAppendixFig. S4/Supplementary Figure 4H/README/HT29_all biological replicates_western.pptx]

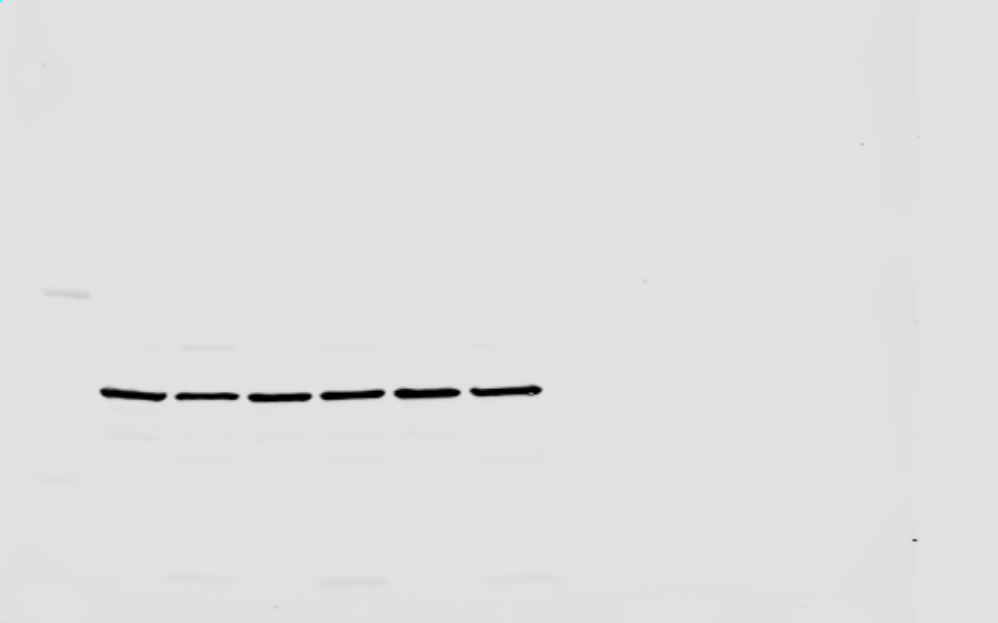

Supplement: Supplementary file 10 — Appendix Figure Source Data [file 44318_2024_319_MOESM10_ESM.zip › EMBOJ-2024-117498-T_SourceDataForAppendix/EMBOJ-2024-117498-T_SourceDataForAppendixFig. S5/Supplementary Figure 5A/HCT116_biological replicate_n1/Anti-actinHCT116 hypoxia-baf samples 12.04.2023 good exp.png]

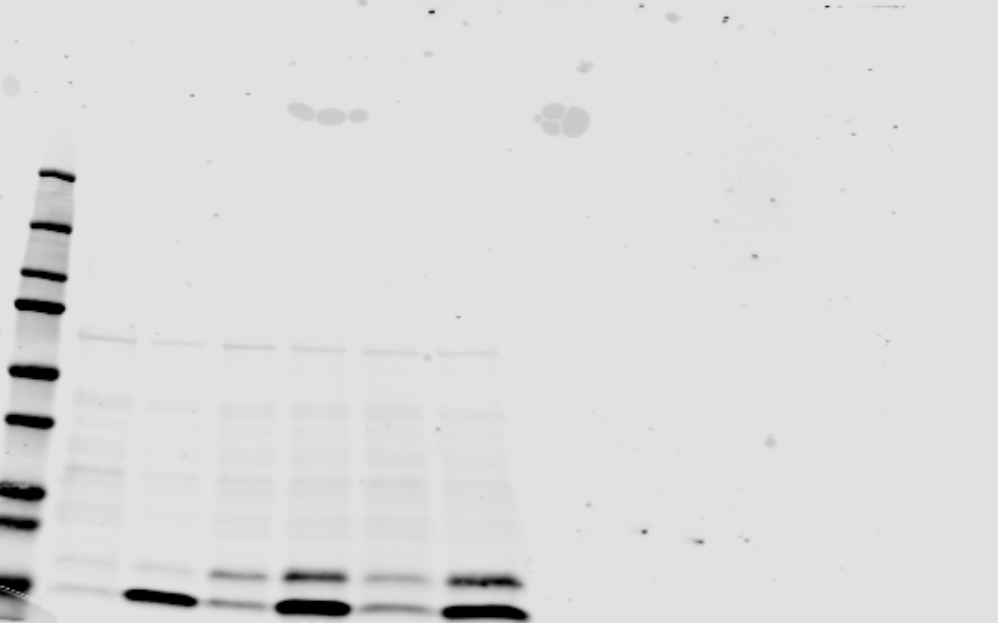

Supplement: Supplementary file 10 — Appendix Figure Source Data [file 44318_2024_319_MOESM10_ESM.zip › EMBOJ-2024-117498-T_SourceDataForAppendix/EMBOJ-2024-117498-T_SourceDataForAppendixFig. S5/Supplementary Figure 5A/HCT116_biological replicate_n1/Anti-LC3 HCT116 hypoxia-baf samples 12.04.2023 good exp.png]

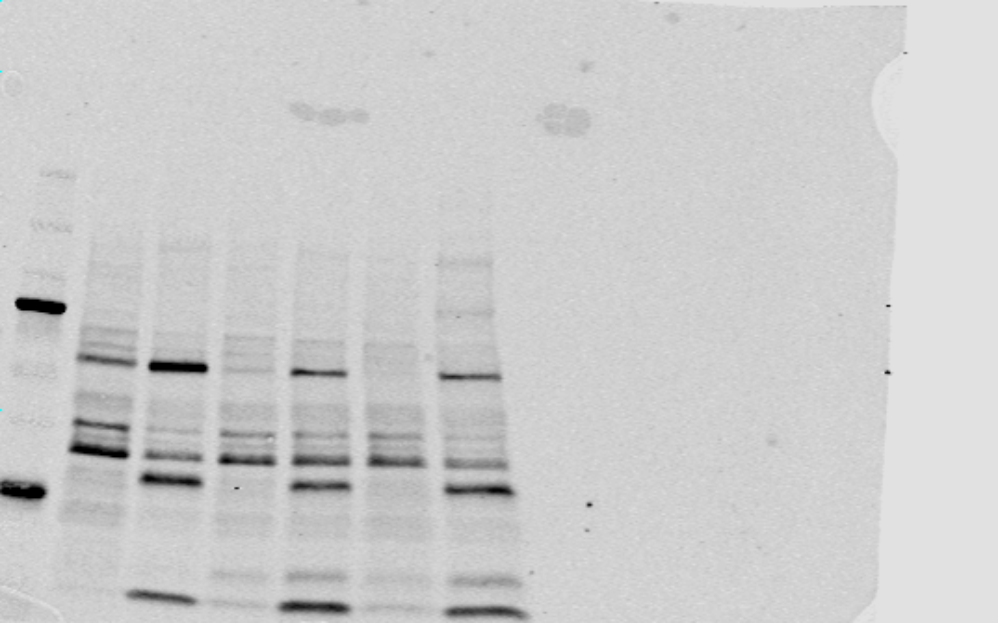

Supplement: Supplementary file 10 — Appendix Figure Source Data [file 44318_2024_319_MOESM10_ESM.zip › EMBOJ-2024-117498-T_SourceDataForAppendix/EMBOJ-2024-117498-T_SourceDataForAppendixFig. S5/Supplementary Figure 5A/HCT116_biological replicate_n1/Anti-p625 HCT116 hypoxia-baf samples 12.04.2023 good exp.png]

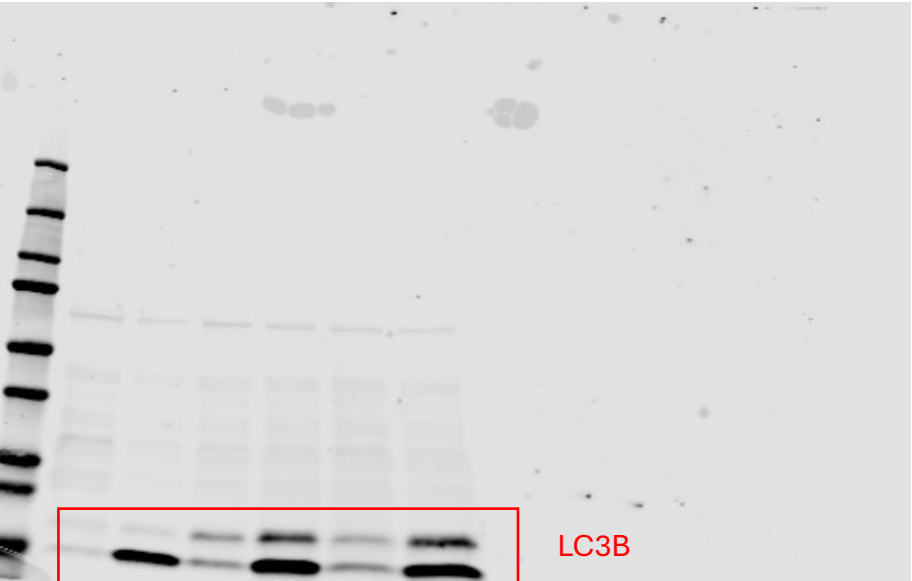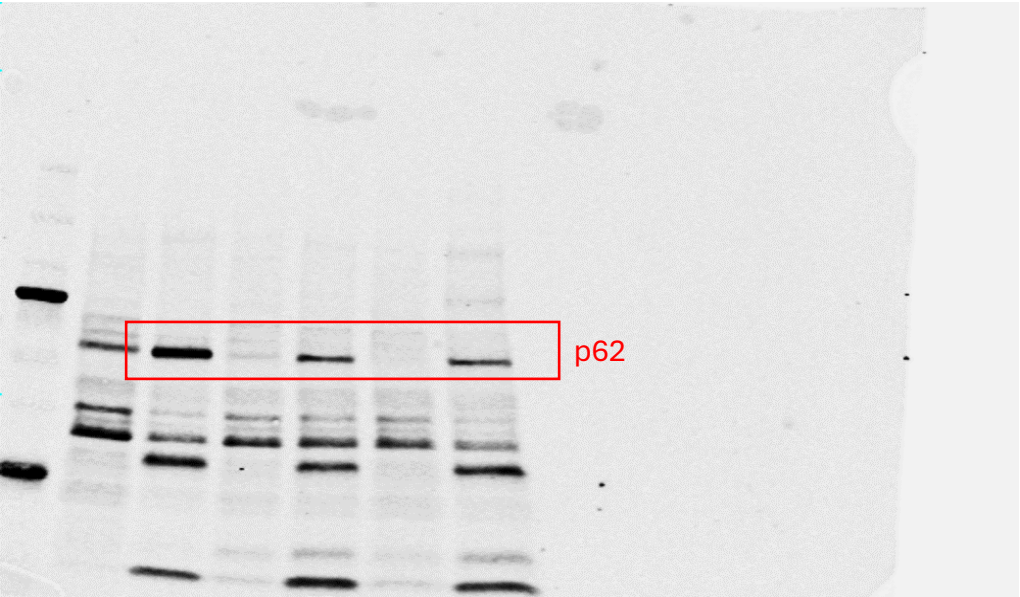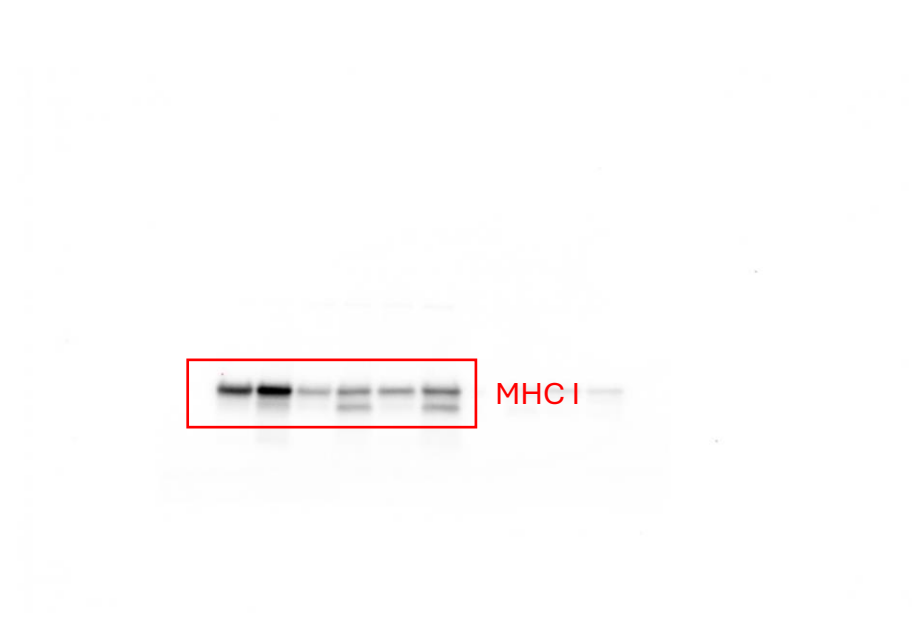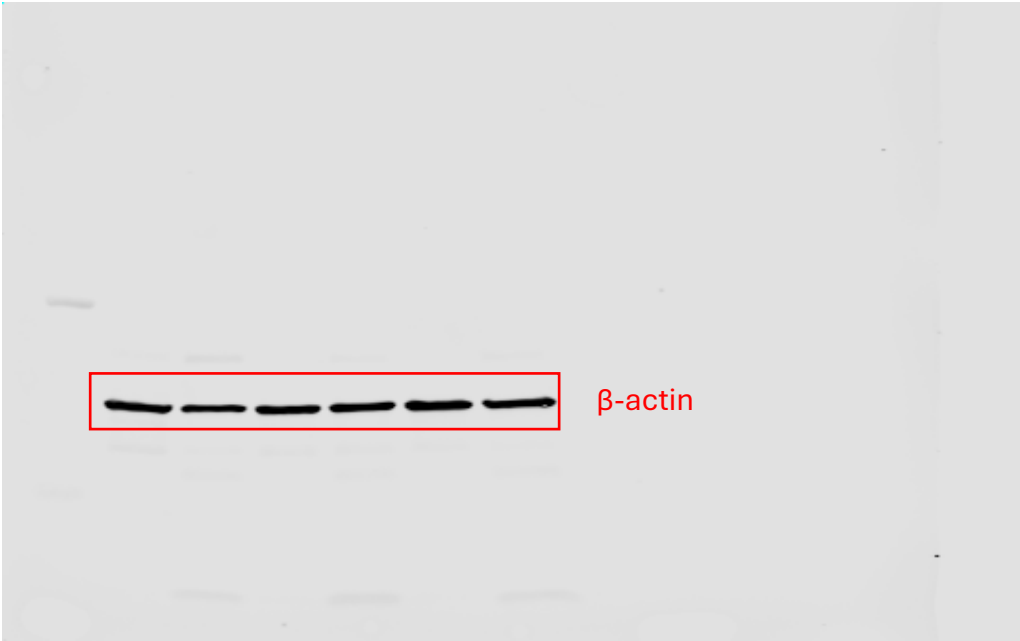

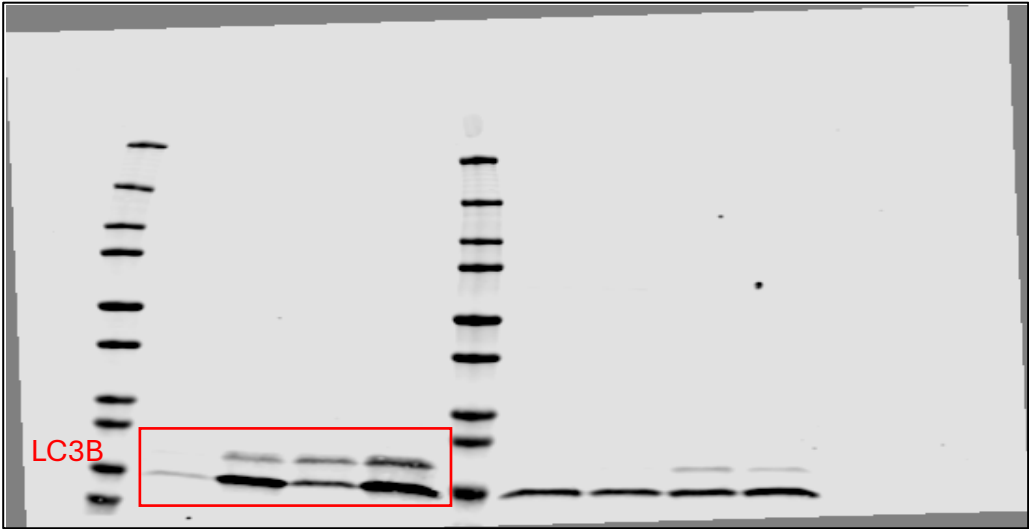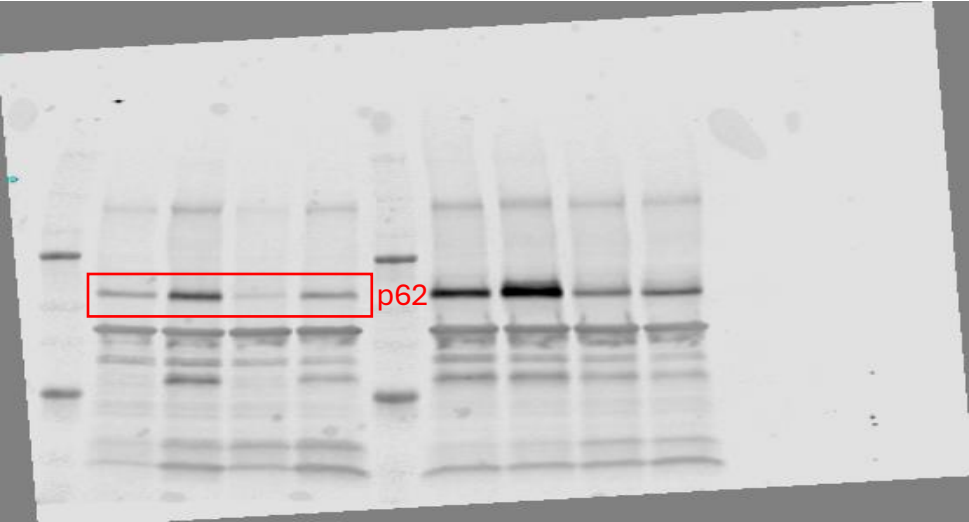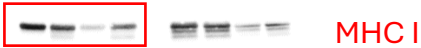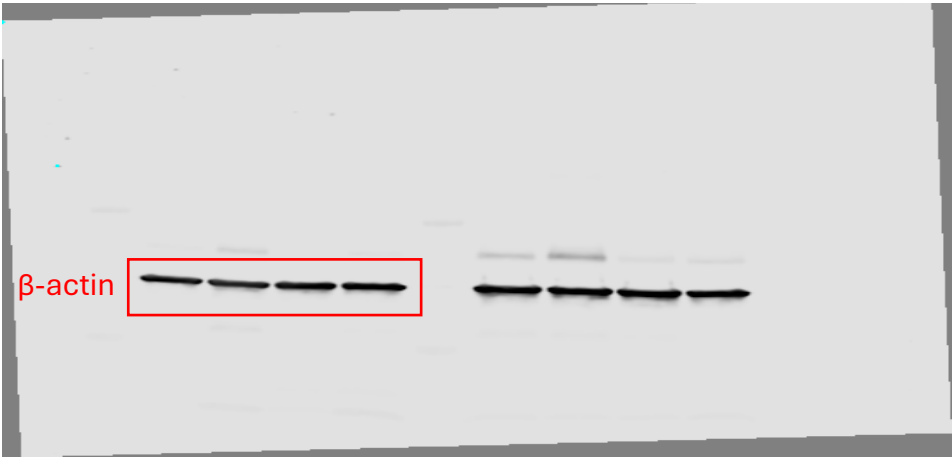

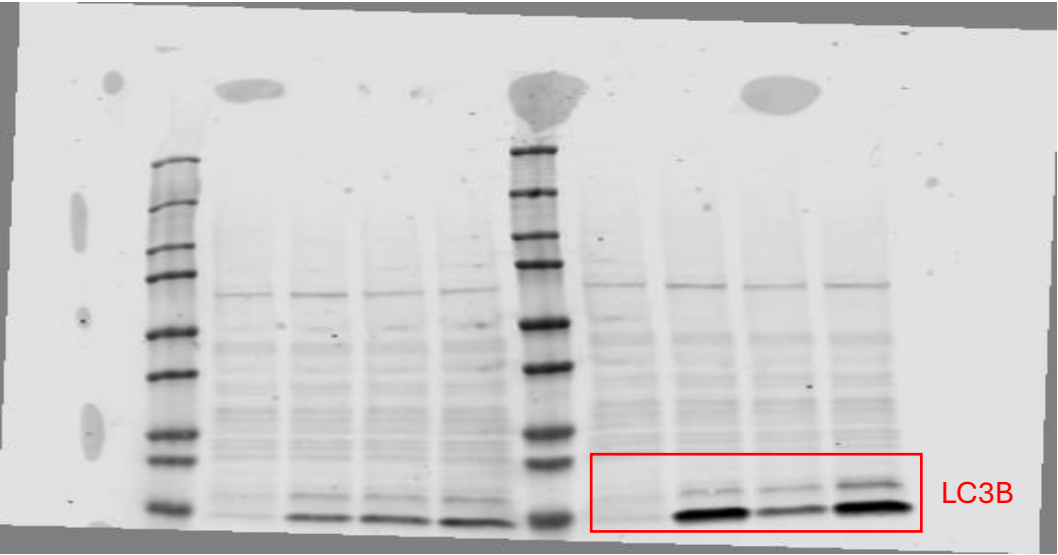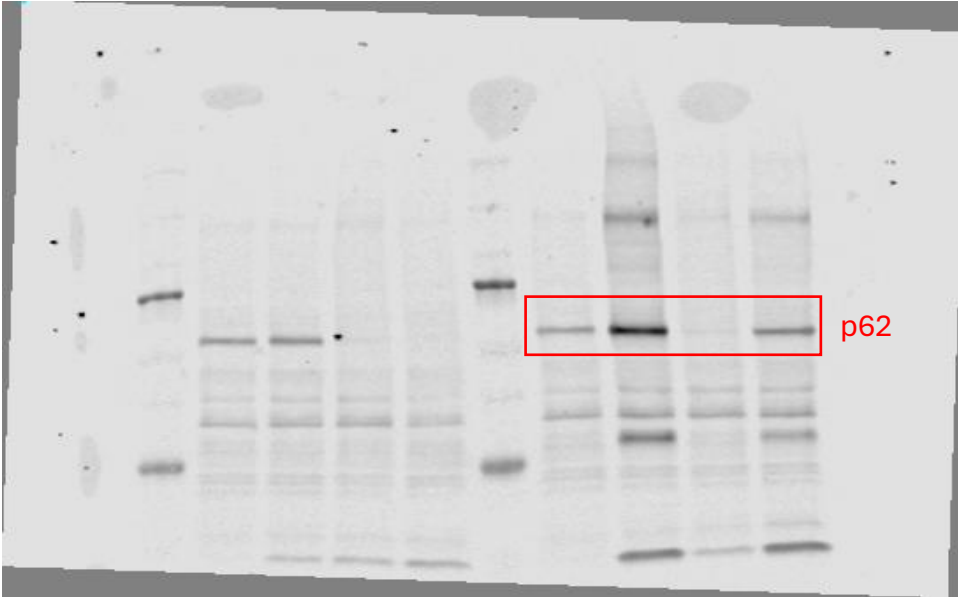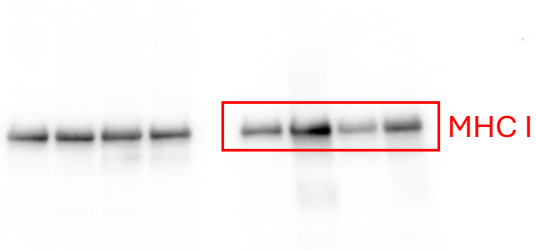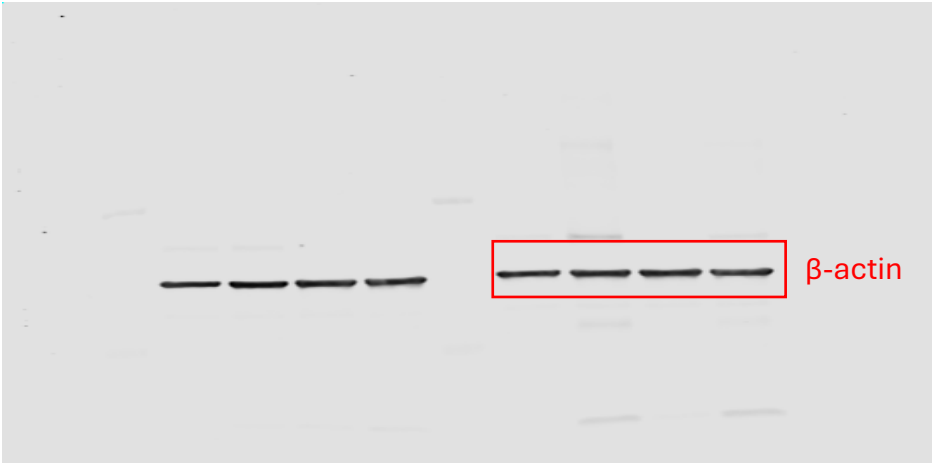

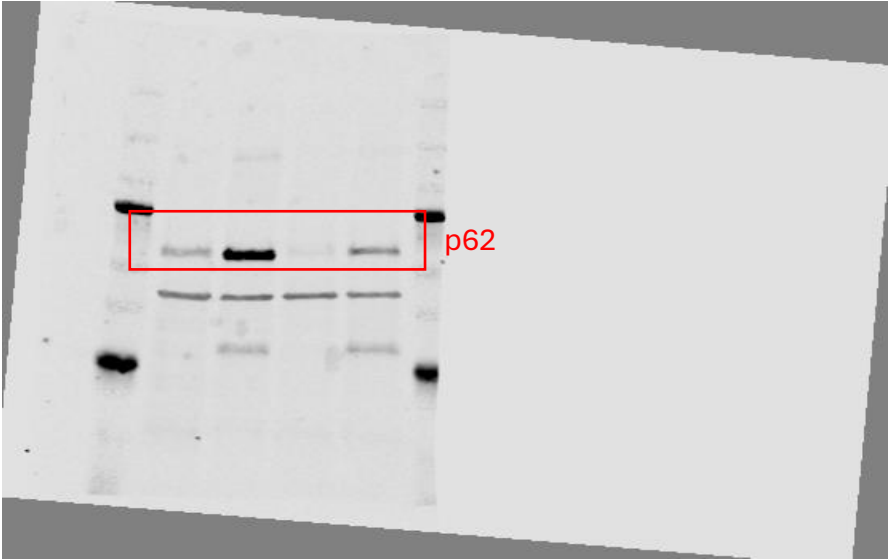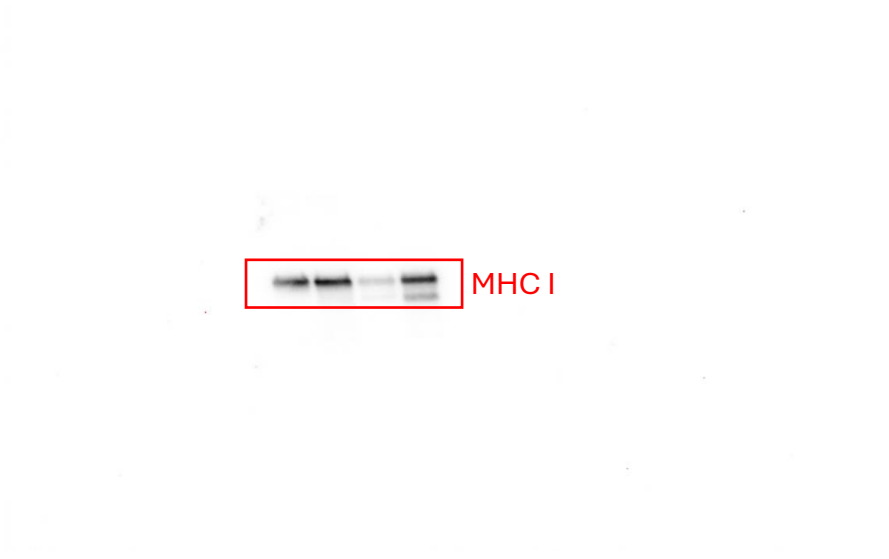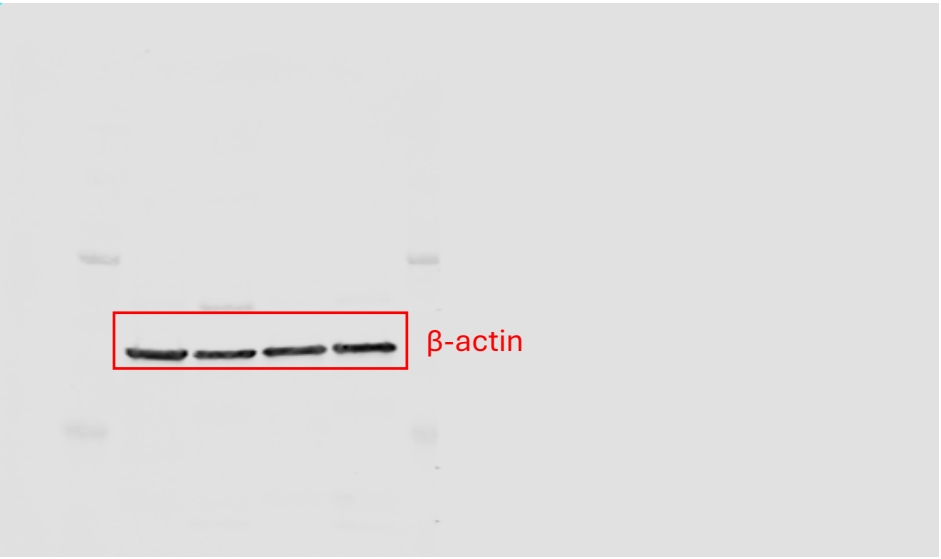

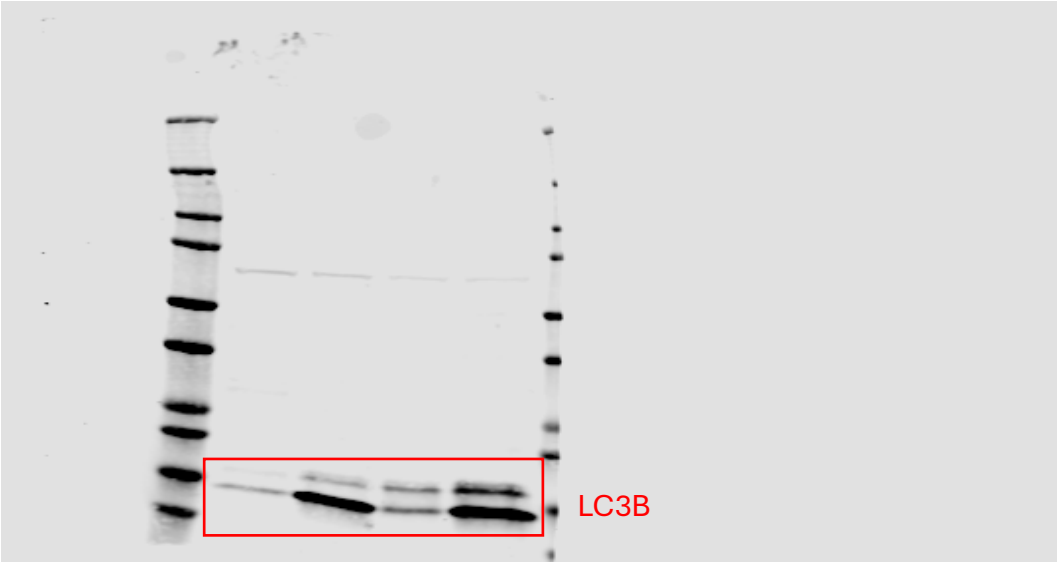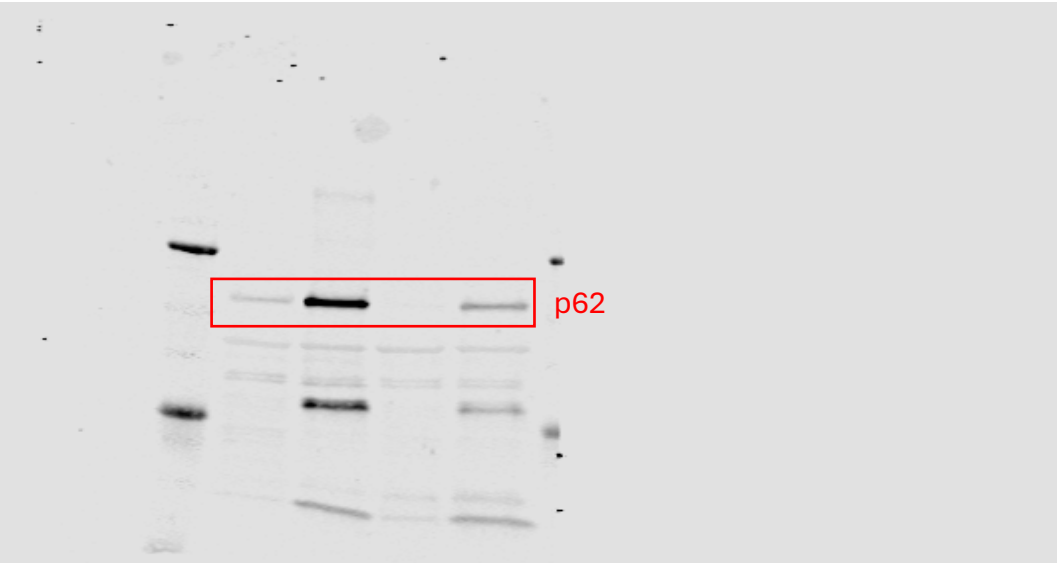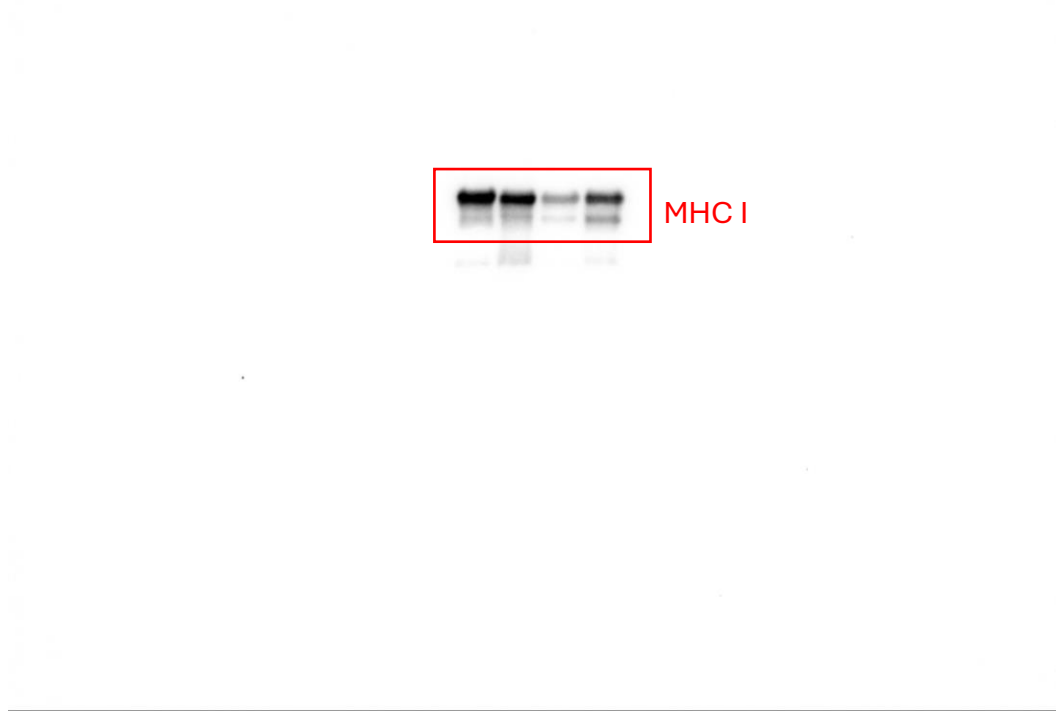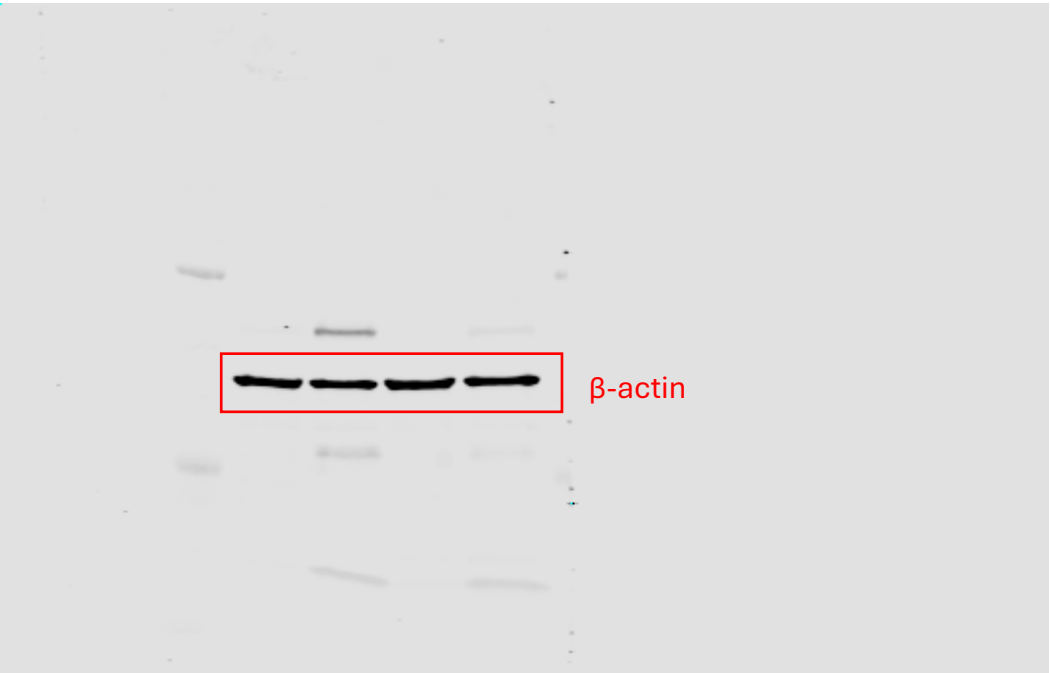

Supplement: Supplementary file 10 — Appendix Figure Source Data [file 44318_2024_319_MOESM10_ESM.zip › EMBOJ-2024-117498-T_SourceDataForAppendix/EMBOJ-2024-117498-T_SourceDataForAppendixFig. S5/Supplementary Figure 5A/README/HCT116_all biological replicates_western.pdf]

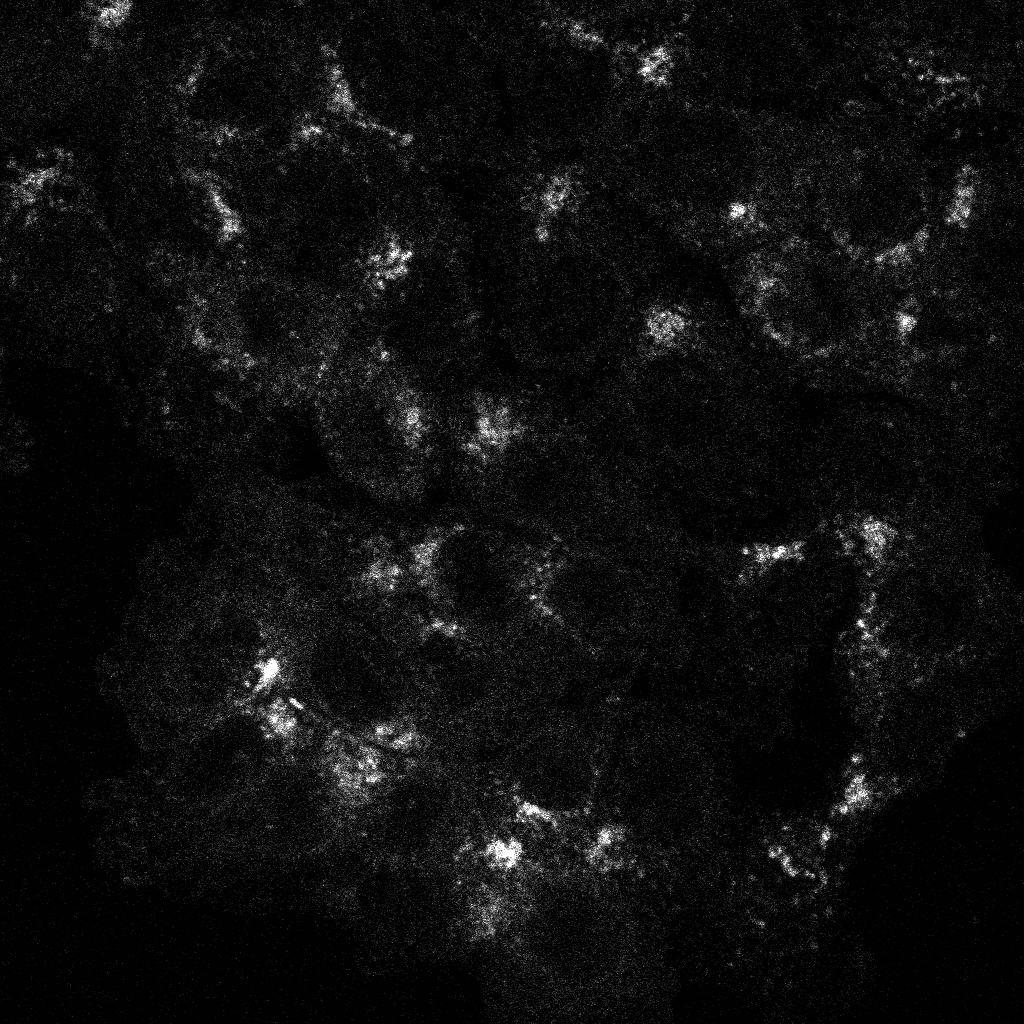

Supplement: Supplementary file 10 — Appendix Figure Source Data [file 44318_2024_319_MOESM10_ESM.zip › EMBOJ-2024-117498-T_SourceDataForAppendix/EMBOJ-2024-117498-T_SourceDataForAppendixFig. S5/Supplementary Figure 5C/HCT116_MHC I LAMP1 DAPI IF staining_hypoxia 16h time point .tif]

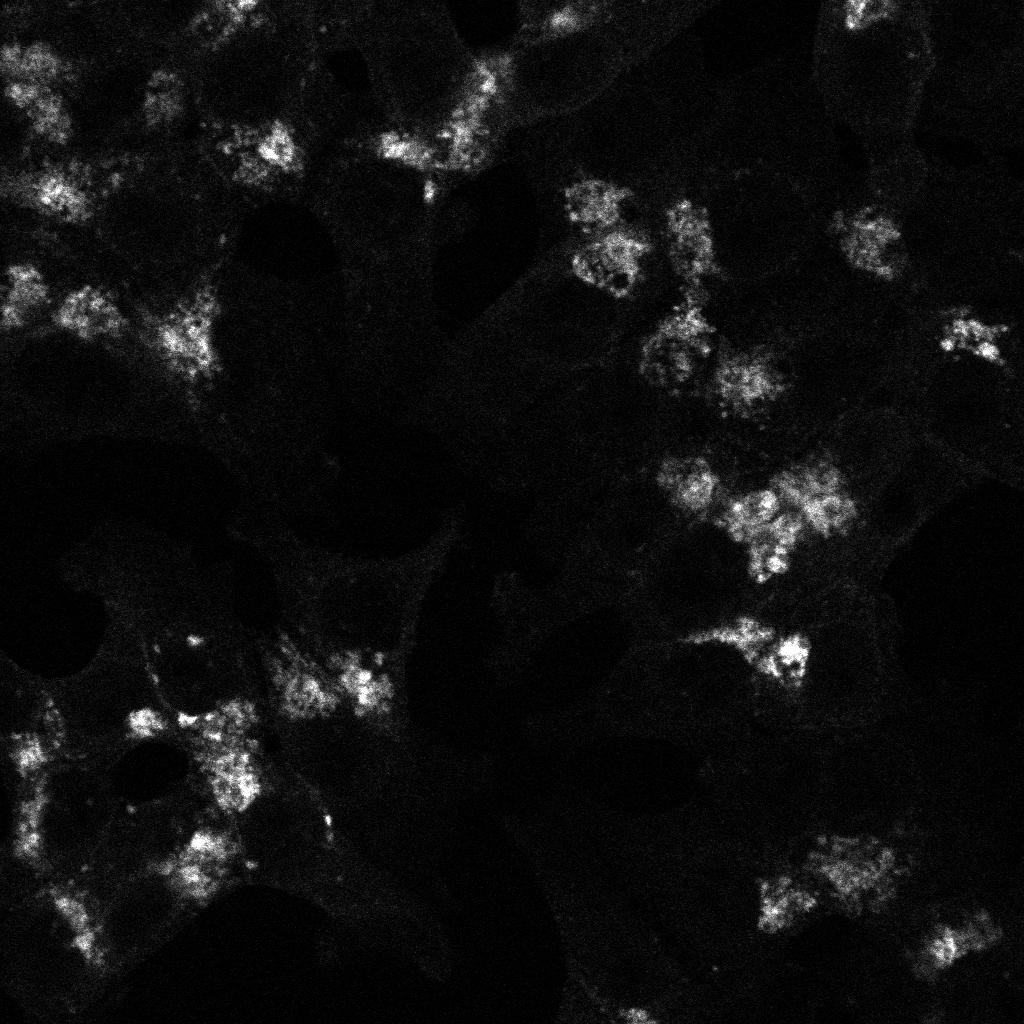

Supplement: Supplementary file 10 — Appendix Figure Source Data [file 44318_2024_319_MOESM10_ESM.zip › EMBOJ-2024-117498-T_SourceDataForAppendix/EMBOJ-2024-117498-T_SourceDataForAppendixFig. S5/Supplementary Figure 5C/HCT116_MHC I LAMP1 DAPI IF staining_hypoxia 16h time point BafA1 .tif]

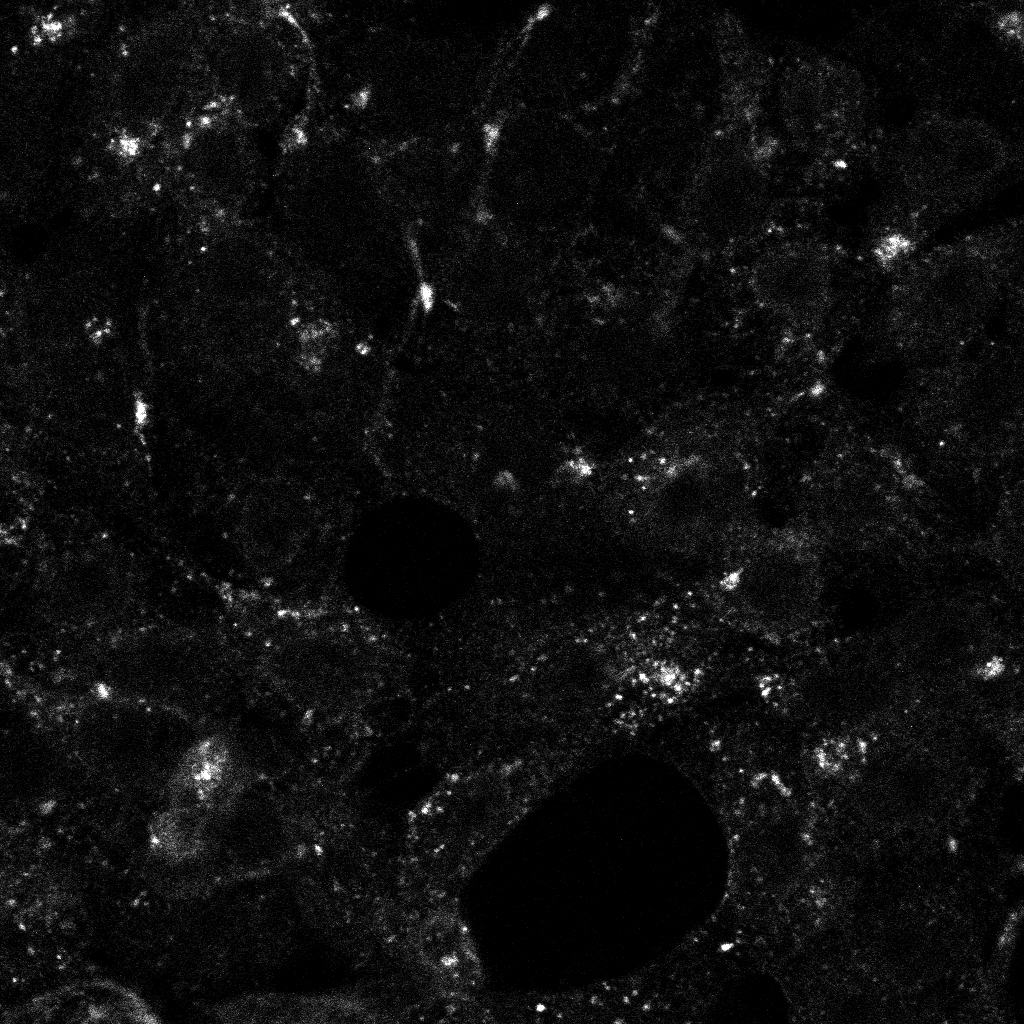

Supplement: Supplementary file 10 — Appendix Figure Source Data [file 44318_2024_319_MOESM10_ESM.zip › EMBOJ-2024-117498-T_SourceDataForAppendix/EMBOJ-2024-117498-T_SourceDataForAppendixFig. S5/Supplementary Figure 5C/HCT116_MHC I LAMP1 DAPI IF staining_hypoxia 24h time point .tif]

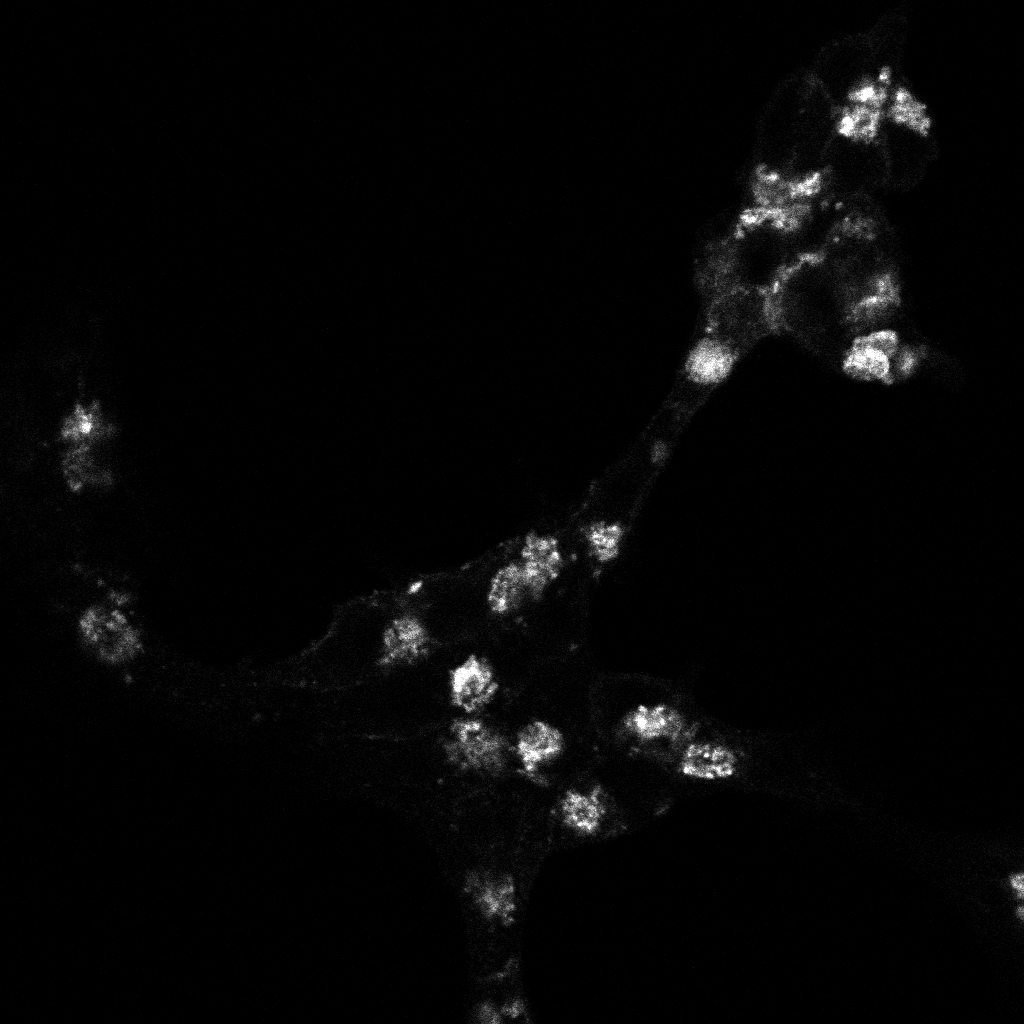

Supplement: Supplementary file 10 — Appendix Figure Source Data [file 44318_2024_319_MOESM10_ESM.zip › EMBOJ-2024-117498-T_SourceDataForAppendix/EMBOJ-2024-117498-T_SourceDataForAppendixFig. S5/Supplementary Figure 5C/HCT116_MHC I LAMP1 DAPI IF staining_hypoxia 24h time point BafA1 .tif]

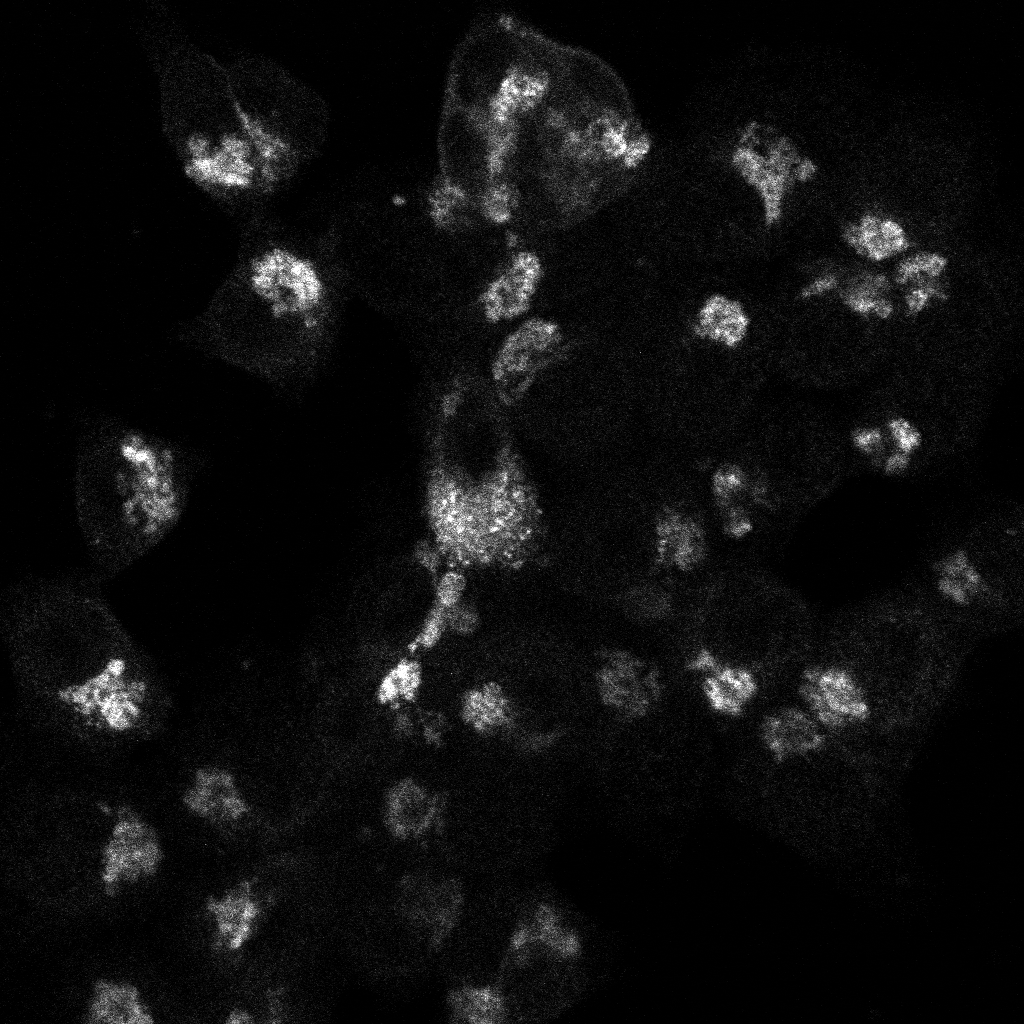

Supplement: Supplementary file 10 — Appendix Figure Source Data [file 44318_2024_319_MOESM10_ESM.zip › EMBOJ-2024-117498-T_SourceDataForAppendix/EMBOJ-2024-117498-T_SourceDataForAppendixFig. S5/Supplementary Figure 5C/HCT116_MHC I LAMP1 DAPI IF staining_normoxia BafA1.tif]

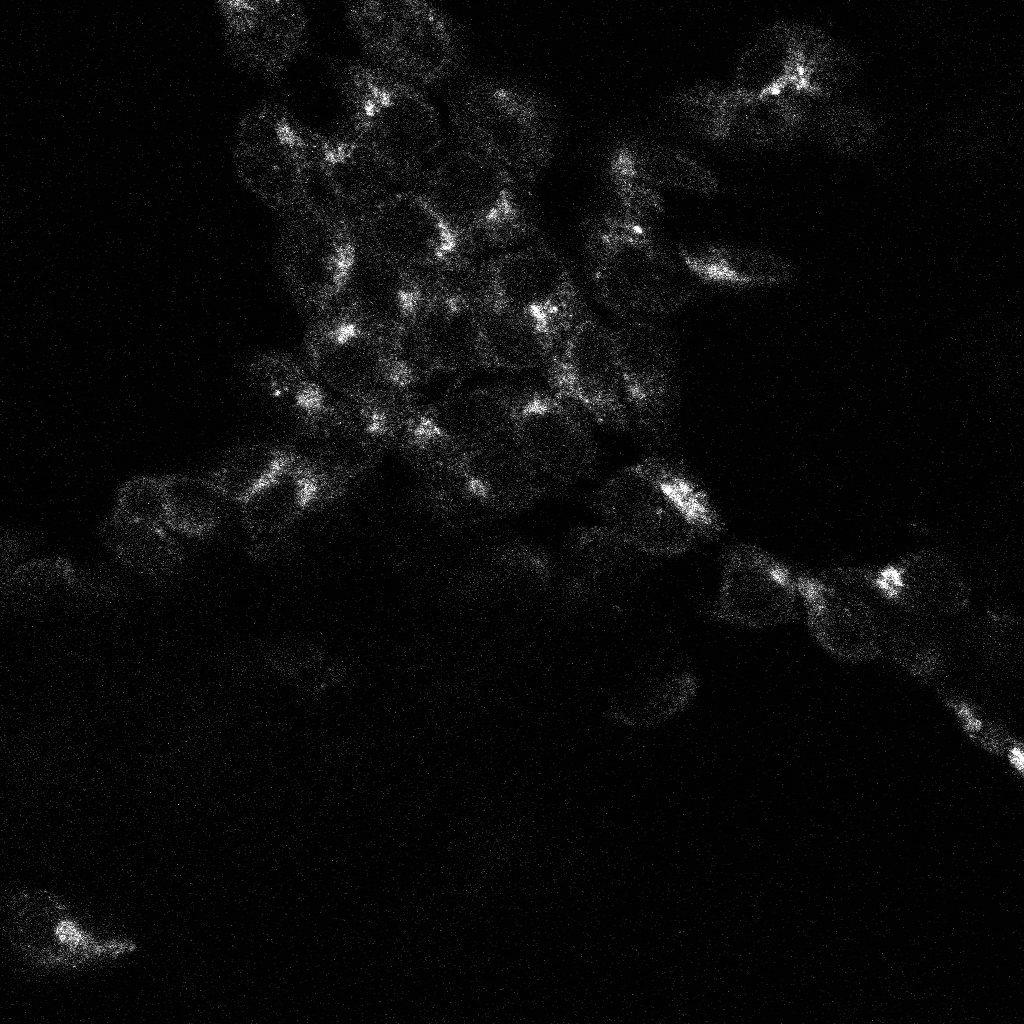

Supplement: Supplementary file 10 — Appendix Figure Source Data [file 44318_2024_319_MOESM10_ESM.zip › EMBOJ-2024-117498-T_SourceDataForAppendix/EMBOJ-2024-117498-T_SourceDataForAppendixFig. S5/Supplementary Figure 5C/HCT116_MHC I LAMP1 DAPI IF staining_normoxia veh .tif]

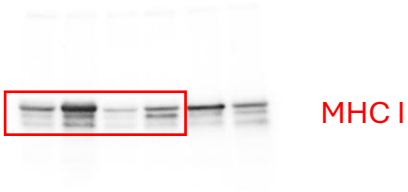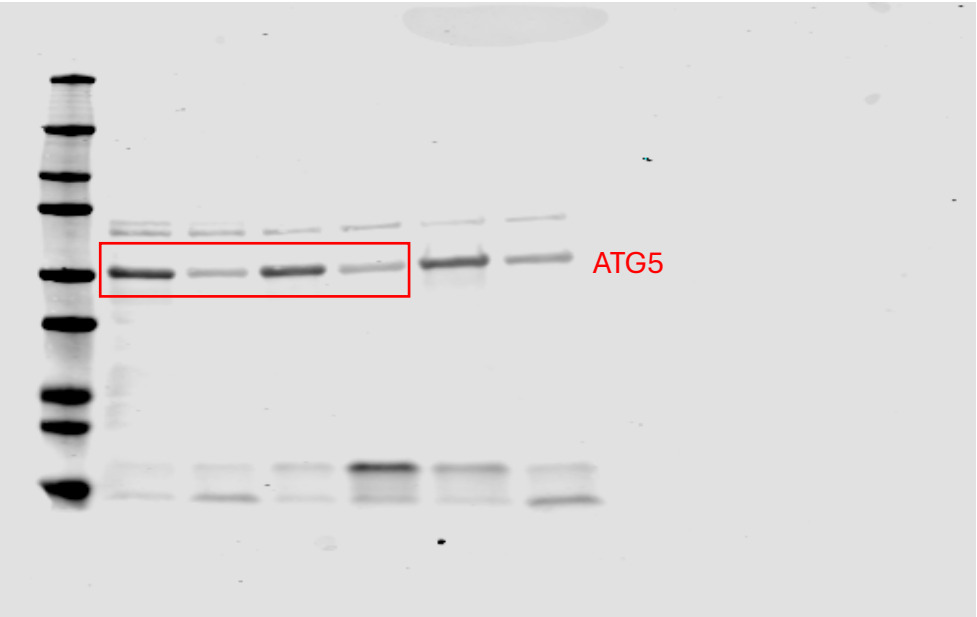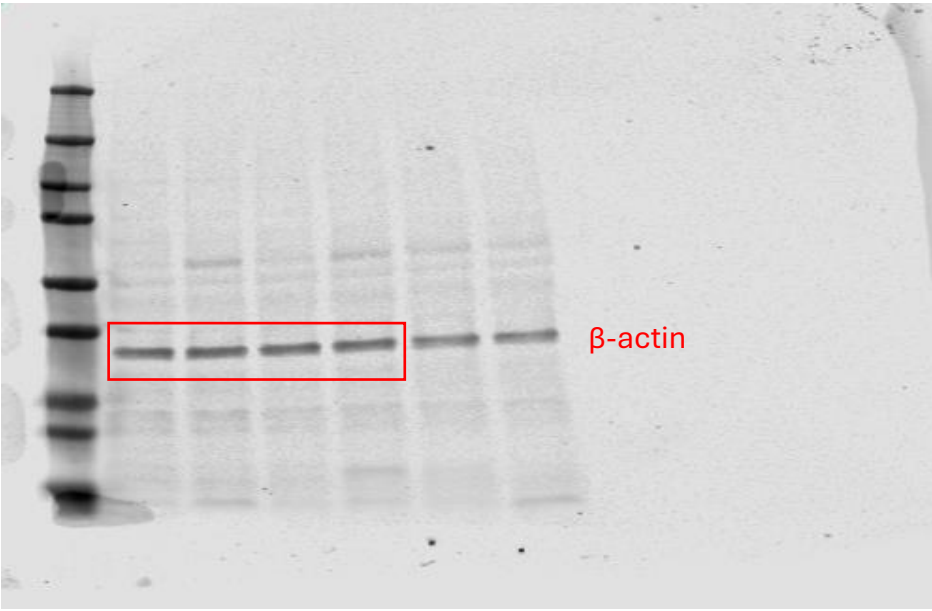

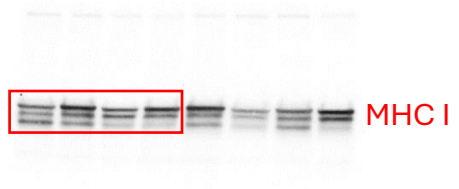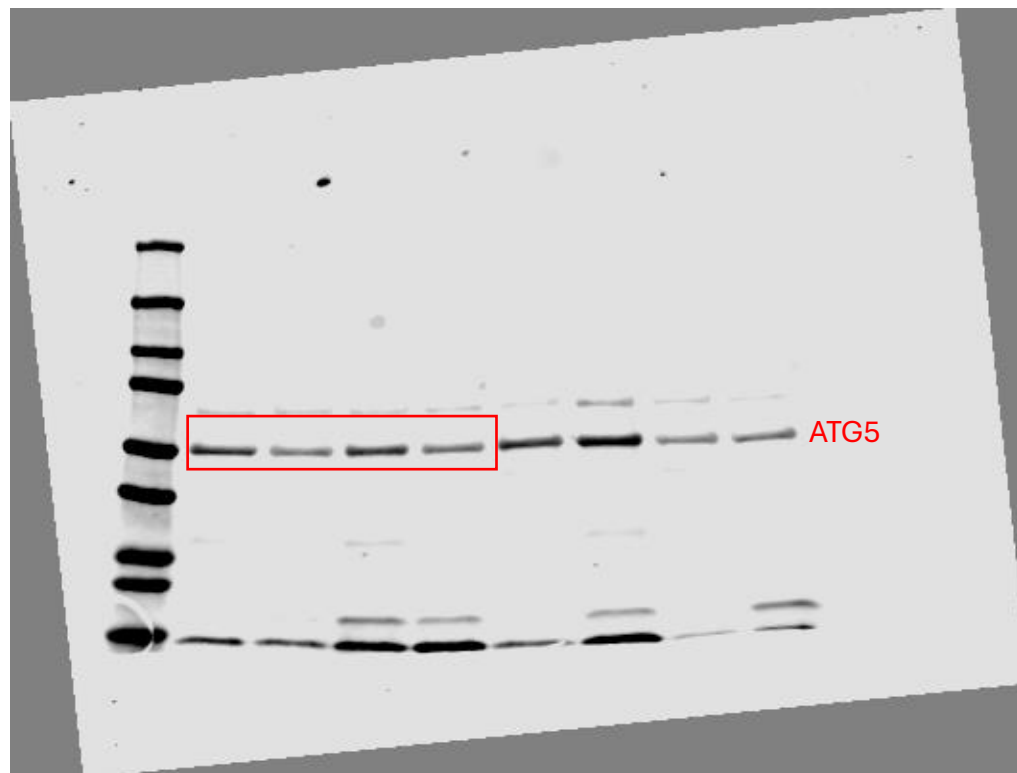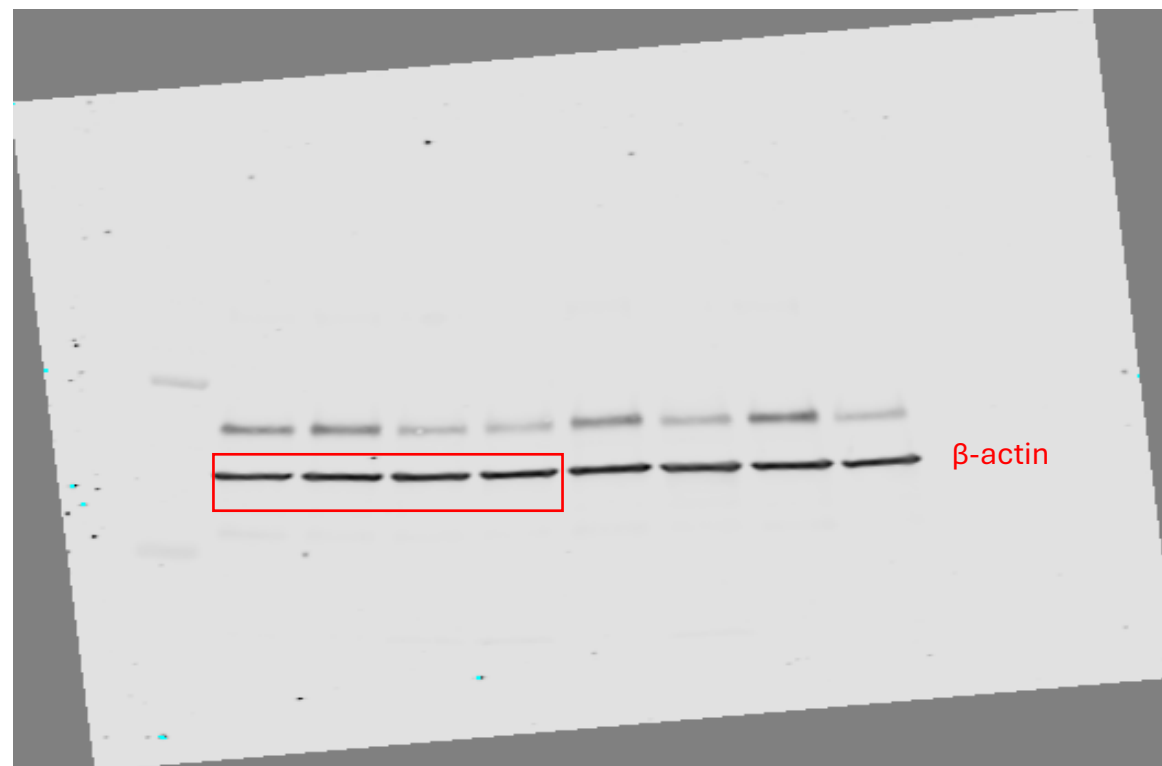

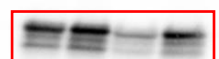

MHC I

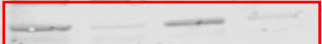

ATG5

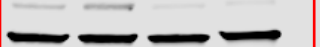

$\beta$ -actin

Supplement: Supplementary file 10 — Appendix Figure Source Data [file 44318_2024_319_MOESM10_ESM.zip › EMBOJ-2024-117498-T_SourceDataForAppendix/EMBOJ-2024-117498-T_SourceDataForAppendixFig. S5/Supplementary Figure 5E/README/HT29_all biological replicates_western.pdf]

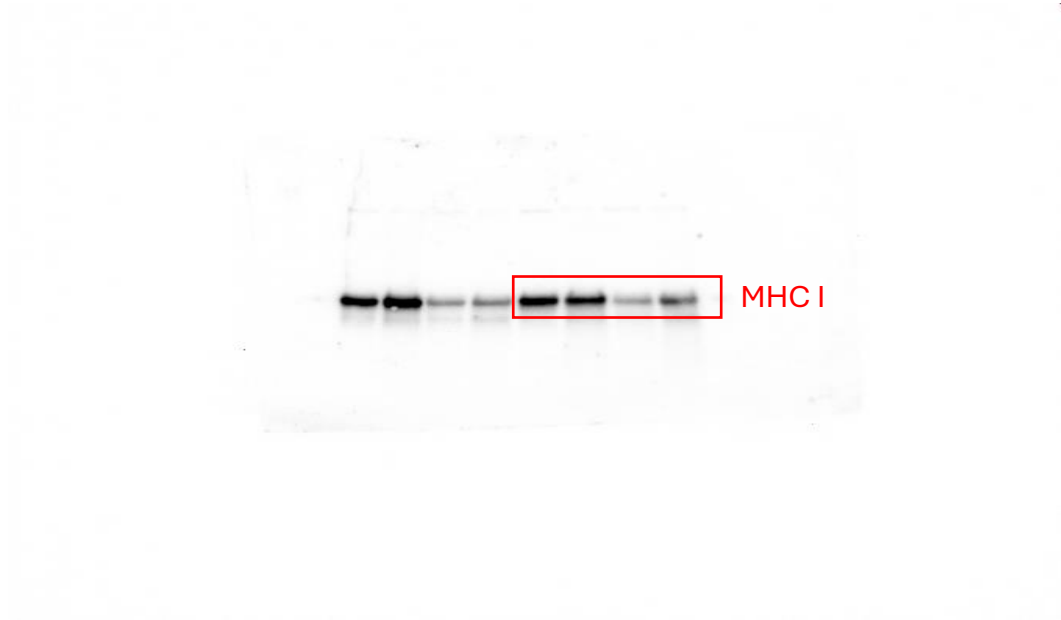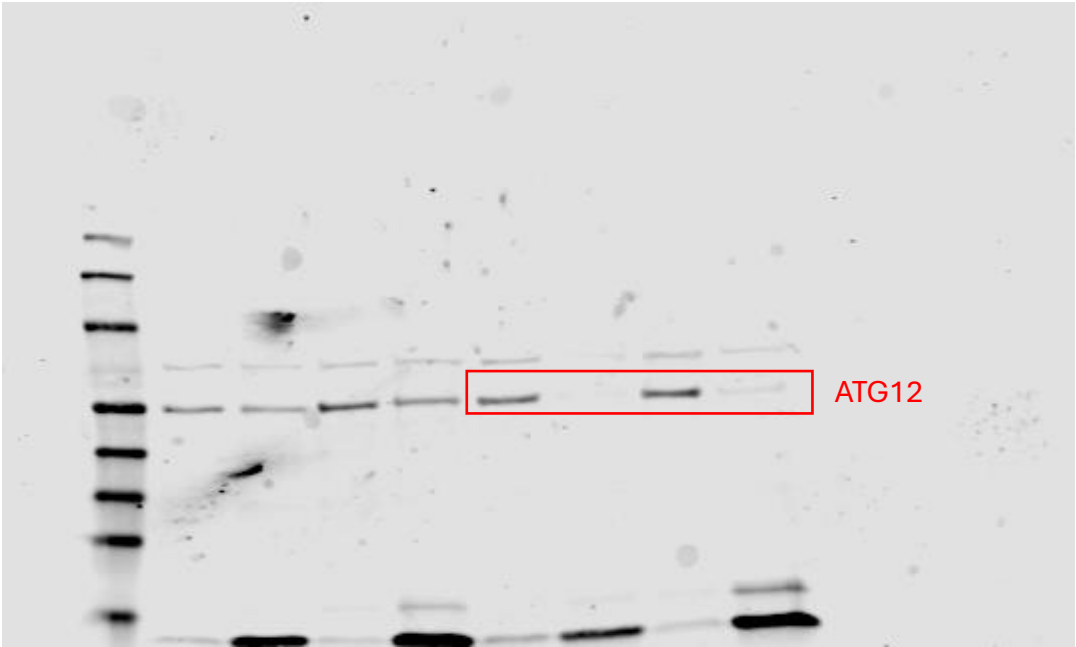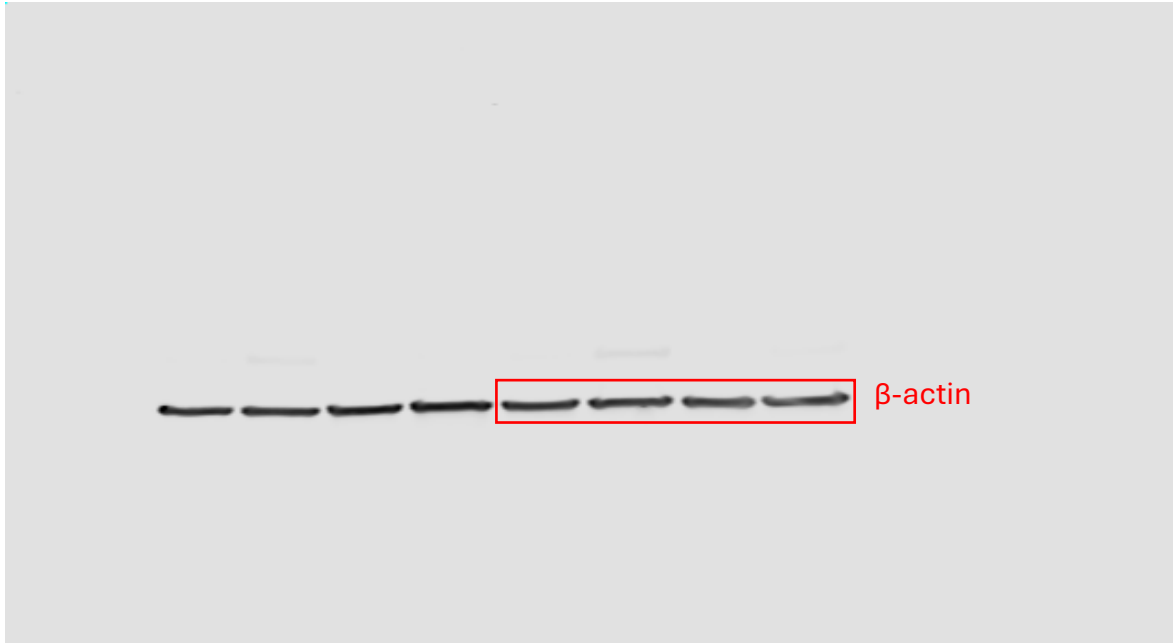

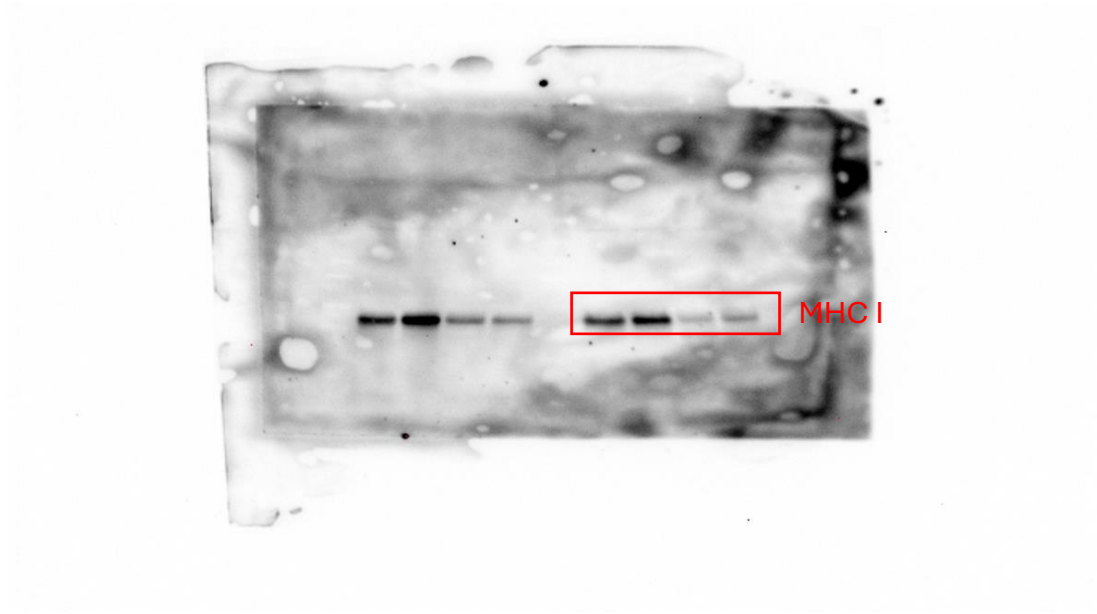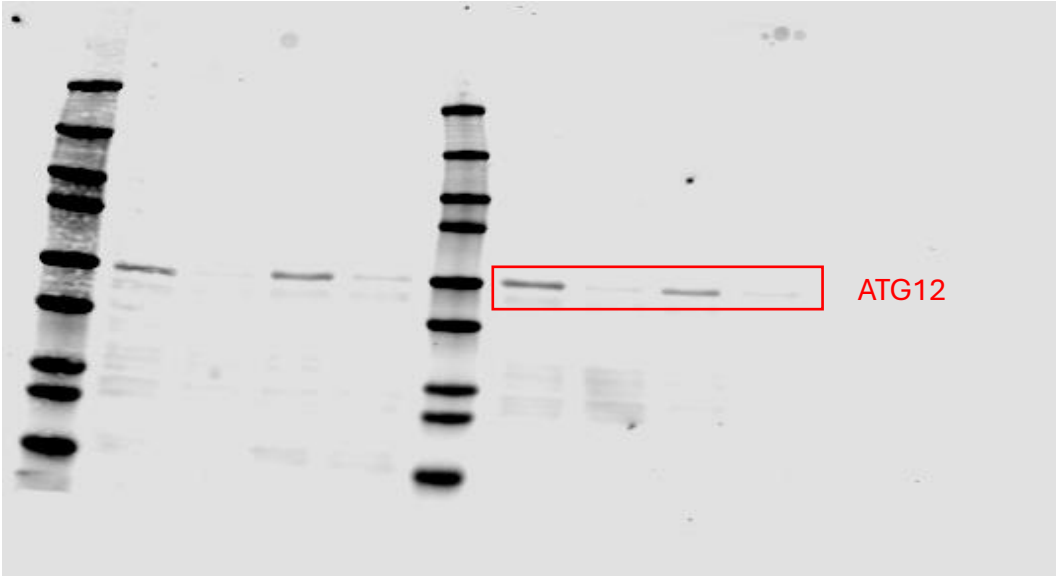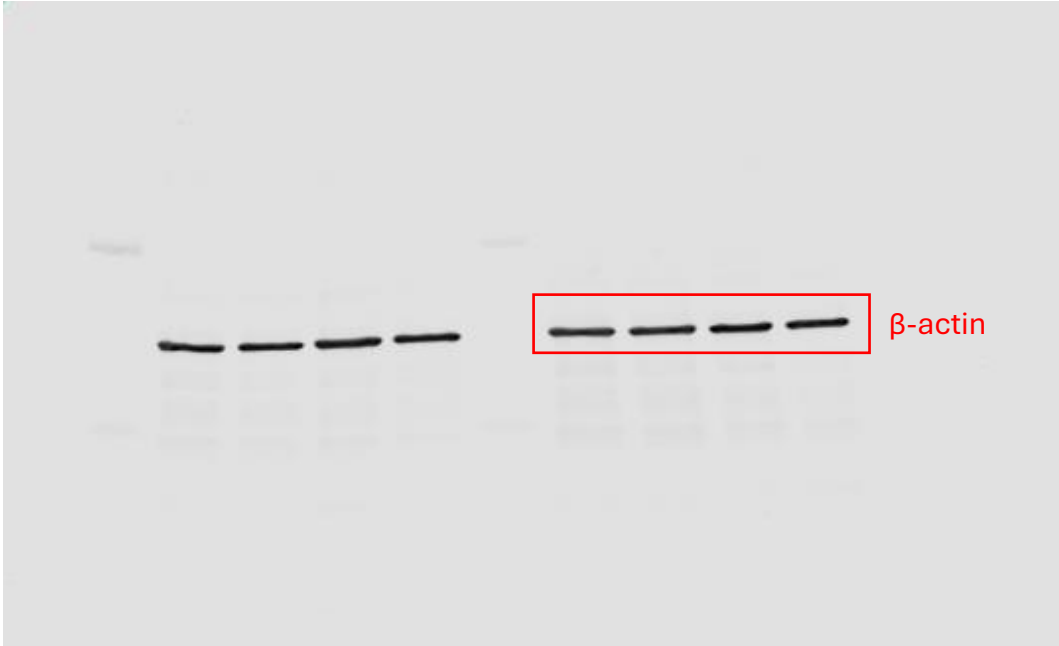

Supplement: Supplementary file 10 — Appendix Figure Source Data [file 44318_2024_319_MOESM10_ESM.zip › EMBOJ-2024-117498-T_SourceDataForAppendix/EMBOJ-2024-117498-T_SourceDataForAppendixFig. S5/Supplementary Figure 5F/README/HT29_all biological replicates_western.pdf]

Bits

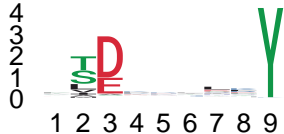

Bits

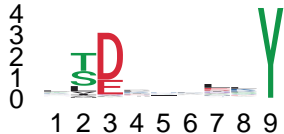

Bits

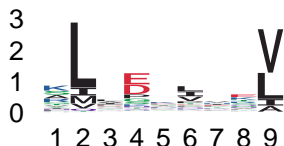

Bits

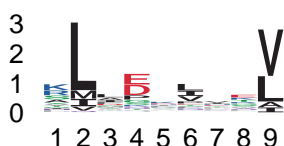

Bits

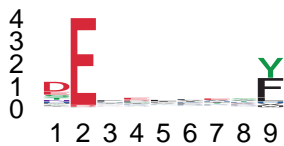

Bits

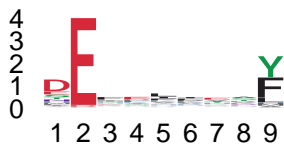

Bits

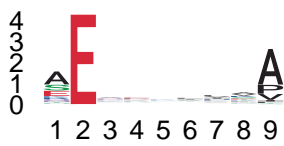

Bits

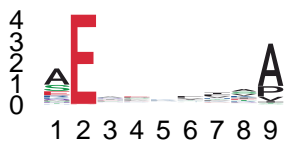

Bits

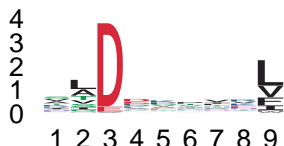

Bits

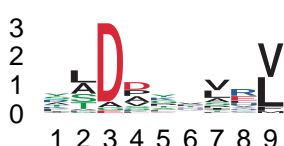

Bits

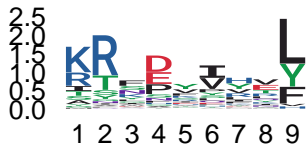

Bits

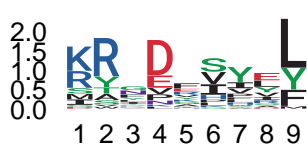

Supplement: Supplementary file 10 — Appendix Figure Source Data [file 44318_2024_319_MOESM10_ESM.zip › EMBOJ-2024-117498-T_SourceDataForAppendix/EMBOJ-2024-117498-T_SourceDataForAppendixFig. S6/Supplementary Figure 6B/seqlogo HCT116 MHC I subtypes .pdf]

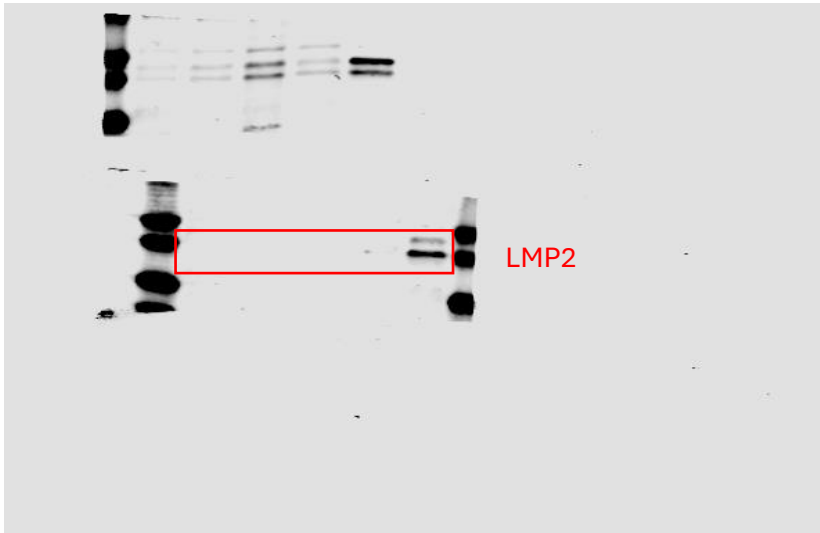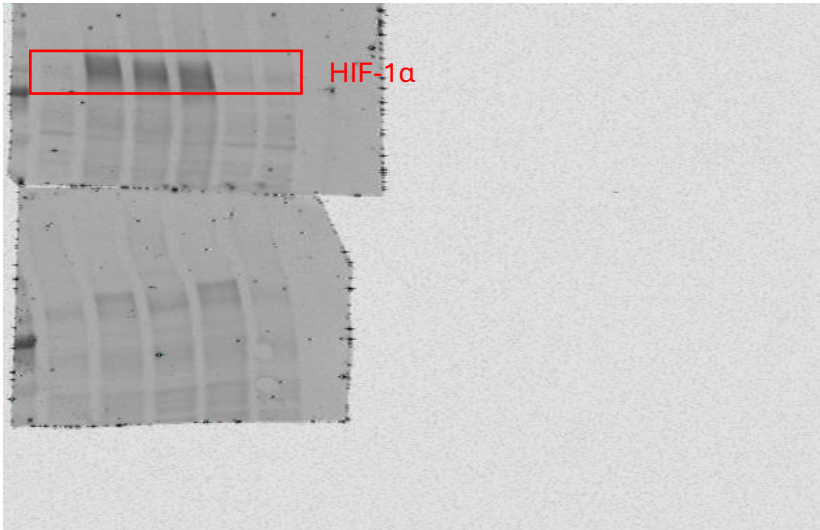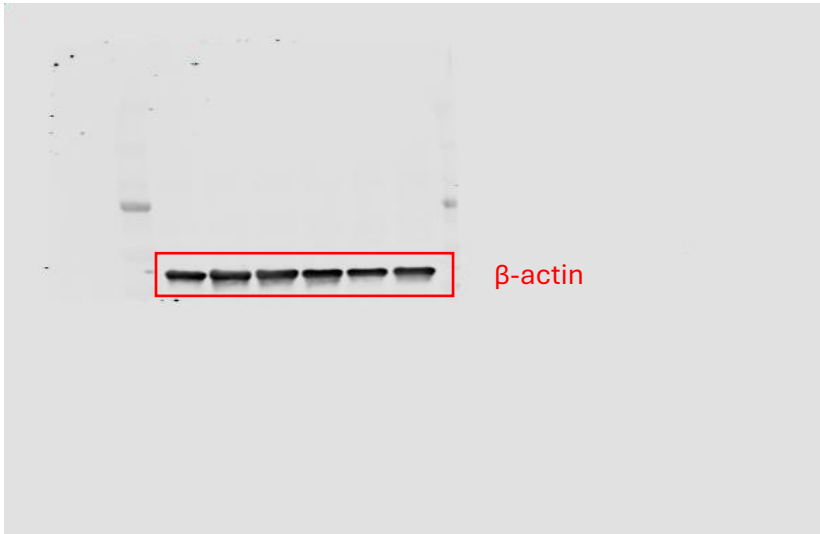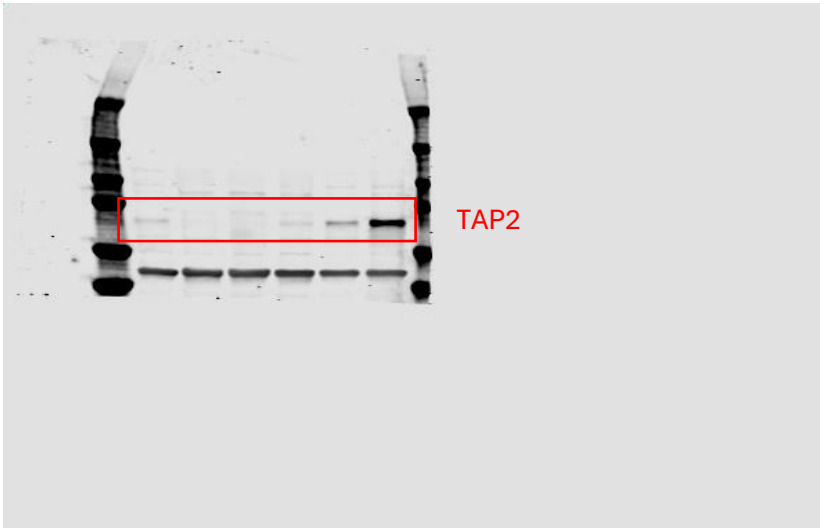

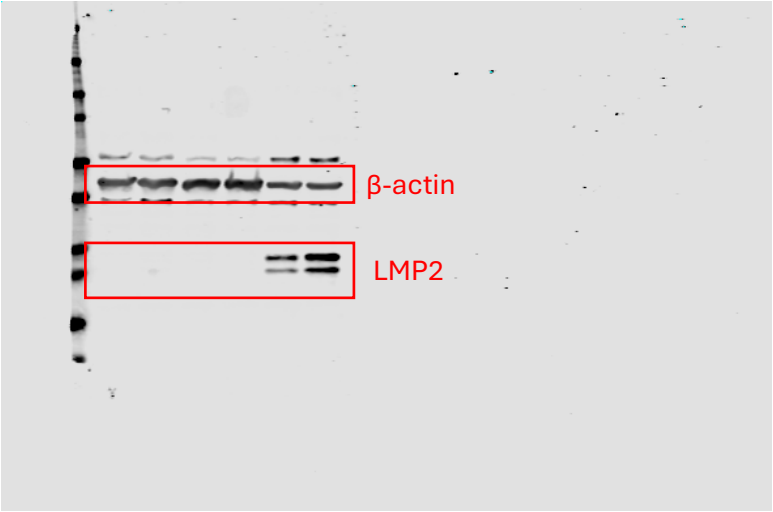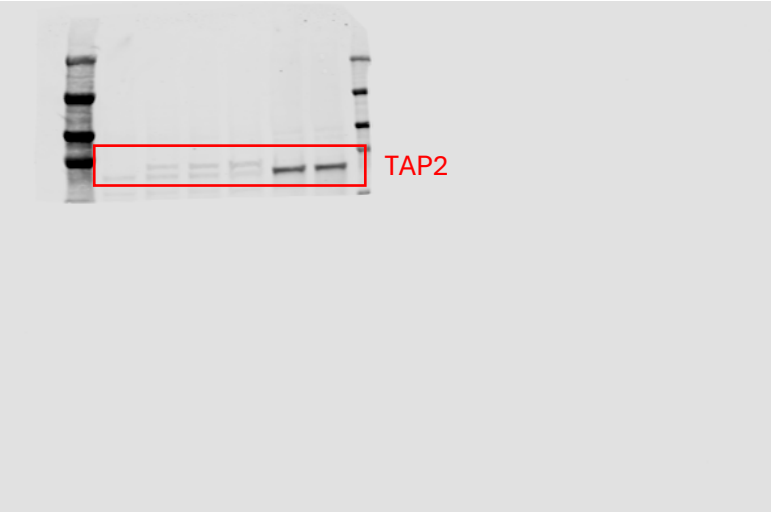

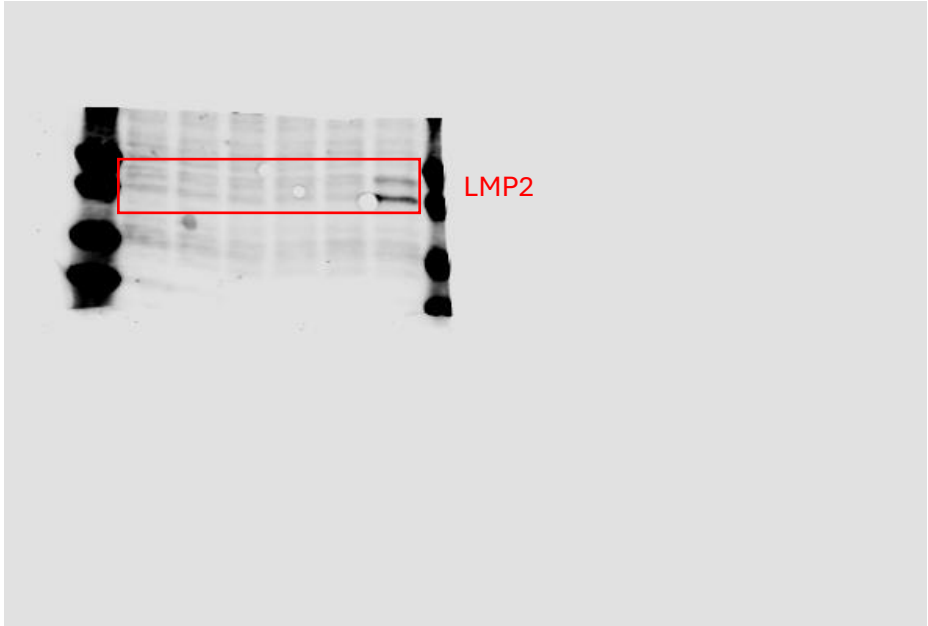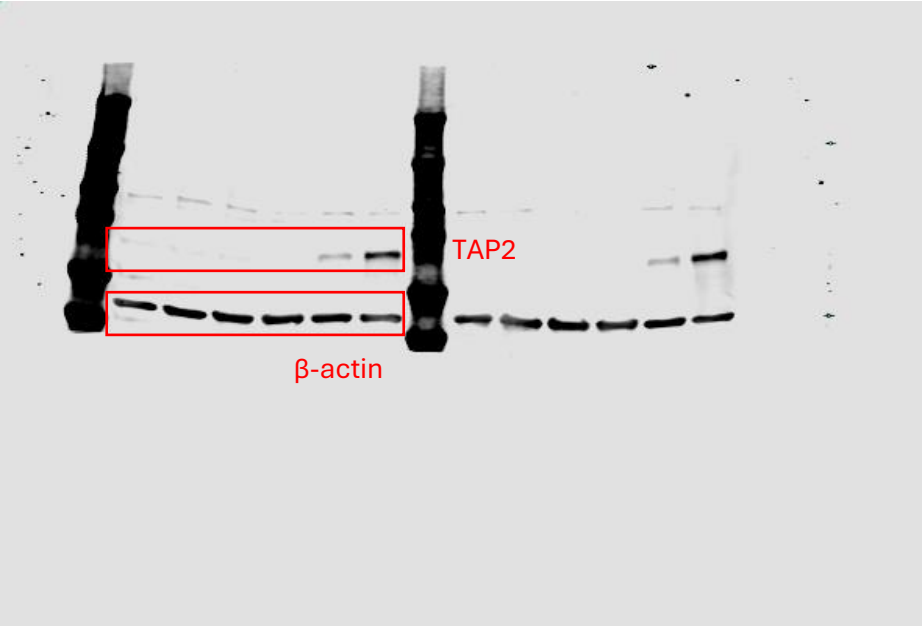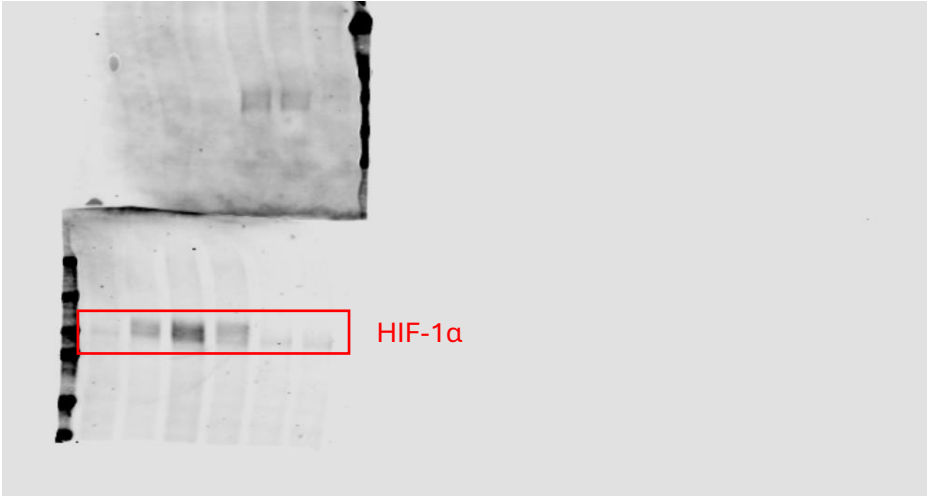

Supplement: Supplementary file 10 — Appendix Figure Source Data [file 44318_2024_319_MOESM10_ESM.zip › EMBOJ-2024-117498-T_SourceDataForAppendix/EMBOJ-2024-117498-T_SourceDataForAppendixFig. S6/Supplementary Figure 6C/README/Calu6_all biological repeats_western.pdf]

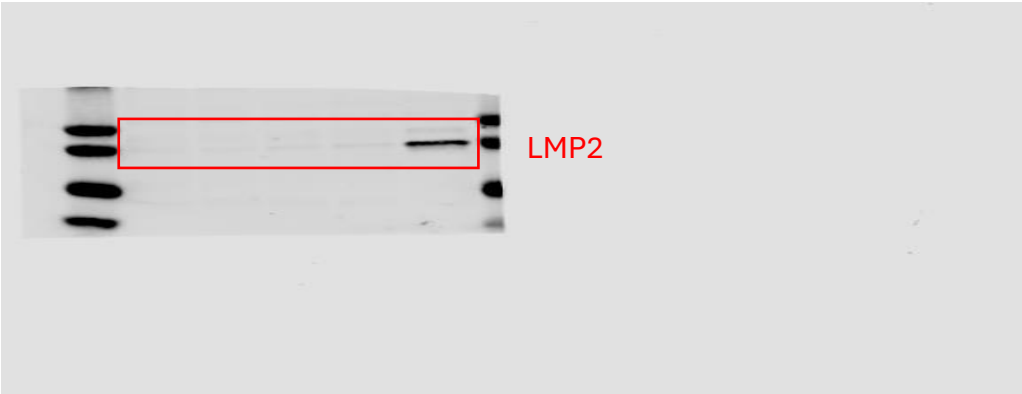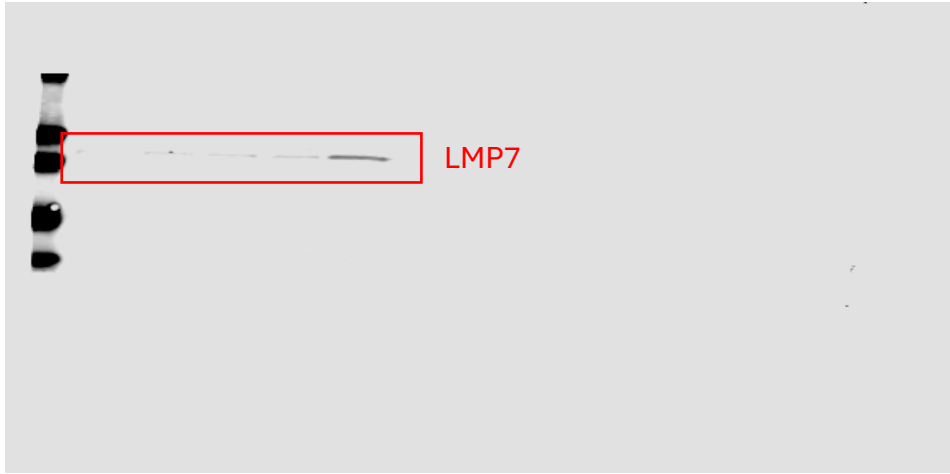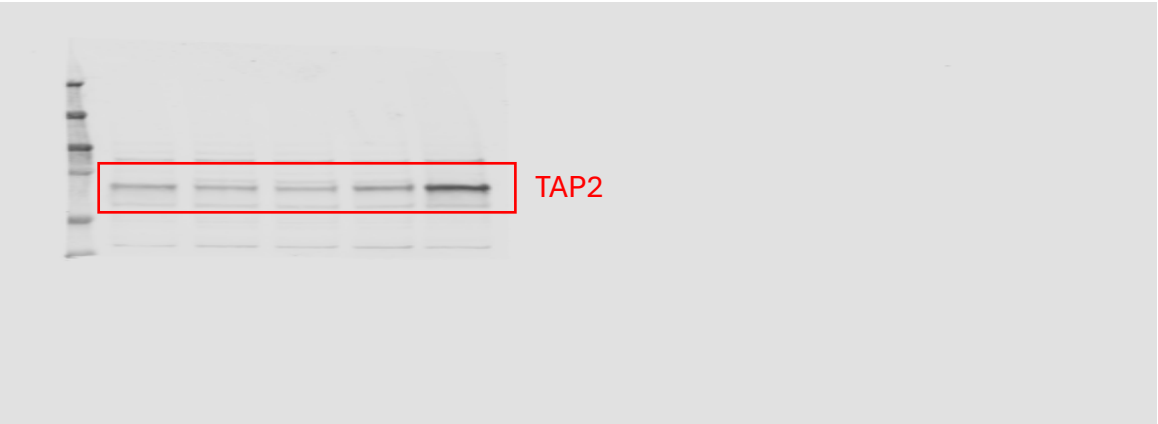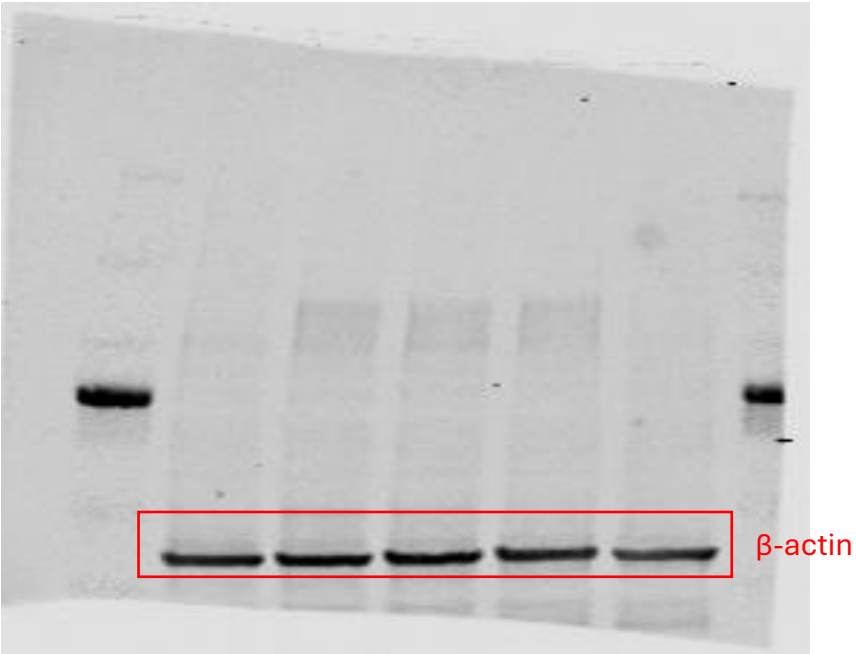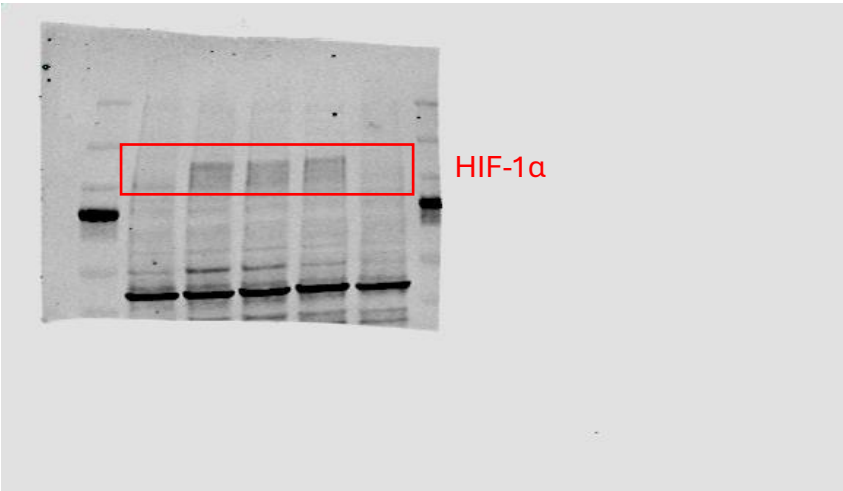

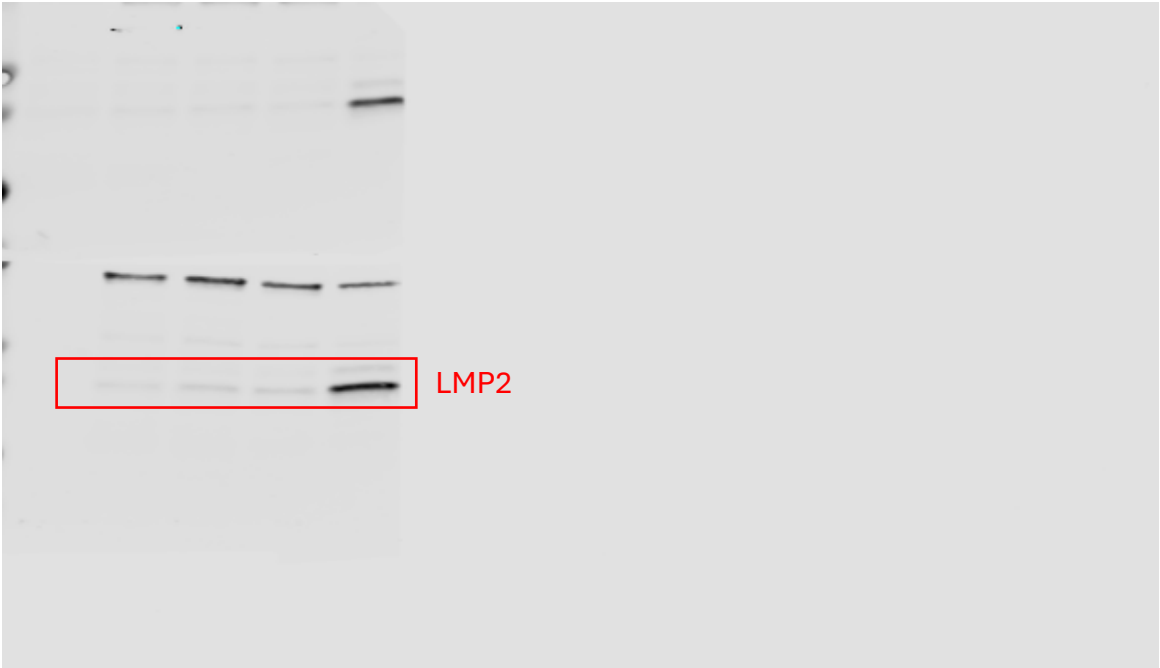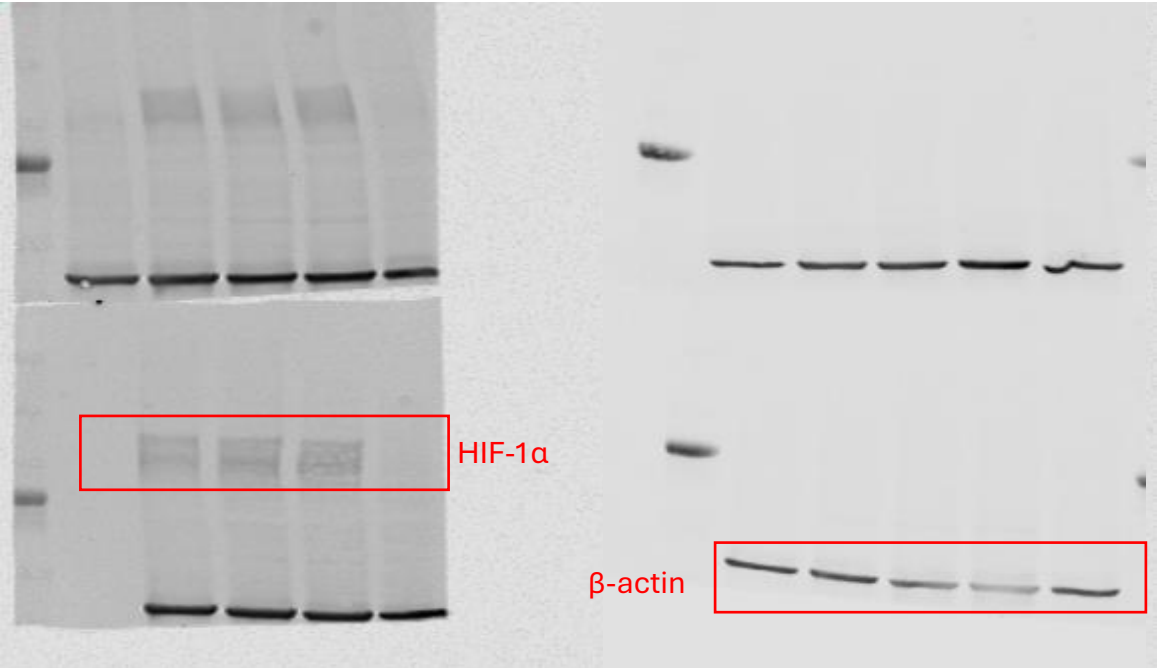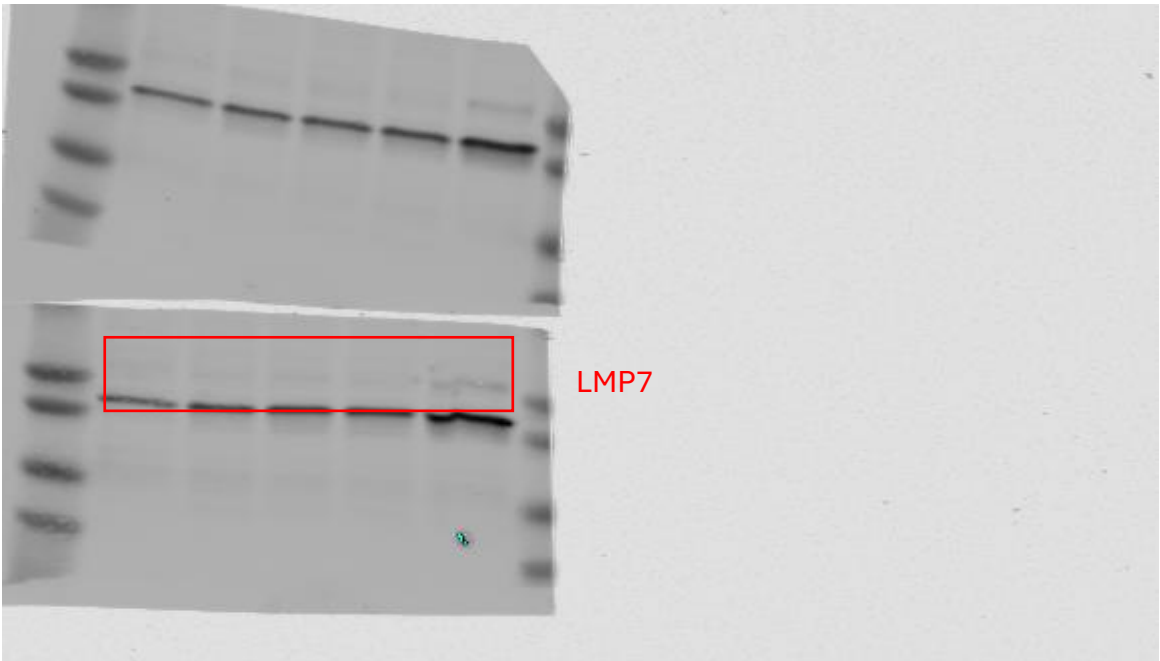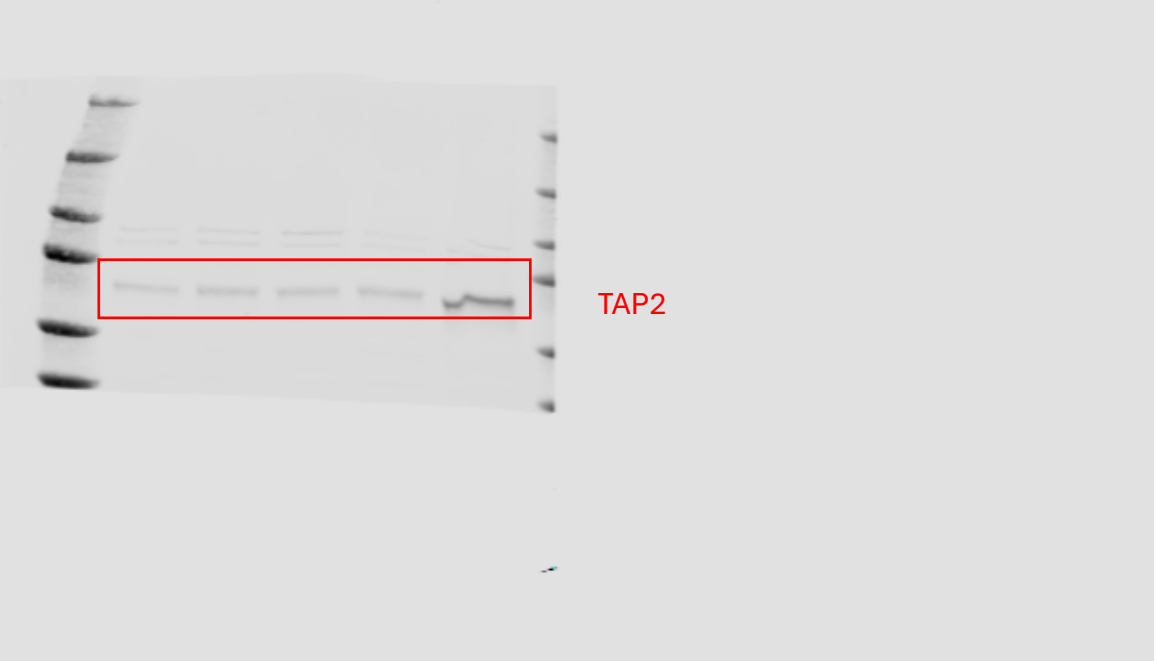

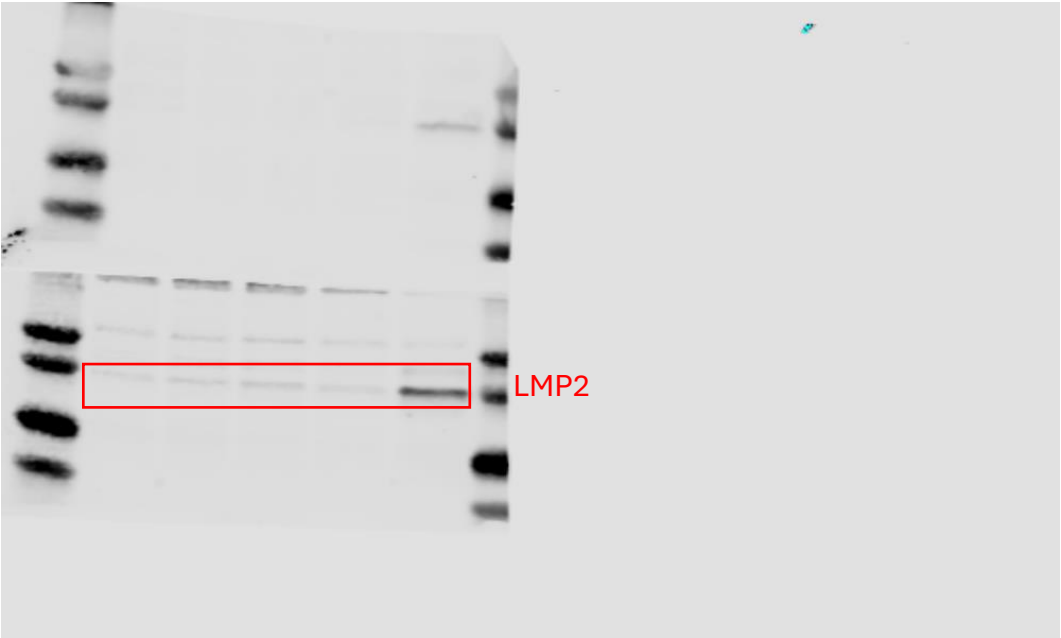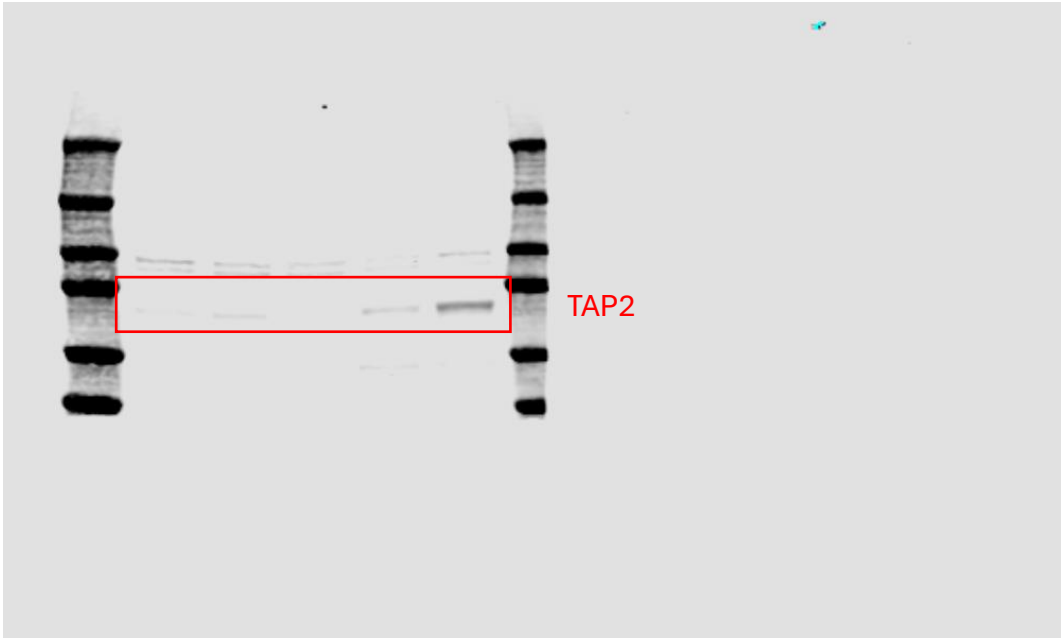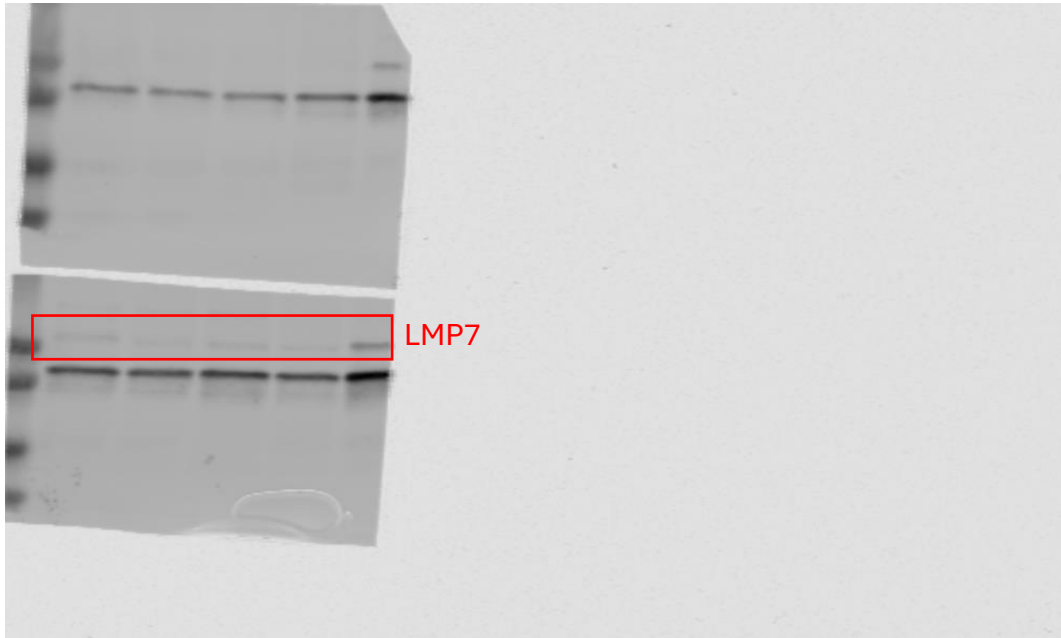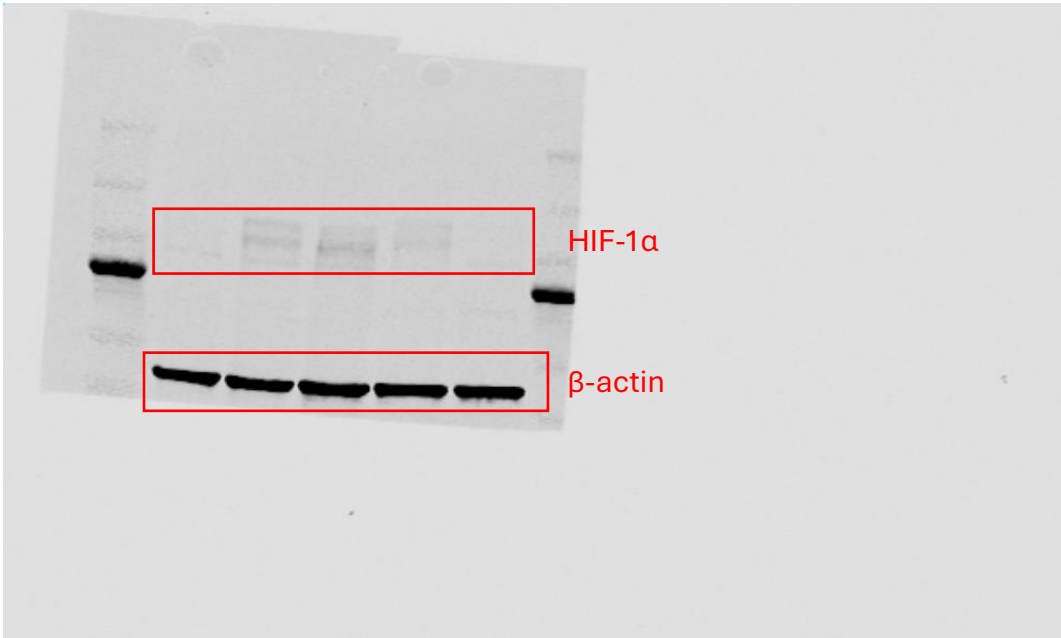

Supplement: Supplementary file 10 — Appendix Figure Source Data [file 44318_2024_319_MOESM10_ESM.zip › EMBOJ-2024-117498-T_SourceDataForAppendix/EMBOJ-2024-117498-T_SourceDataForAppendixFig. S6/Supplementary Figure 6C/README/DLD1_all biological repeats_western.pdf]

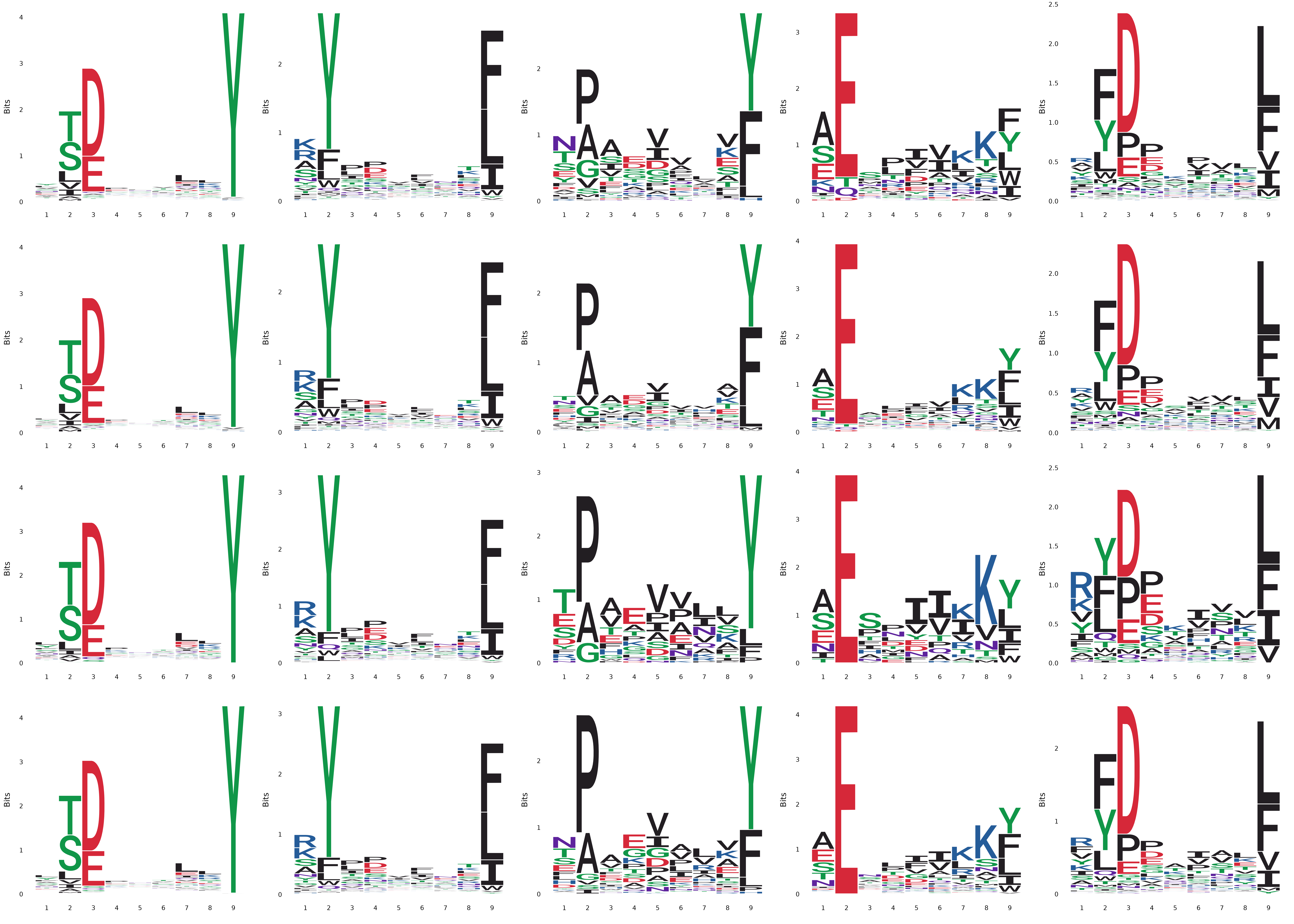

Supplement: Supplementary file 10 — Appendix Figure Source Data [file 44318_2024_319_MOESM10_ESM.zip › EMBOJ-2024-117498-T_SourceDataForAppendix/EMBOJ-2024-117498-T_SourceDataForAppendixFig. S7/seqlogo.png]

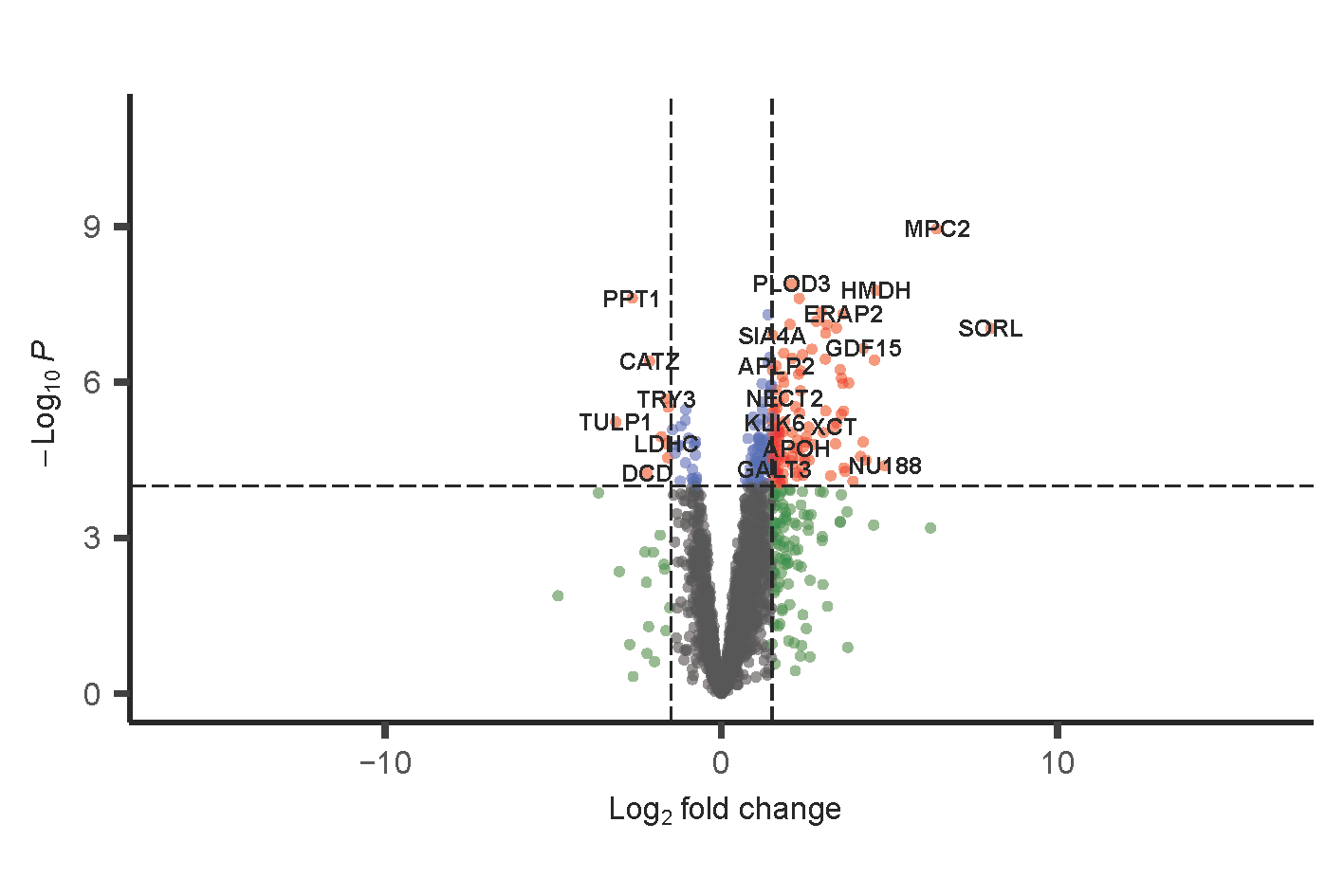

Supplement: Supplementary file 10 — Appendix Figure Source Data [file 44318_2024_319_MOESM10_ESM.zip › EMBOJ-2024-117498-T_SourceDataForAppendix/EMBOJ-2024-117498-T_SourceDataForAppendixFig. S8/Supplementary Figure 8B/Hypo_BAF_vs_Hypo_volcano.png]

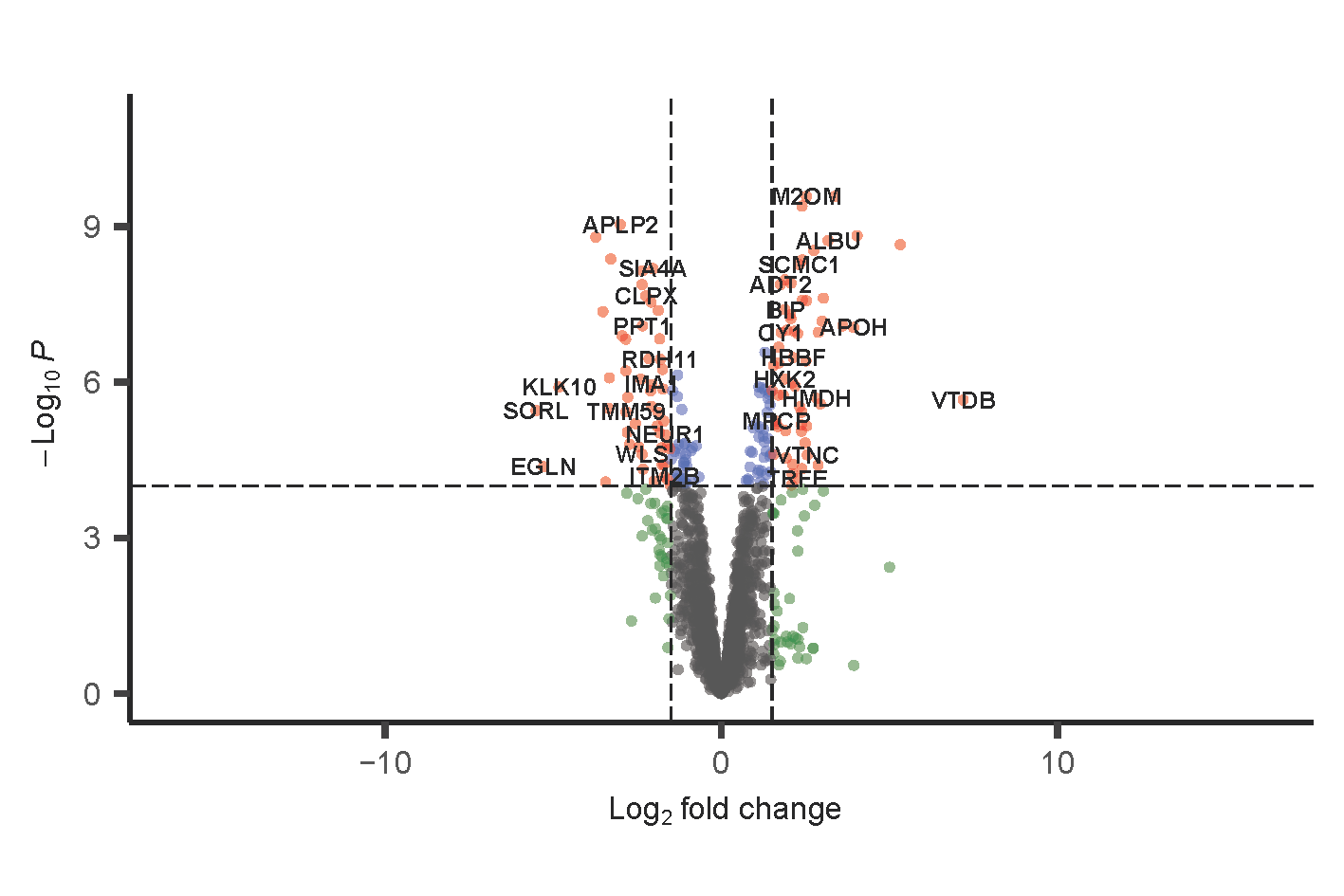

Supplement: Supplementary file 10 — Appendix Figure Source Data [file 44318_2024_319_MOESM10_ESM.zip › EMBOJ-2024-117498-T_SourceDataForAppendix/EMBOJ-2024-117498-T_SourceDataForAppendixFig. S8/Supplementary Figure 8B/Hypo_BAF_vs_Norm_BAF_volcano.png]

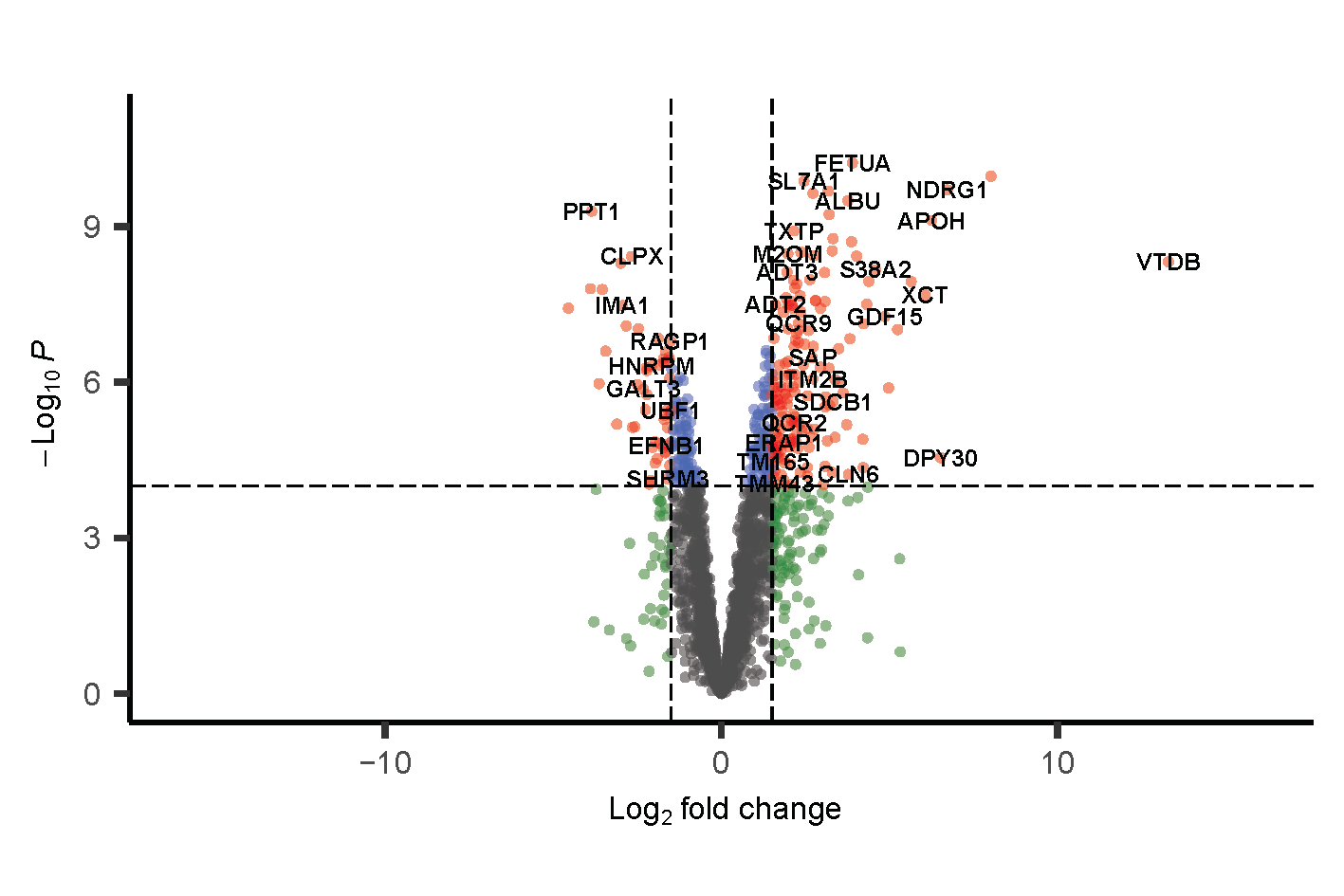

Supplement: Supplementary file 10 — Appendix Figure Source Data [file 44318_2024_319_MOESM10_ESM.zip › EMBOJ-2024-117498-T_SourceDataForAppendix/EMBOJ-2024-117498-T_SourceDataForAppendixFig. S8/Supplementary Figure 8B/Hypo_BAF_vs_Norm_volcano.png]

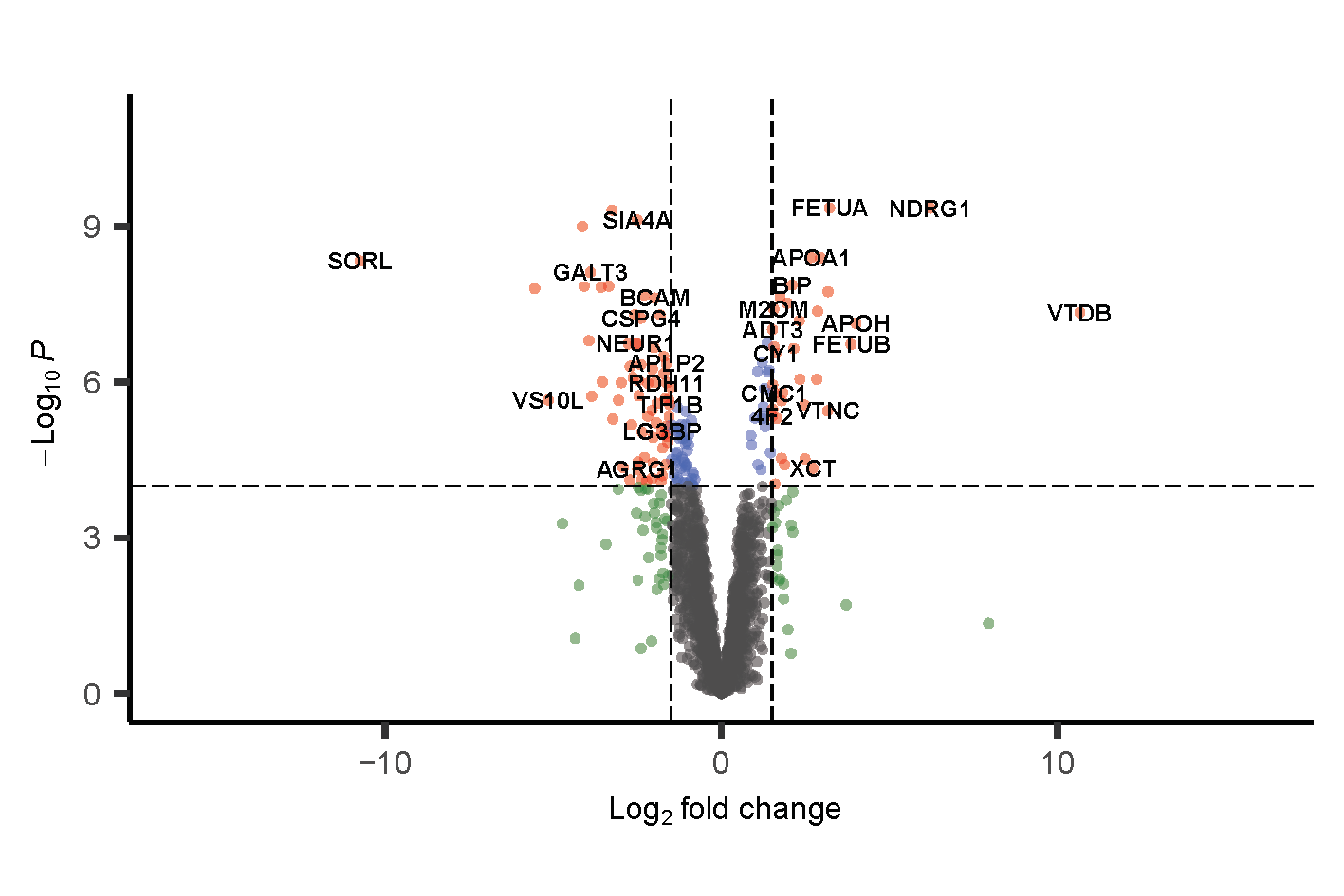

Supplement: Supplementary file 10 — Appendix Figure Source Data [file 44318_2024_319_MOESM10_ESM.zip › EMBOJ-2024-117498-T_SourceDataForAppendix/EMBOJ-2024-117498-T_SourceDataForAppendixFig. S8/Supplementary Figure 8B/Hypo_vs_Norm_volcano.png]

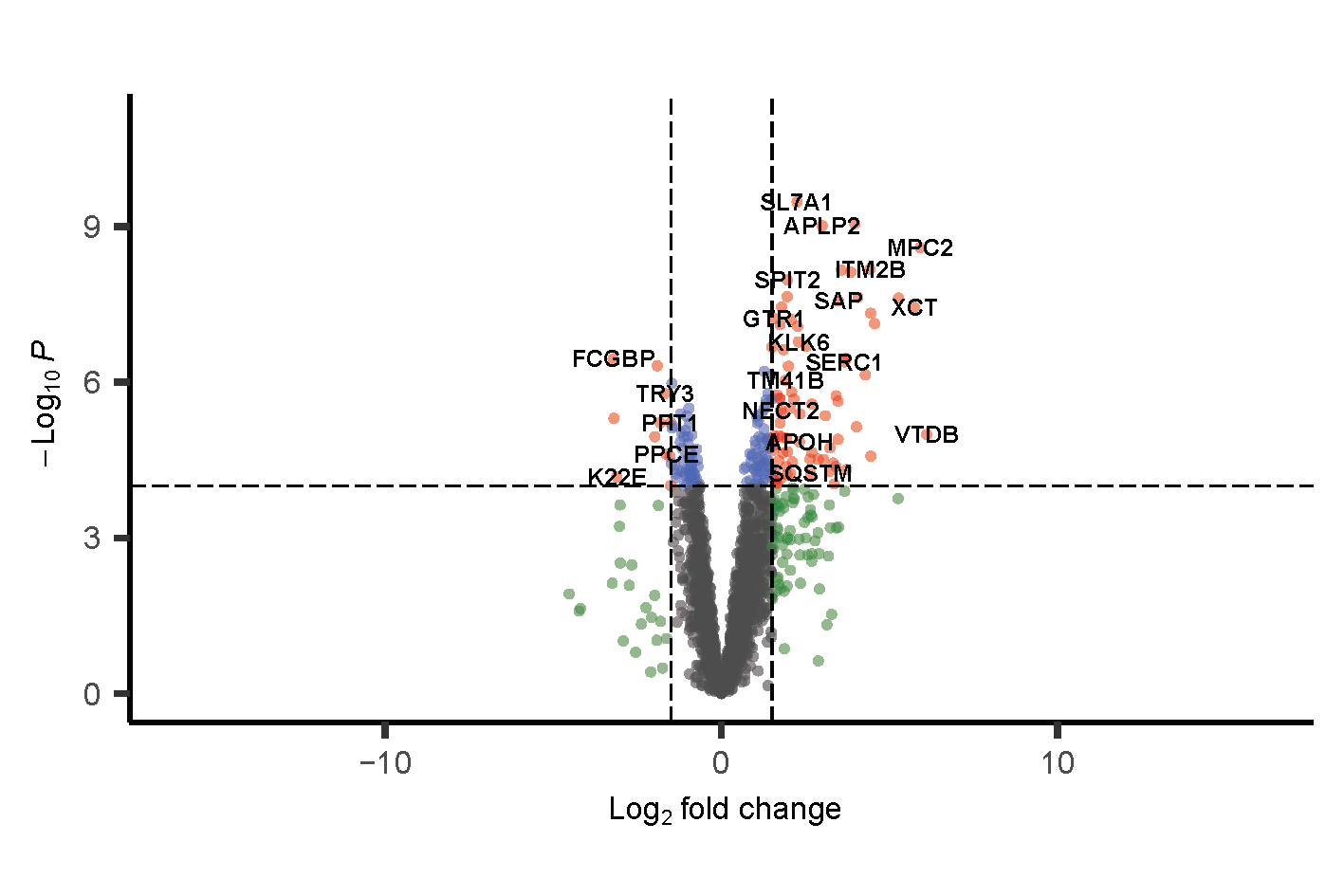

Supplement: Supplementary file 10 — Appendix Figure Source Data [file 44318_2024_319_MOESM10_ESM.zip › EMBOJ-2024-117498-T_SourceDataForAppendix/EMBOJ-2024-117498-T_SourceDataForAppendixFig. S8/Supplementary Figure 8B/Norm_BAF_vs_Hypo_volcano.png]

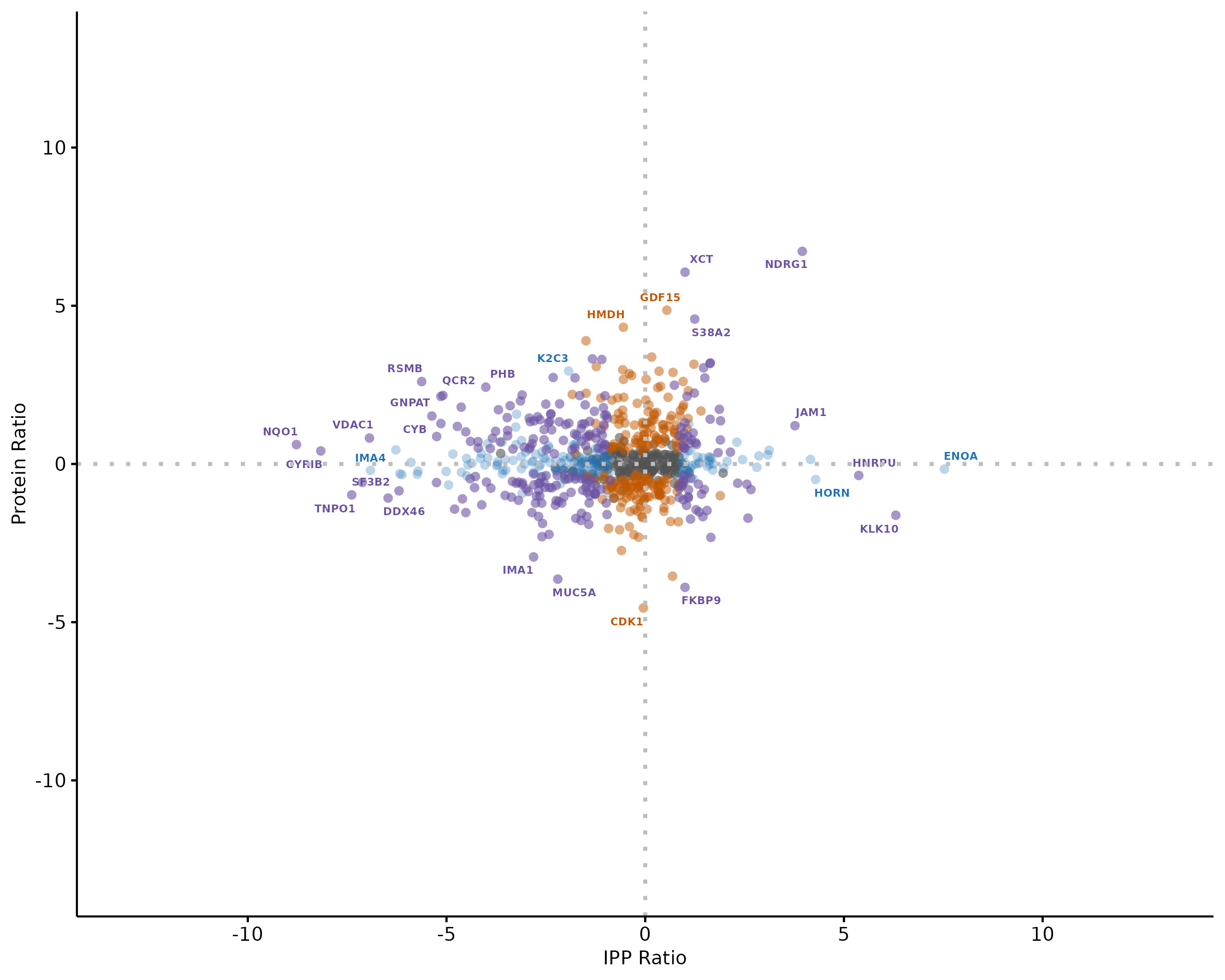

Supplement: Supplementary file 10 — Appendix Figure Source Data [file 44318_2024_319_MOESM10_ESM.zip › EMBOJ-2024-117498-T_SourceDataForAppendix/EMBOJ-2024-117498-T_SourceDataForAppendixFig. S9/correlationplot_hypobaf_v_norm.png]

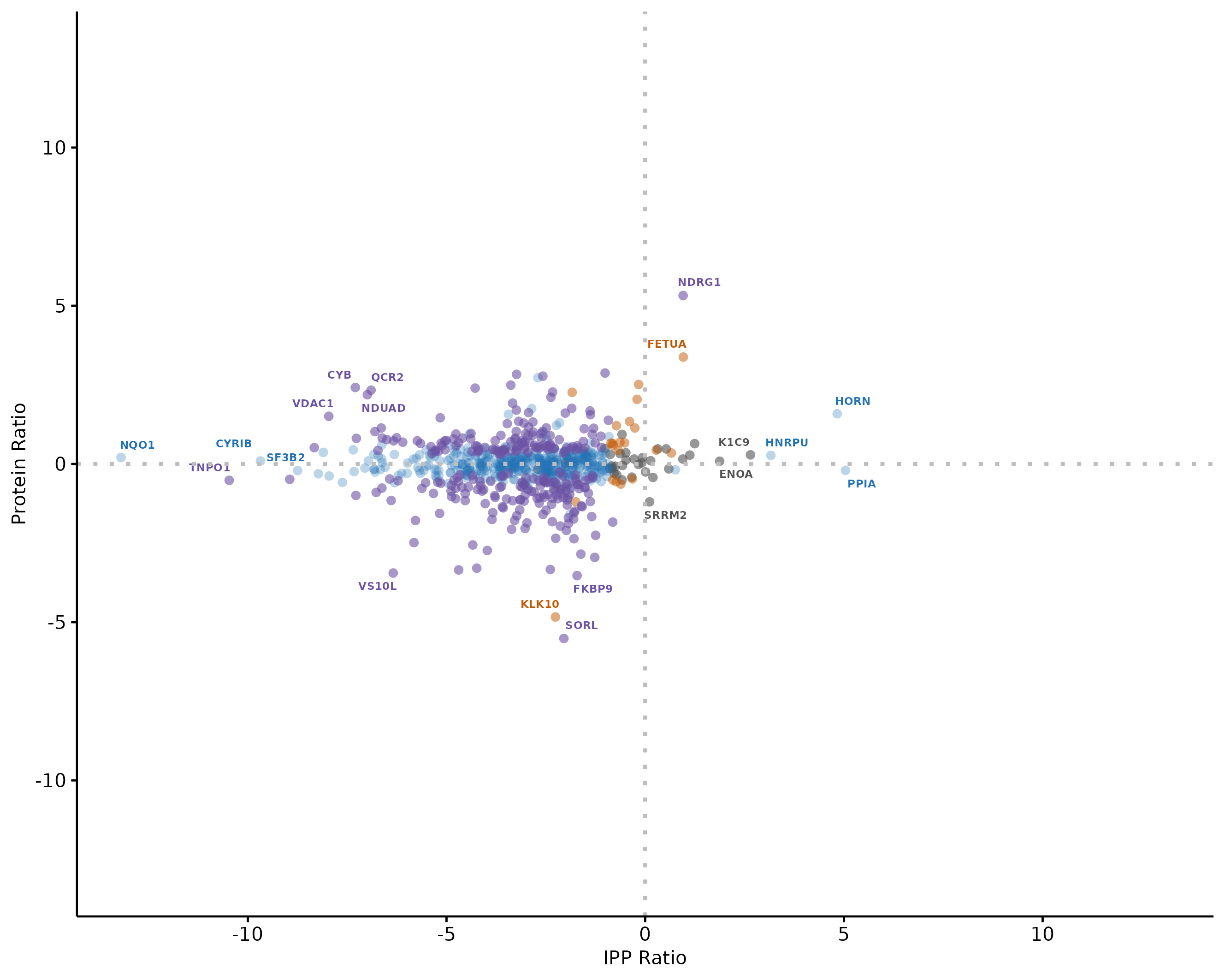

Supplement: Supplementary file 10 — Appendix Figure Source Data [file 44318_2024_319_MOESM10_ESM.zip › EMBOJ-2024-117498-T_SourceDataForAppendix/EMBOJ-2024-117498-T_SourceDataForAppendixFig. S9/correlationplot_hypobaf_v_normbaf.png]

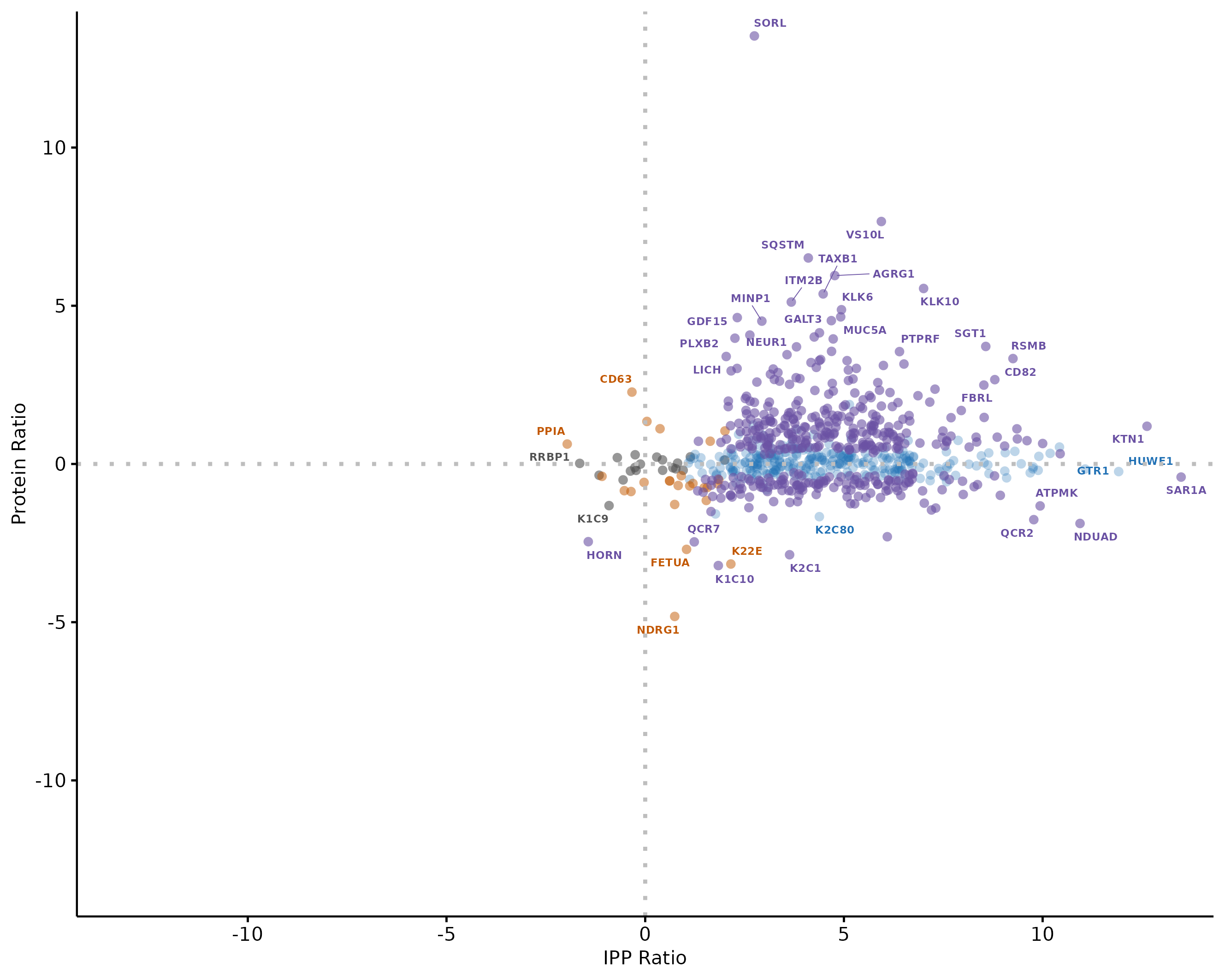

Supplement: Supplementary file 10 — Appendix Figure Source Data [file 44318_2024_319_MOESM10_ESM.zip › EMBOJ-2024-117498-T_SourceDataForAppendix/EMBOJ-2024-117498-T_SourceDataForAppendixFig. S9/correlationplot_normbaf_v_hypo.png]

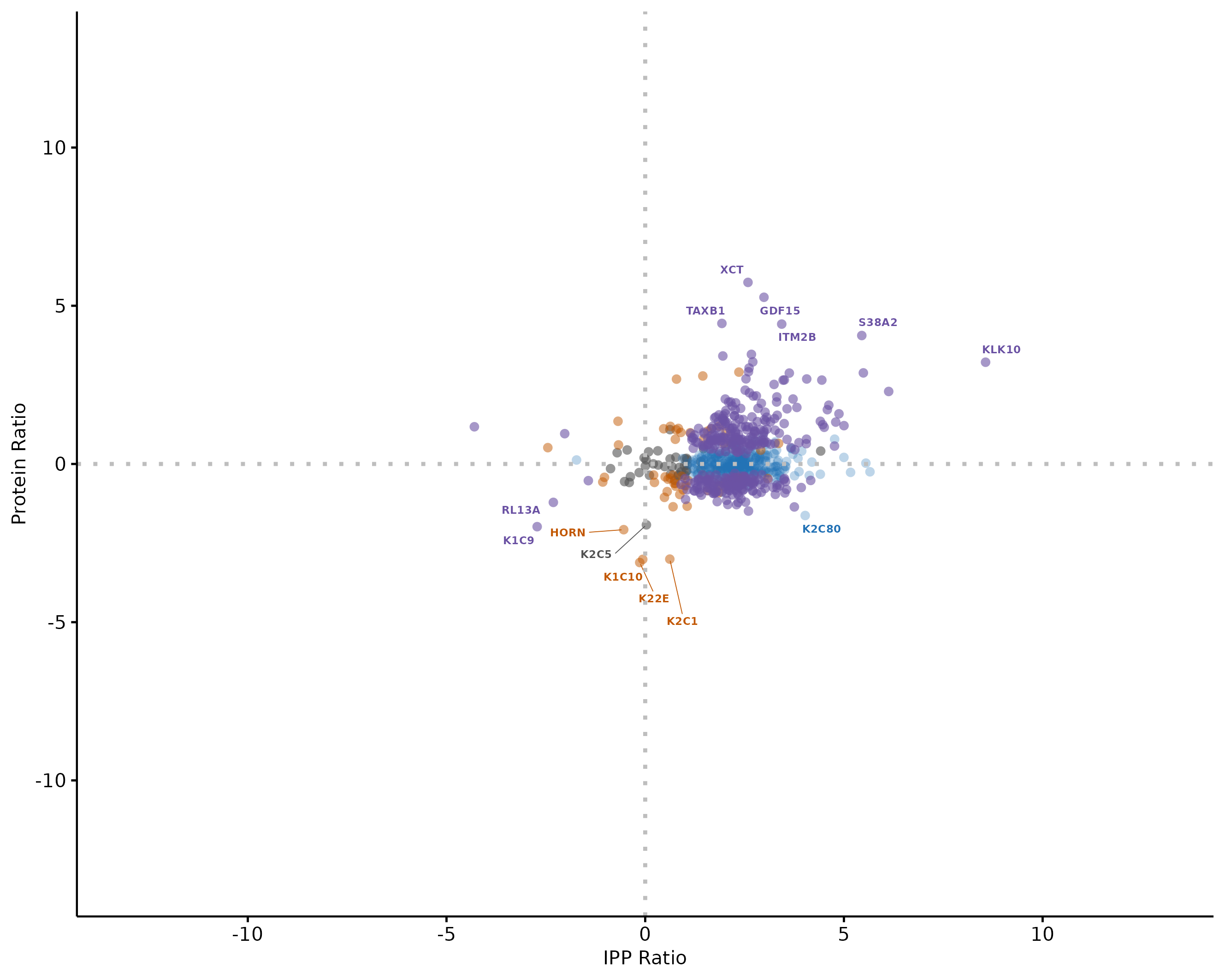

Supplement: Supplementary file 10 — Appendix Figure Source Data [file 44318_2024_319_MOESM10_ESM.zip › EMBOJ-2024-117498-T_SourceDataForAppendix/EMBOJ-2024-117498-T_SourceDataForAppendixFig. S9/correlationplot_normbaf_v_norm.png]
