# Supplementary material for: Hypoxia promotes tumor immune evasion by suppressing MHC-I expression and antigen presentation
Source: EMBO J. 2025 Jan 3;44(3):903–22. doi: 10.1038/s44318-024-00319-7 (PMC11790895; doi:10.1038/s44318-024-00319-7)
Supplement: Supplementary file 10 — Appendix Figure Source Data [file 44318_2024_319_MOESM10_ESM.zip › EMBOJ-2024-117498-T_SourceDataForAppendix/EMBOJ-2024-117498-T_SourceDataForAppendixFig. S5/Supplementary Figure 5E/README/HT29_all biological replicates_western.pptx]

## Slide 1
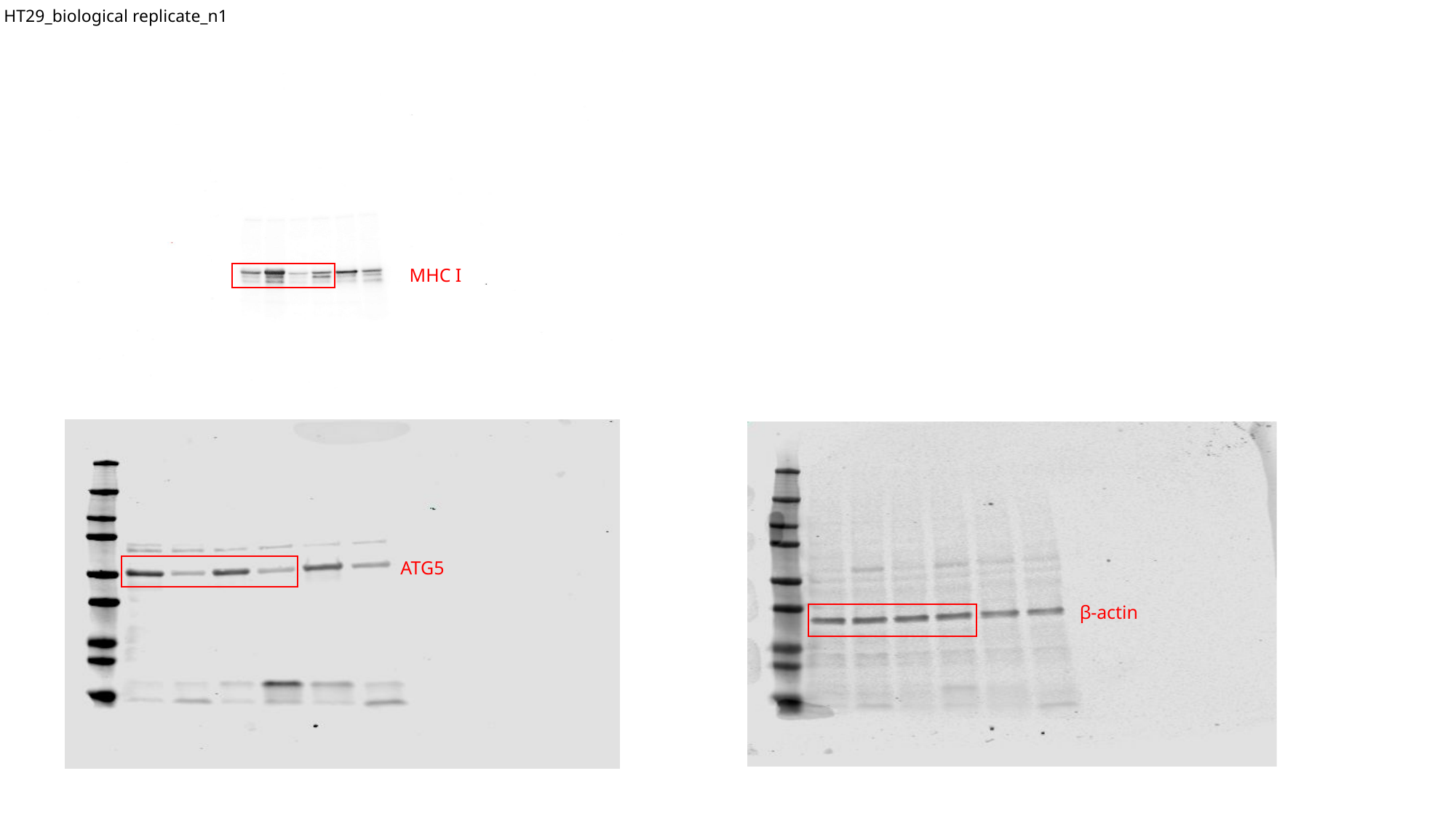

HT29_biological replicate_n1
MHC I
ATG5
β-actin

## Slide 2
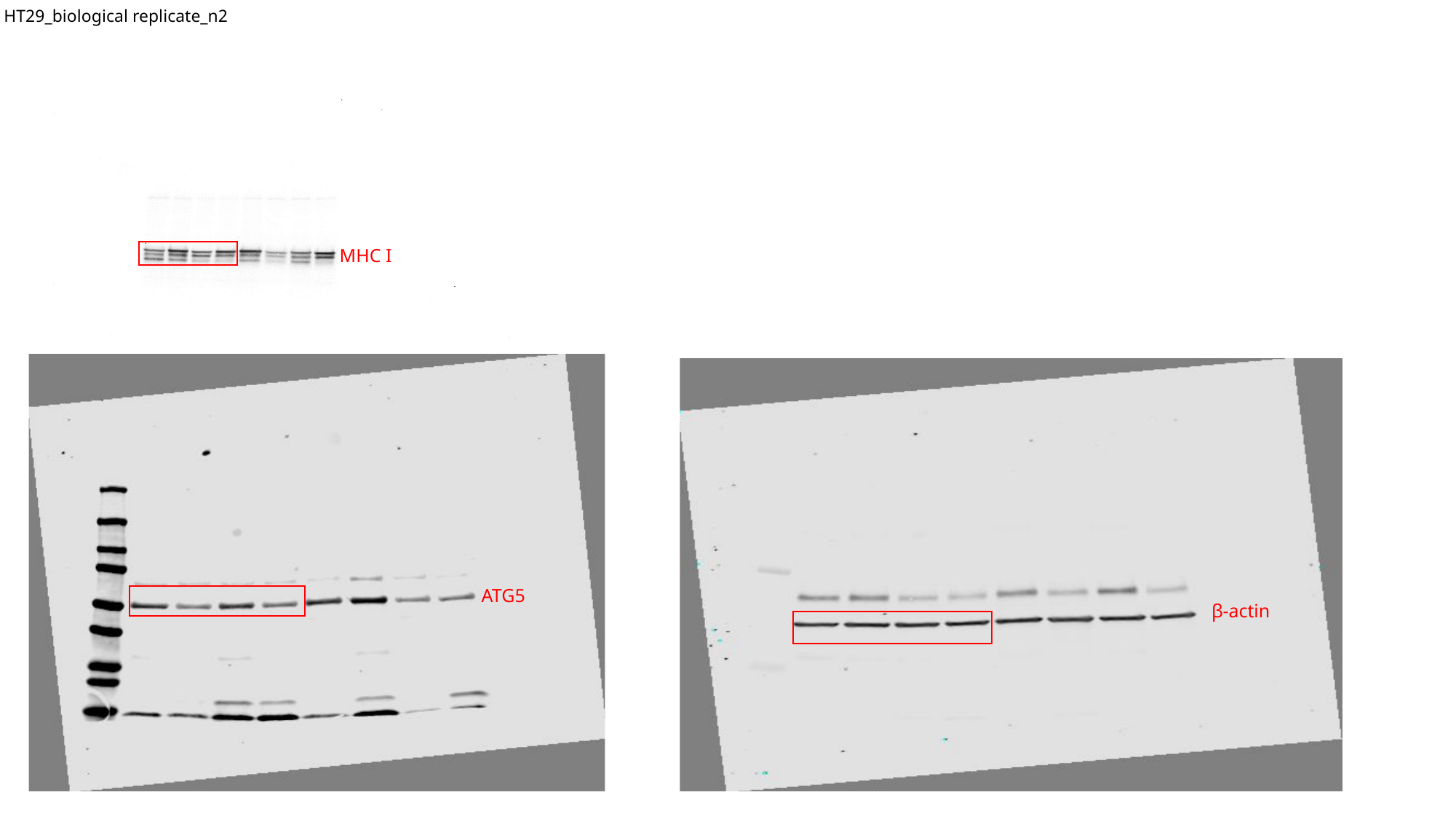

HT29_biological replicate_n2
MHC I
ATG5
β-actin

## Slide 3
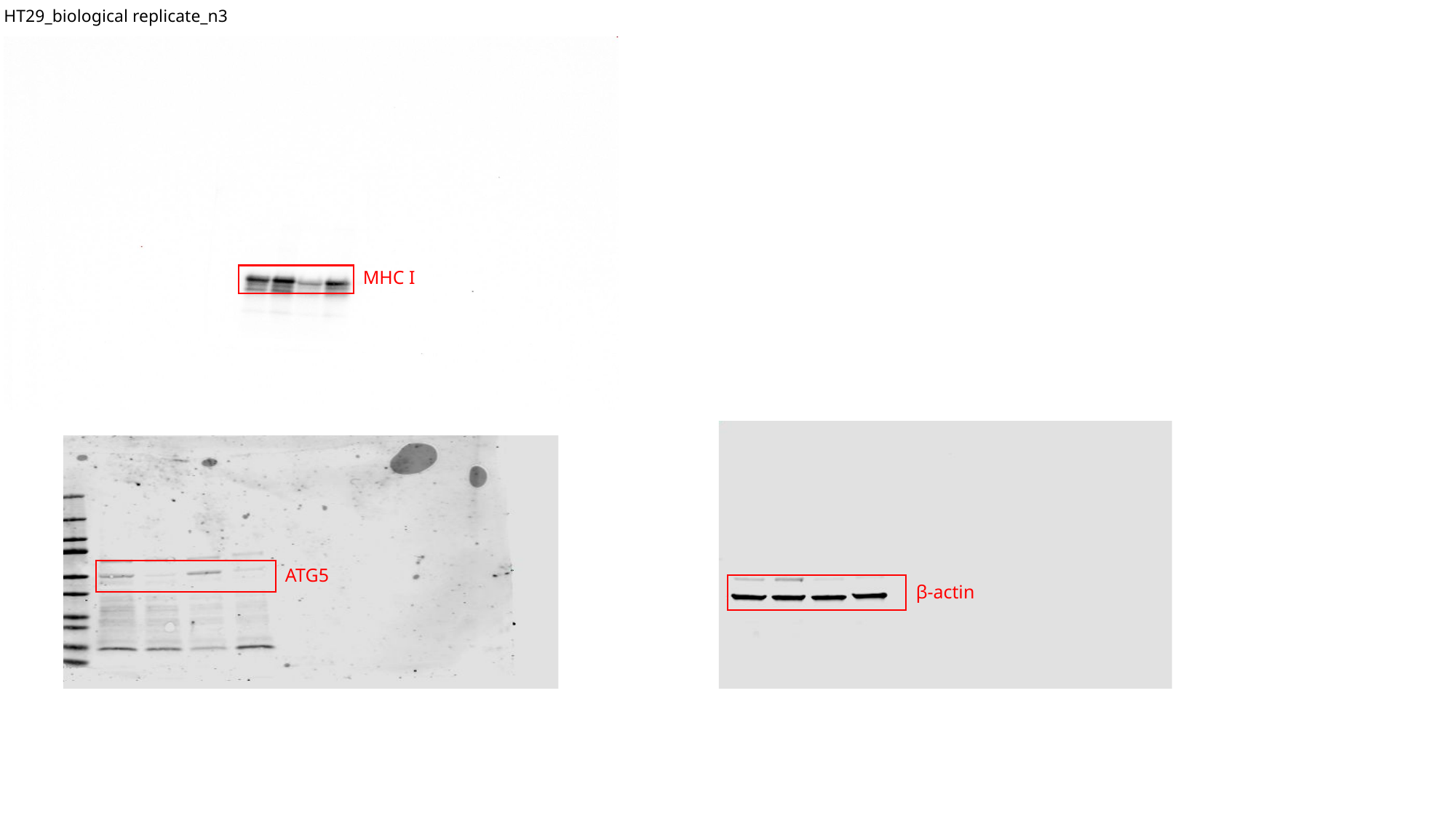

HT29_biological replicate_n3
MHC I
ATG5
β-actin
