# Supplementary material for: Hypoxia promotes tumor immune evasion by suppressing MHC-I expression and antigen presentation
Source: EMBO J. 2025 Jan 3;44(3):903–22. doi: 10.1038/s44318-024-00319-7 (PMC11790895; doi:10.1038/s44318-024-00319-7)
Supplement: Supplementary file 10 — Appendix Figure Source Data [file 44318_2024_319_MOESM10_ESM.zip › EMBOJ-2024-117498-T_SourceDataForAppendix/EMBOJ-2024-117498-T_SourceDataForAppendixFig. S6/Supplementary Figure 6C/README/Calu6_all biological repeats_western.pptx]

## Slide 1
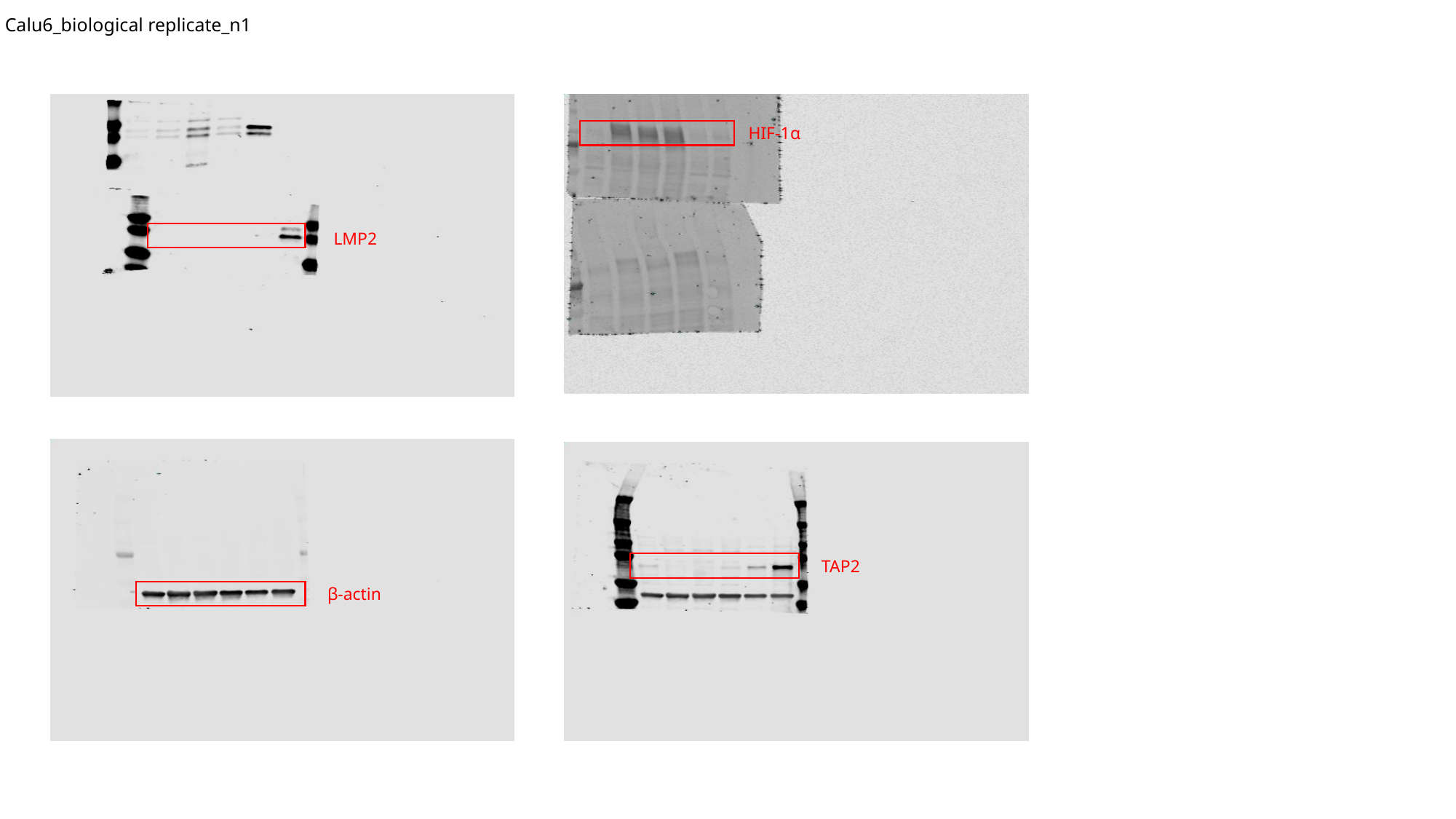

Calu6_biological replicate_n1
HIF-1α
LMP2
TAP2
β-actin

## Slide 2
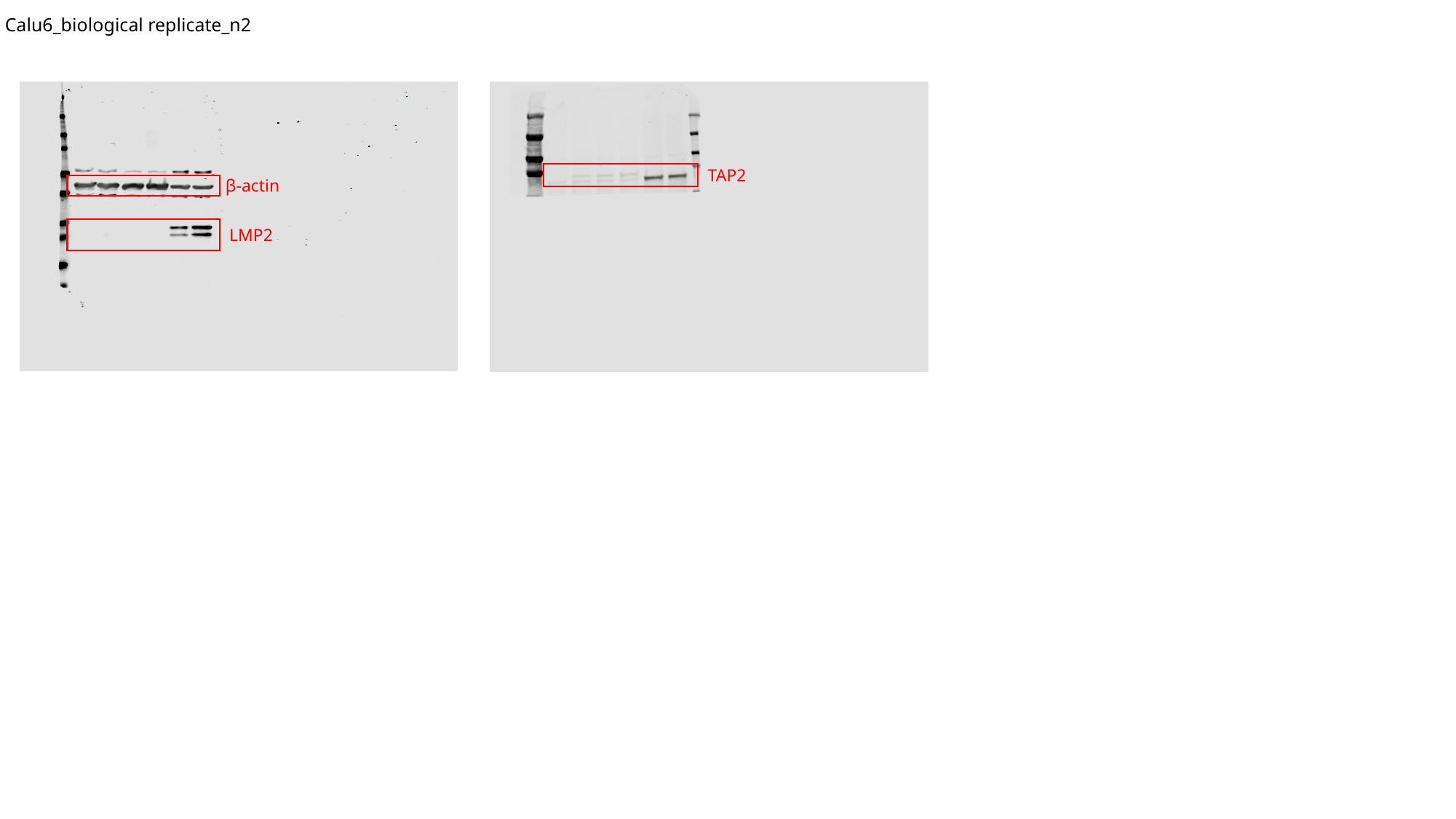

Calu6_biological replicate_n2
TAP2
β-actin
LMP2

## Slide 3
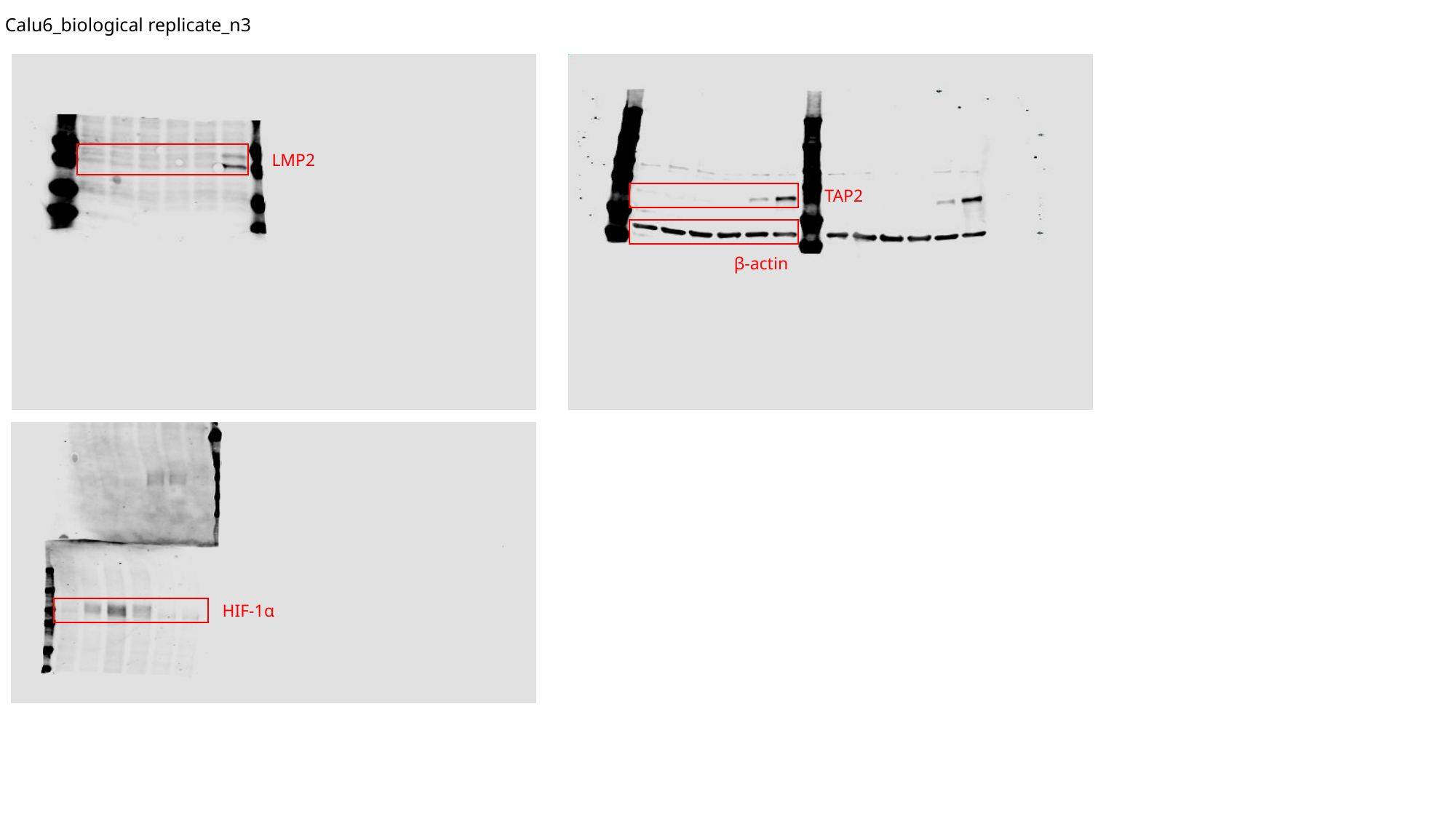

Calu6_biological replicate_n3
LMP2
TAP2
β-actin
HIF-1α
