# Supplementary material for: Hypoxia promotes tumor immune evasion by suppressing MHC-I expression and antigen presentation
Source: EMBO J. 2025 Jan 3;44(3):903–22. doi: 10.1038/s44318-024-00319-7 (PMC11790895; doi:10.1038/s44318-024-00319-7)
Supplement: Supplementary file 10 — Appendix Figure Source Data [file 44318_2024_319_MOESM10_ESM.zip › EMBOJ-2024-117498-T_SourceDataForAppendix/EMBOJ-2024-117498-T_SourceDataForAppendixFig. S6/Supplementary Figure 6C/README/DLD1_all biological repeats_western.pptx]

## Slide 1
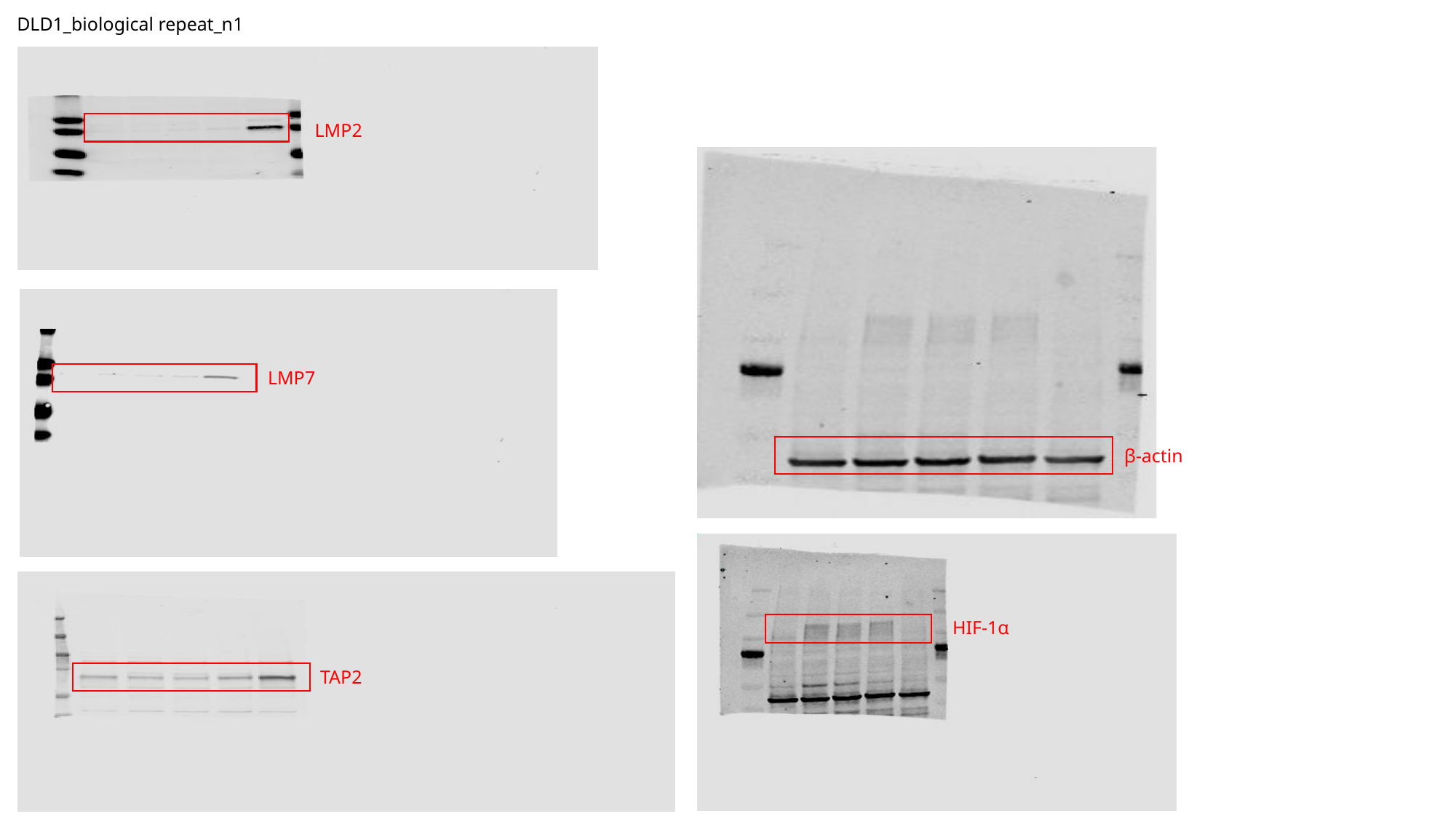

DLD1_biological repeat_n1
LMP2
LMP7
β-actin
HIF-1α
TAP2

## Slide 2
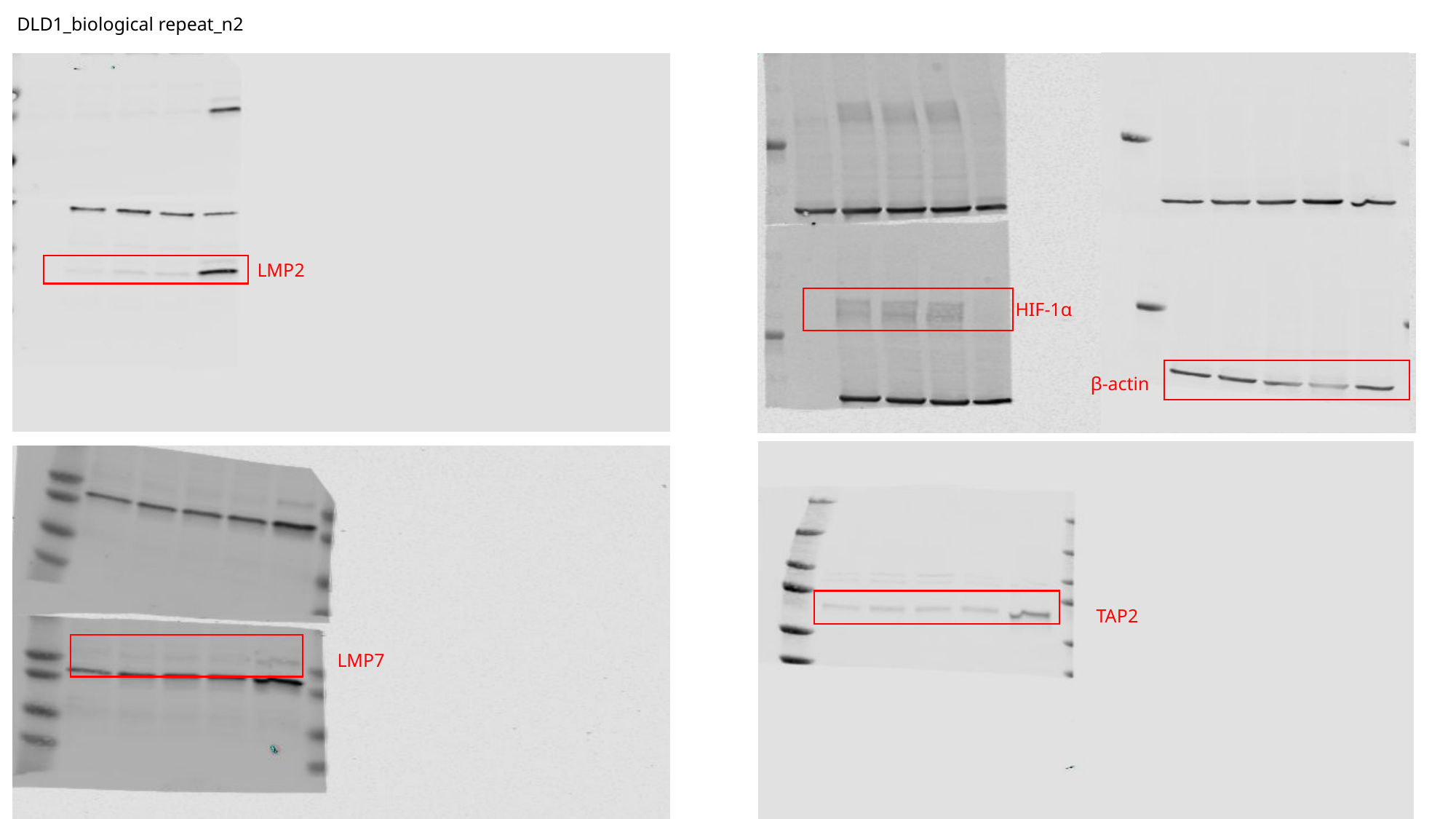

DLD1_biological repeat_n2
LMP2
HIF-1α
β-actin
TAP2
LMP7

## Slide 3
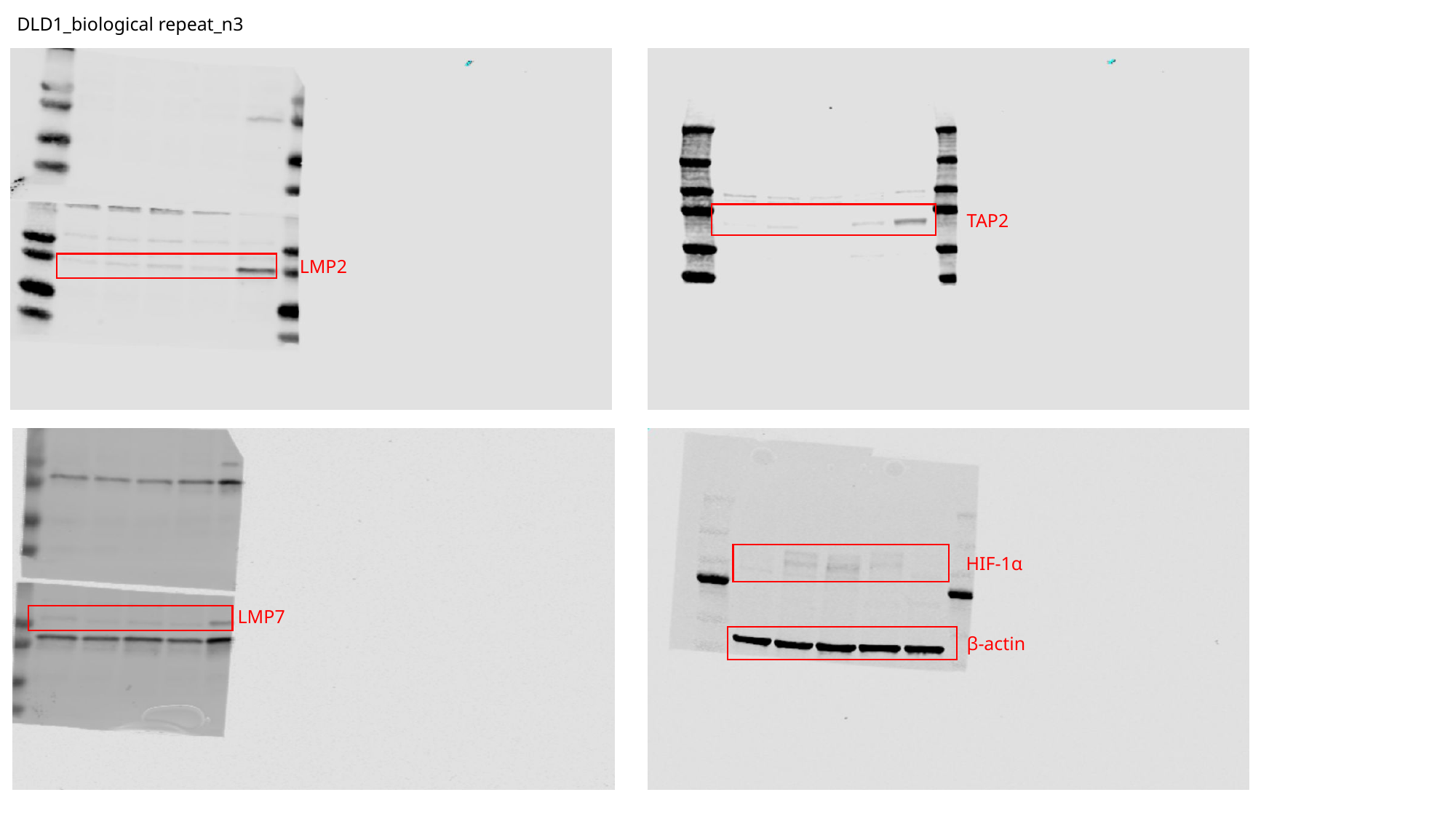

DLD1_biological repeat_n3
TAP2
LMP2
HIF-1α
LMP7
β-actin
